# Combinatorial activation of the WNT‐dependent fibrogenic program by distinct complement subunits in dystrophic muscle

**DOI:** 10.15252/emmm.202317405

**Published:** 2023-11-06

**Authors:** Francesca Florio, Sara Vencato, Filomena T Papa, Michela Libergoli, Eyemen Kheir, Imen Ghzaiel, Thomas A Rando, Yvan Torrente, Stefano Biressi

**Affiliations:** ^1^ Department of Cellular, Computational and Integrative Biology (CIBIO) University of Trento Trento Italy; ^2^ Dulbecco Telethon Institute at University of Trento Trento Italy; ^3^ Neurology Unit Fondazione IRCCS Ca' Granda Ospedale Maggiore Policlinico Milan Italy; ^4^ Broad Stem Cell Research Center University of California Los Angeles Los Angeles CA USA; ^5^ Stem Cell Laboratory, Dino Ferrari Center, Department of Pathophysiology and Transplantation University of Milan Milan Italy

**Keywords:** complement C1 complex, Duchenne muscular dystrophy, fibro‐adipogenic progenitors, fibrosis, skeletal muscle regeneration, Musculoskeletal System

## Abstract

Fibrosis is associated with compromised muscle functionality in Duchenne muscular dystrophy (DMD). We report observations with tissues from dystrophic patients and mice supporting a model to explain fibrosis in DMD, which relies on the crosstalk between the complement and the WNT signaling pathways and the functional interactions of two cellular types. Fibro‐adipogenic progenitors and macrophages, which populate the inflamed dystrophic muscles, act as a combinatorial source of WNT activity by secreting distinct subunits of the C1 complement complex. The resulting aberrant activation of the WNT signaling in responsive cells, such as fibro‐adipogenic progenitors, contributes to fibrosis. Indeed, pharmacological inhibition of the C1r/s subunits in a murine model of DMD mitigated the activation of the WNT signaling pathway, reduced the fibrogenic characteristics of the fibro‐adipogenic progenitors, and ameliorated the dystrophic phenotype. These studies shed new light on the molecular and cellular mechanisms responsible for fibrosis in muscular dystrophy and open to new therapeutic strategies.

## Graphical abstract

In Duchenne muscular dystrophy patients, a chronic condition of inflammation and degeneration is determining a progressive substitution of muscle fibers by fibrotic extracellular matrix. Based on the following observations we propose the involvement of the complement C1 complex in this process.

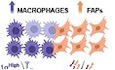

**The paper explained**

**Problem**

Fibrosis is associated with compromised muscle functionality in Duchenne muscular dystrophy. It contributes to the symptomatology of the disease and is a barrier to effective therapies. Elevated WNT signaling has been shown to play a detrimental role in muscle regeneration and promote the accumulation of fibrosis in dystrophic muscles. However, the molecular and cellular pathways responsible for this process are poorly characterized.

**Results**

We found increased levels of complement C1 in the skeletal muscles of both dystrophic patients and mice compared to healthy controls. We report here observations supporting the existence of a crosstalk between the complement and the WNT signaling pathways. Macrophages and fibro‐adipogenic progenitors are increased in the *MDX* muscles proximal to the *bona fide* regenerating areas and secreted distinct subunits of the C1 complex (i.e., C1q and C1r/s, respectively). Similar results were obtained in human muscles. The enhanced complement levels positively correlated with the increased expression of WNT‐target proteins in the *MDX‐*regenerating areas. Furthermore, C1 complex induced WNT signaling in murine FACS‐isolated fibro‐adipogenic progenitors, fibroblasts, and myoblasts *in vitro*. The *in vivo* pharmacological inhibition of C1r/s in the accelerated fib‐*MDX* murine model of muscular dystrophy led to the amelioration of the dystrophic phenotype (i.e., reduced expression of WNT signaling targets and fibrogenic genes in the dystrophic fibro‐adipogenic progenitor cells, and reduced collagen deposition in the muscle interstitium).

**Impact**

Our data support that complement is detrimental in the dystrophic environment and that inhibiting the classical complement pathway can reduce consequential tissue destruction. Our data provide evidence linking complement with degenerative disease and support the investigation of novel therapeutic strategies to delay the progression of Duchenne muscular dystrophy.

## Introduction

Duchenne muscular dystrophy (DMD) is one of the most severe and frequent forms of dystrophy (Emery, 2002; Mercuri *et al*, 2019). DMD patients show progressive dysfunction of skeletal and cardiac muscles (Emery, 2002; Mercuri *et al*, 2019). Although multidisciplinary care and glucocorticoid treatment are associated with reduced disease progression and improved patient survival, no definitive cure is currently available for DMD, and patients die by their third decade of life (Birnkrant *et al*, 2018; McDonald *et al*, 2018). DMD occurs due to mutations in the X‐chromosome dystrophin gene (O'Brien & Kunkel, 2001). The absence of functional dystrophin causes repetitive cycles of degeneration/regeneration of the muscle fibers. Portions of the dystrophic muscles (regeneration foci) continuously attempt to regenerate and are characterized by the infiltration of inflammatory cells (Ciciliot & Schiaffino, 2010). This chronic condition of inflammation and degeneration determines impairment of muscle repair potential, and fibrotic extracellular matrix progressively substitutes contractile fibers, determining a severe deficit of muscular function (Ciciliot & Schiaffino, 2010).

Upon damage, muscle regeneration is ensured by muscle satellite cells (MuSCs), a muscle‐specific stem cell population required to restore tissue functionality (Scharner & Zammit, 2011). MuSCs' activity is influenced by intrinsic and extrinsic factors (Sousa‐Victor *et al*, 2022). Particularly, MuSCs function is supported by a heterogeneous pool of cells occupying the interstitial space between fibers or associated with the vasculature (Wosczyna & Rando, 2018). Among them, mesenchymal cells, called fibro‐adipogenic progenitors (FAPs), characterized by the expression of PDGF receptor‐α (PDGFRα) and Sca1 critically influence muscle regeneration and homeostasis (Joe *et al*, 2010; Uezumi *et al*, 2010; Wosczyna *et al*, 2019). Moreover, a large body of evidence identified various subpopulations of immune cells as crucial mediators of effective muscle repair (Shen *et al*, 2008; Burzyn *et al*, 2013; Heredia *et al*, 2013; Lemos *et al*, 2015; Liu *et al*, 2017). Importantly, in diseased muscle, macrophages' fate is disturbed, and the communication between MuSCs and different subpopulations of inflammatory and interstitial cells is compromised (Tidball & Villalta, 2010; Mozzetta *et al*, 2013). These alterations are believed to contribute to the defective regeneration and promotion of fibrosis (Desguerre *et al*, 2009; Serrano & Munoz‐Canoves, 2010).

Multiple cell types aberrantly secrete extracellular matrix in the dystrophic setting, particularly collagens 1 and 3, which predominate in fibrotic DMD muscle (Foidart *et al*, 1981). MuSCs and cells belonging to the endothelial and hematopoietic lineage adopt fibrogenic features in DMD (Biressi *et al*, 2014; Pessina *et al*, 2015; Wang *et al*, 2016; Florio *et al*, 2022). Nevertheless, an increasing amount of evidence proposes FAPs as a major cellular source for fibrotic tissue in dystrophic muscle (Molina *et al*, 2021). A subset of PDGFRα^+ve^ stromal cells activated upon acute injury in the muscle reportedly gives rise to a significant fraction of collagen‐1‐overproducing cells generated during transient scarring, typical of the healing process (Dulauroy *et al*, 2012). Genetic ablation of this fraction is sufficient to limit interstitial collagen accumulation (Dulauroy *et al*, 2012). The accumulation of PDGFRα^+ve^ cells expressing fibrosis markers (i.e., Col1a1, Col3a1, and CTGF) in the diaphragm in the *MDX* dystrophic murine model supports the involvement of FAPs in the process of fibrosis not only during an acute injury but also in DMD (Uezumi *et al*, 2011). In keeping with this, interfering *in vivo* with the profibrotic PDGF‐signaling pathway alters FAPs activation and fibrosis (Ieronimakis *et al*, 2016; Mueller *et al*, 2016).

The canonical WNT signaling is progressively acquiring a central role in different tissues, including skeletal muscle (Cisternas *et al*, 2014). Increased canonical WNT signaling has been reported in muscles of the *MDX* mice and DMD patients (Trensz *et al*, 2010; Biressi *et al*, 2014; Liu *et al*, 2016). In *MDX* muscle, WNT signaling promotes collagen deposition by increasing the proliferation of resident Sca1^+ve^ cells (Trensz *et al*, 2010). This observation finds parallelism in the aging muscle, also characterized by WNT‐dependent defective regeneration and fibrosis (Brack *et al*, 2007; Naito *et al*, 2012). In both aging and dystrophic muscles, the activation of the canonical WNT signaling is associated with the acquisition of fibrotic features by MuSCs (Brack *et al*, 2007; Biressi *et al*, 2014). Particularly, MuSCs' loss of myogenic properties in dystrophic muscles depends on the presence of TGFβ, as the administration of a TGFβ‐blocking antibody was able to normalize the expression of myogenic and fibrotic markers (Biressi *et al*, 2014). The profibrotic role of WNT signaling is further corroborated by its ability to establish a reciprocal reinforcing crosstalk with the TGFβ‐signaling pathway, which is a powerful promoter of fibrosis (Girardi & Le Grand, 2018). On the one hand, the canonical WNT signaling was shown to promote the expression of TGFβ, particularly the TGFβ2 isoform (Carthy *et al*, 2011; Biressi *et al*, 2014). On the other hand, TGFβ facilitates the activation of the WNT signaling pathway by different mechanisms, which include the modulation of the secretion of the WNT antagonist Dickkopf‐1 and various WNT proteins or the involvement of microRNAs capable of targeting multiple components of the WNT/TGFβ axis (Akhmetshina *et al*, 2012; Blyszczuk *et al*, 2017; Seo *et al*, 2018).

DMD is characterized by chronic inflammation, and the involvement of cells and signaling cascades that are part of the innate immune system is just beginning to be investigated in dystrophic tissues (Porter *et al*, 2002; Tidball & Villalta, 2010; Tripodi *et al*, 2021). The role played by the complement cascade in this context is poorly understood (Engel & Biesecker, 1982; Sewry *et al*, 1987; Spuler & Engel, 1998). Intriguingly, recent reports indicate that in addition to its role as initiator of the classical complement cascade, the complement complex C1 is able to directly activate the canonical WNT signaling pathway (Naito *et al*, 2012; Sumida *et al*, 2015). This activation requires the presence of the C1q component of the complex (formed by C1qa, b, and c subunits codified by three genes aligned on the same chromosome) that binds to the frizzled WNT receptor and exposes the WNT co‐receptor LRP6 to the enzymatic activity of the two other subunits of the C1 complex, the C1r and C1s esterases (also codified by adjacent genes) (Kusumoto *et al*, 1988; Sellar *et al*, 1991; Naito *et al*, 2012). The result of this interaction is the cleavage of the extracellular domain of the WNT co‐receptor LRP that determines the activation of the WNT signaling pathway (Naito *et al*, 2012). Although several orders of magnitude weaker compared to the activation induced by the binding of the canonical WNT ligands to the Frizzled receptors, the activation of the WNT signaling pathway by the C1 complex is reportedly sufficient to promote defective regeneration and fibrosis in aging muscle (Naito *et al*, 2012). Given the even greater inflammation and disruption of regenerative potential in dystrophic muscle, we hypothesized the involvement of the complement C1/WNT axis in the progression of DMD.

We present data suggesting a causal link between the upregulation of complement C1 and the accumulation of fibrotic tissue typical of the dystrophic setting. Performing a molecular investigation in tissues from DMD patients and dystrophic animal models, we propose that distinct cell types (i.e., macrophages and FAPs) colonizing the inflamed dystrophic muscles can act as a combinatorial source of WNT activity by secreting distinct subunits of the C1 complement complex. The resulting aberrant activation of the complement C1/WNT axis in dystrophic muscles induces a cascade of events promoting fibrosis. Using an *in vivo* pharmacological approach in an accelerated murine model of DMD, we observed that halting the enzymatic activity of C1r/s normalizes the expression of fibrotic genes in FAPs and improves the muscle condition.

## Results

### Complement C1 is locally produced in dystrophic muscles

**Figure 1:**
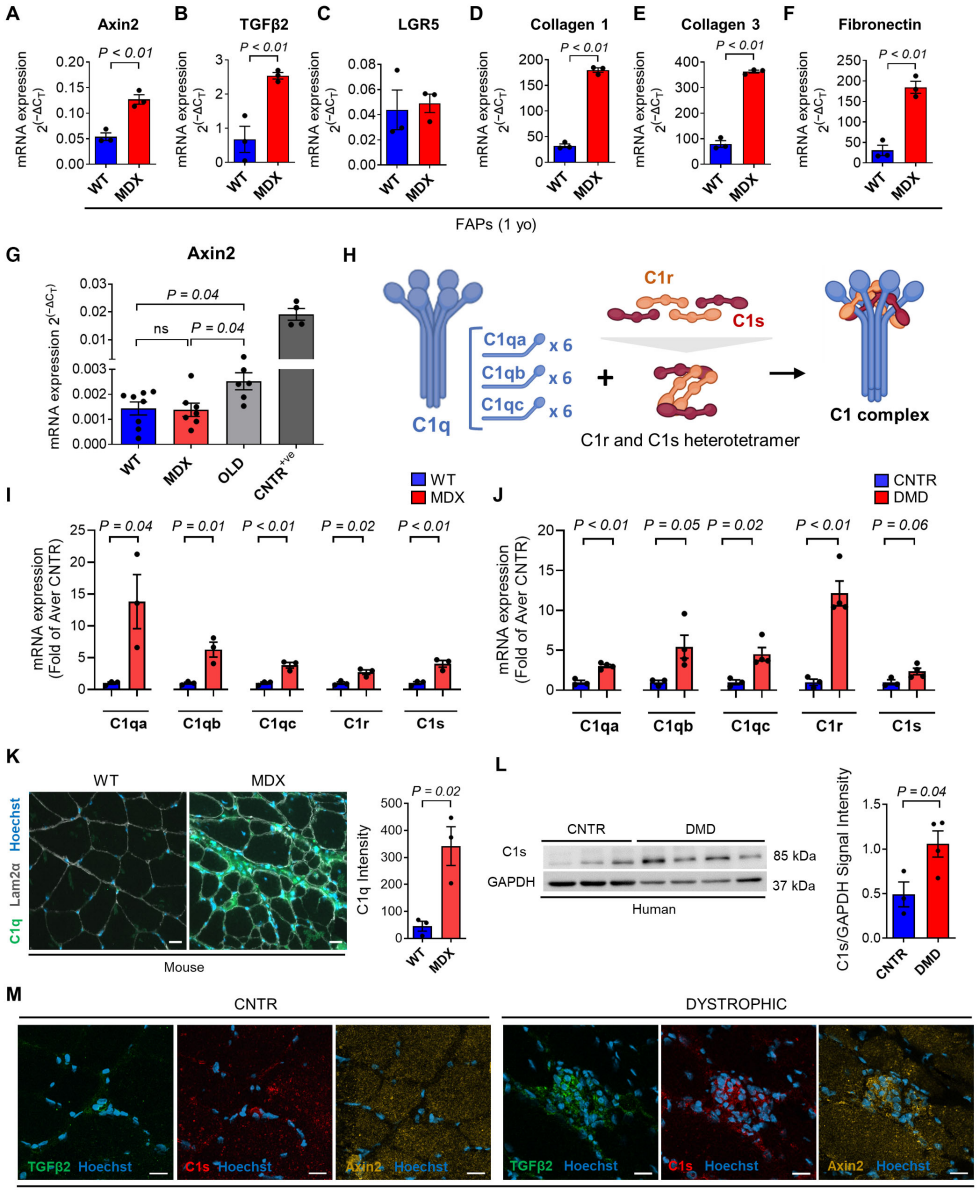
C1 is locally produced by skeletal muscles and is enhanced in dystrophy A–F
*Axin2* (A), *TGFβ2* (B), *LGR5* (C), *collagen 1a1* (D), *collagen 3a1* (E), and *fibronectin* (F) mRNA expression in FACS‐isolated FAPs from ~1‐year‐old *WT* and *MDX* hindlimb muscles. *N* (biological replicates) = 3.G
*Axin2* mRNA expression in C2C12 cultured for 6 h in DMEM with 5% serum collected from ~7‐months‐old *WT*, ~7‐months‐old *MDX*, and ~2.5‐years‐old *WT* (OLD) mice or with 50 ng/ml WNT3A (CNTR^+ve^). *N* (biological replicates) = 8 (*WT*), 7 (*MDX*), 6 (OLD), and 4 (CNTR^+ve^).HSchematic representation of C1 protein complex. Created with BioRender.com.I, J
*C1qa*, *C1qb*, *C1qc*, *C1r*, and *C1s* mRNA expression in hindlimb muscles of ~1‐year‐old *WT* and *MDX* mice (I) and biceps muscles of healthy controls (CNTR) and DMD patients (DMD) (J). *N* (biological replicates) = 3 (*WT*, *MDX*, CNTR) and 4 (DMD).KRepresentative immunofluorescence (left) and quantification (right) of C1q in the *gastrocnemius* of ~1‐year‐old *WT* and *MDX* stained with anti‐C1q (green), anti‐Laminin2α (gray) antibodies, and Hoechst (blue). Scale bar: 20 μm. *N* (biological replicates) = 3.LWestern blot (left) and quantification (right) of C1s in human healthy (CNTR) and dystrophic (DMD) biceps muscles. *N* (biological replicates) = 3 (CNTR) and 4 (DMD).MRepresentative immunofluorescence of serial sections of human healthy (CNTR) and dystrophic (DMD) biceps muscles stained with anti‐TGFβ2 (green), anti‐C1s (red), anti‐Axin2 (yellow), and Hoechst (blue). Note that TGFβ2, C1s, and Axin2 colocalize in the interstitial areas of DMD muscles. Scale bar: 20 μm. Data information: Data are presented as mean ± SEM. In (K) each graph dot represents the average value of 9 to 44 measurements on different muscle regions for each biological sample. Statistical differences were calculated in G by one‐way ANOVA test among *WT*, *MDX*, and *OLD* groups. Tukey's multiple‐comparison test was used as a *post hoc* test. In (A–F) and (I–L), statistical differences were calculated by unpaired two‐tailed Student's *t*‐test. *P*‐values are as indicated. Source data are available online for this figure.

**Figure EV1:**
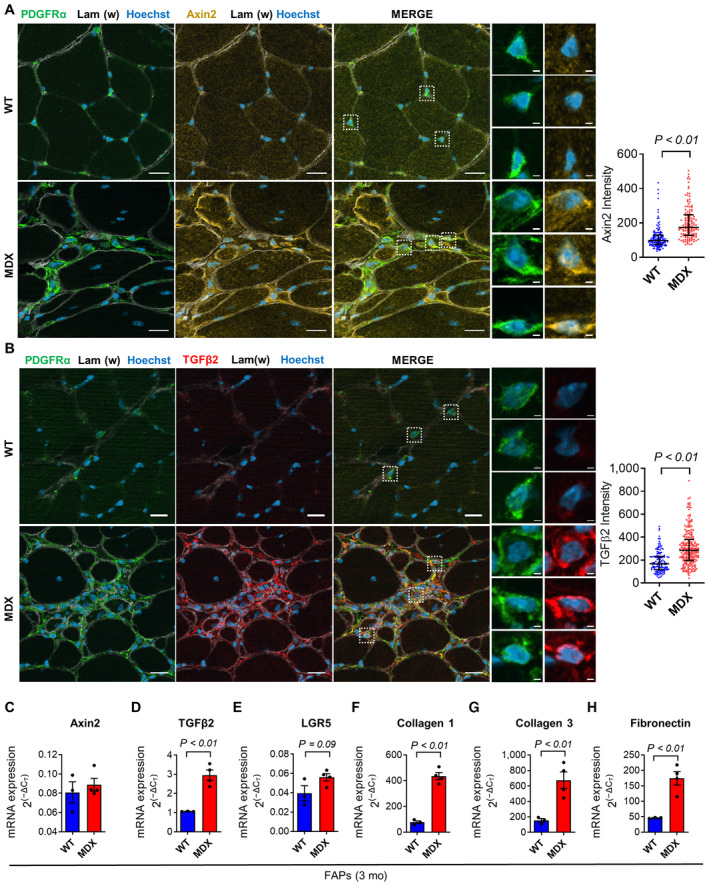
Canonical WNT signaling is increased in dystrophic FAPs A, BRepresentative immunofluorescence (left) and quantification (right) of Axin2 (A) and TGFβ2 (B) signal intensities in the PDGFRα^+ve^ cells (i.e., FAPs) of the *gastrocnemius* of ~1‐year‐old *WT* and *MDX* stained with anti‐Axin2 (yellow in A), anti‐PDGFRα (green), anti‐TGFβ2 (red in B), anti‐laminin 2α (white) antibodies, and Hoechst (blue). High‐magnification images in the left panels show representative PDGFRα^+ve^ cells. *N* (biological replicates) = 3 for all samples (except for *WT* TGFβ2 analysis, *N* = 2). Scale bar: 20 μm (low‐magnification images), scale bar: 2 μm (high‐magnification images).C–H
*Axin2* (C), *TGFβ2* (D), *LGR5* (E), *collagen 1a1* (F), *collagen 3a1* (G), and *fibronectin* (H) mRNA expression in FACS‐isolated FAPs from ~3‐months‐old *WT* and *MDX* hindlimb muscles. *N* (biological replicates) = 3. Data information: Data are presented as mean with interquartile range in (A and B) and as mean ± SEM in (C–H). In (A and B), dots represent single cells' measurements (131 for *WT* and 130 for *MDX* in Axin2 analysis, 126 for *WT* and 215 for *MDX* in TGFβ2 analysis). Statistical differences were calculated by unpaired two‐tailed Mann–Whitney test in (A and B) and by unpaired two‐tailed Student's *t‐*test in (C–H). *P*‐values are as indicated. Source data are available online for this figure.

Different research groups, including ours, have shown enhanced canonical WNT signaling pathway activity in both dystrophic and aged mice (Appendix Fig S1A) (Brack *et al*, 2007; Trensz *et al*, 2010; Naito *et al*, 2012; Biressi *et al*, 2014). An increment in canonical WNT signaling also characterizes the muscles of DMD patients, as disclosed by the quantification of known targets of the WNT signaling pathway (i.e., Axin2 and TGFβ2) in the interstitial space of human muscle biopsies (Appendix Fig S1B and C). Among the various cell types colonizing the muscle interstitium, FAPs are characterized by an active WNT signaling pathway in the *MDX* dystrophic environment, which parallels a robust transcription of fibrogenic markers, including collagens 1 and 3 (Figs 1A–F and EV1A–H).

**Figure EV2:**
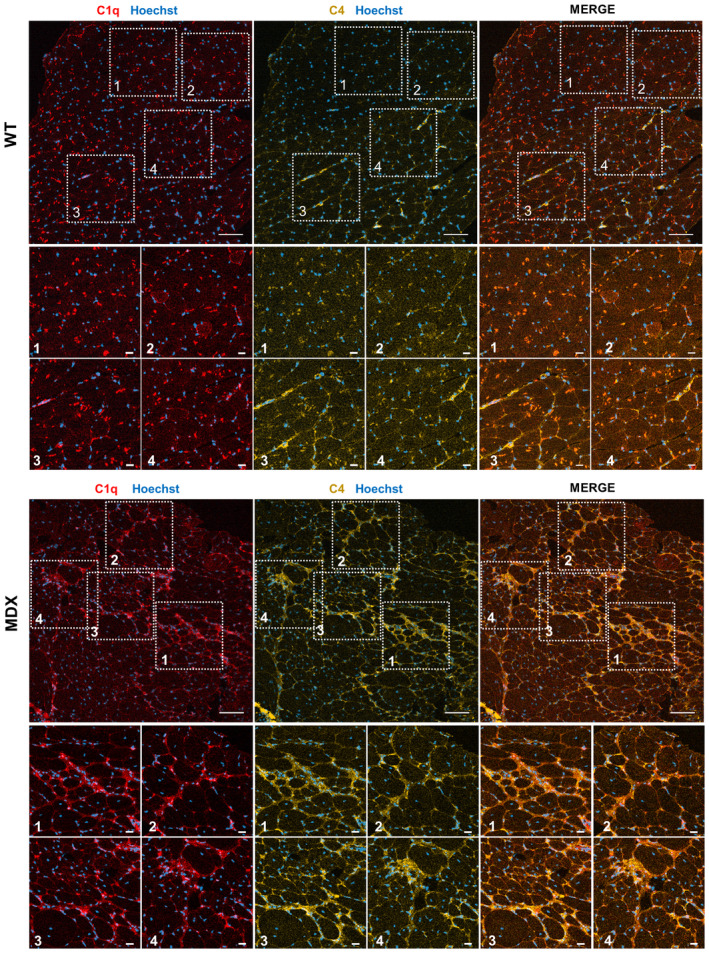
C1 and C4 complement levels are increased in dystrophic muscles Representative immunofluorescence of ~1‐year‐old *WT* and *MDX gastrocnemius* stained with anti‐C1q (red), anti‐C4 (yellow) antibodies, and Hoechst (blue). High‐magnification images represent different zoomed areas of the muscle. Note the expression of both C4 and C1q in the same muscle areas. Scale bar: 100 μm (low‐magnification images), scale bar: 20 μm (high‐magnification images). Source data are available online for this figure.

Increased levels of complement proteins in the serum have been reported to lead to increased activity of the WNT signaling pathway in old mice (Naito *et al*, 2012). To evaluate whether the factors that contribute to the enhanced WNT signaling in the dystrophic muscle have a serum origin, C2C12 myoblasts were cultured in the presence of serum collected from *MDX* and *WT* mice of different ages. The expression of the well‐established transcriptional target of canonical WNT signaling *Axin2* was evaluated by quantitative PCR (Fig 1G). While *Axin2* expression was increased in cells cultured with the serum collected from ~2.5‐years‐old mice compared to the ~7‐months‐old *WT* and *MDX* mice, its expression in cells cultured with the *MDX* serum was not significantly different from cells cultured with the age‐matched *WT* serum (Fig 1G). In line with this observation, only a negligible increase in the amount of C1q and C1s was observed in the *MDX* serum compared to the *WT* (Appendix Fig S2A and B). These data suggest that the events leading to increased WNT activity in dystrophic muscles differ from those occurring in aging tissues and likely do not depend on the release of complement C1 in the circulation. Therefore, we hypothesize that the increased WNT activity in dystrophic muscles could depend on the local production of molecules capable of initiating the WNT signaling pathway. We focused our attention on the complement C1 complex. C1 complex comprises C1q, which consists of C1qa, C1qb, and C1qc, and the heterotetramer C1s/C1r/C1r/C1s (Fig 1H). We found that all C1 components are actively transcribed in dystrophic muscles, and their mRNA levels are increased compared to the *WT* age‐matched controls (Fig 1I). Importantly, a similar pattern was observed in muscles from human dystrophic patients (Fig 1J and Appendix Table S1). Moreover, C1q and C1s protein expression resulted higher in dystrophic mice muscles compared to the *WT* controls in our immunofluorescence analysis (Fig 1K and Appendix Fig S3A). The overexpression of C1s protein and the activation of the C1/WNT axis (i.e., C1s, Axin2, and TGFβ2 expression) were confirmed in muscle biopsies from dystrophic patients compared to healthy controls (Fig 1L and M). In keeping with the overexpression of all components of the C1 complex, we observed the deposition of C4, an event occurring downstream to C1, in the interstitial space of *MDX* muscles (Fig EV2A). Our ELISA assay confirmed the presence of increased amounts of C4 in dystrophic mice muscles compared to the *WT* age‐matched counterparts (Appendix Fig S3B). These data indicate that the enhanced WNT signaling in the *MDX* mice is likely not caused by factors in the serum. The increased gene expression of C1 complex components in both dystrophic mice and patients suggests the local production of these proteins in the affected muscles.

### Distinct cell types express different C1 components in muscles

**Figure 2:**
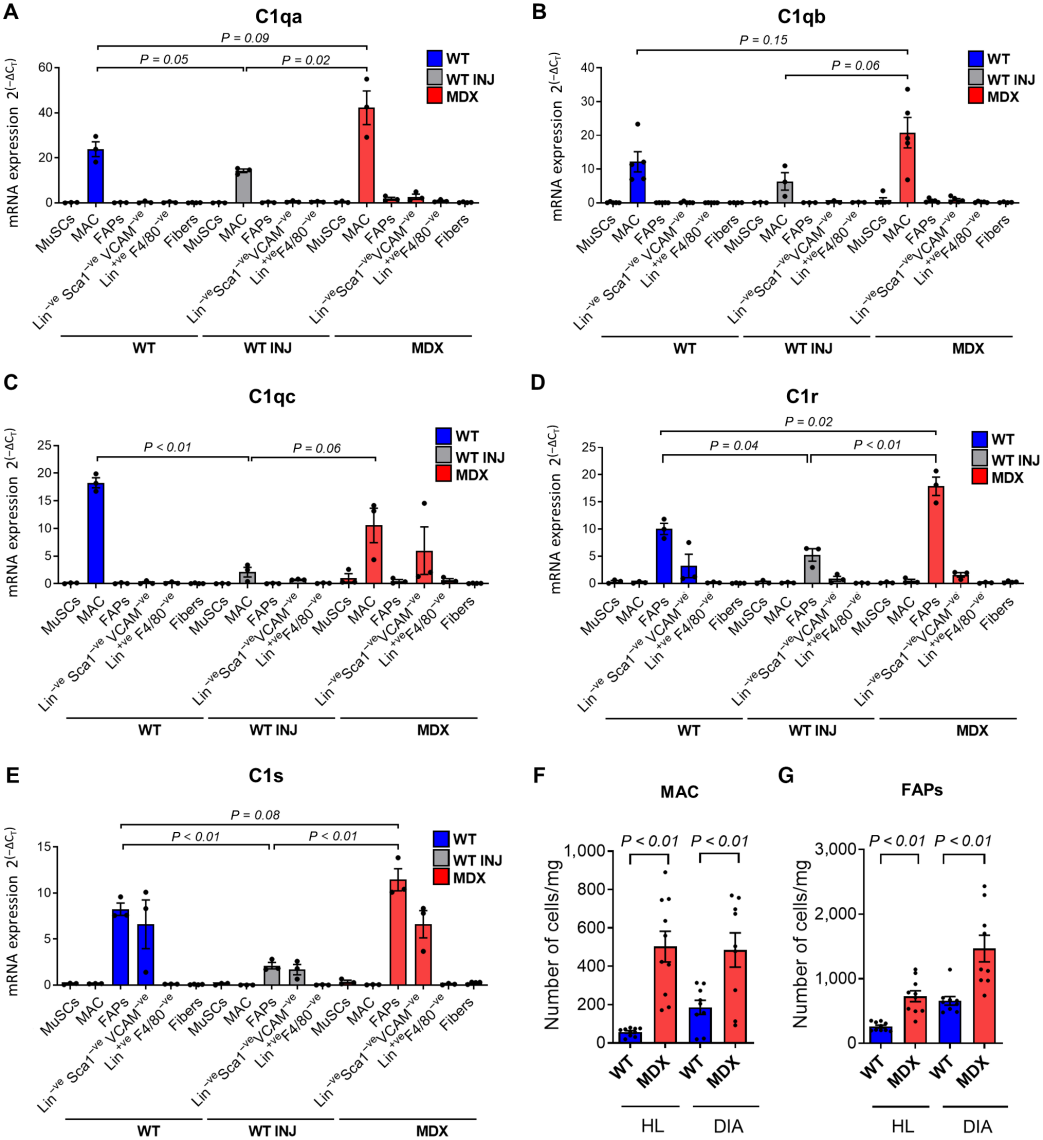
Distinct cell types express the C1 complex's components in the skeletal muscles A–E
*C1qa* (A), *C1qb* (B), *C1qc* (C), *C1r* (D), and *C1s* (E) mRNA expression in MuSCs, macrophages (MAC), FAPs, Lin^−ve^Sca1^−ve^VCAM^−ve^, Lin^+ve^F4/80^−ve^, and myofibers isolated from hindlimb muscles of ~1‐year‐old *WT* and *MDX* mice and muscles of *WT* mice 2.5 days after acute injury (*WT* INJ). *N* (biological samples) = 3 for all samples except for C1qb (*N* = 5) and fibers (*N* = 4).F, GNumber of macrophages (F) and FAPs (G) per mg of tissue in hindlimb (HL) and diaphragm (DIA) muscles of ~1‐year‐old *WT* and *MDX* mice. *N* (biological samples) = 10 (HL) and 9 (DIA). Data information: Data are presented as mean ± SEM. In (A–E), statistical differences between two groups were calculated by unpaired two‐tailed Student's *t*‐test, and the corresponding *P*‐values are reported on the graphs. Statistical differences between three or more groups were calculated by one‐way ANOVA test, Tukey's multiple‐comparison test was used as a *post hoc* test, and all the corresponding *P*‐values are enclosed in Appendix Tables S2 and S3. In (F, G) statistical differences between two groups were calculated by unpaired two‐tailed Student's *t*‐test. *P*‐values are as indicated. Source data are available online for this figure.

To further corroborate the idea of a local overproduction of complement C1 in dystrophic muscles, we investigated which muscle cell type/s express each of the five C1 subunits. We fractionated by FACS the mononucleated cells in skeletal muscle in five distinct and not overlapping subfractions: macrophages, FAPs, MuSCs, Lin^+ve^F4/80^−ve^ cells (i.e., endothelial cells and cells of hematopoietic lineage with the exclusion of mature macrophages), and Lin^−ve^Sca1^−ve^VCAM^−ve^ cells (i.e., all the remaining muscle mononucleated cells) (Appendix Fig S4A). We also enzymatically dissociated the muscles to obtain single myofibers. The analysis on *MDX* muscles was performed in parallel with uninjured *WT* muscles and *WT* muscles undergoing regeneration after an acute injury to highlight unique aspects associated with the chronic condition of regeneration typical of DMD. Our quantitative PCR analysis showed that: (i) *C1qa*, *C1qb*, and *C1qc* are uniquely expressed by macrophages in all three conditions (i.e., *WT*, *MDX*, and *WT* after injury) (Fig 2A–C and Appendix Table S2); and (ii) *C1r* and *C1s* are mostly expressed by FAPs and to a minor extent by Lin^−ve^Sca1^−ve^VCAM^−ve^ cells in all three conditions (Fig 2D and E, and Appendix Table S3). The population‐specific expression of C1 was confirmed at the protein level. C1q protein was observed in FACS‐isolated F4/80^+ve^ cells (macrophages) of *MDX* muscles through immunofluorescence but not in FAPs nor MuSCs isolated from the same muscles (Appendix Fig S5A). Moreover, the expression of C1q in macrophages was confirmed in *MDX* muscle sections (Appendix Fig S5B and C). Similarly, when we interrogated public human muscle transcripts datasets, C1q genes appeared uniquely expressed by macrophages, whereas C1r and C1s were enriched in *bona fide* FAPs (i.e., mesenchymal stem cells) (Appendix Fig S6A–F). We confirmed this pattern of expression at the protein level. C1q protein expression was higher in human hematopoietic cells (i.e., CD45^+ve^, which include macrophages) compared to FAPs (Appendix Fig S6G), whereas C1s protein expression was higher in human FAPs compared to both hematopoietic and endothelial cells (Appendix Fig S6H).

Intriguingly, our transcriptional analysis revealed also that: (i) *C1r* and *C1s* are less expressed in FAPs isolated from injured *WT* muscles compared to the *MDX* (Fig 2D and E); and (ii) *C1q* cluster genes are less expressed in macrophages isolated from injured *WT* muscles compared to the *MDX*, with *C1qa* expression reaching statistical significance and *C1qb* and *C1qc* exhibiting a trend (Fig 2A–C). Noteworthy, we found a similar expression of the cell proliferation marker gene *E2F1* in macrophages and FAPs isolated from *MDX* and injured *WT* muscles, suggesting that the differential C1 expression between dystrophic and acute degenerative conditions is unlikely depending on differences in the cellular proliferative state (Appendix Fig S7A) (Chen *et al*, 2009). In summary, different cell types within the skeletal muscles express the various C1 components. The cellular transcription of the C1 subunits is higher overall in dystrophic muscles than in muscles undergoing regeneration following a single acute injury event.

We next quantified the number of macrophages and FAPs to assess whether an increased number of these cell types may also contribute to the enhanced expression of complement levels in the dystrophic muscles (see Fig 1I–M, and Appendix Fig S3A and B). In agreement with previous reports, the number of both macrophages and FAPs was significantly increased in dystrophic muscles compared to the *WT* age‐matched controls when calculated both as total number of cells per mg of tissue, as well as percentage, respectively, of CD45^+ve^CD31^+ve^ cells and CD45^−ve^CD31^−ve^ cells (Fig 2F and G, and Appendix Fig S8A and B) (Tidball & Villalta, 2010; Contreras *et al*, 2016). Altogether our evaluation of the complement levels both at the gene and protein levels suggests that the increased C1 expression in dystrophic muscles is due to a combination of two factors: (i) an increased number of macrophages and FAPs in the dystrophic muscles, with these two cell types being, respectively, the main cellular sources of C1qa/b/c/ and C1r/s; (ii) increased production by the FAPs (for *C1r* and *C1s*) and macrophage (for *C1q*) populations in the dystrophic compared to the injured *WT* environment.

**Figure EV3:**
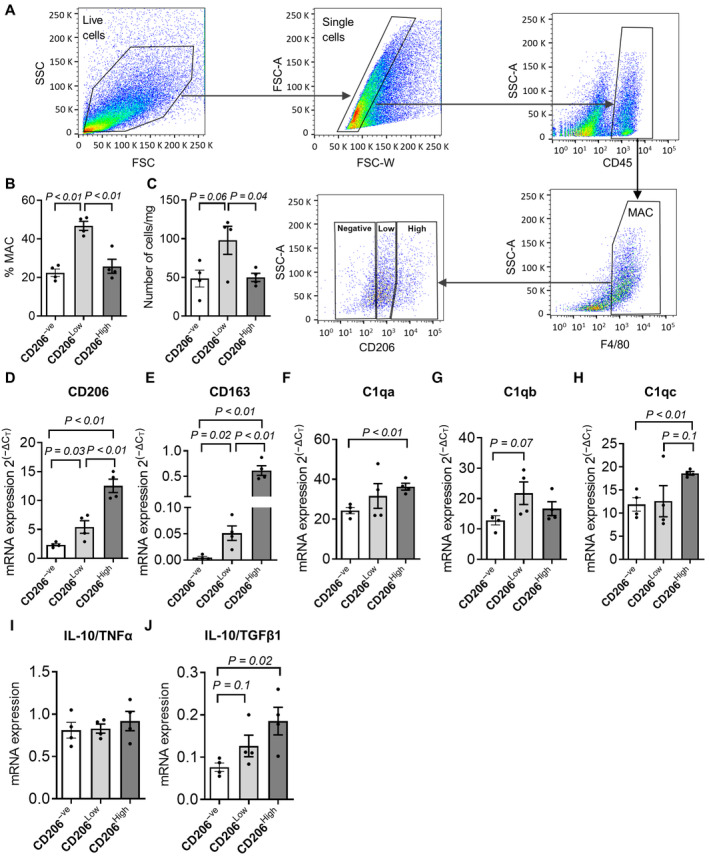
Complement expression in different subsets of macrophages in dystrophic muscles ARepresentative gating and sorting strategy used to FACS isolate different macrophage populations from *MDX* mice. Macrophages were gated as CD45^+ve^F4/80^+ve^ cells. Within the macrophage population, CD206^−ve^, CD206^Low^, and CD206^High^ subpopulations were isolated.B, CNumber of CD206^−ve^, CD206^Low^, and CD206^High^ macrophages from *MDX* mice FACS‐isolated as in (A). Data are expressed as the percentage of F4/80^+ve^ cells (B) and the number of cells per mg of tissue (C). *N* (biological samples) = 4.D–H
*CD206* (D), *CD163* (E), *C1qa* (F), *C1qb* (G), and *C1qc* (H) mRNA expression in CD206^−ve^, CD206^Low^, and CD206^High^ macrophages from *MDX* mice FACS isolated as in (A). *N* (biological samples) = 4.I, J
*IL‐10/TNFα* (I) and *IL‐10/TGFβ1* (J) mRNA expression in CD206^−ve^, CD206^Low^, and CD206^High^ macrophages from *MDX* mice FACS isolated as in (A). *N* (biological samples) = 4. Data information: Data are presented as mean ± SEM. Statistical differences between two groups were calculated by unpaired two‐tailed Student's *t‐*test. *P*‐values are as indicated. Source data are available online for this figure.

Differentiated macrophages (F4/80^+ve^) are divided by functional and molecular criteria into several subpopulations (Tidball & Villalta, 2010; Wang & Zhou, 2022). Our immunofluorescence analysis suggests that high levels of the C1q subunits are expressed only by a fraction of the whole macrophage population (Appendix Figs S5 and S6). To gain insight into this aspect, we interrogated publicly available murine and human single‐cell gene expression datasets and observed a correspondence between the fraction of C1q‐expressing macrophages and the subsets expressing CD163 or CD206 (Appendix Figs S6A–D and S9A–G). CD206 and CD163 are reportedly marking muscle‐resident or anti‐inflammatory subsets of macrophages (Villalta *et al*, 2009; Wang *et al*, 2020). Notably, the fraction of CD206^+ve^ macrophages expressing C1q appears to be distinct from the subset expressing the anti‐inflammatory marker IL‐10 (Appendix Fig S9H) (Purcu *et al*, 2022). To corroborate these observations, we FACS purified F4/80^+ve^ macrophages from dystrophic and acutely injured muscles, and we fractionated them based on the intensity of the CD206 staining (Fig EV3A and Appendix Fig S10A). Under both injury paradigms, CD206 is expressed in most macrophages, although with different intensities (Fig EV3B and Appendix Fig S10B). Notably, when we performed a gene expression analysis in the CD206^−ve^, CD206^Low^, and CD206^High^ macrophages, we could observe that only in the dystrophic environment the enhanced CD206 and CD163 expression in the CD206^Low/High^ subfractions correlates with an increase in the transcription of C1qa, C1qb, and C1qc (Fig EV3D–H and Appendix Fig S10D–H). The analysis of IL‐10, TNFα, and TGFβ1, which are, respectively, predominating in macrophages with anti‐inflammatory, inflammatory, and profibrotic functions, suggests that the CD206‐expressing macrophages in acutely injured muscles might be more anti‐inflammatory compared to their dystrophic counterpart (Fig EV3I and J, and Appendix Fig S10I and J) (Tidball & Villalta, 2010; Wang & Zhou, 2022). Altogether, these observations suggest that C1q subunits might be preferentially expressed in a CD206^+ve^CD163^+ve^ subset of macrophages that is not anti‐inflammatory and is enriched in dystrophic muscle.

### C1 activates the WNT signaling in fibroblasts, myoblasts, and FAPs

**Figure 3:**
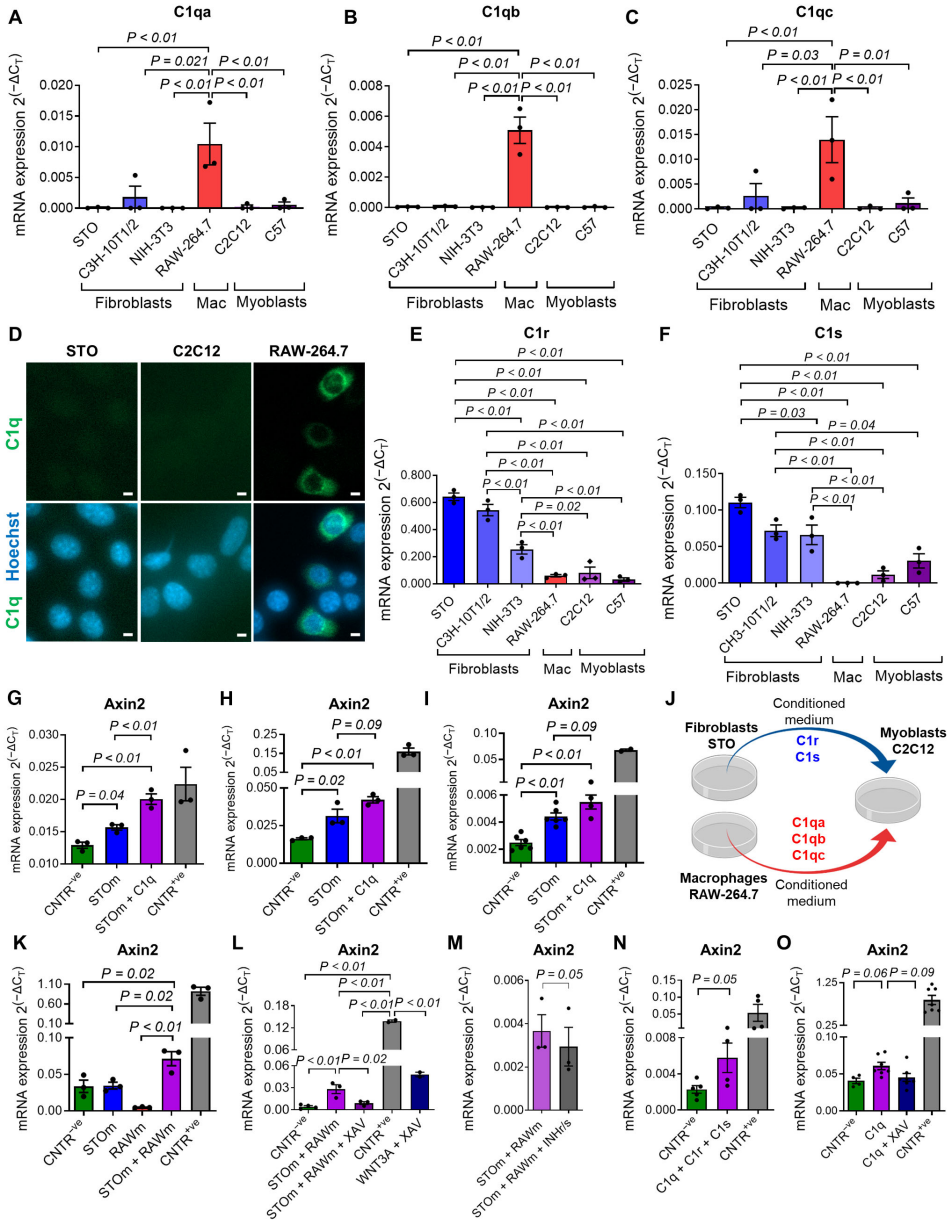
C1 activates the canonical WNT signaling in murine fibroblasts, myoblasts, and FAPs A–C
*C1qa* (A), *C1qb* (B), and *C1qc* (C) mRNA expression in cell lines as indicated (Mac: macrophages). *N* (independent experiments) = 3.DRepresentative immunofluorescence image of cells as indicated stained with an anti‐C1q (green) antibody and Hoechst (blue). C1q protein is expressed selectively by macrophages. Scale bar: 10 μm.E, F
*C1r* (E) and *C1s* (F) mRNA expression in cell lines as indicated. *N* (independent experiments) = 3.G–I
*Axin2* mRNA expression in STO (G, H) and C2C12 (I) cells cultured with STO‐conditioned medium (STOm) or STOm+C1q for 6 (G) or 24 (H, I) hours. *N* (biological samples) = 3 (G, H), 6 (CNTR^−ve^ and STOm in I), 4 (STOm + C1q in I), and 2 (CNTR^+ve^ in I). CNTR^+ve^: WNT3A 25 ng/ml in (G, H) and 100 ng/ml in (I). CNTR^−ve^: cell medium plus reagents' solvents.JScheme of the experiments shown in (K–M).K–N
*Axin2* mRNA expression in C2C12 cells cultured with cell media (CNTR^−ve^) or reagents as indicated for 24 (K, M), 6 (L), or 18 (N) hours. *N* (biological replicates) = 3 (K, M, STOm + RAWm, and STOm + RAWm + XAV in L), 4 (CNTR^−ve^ in L, C1q + C1r + C1s, and CNTR^+ve^ in N), 5 (CNTR^−ve^ in N), and 2 (CNTR^+ve^ and WNT3A + XAV in L). XAV: XAV939, INHr/s: C1r/s inhibitor. CNTR^−ve^: cell medium plus reagents' solvent. CNTR^+ve^: WNT3A 100 ng/ml (K) and 50 ng/ml (L, N).O
*Axin2* mRNA expression in FAPs freshly FACS isolated from *WT* mice 8 days after cardiotoxin injury and cultured with cell media (CNTR^−ve^) or reagents/inhibitors as indicated for 24 h. *N* (biological replicates) = 7 (except for CNTR^−ve^, *N* = 4). CNTR^−ve^: cell medium plus reagents/inhibitors' solvent. CNTR^+ve^: WNT3A 100 ng/ml. Data information: In (A–C), (E–I), and (K–O), data are presented as mean ± SEM. Statistical differences between three or more groups were calculated by one‐way ANOVA test, and Tukey's multiple‐comparison test was used as a *post hoc* test. The statistics were calculated by paired two‐tailed Student's *t*‐test in (M) and by unpaired two‐tailed Student's *t*‐test in (N). *P*‐values are as indicated. Source data are available online for this figure.

We tested C1 expression in murine fibroblasts (i.e., STO, C3H‐10T1/2, and NIH‐3T3), macrophages (i.e., RAW‐264.7), and myoblasts (i.e., C2C12 and primary myoblasts) in order to establish an *in vitro* model that recapitulates the differential expression of C1 components observed *in vivo*. Similar to our analysis of freshly isolated cells (Fig 2A–E, and Appendix Tables S2 and S3), we observed that *C1qa*, *C1qb*, and *C1qc* are mostly transcribed by macrophages (Fig 3A–C); C1q protein expression was observed through immunofluorescence in a fraction of macrophages, but not in fibroblasts, nor in myoblasts (Fig 3D). *C1s* and *C1r* are mostly expressed by fibroblasts (Fig 3E and F), and myoblasts express only traces of all C1 components (Fig 3A–C, E and F).

We next studied C1‐mediated modulation of the canonical WNT signaling *in vitro* in three different cell types: (i) fibroblasts, which express high levels of C1s and C1r similarly to the FAPs in muscles (Fig 3E and F), (ii) myoblasts that express low levels of C1 similarly to satellite cells and myofibers (Fig 3A–F), and (iii) muscle‐isolated FAPs. All C1 subunits are secreted proteins (Gulati *et al*, 1994). We used fibroblasts' (STO) and macrophages' (RAW‐264.7) conditioned media (expected to be, respectively, enriched in C1r/C1s and C1q proteins) to treat STO fibroblasts and C2C12 myoblasts. An increased expression of *Axin2* was observed in STO treated with STO medium supplemented with recombinant C1q compared to cells treated with STO medium alone or with the unconditioned medium (Fig 3G and H). An analogous trend was observed when C2C12 was treated similarly (Fig 3I). An increased expression of *Axin2* was also observed in C2C12 myoblasts treated with the combination of STO and RAW‐264.7 media compared to the same cells treated with just one of the two media (Fig 3J and K). *Axin2* increased expression was rescued in C2C12 cells treated with XAV‐939, a well‐known inhibitor of canonical WNT signaling (Fig 3L). A partial but statistically significant reduction was observed when a C1 inhibitor was added, indicating that at least part of the WNT activity depends on C1 components secreted in the media (Fig 3M). Myoblasts were treated with recombinant C1q, C1r, and C1s proteins, and an increased level of *Axin2* was observed compared to the cells cultured with the control solution, further suggesting that C1 might be the factor leading to an increased WNT signaling in these cells (Fig 3N). Finally, the supplementation of C1q was able to increase *Axin2* expression also in muscle‐isolated FAPs, and the addition of XAV‐939 blocked this response (Fig 3O). Altogether, these *in vitro* observations suggest that the formation of the C1 complex has the potential to enhance the activity of the canonical WNT signaling in various cell types, including FAPs. These data further support the idea that the different subunits of the C1 complex are released by different cell types and act as a combinatorial source of WNT signaling.

### Increased macrophages, FAPs, and C1/WNT axis in the *MDX*

**Figure 4:**
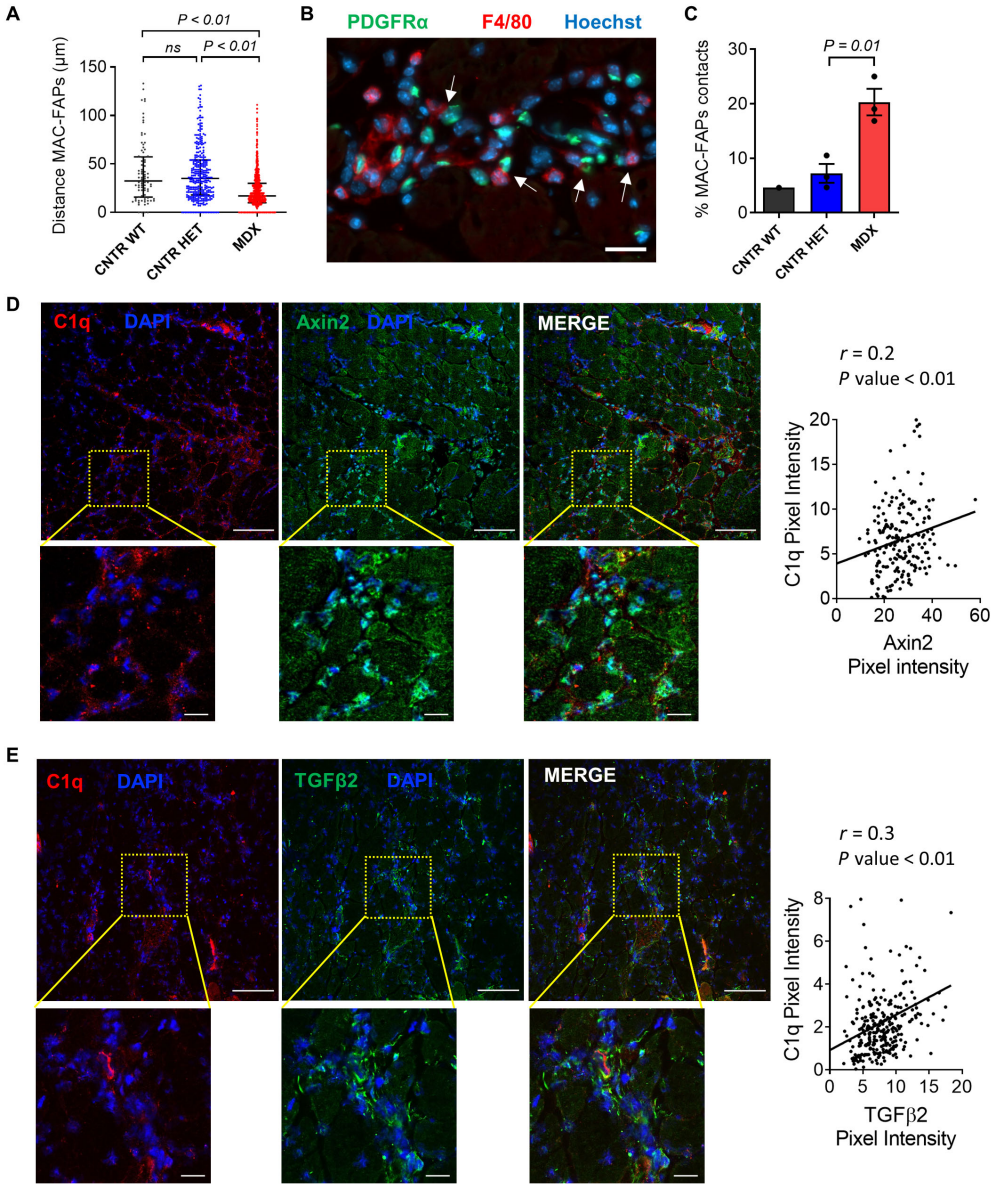
Enhanced C1/WNT axis in the dystrophic regenerating foci ADistance (μm) between macrophages (MAC) and FAPs in *gastrocnemius* of ~12‐months‐old male *PDGFRα* ^*eGFP/WT*^ (CNTR *WT*), ~18‐months‐old female *PDGFRα* ^*eGFP/WT*^;*MDX* ^+/−^ (CNTR HET), and ~18‐months‐old male *PDGFRα* ^*eGFP/WT*^;*MDX* (*MDX*) mice. The analysis was performed by measuring the distance of each macrophage in the image field from its closest FAP. *N* (biological replicates) = 3 (CNTR HET, *MDX*) and 1 (CNTR *WT*).BRepresentative immunofluorescence of a *bona fide* regenerating area in the *gastrocnemius* of a ~18‐months‐old male *PDGFRα* ^*eGFP/WT*^;*MDX* mouse stained with anti‐F4/80 (red), anti‐GFP (green) antibodies, and Hoechst (blue). White arrows indicate contacts between macrophages (F4/80^+ve^) and FAPs (eGFP^+ve^). Scale bar: 20 μm.CPercentage of contacts (i.e., distance of 3 μm or less) between macrophages and FAPs calculated in same muscles as in (A). *N* (biological replicates) = 3 (CNTR HET, *MDX*) and 1 (CNTR *WT*).D, ERepresentative immunofluorescence of *gastrocnemius* of ~1‐year‐old *MDX* stained with anti‐C1q (red), anti‐Axin2 (green in D), anti‐TGFβ2 (green in E) antibodies, and DAPI (blue). Scale bar: 100 μm (low‐magnification images) and 20 μm (high‐magnification images). The positive correlation between C1q and Axin2 (D) and C1q and TGFβ2 (E) intensity values is shown. *N* (biological replicates) = 3. Data information: In (A) data are presented as median with interquartile range. Each dot on the graph represents a distance measurement (86 for *WT*, 335 for CNTR *HET*, and 827 for *MDX*). Statistical differences were calculated by the Kruskal–Wallis test. Dunn's multiple‐comparison test was used as a *post hoc* test. In (C) data are presented as mean ± SEM. Statistical differences between CNTR *HET* and *MDX* groups were calculated by unpaired two‐tailed Student's *t‐*test. In (D and E) each graph dot represents a measurement of a muscle area (196 in D and 289 in E). The positive correlation between C1q and Axin2/TGFβ2 was assessed using two‐tailed correlation test and computing the Spearman coefficient (*r*) for Axin2 (in D) or TGFβ2 (in E) versus C1q dataset. *P*‐values are as indicated. Source data are available online for this figure.

In order to get insights into the possibility that macrophages and FAPs may act in combination to induce WNT signaling *in vivo*, we first studied the localization of FAPs and macrophages within the dystrophic muscles. Both these cells are required for the formation of C1 as they, respectively, release C1r/s and C1q. They are, therefore, expected to be physically close in the *MDX*‐regenerating foci. To identify FAPs, we used the *PDGFRα* ^*eGFP*^ reporter strain that has the H2B‐eGFP fusion protein knocked into the *PDGFRα* locus and has been previously used to mark FAPs in muscles (Mueller *et al*, 2016). Our FACS and immunofluorescence analysis performed on muscles collected from *PDGFRα* ^*eGFP/WT*^;*MDX* mice confirmed that the eGFP^+ve^ population overlaps with the interstitial FAPs cell population (Appendix Fig S11A and B). Muscles from dystrophic *PDGFRα‐eGFP*;*MDX* males were stained with anti‐F4/80 antibody to mark macrophages, and the distance between macrophages and FAPs was measured (Fig 4A). Muscles from *PDGFRα* ^*eGFP/WT*^;*MDX* ^+/−^ heterozygous female carriers and non‐dystrophic *PDGFRα* ^*eGFP/WT*^ mice were used as asymptomatic controls (Fig 4A). Clusters of macrophages and FAPs were observed in the muscle areas characterized by high cellularization (i.e., in *bona fide* regenerative foci) of the dystrophic *PDGFRα* ^*eGFP/WT*^;*MDX* males (Fig 4B). Both macrophages‐to‐FAPs and FAPs‐to‐macrophages distances were measured, demonstrating that macrophages and FAPs are closer to each other in dystrophic muscles than in controls (Fig 4A and Appendix Fig S11C–F). Moreover, dystrophic *PDGFRα* ^*eGFP/WT*^;*MDX* muscles showed a higher percentage of macrophages and FAPs direct contacts compared to the controls (Fig 4B and C).

We showed in this study that macrophages and FAPs are increased in dystrophic muscles and near each other in the regenerative foci of dystrophic muscles, where they can potentially promote WNT activity by secreting distinct subunits of the C1 complex. To corroborate this view, we investigated whether the increased presence of these cells correlates with the enhanced WNT signaling activity. We found an increased expression of the profibrotic WNT‐target TGFβ2 in the regenerating areas of dystrophic mice, which were also characterized by an increased number of macrophages and FAPs compared to the non‐dystrophic controls (Appendix Fig S12A; Biressi *et al*, 2014). Similar to TGFβ2, we also found that the canonical WNT signaling effectors/target proteins β‐catenin colocalize with F4/80^+ve^ cells in the regenerating areas of *MDX* muscles (Appendix Fig S12B and C). Moreover, regions within the regenerating areas of *MDX* muscles that were characterized by strong expression of Axin2 or TGFβ2 also exhibited a strong expression of C1q protein (Fig 4D and E). The positive correlation between C1q/Axin2 and C1q/TGFβ2 intensities is consistent with the possibility that the augmented complement contributes to the increased WNT/TGFβ axis reported in dystrophy (Biressi *et al*, 2014).

### Increased WNT signaling and fibrosis in the fib‐*MDX* mouse FAPs

**Figure 5:**
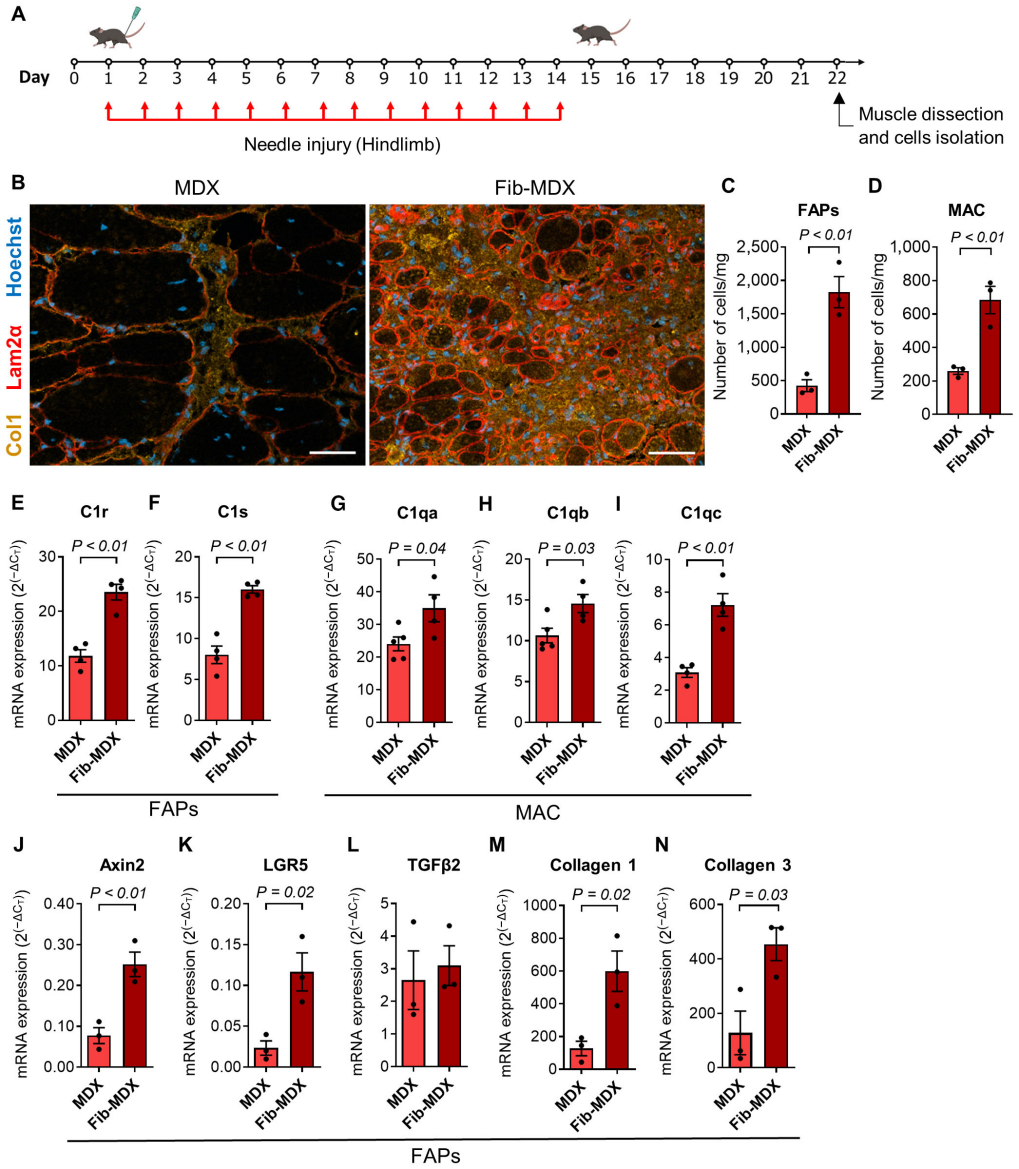
The fib‐*MDX* mouse is a valuable model for studying FAPs in dystrophy AScheme of the generation of the fib‐*MDX* mouse model.BRepresentative immunofluorescence image of a *gastrocnemius* of a ~1‐year‐old *MDX* and fib‐*MDX* stained with anti‐collagen 1 (yellow), anti‐laminin 2α (red) antibodies, and Hoechst (blue). Scale bar: 50 μm.C, DNumber of FAPs (C) and macrophages (D) per mg of tissue in the hindlimb of fib‐*MDX* or uninjured *MDX* muscles. *N* (biological samples) = 3.E–I
*C1r* (E), *C1s* (F), *C1qa* (G), *C1qb* (H), and *C1qc* (I), mRNA expression in FAPs (E, F) and macrophages (G–I) FACS isolated from fib‐*MDX* or uninjured *MDX* hindlimb muscles. *N* (biological samples) = 4 (for all samples except *MDX* C1qa and *MDX* C1qb, *N* = 5).J–N
*Axin2* (J), *LGR5* (K), *TGFβ2* (L), *collagen 1a1* (M), and *collagen 3a1* (N) mRNA expression in FAPs FACS isolated from fib‐*MDX* or uninjured *MDX* hindlimb muscles. *N* (biological samples) = 3. Data information: In (C–N) data are presented as mean ± SEM. Statistical differences were calculated by unpaired two‐tailed Student's *t‐*test. *P*‐values are as indicated. Source data are available online for this figure.

**Figure EV4:**
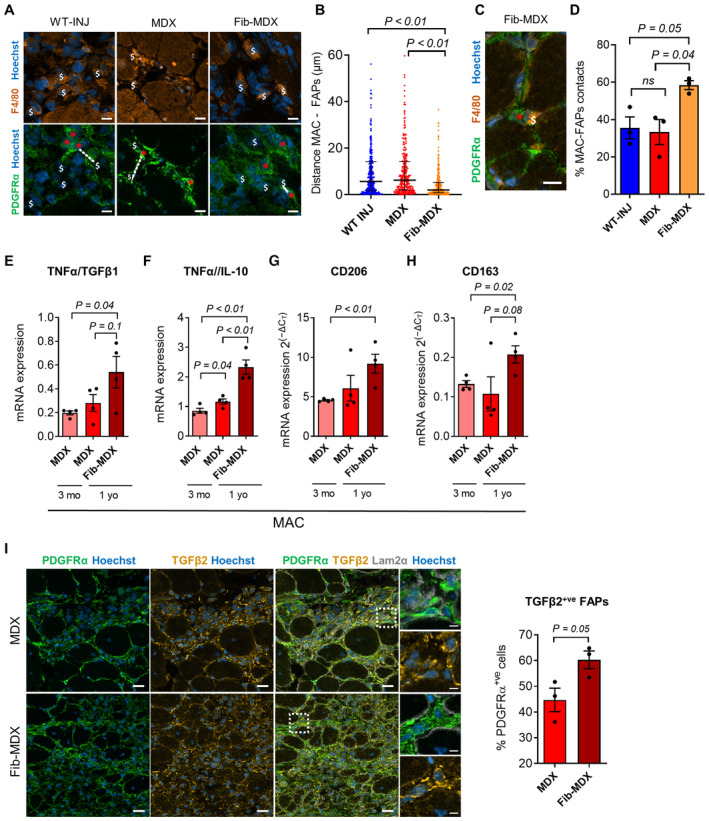
The fib‐*MDX* skeletal muscle: macrophages/FAPs interplay ARepresentative immunofluorescence of *gastrocnemius* of ~1‐year‐old *WT* 4 days after cardiotoxin injury (*WT* INJ), *MDX*, and fib‐*MDX* mice stained with anti‐PDGFrα (green), anti‐F4/80 (orange) antibodies, and Hoechst (blue). White dollar symbols indicate macrophages (i.e., F4/80^+ve^ cells), red asterisks indicate FAPs (i.e., PDGFRα^+ve^ cells), and dotted lines indicate examples of distance measured between macrophages and FAPs. Scale bar: 10 μm.BDistance (μm) between macrophages (MAC) and FAPs measured in the *gastrocnemius* of ~1‐year‐old *WT* 4 days after cardiotoxin injury (*WT* INJ), *MDX*, and fib‐*MDX* mice stained as in (A). *N* (biological samples) = 3.CExample of a contact between a macrophage and a FAP in muscles represented in (A). Scale bar: 10 μm.DPercentage of contacts (i.e., a distance of 3 μm or less) between macrophages and FAPs calculated in the same muscles as in (A). *N* (biological samples) = 3.E, F
*TNFα/TGFβ1* (E) and *TNFα/IL‐10* (F) mRNA expression in F4/80^+ve^ macrophages FACS isolated from ~3‐months‐old *MDX*, ~1‐year‐old *MDX*, and ~1‐year‐old fib‐*MDX* hindlimb muscles. *N* (biological replicates) = 4.G, H
*CD206* (G) and *CD163* (H) mRNA expression in macrophages FACS isolated as in (E and F). *N* (biological replicates) = 4.IRepresentative immunofluorescence (left) and quantification (right) of the TGFβ2^+ve^ FAPs (i.e., PDGFRα^+ve^ and TGFβ2^+ve^ cells) expressed as percentage of the total number of FAPs (i.e., PDGFRα^+ve^ cells) in the *gastrocnemius* of ~1‐year‐old *MDX* and fib‐*MDX* mice stained with anti‐PDGFrα (green), anti‐TGFB2 (yellow), anti‐Laminin2α (gray) antibodies, and Hoechst (blue). *N* (biological samples) = 3. Scale bar: 20 μm (low‐magnification images), scale bar: 5 μm (high‐magnification images). Data information: In (B) data are presented as median with interquartile range. Each dot on the graph represents a distance measurement (307 for *WT* INJ, 261 for *MDX*, and 303 for fib‐*MDX*). Statistical differences were calculated by the Kruskal–Wallis test. Dunn's multiple–comparison test was used as a *post hoc* test. In (D–I), data are presented as mean ± SEM. In (I) each graph dot represents the percentage of TGFβ2^+ve^ cells calculated in 344 to 412 (*MDX*) and 315 to 617 (fib‐*MDX*) FAPs randomly selected in different muscles' interstitial regions for each biological sample. Statistical differences between two groups were calculated in (E–I) by unpaired two‐tailed Student's *t‐*test and between three groups in (D) by one‐way ANOVA test using Tukey's multiple‐comparison test as a *post hoc* test. *P*‐values are as indicated. Source data are available online for this figure.

The fib‐*MDX* mouse model is a dystrophic mouse that overcomes some limitations associated with the mild pathological phenotype of the *MDX* mice compared to the human disease (Desguerre *et al*, 2012; McGreevy *et al*, 2015). To get access to muscles that closely recapitulate the severity of the human pathology, we tested the fib‐*MDX* mouse model by performing daily micro‐injuries in the distal muscles of the hindlimb of *MDX* mice for 2 weeks (Fig 5A). This procedure leads to a dramatic alteration of muscle histology and deposition of interstitial collagen (Fig 5B and Appendix Fig S13A). Notably, we could observe the accumulation of FAPs in the collagen‐rich interstitium of the fib‐*MDX* muscles (Appendix Fig S13B). To investigate the C1/WNT axis in the fib‐*MDX* mouse model, we quantified the number of C1‐producing cells. The number of both FAPs and macrophages was increased in the fib‐*MDX* compared to the uninjured *MDX* muscles (Fig 5C and D, and Appendix Fig S13C and D). This increment parallels a further increase in the proximity between FAPs and macrophages in the fib‐*MDX* model in comparison to the classic *MDX* and to acutely cardiotoxin‐injured *WT* muscles (Fig EV4A–D). Moreover, when we isolated FAPs from the fib‐*MDX*, we could observe an increase in the transcription of C1r and C1s subunits compared to the classic *MDX* mice (Fig 5E and F). A similar result was also obtained with the three C1q subunits in F4/80^+ve^ macrophages (Fig 5G–I). Intriguingly, when we further characterized macrophages from the fib‐*MDX* for the expression of know‐heterogeneity markers, we could observe not only a general shift toward a more proinflammatory phenotype (i.e., characterized by a predominance of TNFα) compared to the classic *MDX* but also an upregulation of CD206 and CD163 that are marking a fraction of macrophages enriched for C1q expression in dystrophic muscle (Fig EV4E–H).

Next, we wondered if the increase in C1‐producing cells and the increment in the expression of C1 subunits observed in the fib‐*MDX* model could lead to enhanced activation of the WNT signaling pathway and fibrogenic program. FAPs and MuSCs were FACS isolated 1 week after the last injury and analyzed for the expression of both canonical WNT signaling targets (i.e., *Axin2*, *TGFβ2*, and *LGR5*) and fibrosis‐related genes (i.e., *collagen1a1* and *collagen3a1*). Interestingly, Axin2, LGR5, collagen 1a1, and collagen 3a1 transcripts were strongly increased in the FAPs of the fib‐*MDX* muscles compared to the classic uninjured *MDX* muscles (Fig 5J–N). Only a trend was observed in the expression of TGFβ2, collagen 1a1, and collagen 3a1 in MuSCs (Appendix Fig S14C–E). Moreover, immunofluorescence analysis revealed that the percentage of FAPs expressing the profibrotic WNT‐target TGFβ2 was increased in the fib‐*MDX* model (Fig EV4I). On the one hand, these data are in line with increased inflammation, the augmented activity of the C1/WNT axis and extracellular matrix deposition, and a more severe dystrophic phenotype occurring in fib‐*MDX* mouse model compared to the classical *MDX* mice (Desguerre *et al*, 2012). On the other hand, these observations candidate the fib‐*MDX* mice as a valuable model to study the profibrotic role of FAPs.

To get further insights into the nature of the cells responsive to the C1q‐mediated induction of the WNT signaling in dystrophic muscle, we tested the expression of the WNT co‐receptor LRP6, which reportedly mediates the C1‐dependent activation of the WNT signaling pathway in aging (Naito *et al*, 2012). LRP6 was found to be expressed in MuSCs, macrophages, FAPs, Lin^−ve^Sca1^−ve^VCAM^−ve^, Lin^+ve^F4/80^−ve^, and isolated fibers, both in the *WT* and dystrophic muscles (Appendix Fig S14F). However, despite MuSCs and FAPs expressing comparable amounts of TGFβ2 (Appendix Fig S14G), FAPs had the highest expression of LRP6 among all the dystrophic cells, suggesting their major contribution to the enhanced canonical WNT signaling in dystrophy (Appendix Fig S14F). In line with its expression in freshly isolated cells, LRP6 was also more expressed in fibroblasts (STO and C3H‐10T1/2) compared to myoblast (C2C12) cell lines (Appendix Fig S14F). Altogether, these observations ascribe to FAPs a central role in the profibrotic events that are operating downstream to the C1/WNT axis in muscular dystrophy, as revealed by the fib‐*MDX* model.

### C1r/s inhibition alleviates dystrophy in the fib‐*MDX* model

**Figure 6:**
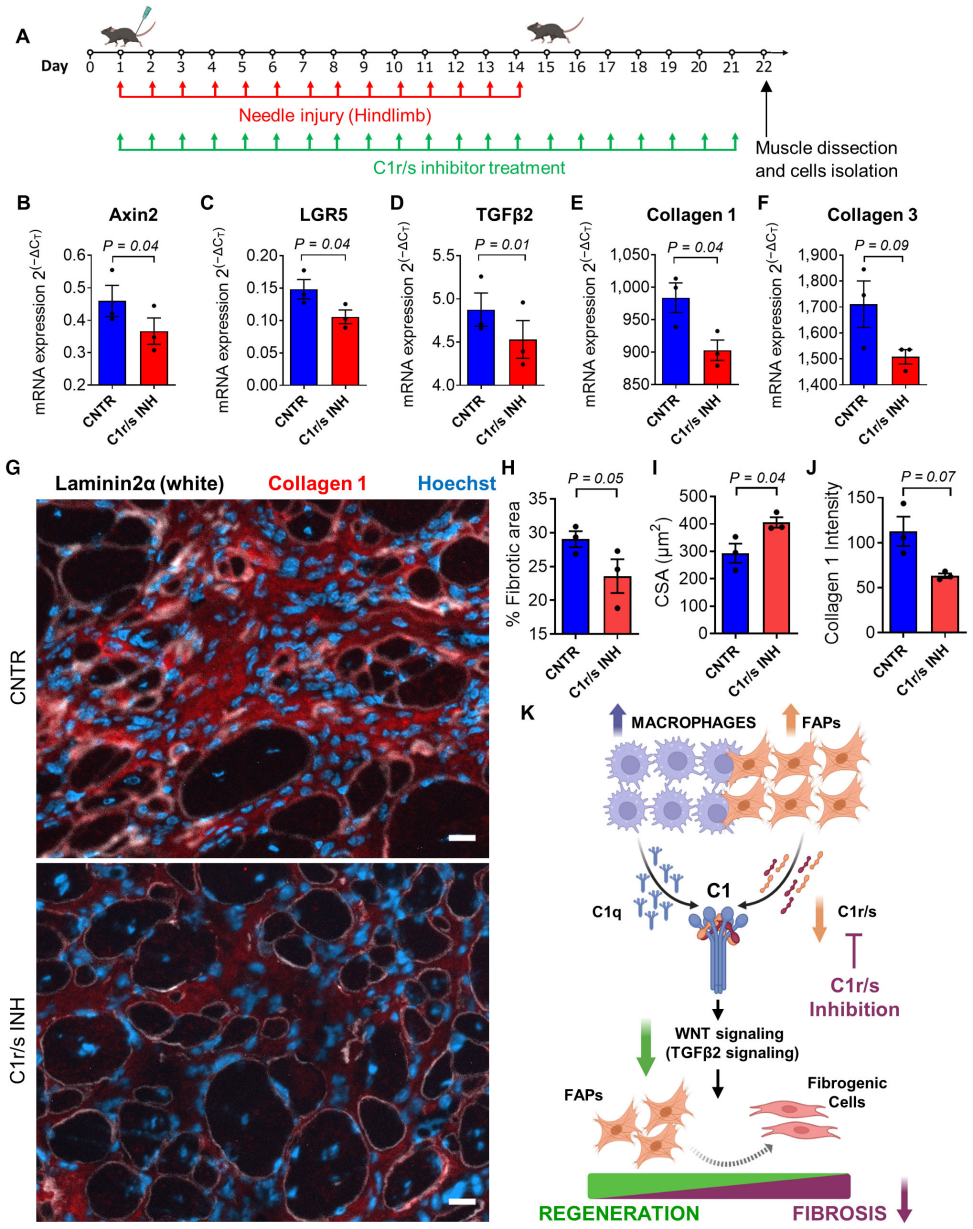
*In vivo* inhibition of C1r/s rescues the dystrophic FAPs phenotype AScheme of the C1r/s inhibition experiment in the fib‐*MDX* mice.B–F
*Axin2* (B), *LGR5* (C), *TGFβ2* (D), *collagen 1a1* (E), and *collagen 3a1* (F) mRNA expression in FACS‐isolated FAPs from ~1‐year‐old *MDX* mice processed as in (A). *N* (biological samples) = 3.GRepresentative immunofluorescence of *gastrocnemius* muscles from ~1‐year‐old *MDX* mice processed as in (A). Muscles were stained with anti‐collagen 1 (red), anti‐laminin 2α (white) antibodies, and Hoechst (blue). Scale bar: 50 μm.HFibrotic area quantification of the same muscles as in (G). *N* (biological samples) = 3.ICross‐sectional area (CSA) quantification of the muscle fibers of the same muscles as in (G). *N* (biological samples) = 3.JCollagen 1 pixel intensity quantification in the interstitial space between myofibers of the same muscles as in (G). *N* (biological samples) = 3.KModel of the mechanism through which the C1/WNT axis affects FAPs in dystrophic muscles. C1r/s subunits are indicated as pharmacological targets for the effective inhibition of fibrosis. Created with BioRender.com. Data information: In (B–F) and (H–J), data are presented as mean ± SEM. In (H and J), each graph dot represents the average value of 144 to 904 (H) and 17 to 27 (J) measurements on different muscle regions for each biological sample. In (I) each graph dot represents the median value of 201 to 372 cross‐sectional area measurements on different muscle regions for each biological sample. Statistical differences were calculated by paired two‐tailed Student's *t‐*test. *P*‐values are as indicated. Source data are available online for this figure.

Our findings suggest that inhibiting complement C1 might be a way to counteract the aberrant behavior of FAPs in the muscles of dystrophic mice. To directly test this possibility, we systemically administered to the fib‐*MDX* mice a C1r/s inhibitor (Fig 6A). The gene expression analysis of FACS‐isolated FAPs from muscles revealed a reduced expression of both the canonical WNT signaling targets (i.e., *Axin2*, *LGR5*, and *TGFβ2*) and the fibrogenic genes (i.e., c*ollagen 1a1* and *collagen 3a1*) in the fib‐*MDX* mice after the complement inhibition (Fig 6B–F). Moreover, the intensity of the staining for TGFβ2 and the intracellular fibrotic marker HSP47 was reduced in FAPs in the muscles of the fib‐*MDX* mice treated with the complement inhibitor (Appendix Fig S15A and B). The inhibition of the fibrotic program associated with the WNT signaling appears to be FAPs specific, as it is not similarly altered in MuSCs (Appendix Fig S15C–G). Importantly, the muscles of the C1r/s inhibitor‐treated fib‐*MDX* mice presented a reduced space filled by fibrotic tissue (Fig 6G and H), which paralleled an increased mean cross‐sectional area of myofibers (Fig 6I). Moreover, the histological analysis revealed a decreased deposition of collagen in the interstitial space between myofibers in the muscles treated with the C1r/s inhibitor compared to the controls (Fig 6G and J, and Appendix Fig S15H and I). Altogether, these data highlight an amelioration of the dystrophic phenotype following the inhibition of the classical complement pathway (Fig 6K).

**Figure EV5:**
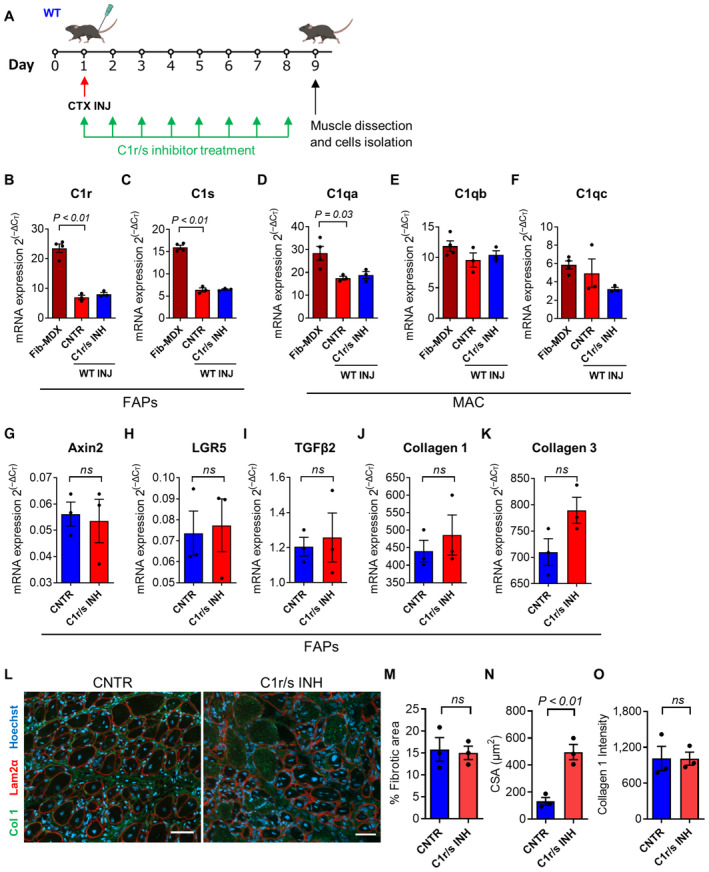
*In vivo* inhibition of C1r/s in *WT* muscles after acute injury: FAPs, macrophages, and tissue analysis AScheme of the C1r/s inhibition experiment in *WT* muscles after acute cardiotoxin injury. Note that the length of the treatment with the C1r/s inhibitor corresponds to the time between the last needle injury and the moment of muscle dissection in the fib‐*MDX* experiments (Fig 6A).B–F
*C1r* (B), *C1s* (C), *C1qa* (D), *C1qb* (E), and *C1qc* (F), mRNA expression in FACS‐isolated FAPs (C1r and C1s), and macrophages (C1qa, C1qb, and C1qc) from fib‐*MDX* and *WT* mice processed as in (A). *N* (biological samples) = 3. Fib‐*MDX* samples are the same as used in the analysis shown in Fig 5E–I.G–K
*Axin2* (G), *LGR5* (H), *TGFβ2* (I), *collagen 1a1* (J), and *collagen 3a1* (K) mRNA expression in FACS‐isolated FAPs from *WT* mice processed as in (A). *N* (biological samples) = 3.LRepresentative immunofluorescence of *gastrocnemius* muscles from *WT* mice processed as in (A). Muscles were stained with anti‐collagen 1 (green), anti‐laminin 2α (red) antibodies, and Hoechst (blue). Scale bar: 50 μm.MFibrotic area quantification of the same muscles as in (L). *N* (biological samples) = 3.NCross‐sectional area (CSA) quantification of the muscle fibers of the same muscles as in (L). *N* (biological samples) = 3.OCollagen 1 pixel intensity quantification in the interstitial space between myofibers of the same muscles as in (L). *N* (biological samples) = 3. Data information: Data are presented as mean ± SEM. In (M and O) each graph dot represents the average value of 62 to 342 (M) and 31 to 45 (O) measurements on different muscle regions for each biological sample. In (N) each graph dot represents the median value of 351 to 415 cross‐sectional area measurements on different muscle regions for each biological sample. The statistical differences were calculated in (B–F) by unpaired two‐tailed Student's *t‐*test and in (G–K) and (M–O) by paired two‐tailed Student's *t‐*test. *P*‐values are as indicated. Source data are available online for this figure.

Despite being both dystrophy and acute injury characterized by a pronounced infiltration of inflammatory cells, the repertoire of macrophages appears different in the two conditions in terms of C1q expression (see Fig EV3F–H and Appendix Fig S10F–H). We decided to gain insight into this diversity by injecting the hindlimb muscles of *WT* mice with cardiotoxin and administering the same C1r/s inhibitor regimen that we have used in the fib*‐MDX* mice (Fig EV5A). Eight days after injury, we collected the muscles from both treated and control animals and FACS‐isolated FAPs and macrophages. As expected from the analysis performed at an earlier time point after injury (see Fig 2A–E), the five C1 subunits tend to be transcribed less in cells isolated from the acutely injured muscles compared to those isolated from the dystrophic fib‐*MDX* (Fig EV5B–F). Intriguingly, this reduction parallels a shift toward an anti‐inflammatory phenotype (i.e., characterized by a predominance of IL‐10 expression) of the macrophages and a reduction in the expression of CD206 and CD163 (Appendix Fig S16A–D). Importantly, in contrast to what we observed in the fib*‐MDX* model, the C1r/s inhibitor is not changing the expression of the WNT‐target genes and collagens in FAPs isolated from acutely injured *WT* muscles (Fig EV5G–K). Moreover, despite the increase in fiber size, the accumulation of fibrotic tissue is not significantly altered by the administration of the C1r/s inhibitor (Fig EV5L–O). These observations disclose intrinsic differences in the process leading to fibrosis in physiological and dystrophic settings and highlight a peculiar detrimental profibrotic role played by the complement C1/WNT axis in dystrophic muscle (Appendix Fig S17).

## Discussion

Our data link the enhanced local production of the five subunits of the C1 complex by macrophages and FAPs in dystrophic muscles with enhanced WNT signaling and activation of a fibrogenic program in FAPs. Here, we present direct evidence that C1, the upstream component of the classical complement cascade, contributes to the etiology of fibrosis in the muscle affected by DMD.

So far, the involvement of the complement system in the etiology of fibrosis in DMD has been unexplored, being the complement system historically investigated in the process of myofiber necrosis (Engel & Biesecker, 1982; Sewry *et al*, 1987; Spuler & Engel, 1998). The presence of the membrane attack complex, complement C3, C8, and C9 was described in the necrotic fibers of DMD patients, but lack of clarity remains whether complement plays a causative role during the processes leading to fiber damage or is recruited after membrane lesions have already appeared (Sewry *et al*, 1987). Notably, whereas complement components commune to all complement pathways were associated with necrotic fibers, the presence of C1q and C4 was reported to be highly variable in the necrotic areas (Engel & Biesecker, 1982). This observation suggests the predominant involvement of the C1‐independent alternative pathway in the process of necrosis in DMD. Further investigation will be required to understand if the classic complement pathways may also play a role in this context.

We propose that FAPs and macrophages, typically relatively distant in uninjured muscles, invade the regenerative areas of dystrophic muscle and locally secrete distinct complement C1 subunits that combine to induce the WNT‐dependent fibrotic program. This model appears to differ from the one described to explain WNT‐dependent defective regeneration in aging muscle, which likely depends on C1q circulating in the bloodstream (Naito *et al*, 2012). The reduced WNT activity that we report here for the serum collected from the dystrophic animals compared to the old *WT*, together with the similar WNT activity and amount of C1q present in the serum of dystrophic and age‐matched *WT* mice, supports this view (see Fig 1G and Appendix Fig S2A). We could observe a moderate, although non‐statistically significant, increase in C1s in the serum of *MDX* mice compared to the age‐matched *WT* counterpart (Appendix Fig S2B). Additional studies are needed to conclusively evaluate if C1r/s might appear in the dystrophic subjects' bloodstream, possibly by spilling over from diseased muscles. Intriguingly, reduced levels of circulating C1r/s inhibitor have been reported in dystrophic patients (Nagao *et al*, 1987). This observation calls for an investigation of the levels of C1r/s inhibitor in aging and further supports our idea of using C1r/s inhibitor as a pharmacological tool in DMD.

Our study does have some limitations. Although our analysis showed a clear reduction in fibrosis upon C1r/s inhibitor administration, the fib‐*MDX* model that we used does not allow for a long‐term (>3 weeks) functional evaluation. Furthermore, evidence indicates that macrophages consist of heterogeneous populations, which can be discriminated on the preferential involvement during specific phases of the regenerative response or their anatomical origin (i.e., muscle resident or recruited from the circulation) (Tidball & Villalta, 2010; Wang *et al*, 2020; Babaeijandaghi *et al*, 2022). Our data indicate a preferential expression of C1q in a specific macrophage subpopulation dominating the dystrophic muscle and characterized by expression of CD206 and CD163 (see Fig EV3). Nevertheless, the origin of this subpopulation, its relationship with other macrophage subpopulations, and its role during physiological and pathological muscle regeneration remain largely undisclosed and call for future in‐depth characterizations. A similar investigation can be put forward for C1r/s expression in FAPs, which also are reportedly heterogeneous (Farup *et al*, 2021).

Another aspect requiring additional investigation concerns the influence of the complement C1/WNT axis on the behavior of the MuSCs. Previous reports indicate that the activation of the WNT signaling pathway promotes TGFβ signaling and that MuSCs acquire a fibrotic phenotype in a TGFβ‐dependent manner in the dystrophic environment (Biressi *et al*, 2014; Pessina *et al*, 2015). Nevertheless, the pharmacological inhibition of C1r/s in the fib‐*MDX* model inhibited the WNT signaling and the fibrogenic program in FAPs but not in MuSCs. This difference could depend on features of the fib‐*MDX* model that is magnifying the C1/WNT fibrogenic axis only in FAPs (Fig 5J–N and Appendix Fig S14A–E), or it may depend on intrinsic properties of the MuSCs that present a relatively low expression of the co‐receptor LRP6 (Appendix Fig S14F), and possibly depend on complement‐independent molecular mechanisms to activate the TGFβ‐mediated fibrogenic program.

Fibrosis contributes to the symptomatology of DMD and is a barrier to effective therapies (particularly for cell and gene therapy approaches) (Biressi *et al*, 2020). By increasing our understanding of the mechanisms leading to fibrosis and attempting to interfere with them pharmacologically, this study strives to identify novel therapeutic avenues to ameliorate the condition of DMD patients. Given its upstream position in inflammation, complement C1‐targeting drugs are in use for various rare diseases, and some hold orphan drug status (Reis *et al*, 2015). Different pharmaceutical companies have clinical programs for antibodies blocking C1 subunits to treat cold agglutinin disease, Guillain–Barré Syndrome, and Huntington's disease (Jäger *et al*, 2019). Inhibitors of C1r/s obtained as recombinant proteins or from human plasma, as used in our *in vivo* studies, are available in the clinic with a primary indication for the treatment of hereditary angioedema (Feussner *et al*, 2014). This study explored the possibility of using these drugs to improve the condition of DMD patients.

An increased number of macrophages, high WNT signaling activity, and fibrosis are reportedly associated with cardiac dysfunction (Epelman *et al*, 2015; Tao *et al*, 2016). This aspect is relevant for DMD patients, as cardiac complications may be lethal in dystrophic patients (Finsterer & Stöllberger, 2003). Moreover, a chronic condition of inflammation, complement deposition, and enhanced WNT signaling characterize many unrelated pathological conditions, including hypertensive arterial remodeling associated with atherosclerosis (Sumida *et al*, 2015). Future studies will ascertain if the relevance of our findings extends beyond DMD muscle.

## Materials and Methods

### Mice

*C57BL/6J* mice (No. 000664, used as *wild‐type* animals and herein referred to as *WT*), *B6Ros.Cg‐D* ^*mdmdx‐4Cv/J*^ mice (No. 002378, herein referred to as *MDX*), and *B6.129S4‐PDGFRα* ^*tm11(EGFP)Sor/J*^ reporter mice (No. 007669, herein referred to as PDGFRα^eGFP^) were purchased by The Jackson Laboratories. *PDGFRα* ^*eGFP/WT*^;*MDX* mice were obtained after breeding *PDGFRα* ^*eGFP/WT*^ males with *MDX* homozygous females (Mueller *et al*, 2016).

Muscle needle injuries were performed using 29‐gauge needles. Punctures were performed deep into the muscle (~8 mm), randomly but close enough to each other to cover the whole muscle. Muscles were dissected and analyzed 2.5 days after the injury. The fib‐*MDX* mouse model generation protocol was adapted from a previously published protocol (Desguerre *et al*, 2012). Briefly, 150‐μm‐diameter micropins were used to perform 15 punctures in the *tibialis anterior* and 90 punctures in the *gastrocnemius* muscles daily for 14 days. Muscles were dissected and analyzed 1 week after the last injury. For complement inhibition, fib‐*MDX* mice were treated with 15 UI of human plasma‐derived C1r/s esterase inhibitor (Berinert, provided by CSL Behring) daily ~30 min after performing the microinjuries. C1r/s inhibitor was administered through intraperitoneal or intravenous injections on alternate days for 21 days starting from the first day of microinjuries. For specific experiments, muscle injury was performed by intramuscular injection of cardiotoxin from *Naja pallida* snake venom (Latoxan) resuspended at the concentration of 0.1 mg/ml in PBS 4 or 8 days before hindlimb muscle collection. For complement inhibition during physiological muscle regeneration, *WT* mice were treated with 15 UI of human plasma‐derived C1r/s esterase inhibitor (Berinert) after the cardiotoxin injection and for the following 7 days. The following day, the hindlimb muscles were collected and processed for analysis.

Animal care and experimental procedures were conducted in accordance with the Ethical Committee of the University of Trento and were approved by the Italian Ministry of Health (Authorization Nos. 915/2015‐PR and 94/2022‐PR).

### Protein analysis

Snap‐frozen dissected muscles were homogenized and lysed with RIPA buffer (Thermo Scientific), protease inhibitor (1:100, Thermo Scientific), and phosphatase (1:100, New England BioLabs). The protein concentration was quantified using Pierce BCA Protein Assay Kit (Thermo Scientific). Standard indirect ELISA assays were performed on mice muscles and on mice sera. Anti‐LGR5 (Abcam 219107, 1:500), anti‐C4 (Santa Cruz 58930, 1:100), anti‐C1s (LS‐C483829 1:50), and anti‐C1q (Abcam 182451 1:50) antibodies were used. Alkaline phosphatase‐conjugated secondary antibodies (Life Technology) and p‐nitrophenyl phosphate (Sigma) solution were used for the colorimetric assay at 405 nm (Tecan infinite M200 reader and Ensight reader). Background obtained with the secondary antibody was subtracted. Human muscle lysates were resolved by SDS–PAGE and transferred to PVDF membrane (Amersham™ Hybond™, Fisher Scientific). The membranes were blocked with 5% non‐fat dry milk in TBS‐T (50 mM Tris–HCl, pH 7.5, 150 mM NaCl, and 0.1% Tween20) and then incubated with anti‐C1s (LS‐C483829, LS Bio 1:500) or anti‐GAPDH (Thermo Fisher Sci., MA515738, 1:5,000) primary antibodies overnight at 4°C. Membranes were incubated with an HRP‐conjugated anti‐rabbit (Cell Signaling, 7074) or HRP‐linked anti‐mouse (Cell Signaling, 7076) secondary antibody. Immunoreactive bands were detected using ECL LiteAblot plus kit A + B (Euroclone, GEHRPN2235) with an Alliance LD2 device and software (UVITEC). C1s and GAPDH signal intensities were analyzed with ImageJ.

### Cell isolation and culture

Single myofibers were isolated from the *extensor digitorum longus* mouse muscles as previously described (de Morrée *et al*, 2017). To obtain mononucleated cells, murine hindlimb, diaphragm muscles, or human biopsies were processed as previously described (Liu *et al*, 2015). Fluorescence‐activated cell sorting (FACS) Aria III cell sorter (BD Biosciences) was used to separate cell populations. Mouse‐derived MuSCs were purified by negative selection with anti‐CD31, anti‐CD45, and anti‐Sca1 antibodies and positive selection with antivascular cell adhesion molecule (VCAM) antibody (Liu *et al*, 2015), mouse‐derived FAPs were purified by negative selection with anti‐CD31 and anti‐CD45 antibodies and positive selection with an anti‐Sca1 antibody (Judson *et al*, 2017), while human‐derived FAPs were purified by negative selection with anti‐CD31 and anti‐CD45 antibodies and positive selection with an anti‐CD34 antibody (Farup *et al*, 2021), and mouse‐derived macrophages were purified by positive selection with anti‐CD45 and anti‐F4/80 antibodies. For specific experiments, mouse‐derived macrophages were fractionated with an anti‐CD206 antibody. CD45^−ve^CD31^−ve^ cells are indicated as Lin^−ve^ cells, whereas CD45^+ve^CD31^+ve^ cells are indicated as Lin^+ve^ cells. A list of primary antibodies used is enclosed in Appendix Table S4. APC Streptavidin (1:100, BioLegend) was added to samples incubated with a biotin‐conjugated anti‐VCAM antibody. After sorting, cells were cultured and processed for immunostaining or RNA extraction. Mouse FAPs were cultured in DMEM supplemented with 10% fetal bovine serum (FBS) de‐complemented by heat inactivation and 2.5 ng/ml basic fibroblast growth factor (bFGF, LSBio). For immunostaining, cells were resuspended in Ham's F‐10 supplemented with 20% FBS and allowed to adhere overnight on glass ECM‐coated slides (Merck).

C2C12, RAW‐264.7, HEK‐293, C3H‐10T1/2, NIH‐3T3, and STO were obtained from ATCC and maintained in DMEM supplemented with 10% FBS de‐complemented by heat inactivation. C57 primary myoblasts were isolated from *C57BL/6J* mice as previously described (Rando & Blau, 1994; Biressi *et al*, 2014). Primary cultures were plated on 5 μg/ml laminin/collagen‐coated dishes and amplified in Ham's F‐10 with 20% FBS, and 2.5 ng/ml bFGF was added to the culture every 24 h. All cell lines were mycoplasma free. In the experiments with mice sera, C2C12 cells were cultured in DMEM supplemented with 5% mice serum collected from *WT* or *MDX* mice. In experiments with conditioned media, STO, and RAW‐264.7, cells were allowed to reach confluence for 4.5 days. Media were collected and filtered through a 0.42 μm filter. STO and RAW‐264.7 media were combined in an 8:1 ratio, which was previously calculated from *in vivo* analysis of the proportion of macrophages and FAPs in *MDX* muscles (Appendix Table S5). Reagents used are as follows: XAV‐939 (Merck, 100 μM), WNT3A (StemRD, 50–100 ng/ml), C1q (Sigma, 100 μg/ml), C1r (BIOPUR, 25 μg/ml), C1s (BIOPUR, 25 μg/ml), and C1r/s inhibitor (Merck, 100 μg/ml).

### Quantitative RNA analysis

RNA was extracted using TRIzol Reagent (Invitrogen), and RNA was reverse transcribed using High‐Capacity cDNA Reverse Transcription Kit (Thermo Fisher Scientific) according to the manufacturer's instructions. Gene expression was measured by quantitative RT–PCR using SYBR Green Master Mix (Thermo Fisher Scientific). The efficiency of all primers was calculated as ≥ 96%. Primers are indicated in Appendix Table S6. Relative quantification was normalized to mouse or human hypoxanthine–guanine phosphoribosyltransferase (HPRT). UMAP plots referred to the expression of C1 components, CD206, and IL‐10 in human muscles were produced with Plotly.js (v2.11.0) and obtained from Tabula Sapiens single‐cell transcriptomic analysis (Jones *et al*, 2022). The dataset referred to C1 and CD163 expression in mouse limbs is available in the following database: Tabula Muris Dataset: https://tabula‐muris.ds.czbiohub.org/ (Tabula Muris Consortium *et al*, 2018).

### Human biopsies

The Biobank of skeletal muscle, peripheral nerve, DNA, and cell lines—IRCCS Ca′ Granda Ospedale Maggiore Policlinico, Milan (request No 1504)—provided the human biopsies used for C1 expression. Muscle biopsies for FACS and immunofluorescence analysis were collected from orthopedic surgery. Procedures were performed under the International Conference on Harmonisation of Good Clinical Practice guidelines, the Declaration of Helsinki (2008), the Department of Health and Human Services Belmont Report, and the European Directive 2001/20/EC. Informed consent was obtained after the nature and possible consequences of the study were explained. A list of human biopsies used in this study is enclosed in Appendix Table S1.

### Immunofluorescence, cross‐sectional area analysis, interstitial fibrotic area calculation, and hematoxylin–eosin staining

Cells and muscle sections were processed for immunofluorescence as previously described (Biressi *et al*, 2013). Briefly, dissected muscles were fixed for 4 h using 0.5% paraformaldehyde, then transferred to 30% sucrose overnight, frozen in optimum cutting temperature compound (OCT), and cryosectioned at 8 μm. For morphometric and fibrotic area quantification, HSP47 and TGFβ2 quantification muscles were directly frozen in nitrogen‐cooled isopentane and cryosectioned at 6 μm. The acquisition was made with a Zeiss Axio Observer Z1 optical microscope equipped with a monochrome camera (AxioCam 503 mono D), with Nikon AX confocal laser scanning microscope or Leica TCS SP8 confocal microscopy. Primary antibodies are listed in Appendix Table S7. Alexa Fluor 488/594/647 secondary antibodies (Thermo Fisher Scientific) were used.

Zen 2 software (Zeiss) and ImageJ were used for immunofluorescence analysis. Normal or non‐normal dataset distribution, Spearman (*r*), and Pearson coefficient (*r*) were determined with GraphPad Prism software.

Details of the performed quantifications are the following: (i) C1q pixel intensity quantification in *WT* versus *MDX gastrocnemius* (Fig 1K): For each biological replicate, 9–44 different regions of the muscle were evaluated for C1q staining, and 5–18 different regions were evaluated for background signal (staining with only secondary antibody). The average background value was subtracted from the average C1q pixel intensity value for each biological replicate. Pixel intensity was calculated using ZEN 2 software (Zeiss). (ii) Axin2 pixel intensity quantification in human sections (Appendix Fig S1B): For each biological replicate, 8–59 different regions within the regenerating/interstitial muscle portions were evaluated for Axin2 staining, and 9–34 different regions were evaluated for background (staining with only secondary antibody). The background staining was used to evaluate the specificity of Axin2 staining. Pixel intensity was calculated using ZEN 2 software (Zeiss). (iii) TGFβ2 pixel intensity quantification in human sections (Appendix Fig S1C): For each biological replicate, 11–22 different regions (images) of the regenerating/interstitial muscle areas were evaluated for TGFβ2 staining, and 8 to 15 different regions (images) were evaluated for background (staining with only secondary antibody). The average background intensity value was subtracted from the corresponding TGFβ2 signal for each biological sample. Pixel intensity values were calculated with ImageJ. (iv) C1s pixel intensity quantification in *WT* versus *MDX gastrocnemius* (Appendix Fig S3A): For each biological replicate, 8–28 different regions of the muscle were evaluated for C1s staining, and 4–18 different regions were evaluated for background signal (staining with only secondary antibody). For each biological replicate, the average background value was subtracted from the average C1s pixel intensity value. Pixel intensity was calculated using ZEN 2 software (Zeiss). (v) Axin2 and TGFβ2 pixel intensity quantification in *WT* and *MDX* PDGFRα^+ve^ cells (Fig EV1A and B): Axin2 pixel intensity was measured in 131 (for *WT*) and 130 (for *MDX*) PDGFRα^+ve^ cells. TGFβ2 pixel intensity was measured in 126 (for *WT*) and 215 (for *MDX*) PDGFRα^+ve^ cells. The average background intensity was calculated by averaging the intensity of cells stained only with secondary antibodies (46 cells for *WT* and 48 cells for *MDX*) and was subtracted from each measurement. For each biological sample, 7–25 different regions were analyzed for Axin2, 10 to 28 different regions were analyzed for TGFβ2, and 3–4 different regions were analyzed for background intensities. Pixel intensity was calculated using ZEN 2 software (Zeiss). (vi) C1q and C1s pixel intensity quantification in FACS‐isolated cells from human muscles (Appendix Fig S6G and H): The maximum pixel intensity was measured in cells stained with anti‐C1q and anti‐C1s antibodies. C1s was evaluated in 478 FAPs, 171 CD45^+ve^ cells, and 206 CD31^+ve^ pooled from four (FAPs and CD31^+ve^ cells) and three (CD45^+ve^ cells) independent experiments. C1q signal was evaluated in 109 FAPs, and 187 CD45^+ve^ pooled from three (FAPs) and two (CD45^+ve^ cells) independent experiments. The background signal was evaluated in each biological sample using the staining with only secondary antibodies. Specific signal intensities and the extreme top and bottom 5% of the population were excluded. Pixel intensity was calculated using ZEN 2 software (Zeiss). (vii) Macrophages and FAPs distance analysis (Fig 4A and C): The distance between macrophages and FAPs was analyzed by measuring the distance of each macrophage from its closest FAP (nucleus to nucleus); for each biological replicate, 60–200 macrophages were selected in at least 20 different muscle areas; Appendix Fig S11C and D: The distance between FAPs and macrophages was analyzed by measuring the distance of each FAP from its closest macrophage (nucleus to nucleus); for each biological replicate, 100–350 FAPs were selected in at least four different muscles' areas; Fig EV4B and D: The distance between macrophages and FAPs was analyzed by measuring the distance of each macrophage from its closest FAP (nucleus to nucleus); for each biological replicate, 261–307 macrophages were selected in 17–44 (*WT* INJ), 11–34 (*MDX*), and 8–13 (fib‐*MDX*) different muscles' areas. (viii) C1q/Axin2 and C1q/TGFβ2 correlation analysis in *MDX* muscles (Fig 4D and E): The average pixel intensity of C1q, Axin2, and TGFβ2 was measured for each biological replicate in 45–94 randomly selected regions of 1,029 μm^2^ within the regenerating areas of the muscle. The background pixel intensity measured on sections stained only with the secondary antibody was subtracted. Images were acquired with a Leica TCS SP8 confocal microscope. Pixel intensity was calculated using ImageJ. (ix) TGFβ2^+ve^ FAPs quantification in *MDX* and fib‐*MDX* muscles (Fig EV4I): The percentage of TGFB2^+ve^ cells was calculated on a total number of 1,162 (*MDX*) and 1,319 (fib‐*MDX*) PDGFRα^+ve^ cells. For each biological sample, 3–10 different randomly selected muscle regions were analyzed. (x) Myofiber cross‐sectional area (CSA) analysis: The myofiber cross‐sectional area (CSA) was measured in 201–372 (Fig 6I) and 351–415 (Fig EV5N) randomly selected fibers for each biological replicate, and the median values of these measurements were calculated. (xi) Collagen 1 and collagen 3 pixel intensity in muscles (Fig 6J and Appendix Fig S15I): The average collagens 1 and 3 pixel intensity was calculated for each biological replicate in 17 to 27 randomly selected interstitial regions within the muscle sections. For each biological sample, the average background value was calculated from at least nine different randomly selected regions of the muscle stained with only the secondary antibody. The corresponding average background value was subtracted from each collagen 1 and collagen 3 signal intensity measurement. Pixel intensity was calculated using ZEN 2 software (Zeiss); (Fig EV5O): The average collagen 1 pixel intensity was calculated for each biological replicate in 31–45 randomly selected interstitial regions within the muscle sections. For each biological replicate, the average background staining was evaluated in 10–16 randomly selected muscle areas stained with only the secondary antibody. The corresponding average background value was subtracted from each collagen 1 signal intensity measurement. Pixel intensity was calculated using ImageJ. (xii) TGFβ2 pixel intensity quantification in CNTR and C1r/s INH muscles: TGFβ2 average pixel intensity was measured in 155 (CNTR) and 320 (C1r/s INH) PDGFRα^+ve^ cells. For each biological sample, TGFβ2^+ve^ cells were analyzed in 7–14 different muscle regions. For each biological sample, the average background value was calculated from at least 30 cells stained with only secondary antibodies, and randomly selected in at least three different muscle regions. The corresponding average background value was subtracted from each TGFβ2 signal intensity measurement. Pixel intensity was calculated using ZEN 2 software (Zeiss). (xiii) HSP47 pixel intensity quantification in CNTR and C1r/s INH PDGFRα^+ve^ cells (Appendix Fig S15B): HSP47 average pixel intensity was measured in 209 (CNTR) and 234 (C1r/s INH) PDGFRα^+ve^ cells For each biological sample, PDGFRα^+ve^ cells were analyzed in 4–12 different muscle regions. For each biological sample, the average background value was calculated from at least 30 cells stained with only secondary antibodies and randomly selected in at least three different muscle regions. The corresponding average background value was subtracted from each HSP47 signal intensity measurement. Pixel intensity was calculated using ZEN 2 software (Zeiss).

The interstitial fibrotic area was calculated as the percentage of the area with collagen 1 deposition over the total muscle area: Fig 6H: For each biological replicate, 510–904 (CNTR) and 144–497 (C1r/s INH) areas were evaluated for collagen deposition in 11–13 (CNTR) and 10–20 (C1r/s INH) different randomly selected muscles regions and the average fibrotic area value was calculated; Fig EV5M: For each biological replicate, 187–342 (CNTR) and 62–312 (C1r/s INH) areas were evaluated for collagen deposition in 4–5 (CNTR) and 4–7 (C1r/s INH) different randomly selected muscle regions, and the average fibrotic area value was calculated.

For hematoxylin/eosin (H&E) staining, muscles were flash‐frozen in isopentane, and mid‐belly cryostat sections (8 μm) were processed as previously described (George *et al*, 2013).

### Study design and statistical analysis

A primary objective of this study was to evaluate the levels and the role of complement C1 in the skeletal muscles during the dystrophic progression. For this purpose, we compared *MDX* mice with age‐matched *WT* controls and DMD human biopsies with healthy controls, and we analyzed fib‐*MDX* mice after inhibiting the complement pathway. We aimed to investigate the C1/WNT axis in murine muscle cells and dystrophic tissues and to evaluate the C1/WNT axis as a regulator of the FAPs' behavior. Preliminary data and similar studies previously performed with cells and tissues collected from aging and dystrophic mice indicated that 3–10 biological replicates were required in each group to detect a 20% difference in the amounts of gene expression and morphometric measures with a power of 0.8 and significance level of 0.05. Experiments were not blinded. Animals were randomly assigned to experimental and treatment groups based on availability and genotype.

Unless otherwise stated, data are presented as mean ± SEM, one‐way ANOVA test was performed for multiple comparisons, and parametric or non‐parametric Student's *t*‐tests were performed for comparison between two groups. Statistical analysis was performed using GraphPad Prism. The number of biological replicates and tests used to calculate statistical differences are reported in each figure legend. Significant *P*‐values are reported in graphs or tables.

## Author contributions

**Stefano Biressi:** Conceptualization; data curation; formal analysis; supervision; funding acquisition; investigation; visualization; methodology; writing – original draft; project administration; writing – review and editing. **Francesca Florio:** Conceptualization; data curation; formal analysis; investigation; visualization; methodology; writing – original draft; project administration; writing – review and editing. **Sara Vencato:** Investigation; methodology. **Filomena T Papa:** Formal analysis; methodology. **Michela Libergoli:** Methodology. **Eyemen Kheir:** Methodology. **Imen Ghzaiel:** Methodology. **Thomas A Rando:** Conceptualization. **Yvan Torrente:** Resources; funding acquisition; writing – original draft; writing – review and editing.

## Disclosure and competing interests statement

FF and SB compare as inventors in U.S. Provisional Patent Application No. 63/270352 and subsequent PCT Application No. PCT/US22/78510 filed by relevant institutions based on part of the results described in this manuscript. All other authors declare that they have no conflict of interest.

## For more information

For more information about the topics presented in this paper please visit the following websites: (i) https://www.cibio.unitn.it/220/dulbecco‐telethon‐laboratory‐of‐stem‐cells‐and‐regenerative‐medicine (Laboratory of Stem Cell and Regenerative Medicine website). (ii) DMD OMIM: https://www.omim.org/entry/310200.

## Supporting information



Appendix

Expanded View Figures PDF

Source Data for Expanded View

PDF+

Source Data for Figure 1

Source Data for Figure 2

Source Data for Figure 3

Source Data for Figure 4

Source Data for Figure 5

Source Data for Figure 6

## Data Availability

This study includes no data deposited in external repositories.
