# Supplementary material for: Combinatorial activation of the WNT‐dependent fibrogenic program by distinct complement subunits in dystrophic muscle
Source: EMBO Mol Med. 2023 Nov 6;15(12):e17405. doi: 10.15252/emmm.202317405 (PMC10701616; doi:10.15252/emmm.202317405)
Supplement: Supplementary file 1 — Appendix [file EMMM-15-e17405-s011.pdf]

# Appendix for

# Combinatorial activation of the WNT-dependent fibrogenic program by distinct complement subunits in dystrophic muscle

Francesca Florio *et al.*

*Corresponding author: stefano.biressi@unitn.it

## Table of Content

**APPENDIX FIGURES** .......... **2**

**APPENDIX TABLES** .......... **27**

## APPENDIX FIGURES

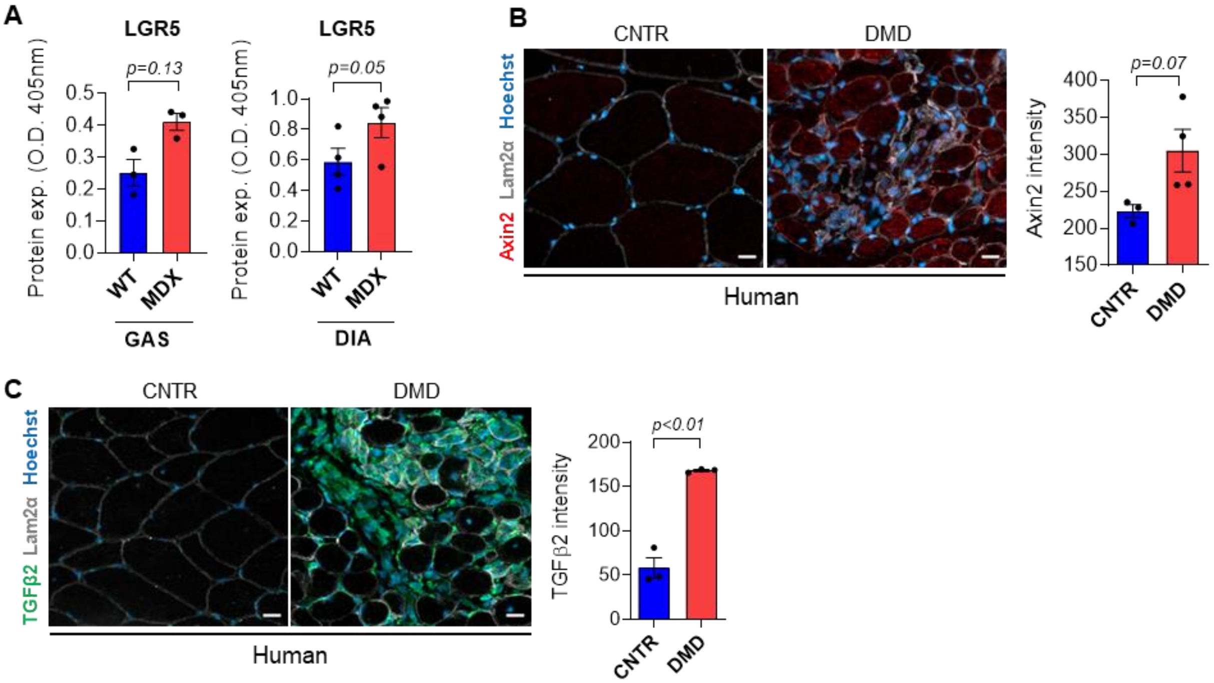


**Appendix Figure S1. Canonical WNT-signaling is increased in dystrophic muscles.**

**A.** ELISA assay of the canonical WTN target protein LGR5 in the *gastrocnemius* (left) and diaphragm (right) of ~1 year-old *WT* and *MDX* mice. N (biological replicates) = 3 for all samples except for LGR5 in diaphragm (N = 4).

**B, C.** Representative immunofluorescence (left) and quantification (right) of human healthy (CNTR) and dystrophic (DMD) muscles stained with anti-Axin2 (red in B), anti-TGFβ2 (green in C), anti-Laminin2α (gray) antibodies and Hoechst (blue). Scale bar: 20 μm. Refer to Table 1 for muscles' details. N (biological replicates) = 3 for all samples (except for Axin2 in DMD, N = 4).

Data information: Data are presented as mean ± SEM. In B and C, each graph dot represents the

average value of 8 to 59 (B) and 11 to 22 (C) measurements on different muscle regions for each biological sample. Statistical differences were calculated by paired (in A) or unpaired (in B and C) two-tailed Student's t-test. P values are as indicated.

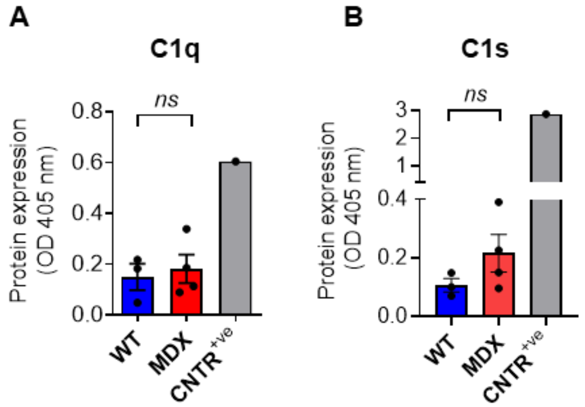


**Appendix Figure S2. Complement evaluation in dystrophic serum.**

**A, B.** ELISA assay of C1q (A) and C1s (B) proteins in the serum of ~1 year-old *WT* and *MDX* mice. N (biological replicates) = 3 (*WT*), 4 (*MDX*), 1 ($CNTR^{+ve}$). $CNTR^{+ve}$: C1q 10 µg/ml (A), C1s 5 µg/ml (B).

Data information: Data are presented as mean ± SEM. Statistical differences were calculated by unpaired two-tailed Student's t-test.

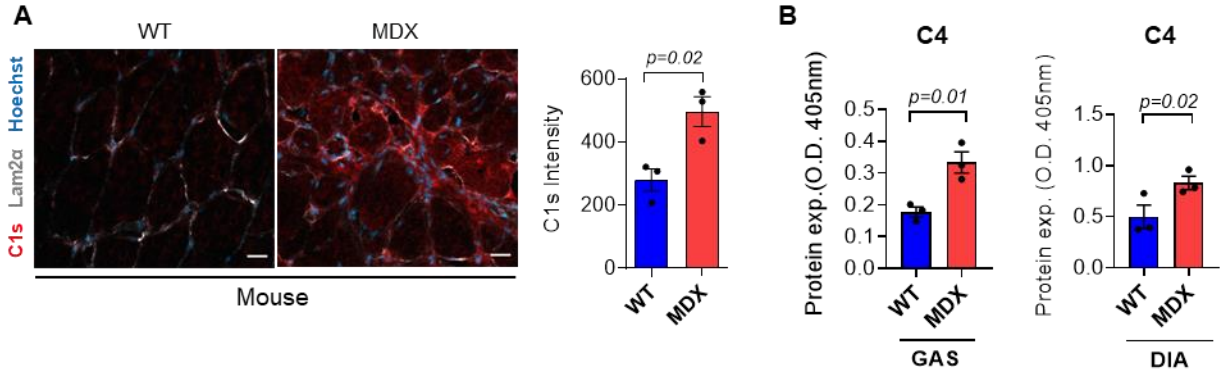


**Appendix Figure S3. Canonical WNT-signaling is increased in dystrophic muscles.**

**A.** Representative immunofluorescence (left) and quantification (right) of C1s in the *gastrocnemius* of ~1 year-old *WT* and *MDX* stained with anti-C1s (red), anti-Laminin2α (gray) antibodies and Hoechst (blue). Scale bar: 20 µm. N (biological replicates) = 3.

**B.** ELISA assay of C4 in the *gastrocnemius* (left) and diaphragm (right) of ~1 year-old *WT* and *MDX* mice. N (biological replicates) = 3 for all samples.

Data information: Data are presented as mean ± SEM. In A, each dot on the graphs represents the average value of 8 to 28 measurements on different muscle regions for each biological sample. Statistical differences were calculated by unpaired (in A) or paired (in B) two-tailed Student’s t-test. P values are as indicated.

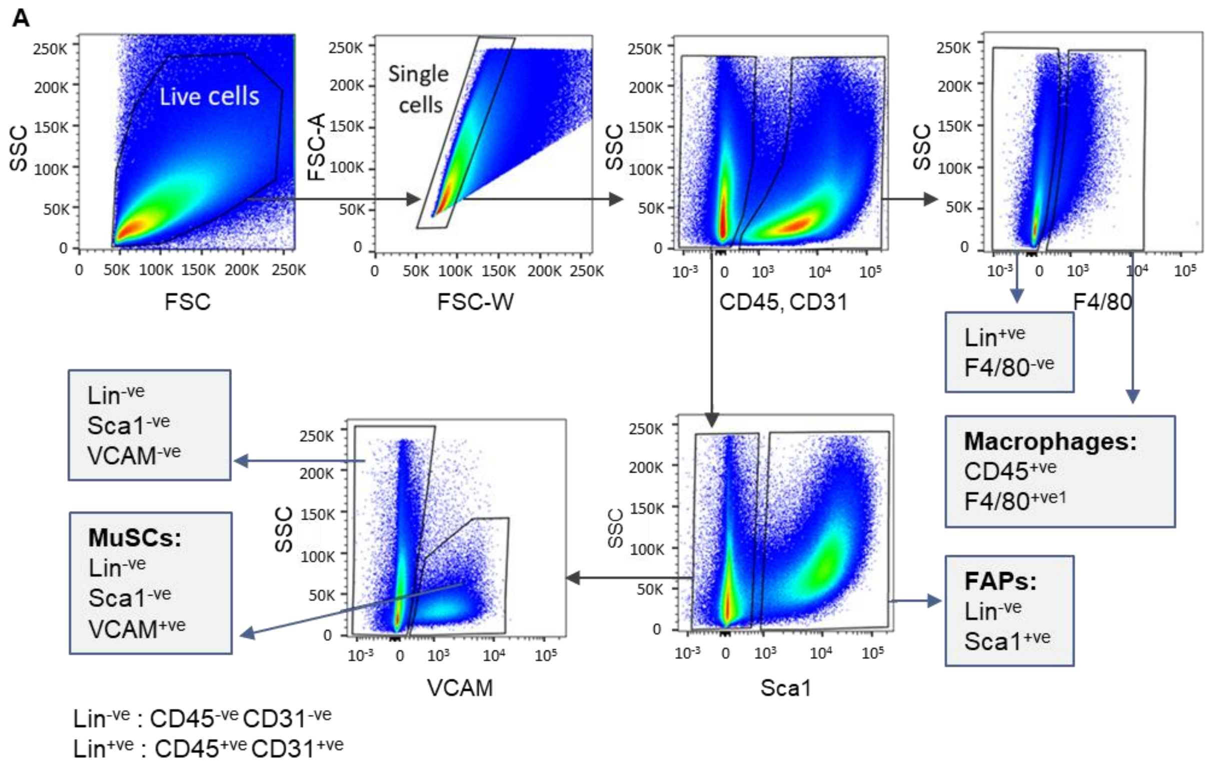


**Appendix Figure S4. FACS-purification of cells from skeletal muscle**

**A.** Representative gating and sorting strategy used to FACS-isolate macrophages (MAC: CD45$^{+ve}$F4/80$^{+ve}$), FAPs (Lin$^{-ve}$Sca1$^{+ve}$), MuSCs (Lin$^{-ve}$Sca1$^{-ve}$VCAM$^{+ve}$), Lin$^{+ve}$F4/80$^{-ve}$ and Lin$^{-ve}$Sca1$^{-ve}$VCAM$^{-ve}$ cell populations from the distal hindlimb of *WT* and *MDX* mice.

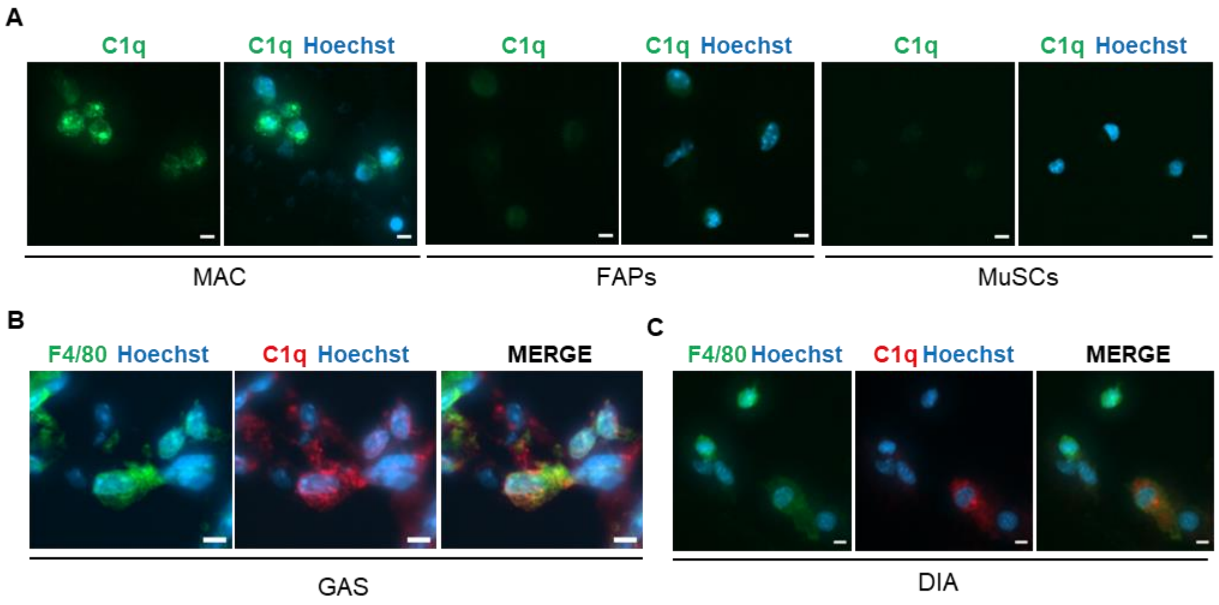


**Appendix Figure S5. Distinct cell types express C1 complex's components in murine muscles.**

**A.** Representative immunofluorescence image of FACS-isolated MuSCs, FAPs, and macrophages (MAC) from ~1 year-old *MDX* hindlimb muscles stained with an anti-C1q antibody (green) and Hoechst (blue). C1q is selectively expressed by macrophages. Scale bar: 5 µm.

**B, C.** Representative immunofluorescence of a cryosection of ~1 year-old *MDX gastrocnemius* (B) and ~5 months-old *MDX* diaphragm (C) stained with anti-F4/80 (green) and anti-C1q (red) antibodies, and Hoechst (blue). Scale bar: 5 µm.

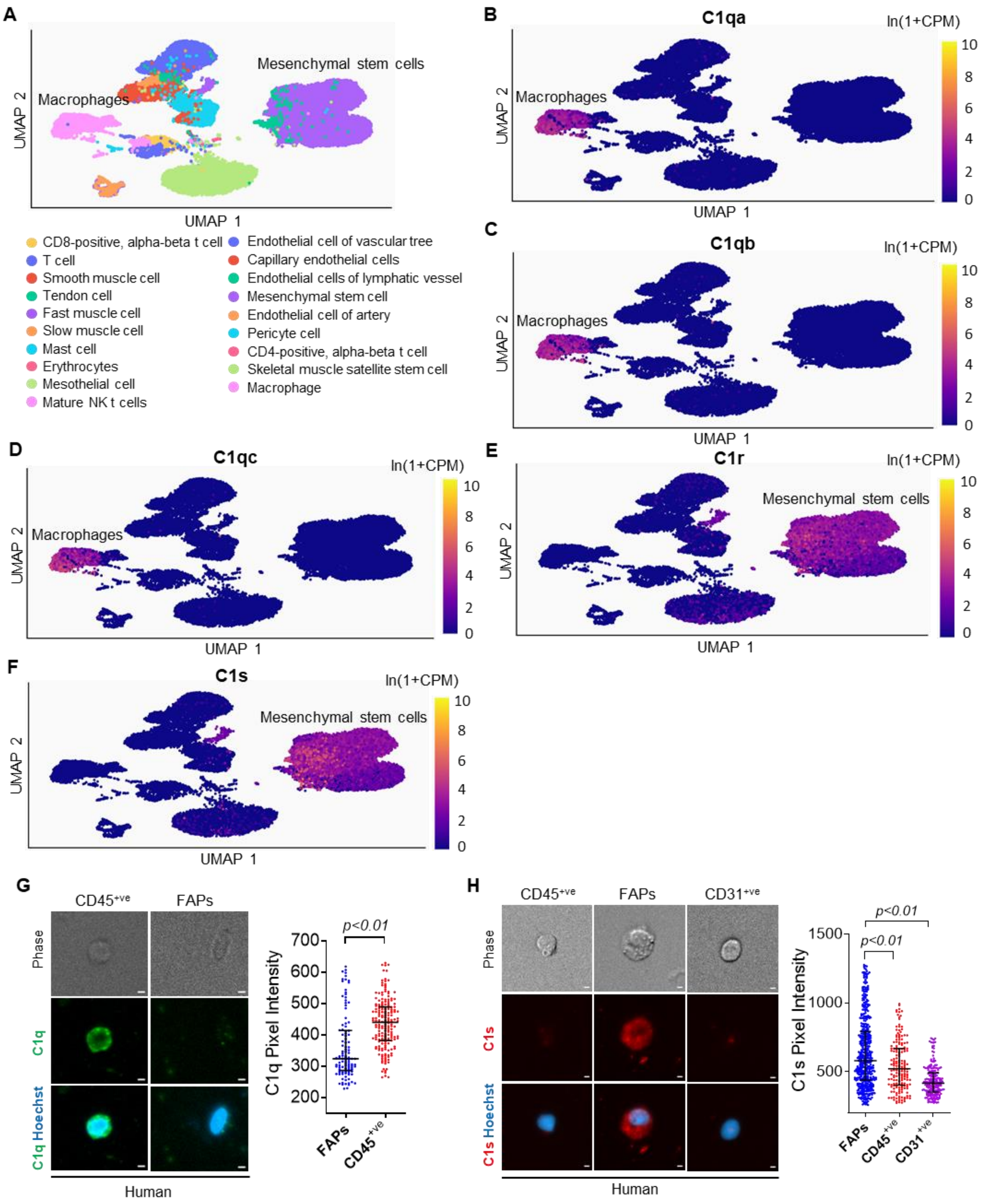
A
Mesenchymal stem cells
Macrophages
UMAP 2
UMAP 1
CD8-positive, alpha-beta t cell
T cell
Smooth muscle cell
Tendon cell
Fast muscle cell
Slow muscle cell
Mast cell
Erythrocytes
Mesothelial cell
Mature NK t cells
Endothelial cell of vascular tree
Capillary endothelial cells
Endothelial cells of lymphatic vessel
Mesenchymal stem cell
Endothelial cell of artery
Pericyte cell
CD4-positive, alpha-beta t cell
Skeletal muscle satellite stem cell
Macrophage
B
C1qa
ln(1+CPM)
Macrophages
C
C1qb
ln(1+CPM)
Macrophages
D
C1qc
ln(1+CPM)
Macrophages
E
C1r
ln(1+CPM)
Mesenchymal stem cells
F
C1s
ln(1+CPM)
Mesenchymal stem cells
G
CD45+ve
FAPs
Phase
C1q
C1q Hoechst
Human
C1q Pixel Intensity
p<0.01
FAPs
CD45+ve
H
CD45+ve
FAPs
CD31+ve
Phase
C1s
C1s Hoechst
Human
C1s Pixel Intensity
p<0.01
p<0.01
FAPs
CD45+ve
CD31+ve

**Appendix Figure S6. Distinct cell types express C1 complex's components in human muscles.**

**A.** UMAP plot showing the human cell types in which the expression of C1 components was evaluated by interrogating the Tabula Sapiens single-cell transcriptomics analysis (Jones et al, 2022 [DATASET]).

**B-F.** UMAP plots showing the expression of C1qa (B), C1qb (C), C1qc (D), C1r (E), and C1s (F) in human cell types as indicated in (A). C1qa, C1qb, and C1qc are expressed mainly by macrophages, whereas C1r and C1s are primarily expressed by mesenchymal stem cells.

**G, H.** Representative immunofluorescence images (left) and quantification (right) of C1q (G) and C1s (H) pixel intensity measured in indicated cells FACS-isolated from human skeletal muscles and stained with anti-C1q (green), anti-C1s (red) antibodies and Hoechst (blue). C1q is more expressed in $CD45^{+ve}$ cells, and C1s is more expressed in FAPs. Scale bar: 2 µm. N (biological samples) = 4 (C1s in FAPs and $CD31^{+ve}$ cells), 3 (C1s in $CD45^{+ve}$ cells and C1q in FAPs), 2 (C1q in $CD45^{+ve}$ cells).

Data information: In G and H, data are presented as median with interquartile range. In G and H, dots on the plots represent single cells' measurements (109 for FAPs and 187 for $CD45^{+ve}$ in C1q analysis; 478 for FAPs, 171 for $CD45^{+ve}$ cells, and 206 for $CD31^{+ve}$ cells in C1s analysis). In G, the statistical difference was calculated by the Mann-Whitney test. In H, the statistical differences were calculated by the Kruskal-Wallis test, and Dunn's multiple comparison test was used as a *post hoc* test. P values are as indicated.

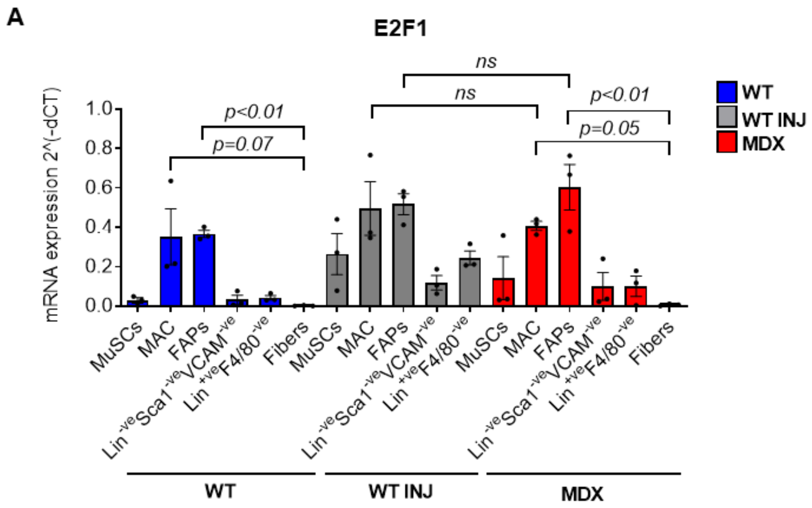


**Appendix Figure S7. Characterization of the muscle-isolated cells.**

**A.** *E2F1* mRNA expression in MuSCs, macrophages (MAC), FAPs, Lin$^{+ve}$F4/80$^{-ve}$, Lin$^{-ve}$Sca1$^{-ve}$VCAM$^{-ve}$, and single fibers isolated from hindlimb muscles as in Figure 2A-E. N (biological samples) = 3. Note the low expression of E2F1 in non-proliferative myofibers.

Data information: Data are presented as mean ± SEM. Statistical differences between two groups were calculated by unpaired two-tailed Student's t-test, and the corresponding p values are reported on the graph. Statistical differences between three or more groups were calculated by one-way ANOVA test, Tukey's multiple comparison test was used as a *post hoc* test, and all the corresponding p values are enclosed in Table 3.

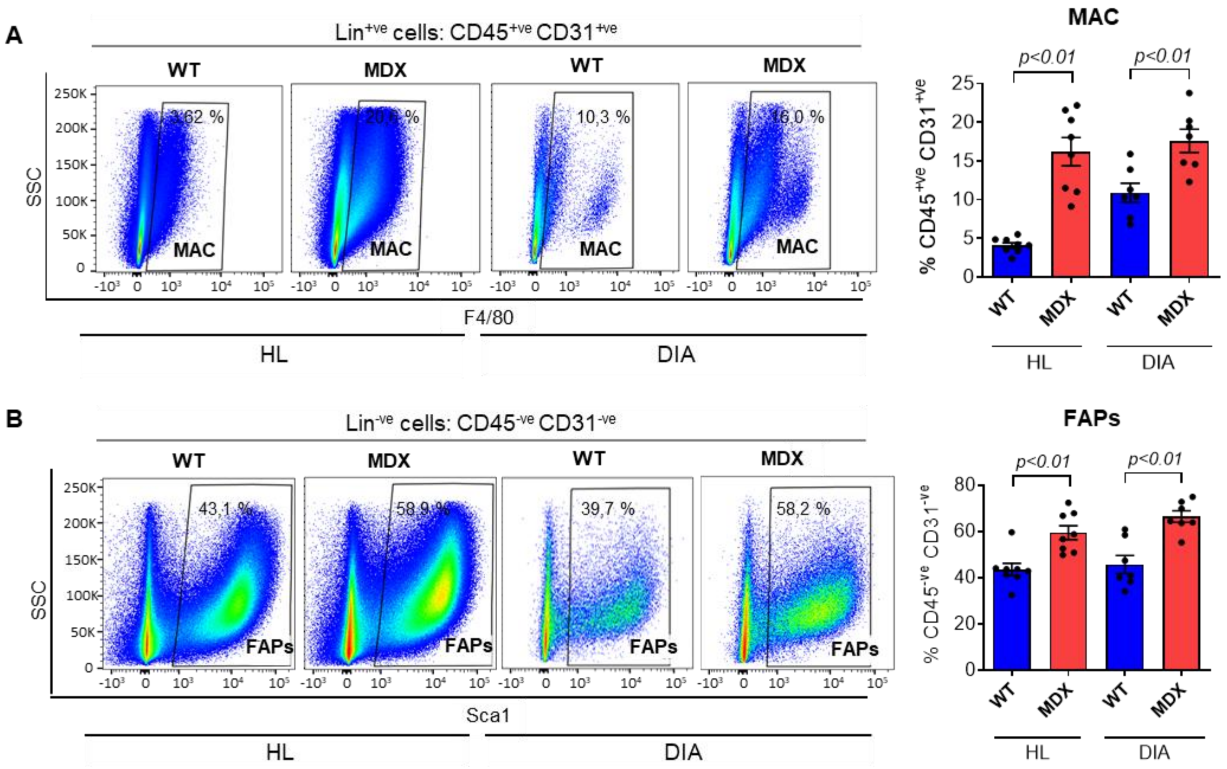


**Appendix Figure S8. Macrophages and FAPs are increased in dystrophic muscles.**

**A, B.** Representative FACS plots (left) and quantification (right) of macrophages (A) and FAPs (B) expressed as the percentage of $Lin^{+ve}$ cells (A) and $Lin^{-ve}$ cells (B) in the hindlimb (HL) and diaphragm (DIA) muscles of ~1 year-old *WT* and *MDX* mice. N (biological samples) = 8 (HL), 7 (DIA).

Data information: Data are presented as mean ± SEM. In A-B, statistical differences between two groups were calculated by unpaired two-tailed Student's t-test. P values are as indicated.

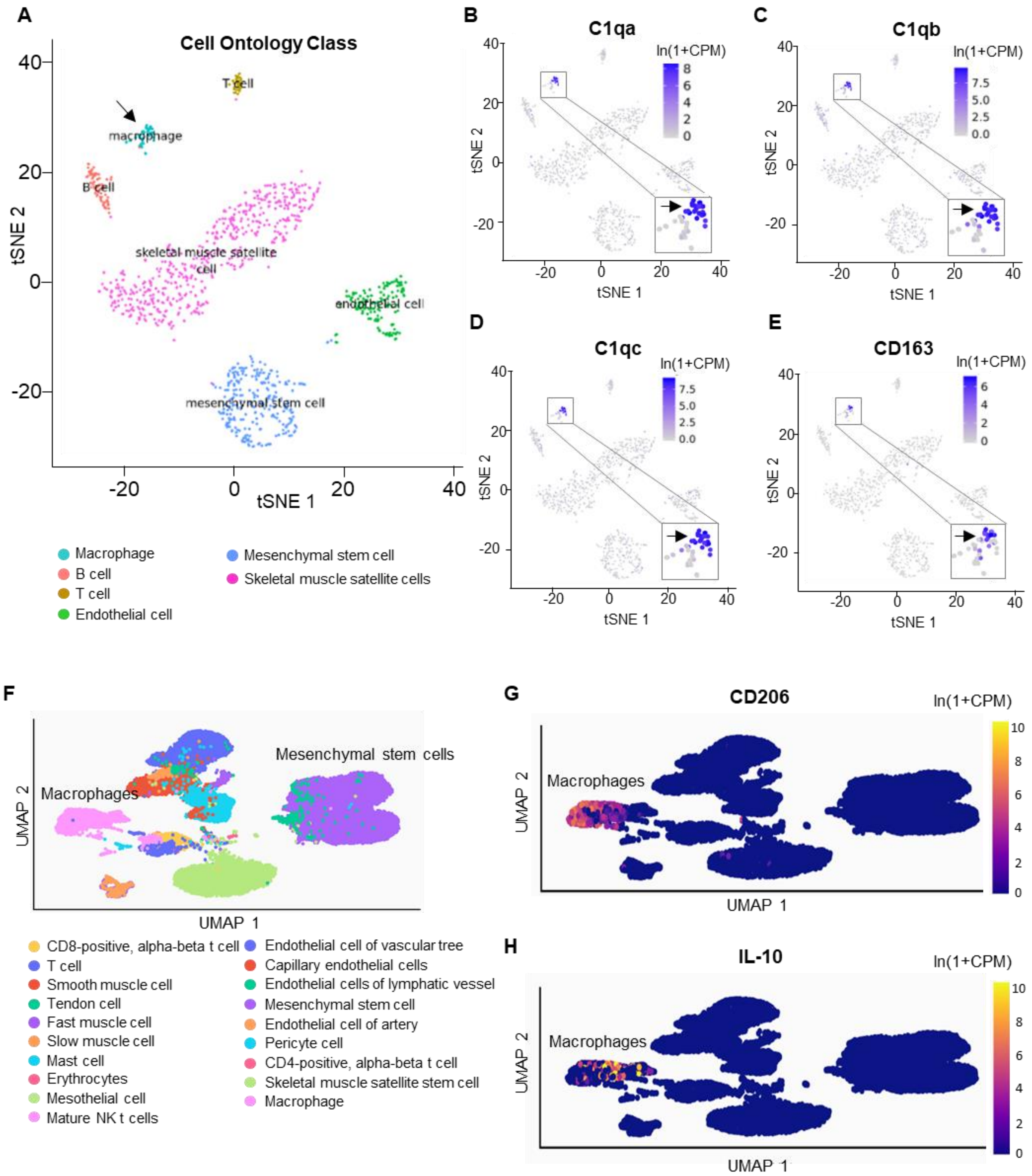
A
Cell Ontology Class
T cell
macrophage
B cell
skeletal muscle satellite cell
endothelial cell
mesenchymal stem cell
tSNE 1
tSNE 2
Macrophage
B cell
T cell
Endothelial cell
Mesenchymal stem cell
Skeletal muscle satellite cells
B
C1qa
C
C1qb
D
C1qc
E
CD163
ln(1+CPM)
F
Macrophages
Mesenchymal stem cells
UMAP 1
UMAP 2
CD8-positive, alpha-beta t cell
T cell
Smooth muscle cell
Tendon cell
Fast muscle cell
Slow muscle cell
Mast cell
Erythrocytes
Mesothelial cell
Mature NK t cells
Endothelial cell of vascular tree
Capillary endothelial cells
Endothelial cells of lymphatic vessel
Mesenchymal stem cell
Endothelial cell of artery
Pericyte cell
CD4-positive, alpha-beta t cell
Skeletal muscle satellite stem cell
Macrophage
G
CD206
H
IL-10
Macrophages

**Appendix Figure S9. Complement expression in different subsets of macrophages in resting muscles.**

**A**. tSNE plot showing the mouse cell types within the limb muscle in which the expression of C1 components and CD163 was evaluated by interrogating the Tabula Muris database derived from a FACS-based full-length transcript analysis (Data ref: Tabula Muris Consortium *et al*, 2018).

**B-E.** C1qa (B), C1qb (C), C1qc (D), and CD163 (E) are heterogeneously expressed by macrophages in mouse limb muscles. Magnification sections and arrows indicate the overlapping expression of C1q components and CD163 in the same fraction of macrophages.

**F.** UMAP plot showing the human muscle cell types present in skeletal muscle identified according to the Tabula Sapiens single-cell transcriptomics analysis (Data ref: Jones et al, 2022).

**G, H.** UMAP plots showing the expression of CD206 (G) and IL-10 (H) in human muscle cell types as indicated in (F). Note the overlap between the CD206 subset of macrophages and the cells expressing C1qa, C1qb, and C1qc (Appendix Figure S6 A-D). IL-10 marks a different subset of macrophages.

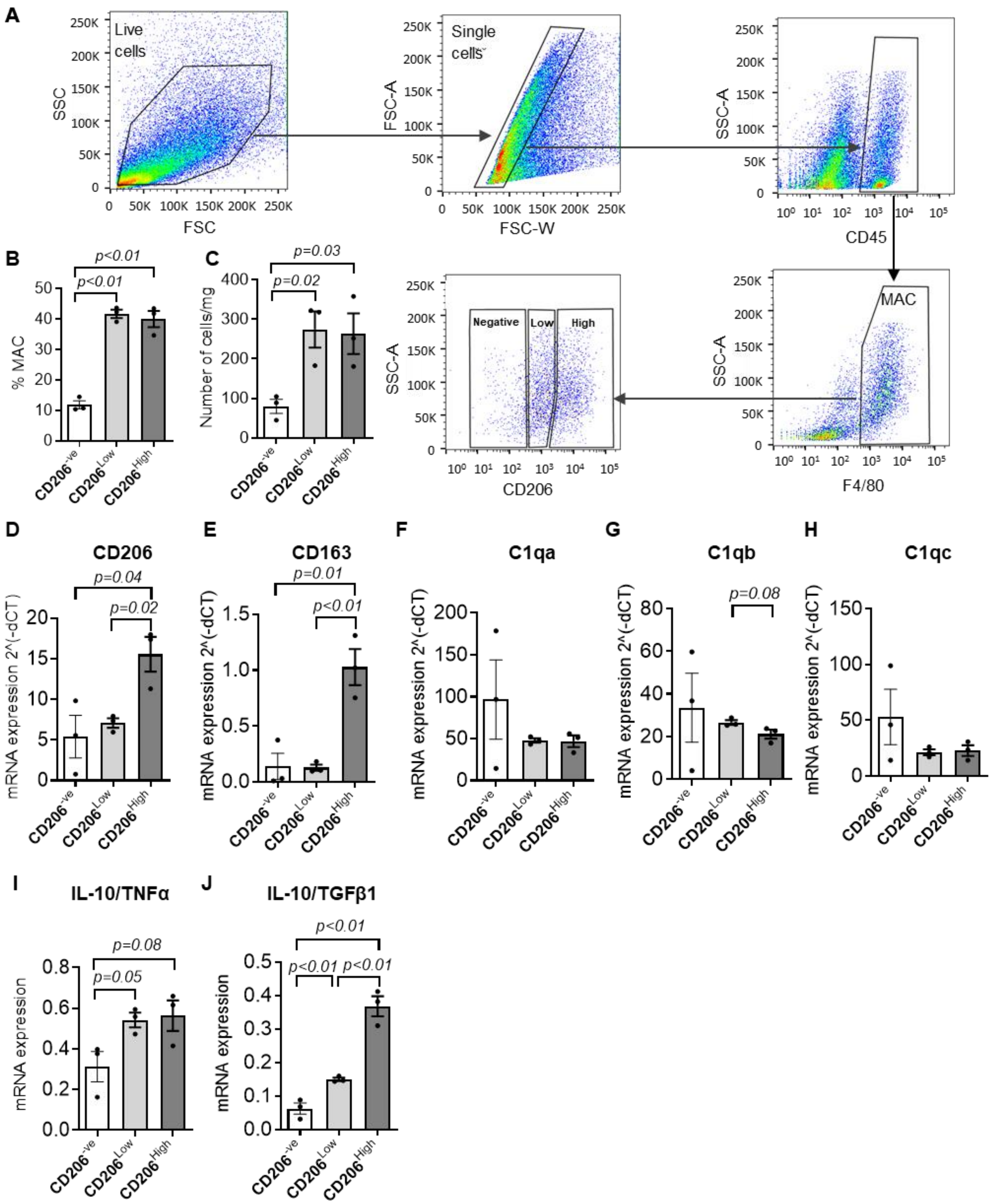
A
Live cells
SSC
FSC
Single cells
FSC-A
FSC-W
SSC-A
CD45
MAC
F4/80
Negative
Low
High
CD206
B
% MAC
p<0.01
p<0.01
C
Number of cells/mg
p=0.03
p=0.02
D
CD206
p=0.04
p=0.02
E
CD163
p=0.01
p<0.01
F
C1qa
G
C1qb
p=0.08
H
C1qc
mRNA expression 2^(-dCT)
I
IL-10/TNFα
p=0.08
p=0.05
J
IL-10/TGFβ1
p<0.01
p<0.01
p<0.01
mRNA expression
CD206-ve
CD206Low
CD206High

**Appendix Figure S10. Complement expression in different subsets of macrophages in WT injured muscles.**

A. Representative gating and sorting strategy used to FACS-isolate different macrophage populations from *WT* mice 8 days after cardiotoxin injury. Macrophages were gated as $CD45^{+ve}F4/80^{+ve}$ cells. Within the macrophage population, $CD206^{-ve}$, $CD206^{Low}$, and $CD206^{High}$ subpopulations were isolated.

B, C. Number of $CD206^{-ve}$, $CD206^{Low}$, and $CD206^{High}$ macrophages from injured *WT* mice processed and FACS-isolated as in A. Data are expressed as a percentage of $CD45^{+ve}$ cells (B) and as the number of cells per mg of tissue (C). N (biological samples) = 3.

D-H. *CD206* (D), *CD163* (E), *C1qa* (F), *C1qb* (G), and *C1qc* (H) mRNA expression in $CD206^{-ve}$, $CD206^{Low}$, and $CD206^{High}$ macrophages from injured *WT* mice FACS-isolated as in A. N (biological samples) = 3.

I-J. *IL-10/TNFα* (I) and *IL-10/TGFβ1* (J) mRNA expression in $CD206^{-ve}$, $CD206^{Low}$ and $CD206^{High}$ macrophages from injured *WT* mice FACS-isolated as in A. N (biological samples) = 3.

Data information: Data are presented as mean ± SEM. Statistical differences between two groups were calculated by unpaired two-tailed Student's test. P values are as indicated.

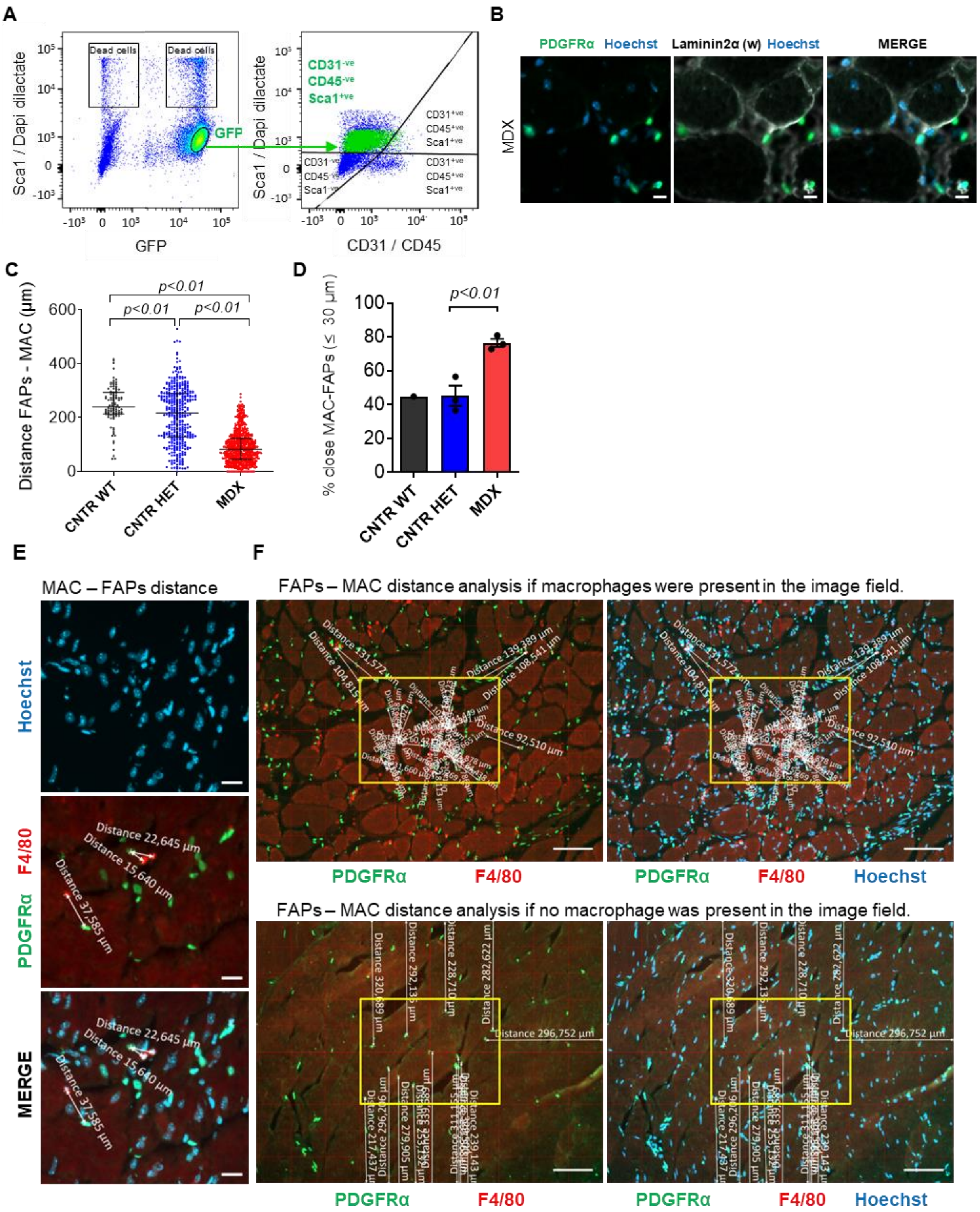

A
Dead cells
Dead cells
GFP
Sca1 / Dapi dilactate
GFP
CD31-ve
CD45-ve
Sca1+ve
CD31+ve
CD45+ve
Sca1+ve
CD31+ve
CD45+ve
Sca1-ve
CD31-ve
CD45
Sca1-ve
CD31 / CD45
B
PDGFRα Hoechst
Laminin2α (w) Hoechst
MERGE
MDX
C
Distance FAPs - MAC (μm)
p<0.01
p<0.01
p<0.01
CNTR WT
CNTR HET
MDX
D
% close MAC-FAPs (≤ 30 μm)
p<0.01
CNTR WT
CNTR HET
MDX
E
MAC – FAPs distance
Hoechst
F4/80
PDGFRα
MERGE
Distance 22,645 μm
Distance 15,640 μm
Distance 37,585 μm
F
FAPs – MAC distance analysis if macrophages were present in the image field.
PDGFRα
F4/80
Hoechst
FAPs – MAC distance analysis if no macrophage was present in the image field.
PDGFRα
F4/80
Hoechst

**Appendix Figure S11. Analysis of distance between macrophages and FAPs in muscles.**

**A.** Representative FACS plot of hindlimb muscles of a ~1 year-old male *PDGFRα*$^{eGFP/WT}$*;MDX* showing that eGFP$^{+ve}$ cells have the same staining signature as FAPs (i.e., CD31$^{-ve}$CD45$^{-ve}$Sca1$^{+ve}$). An intense dapi dilactate stain was used to gate out dead cells.

**B.** Representative immunofluorescence of *gastrocnemius* of a ~18 months-old *PDGFRα* $^{eGFP/WT}$*;MDX* male mouse stained with anti-Laminin2α antibodies (white) and Hoechst (blue). Most of eGFP$^{+ve}$ cells were found in the interstitial space, the typical FAPs localization. Scale bar: 10 µm.

**C.** Distance (µm) between FAPs and macrophages (MAC) in the same muscles as in Figure 4A. The analysis was performed by measuring the distance of each FAP in the image field from its closest macrophage (refer to F). N (biological samples) = 3 (CNTR *HET*, *MDX*), 1 (CNTR *WT*).

**D.** Percentage of macrophages and FAPs close to each other 30 µm or less calculated in the same muscles as in Figure 4A. N (biological samples) = 3 (CNTR *HET*, *MDX*), 1 (CNTR *WT*).

**E.** Representative distance analysis between macrophages (MAC: F4/80$^{+ve}$ cells) and FAPs (PDGFαr$^{+ve}$ cells) (see Figure 4A). The analysis was performed by measuring the distance of each macrophage from its closest FAP within the image field. *Gastrocnemius* section of ~ 18-months old *PDGFRα*$^{eGFP/WT}$*;MDX*$^{+/-}$ heterozygous female (CNTR *HET*) mouse is shown. Scale bar: 20 µm. Staining: anti-F4/80 (red), Hoechst (blue).

**F.** Representative distance analysis between FAPs (PDGFαr$^{+ve}$ cells) and macrophages (MAC: F4/80$^{+ve}$ cells) (refer to C). The analysis was performed by measuring the distance of each FAP from its closest macrophage within a fixed portion of the image field (100 µm$^2$, yellow rectangle). If no macrophage was present in the entire image field (bottom panel), the distance

was estimated between a FAP and the image edge. *Gastrocnemius* section of ~ 12-months old *PDGFRα*$^{eGFP/WT}$*;MDX* (*MDX*) male mouse (top panel) and ~ 18-months old *PDGFRα*$^{eGFP/WT}$*;MDX*$^{+/-}$ heterozygous female (CNTR *HET*) mouse (bottom panel) are shown. Note that clusters of macrophages and FAPs were observed mostly in the *MDX* regenerating areas (top panel). Scale bar: 100 µm.

Data information: In C, data are presented as median with interquartile range. Each dot on the graph represents a distance measurement (99 for *WT*, 381 for CNTR *HET*, and 672 for *MDX*). Statistical differences were calculated by Kruskal-Wallis test. Dunn's multiple comparison test was used as *post hoc* test. In D, data are presented as mean ± SEM. Statistical differences between CNTR *HET* and *MDX* groups were calculated by unpaired two-tailed Student's test. P values are as indicated.

178

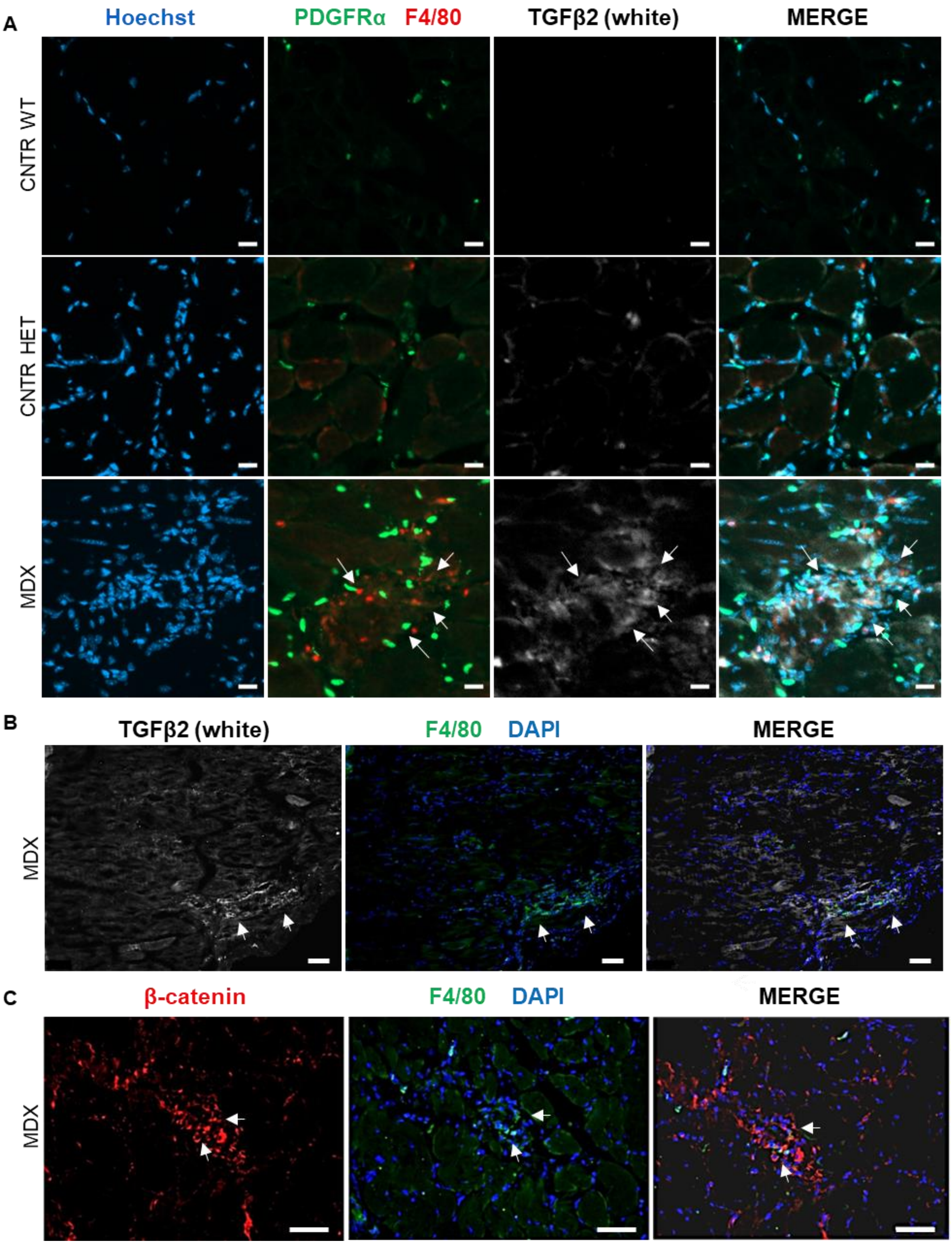
A
Hoechst
PDGFRα F4/80
TGFβ2 (white)
MERGE
CNTR WT
CNTR HET
MDX
B
TGFβ2 (white)
F4/80 DAPI
MERGE
MDX
C
β-catenin
F4/80 DAPI
MERGE
MDX

**Appendix Figure S12. WNT-signaling target proteins co-localize with macrophages and FAPs in the dystrophic regenerating areas.**

**A.** Representative immunofluorescence image of *gastrocnemius* of ~12-months old male *PDGFRα*$^{eGFP/WT}$ (CNTR *WT*), ~18-months old female *PDGFRα*$^{eGFP/WT}$*;MDX*$^{+/-}$ (CNTR HET) and ~18-months old male *PDGFRα*$^{eGFP/WT}$*;MDX* (*MDX*) mice stained with anti-F4/80 (red) and anti-TGFβ2 (white) antibodies, and Hoechst (blue). The number of cells (macrophages and FAPs) and the TGFβ2 protein expression are increased in the *MDX* muscles compared to the controls. White arrows in the *MDX* sample indicate TGFβ2 that co-localizes with macrophages (F4/80$^{+ve}$ cells) and FAPs (PDGFRα$^{+ve}$ cells) within the regenerating area of the muscle. Scale bar: 20 µm.

**B, C.** Representative immunofluorescence image of a diaphragm of a ~1 year-old male *MDX* stained with anti-TGFβ2 (white), anti-F4/80 (green), anti-β-catenin (red) antibodies and DAPI (blue). White arrows indicate the localization of TGFβ2 and F4/80 (B) and β-catenin and F4/80 (C) in the same muscle areas. Scale bar: 50 µm.

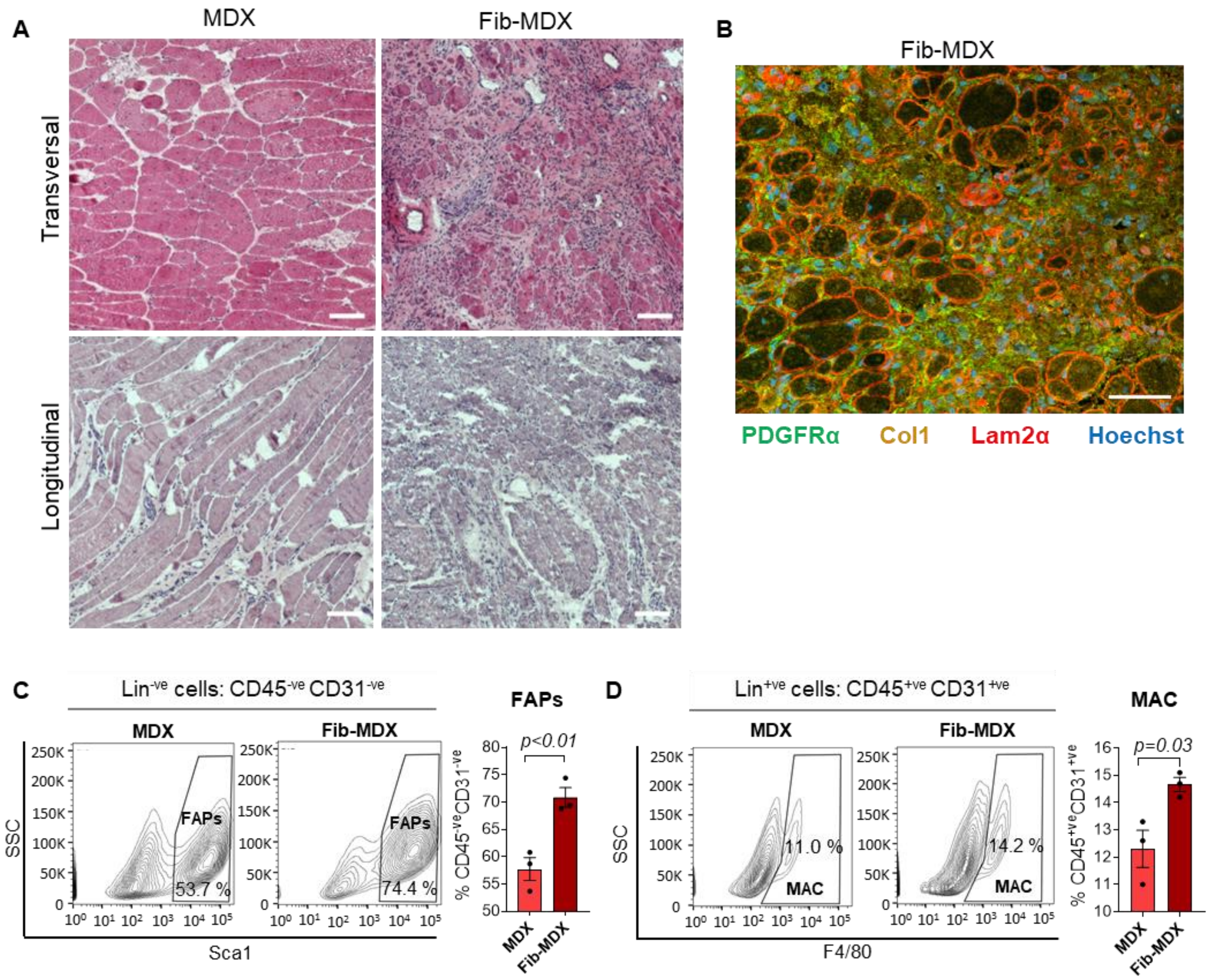


**Appendix Figure S13. Analysis of the fib-MDX skeletal muscle.**

**A.** Representative images of H&E staining of ~1 year-old *MDX* and fib-*MDX gastrocnemius* muscles. Transversal and longitudinal images are from different biological samples. Scale bar: 20 µm

**B.** Immunofluorescence image of a *gastrocnemius* of a fib-*MDX* presented in Figure 5B with the additional staining with an anti-PDGFRα antibody (green). Scale bar: 50 µm.

**C, D.** Representative FACS plots (left) and quantification (right) of FAPs (C) and macrophages (D) expressed respectively as the percentage of Lin$^{-ve}$ and Lin$^{+ve}$ cells in fib-*MDX* or uninjured *MDX* hindlimb muscles. N (biological samples) = 3.

Data information: In C and D, data are presented as mean ± SEM. Statistical differences were calculated by unpaired two-tailed Student's test. P values are as indicated.

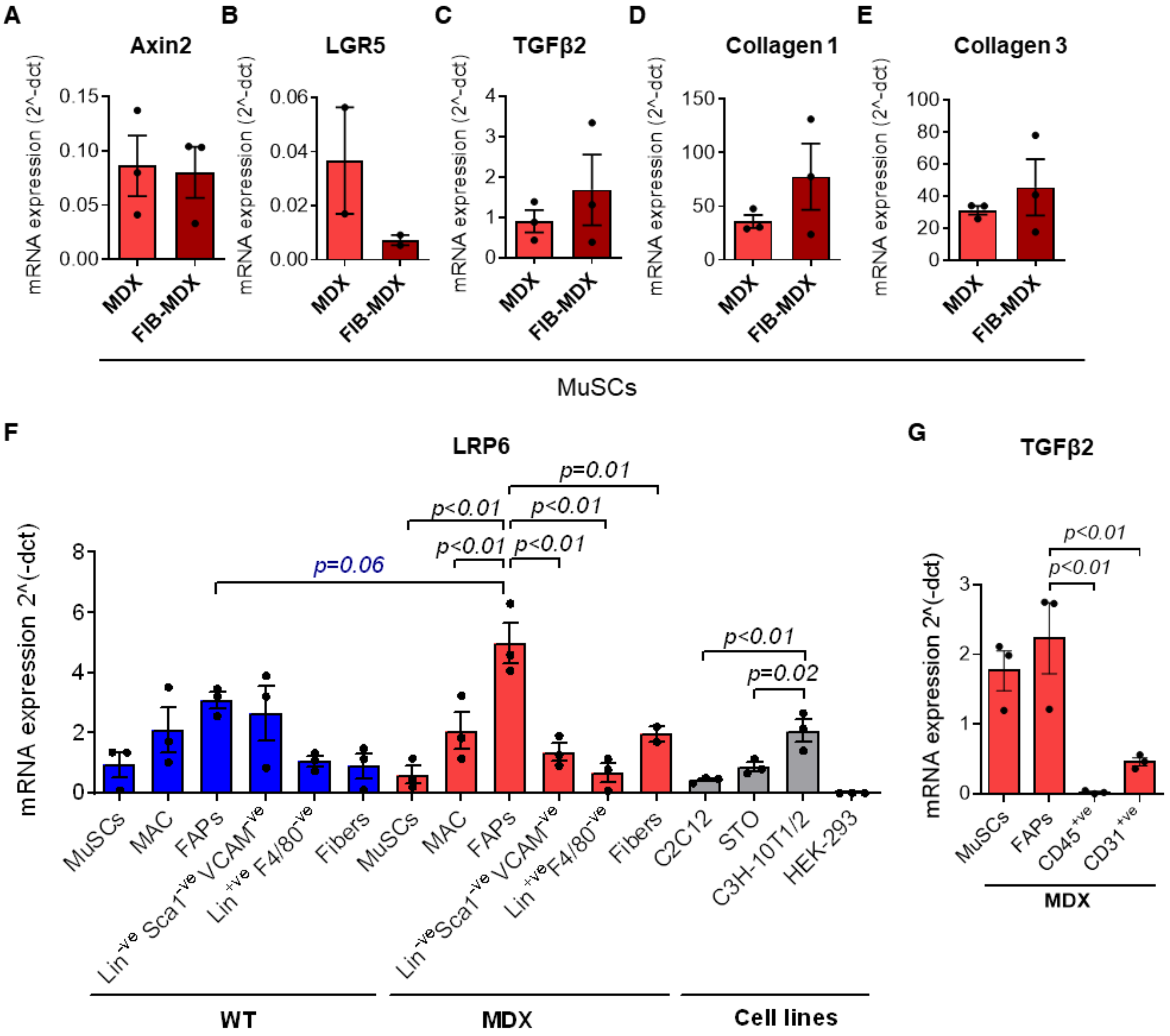

**Appendix Figure S14. Analysis of MuSCs in the fib-MDX mouse model and cellular LRP6 expression.**

**A-E.** *Axin2* (A), *LGR5* (B), *TGFβ2* (C), *Collagen1a1* (D), and *Collagen3a1* (E) mRNA expression in MuSCs FACS-isolated from hindlimb muscles of ~6 months-old *MDX* and fib-*MDX* mice. N (biological samples) = 3 for all, but LGR5 (N=2).

**F.** *LRP6* mRNA expression in MuSCs, macrophages, FAPs, $Lin^{+ve}F4/80^{-ve}$, $Lin^{-ve}Sca1^{-ve}VCAM^{-ve}$, and myofibers isolated from hindlimb muscles of ~1 year-old *WT* and *MDX* mice and in cell lines as indicated. HEK-293 (human cells) were used as a negative control as LRP6 primers amplify only the murine isoform. N (biological samples) = 3 (for all samples except *MDX* fibers, N=2).

G. *TGFβ2* mRNA expression in MuSCs, FAPs, $CD45^{+ve}$, and $CD31^{+ve}$ cells FACS-isolated from the hindlimb muscles of *MDX* mice. N (biological samples) = 3.

Data information: Data are presented as mean ± SEM. In A-E, Statistical differences were calculated by unpaired two-tailed Student's test. In F-G statistical differences between three or more groups were calculated by one-way ANOVA test. Tukey's multiple comparison test was used as a *post hoc* test, and all the corresponding p values are reported on the graph; in F, the statistical difference between two groups was calculated by unpaired two-tailed Student's t-test, and the corresponding p value is reported in blue on the graph.

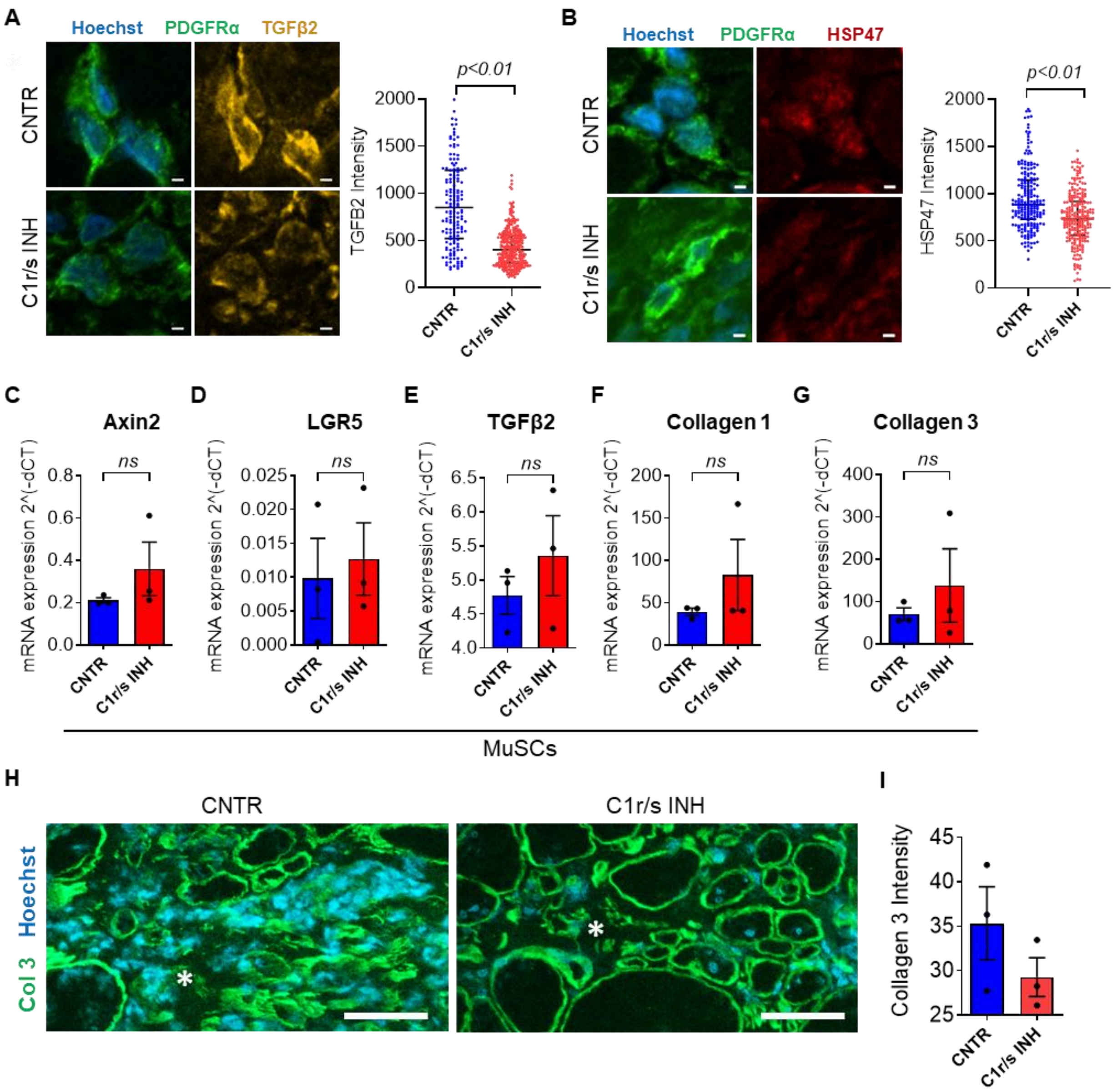


**Appendix Figure S15. Analysis of WNT-signaling targets and fibrotic markers after in vivo C1 inhibition in the fib-MDX mouse.**

**A, B.** Representative immunofluorescence (left) and quantification (right) of TGFβ2 (A) and HSP47 (B) signal intensities in the PDGFRα$^{+ve}$ cells (i.e., FAPs) of *gastrocnemius* of ~1 year-old *MDX* mice processed as in Figure 6A and stained with anti-PDGFRα (green), anti-TGFβ2

(yellow in A), anti-HPS47 (red in B) antibodies and Hoechst (blue). N (biological replicates) = 3. Scale bar: 2 µm.

**C-G.** *Axin2* (C), *LGR5* (D), *TGFβ2* (E), *Collagen1a1* (F), and *Collagen3a1* (G) mRNA expression in FACS-isolated MuSCs from ~1 year-old *MDX* mice processed as described in Figure 6A. N (biological samples) = 3.

**H.** Representative immunofluorescence of *gastrocnemius* muscles from ~1 year-old *MDX* mice processed as in Figure 6A. Muscles were stained with anti-Collagen 3 (green) and Hoechst (blue). Scale bar: 100 µm. The interstitial presence of collagen 3 is indicated by the asterisk.

**I.** Quantification of collagen 3 pixel-intensity in the interstitial space between myofibers of the same muscles as in (H). N (biological samples) = 3.

Data information: Data are presented as median with interquartile range in A and B and as mean ± SEM in C-G and in I. In A and B, dots represent single cells' measurements (155 for CNTR and 320 for C1r/s INH in TGFβ2 analysis, and 209 for CNTR and 234 for C1r/s INH in HSP47 analysis). In I, each graph dot represents the average value of 17 to 22 measurements on different muscle regions for each biological sample. Statistical differences were calculated by the Mann-Whitney test in A and B, and the paired two-tailed Student's test in C-G and I. P values are as indicated.

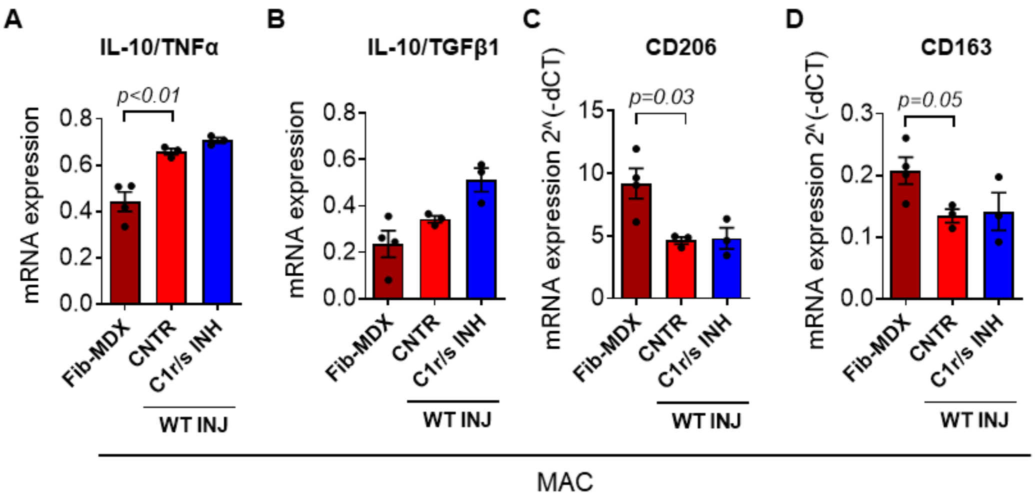


**Appendix Figure S16. Macrophages characterization 8 days after acute injury.**

**A, B.** *IL-10*/*TNFα* (A), *IL-10*/*TGFβ1* (B) mRNA expression in FACS-isolated macrophages from fib-*MDX* mice and *WT* mice processed as in Figure EV5A. N (biological samples) = 3. Fib-*MDX* data are the same as shown in Figure EV4E-F.

**C, D.** *CD206* (G), *CD163* (H) mRNA expression in macrophages FACS-isolated as in A, B. N (biological replicates) = 3. Fib-*MDX* data are the same as shown in Figure EV4G-H.

Data information: Data are presented as mean ± SEM. Statistical differences between fib-*MDX* and *WT* INJ CNTR groups were calculated by unpaired two-tailed Student's test. P values are as indicated.

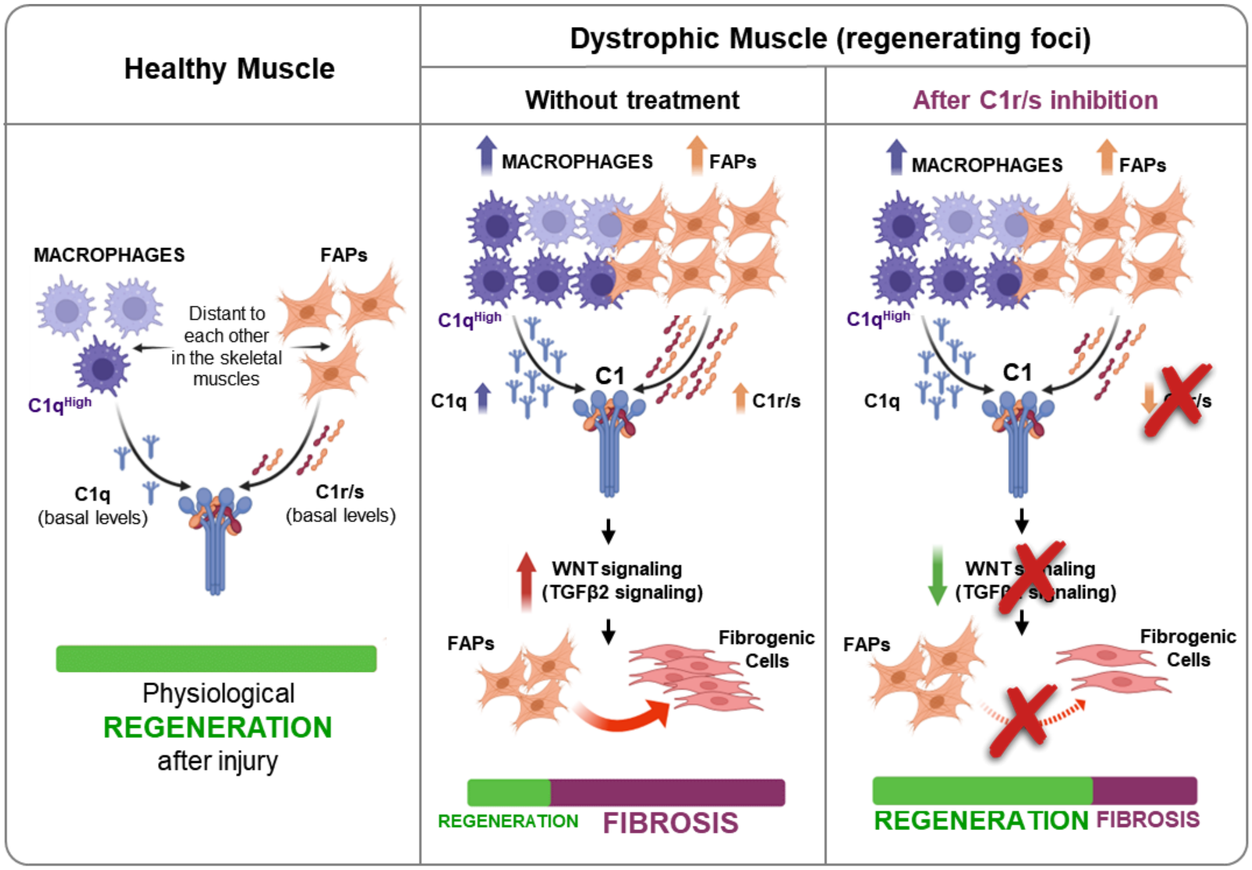


**Appendix Figure S17. Combinatorial activation of the WNT-dependent fibrogenic program by distinct complement subunits in dystrophic muscle.**

Model of the mechanism through which the C1/WNT axis is activated in the regenerating foci of dystrophic muscles. In dystrophic muscles, FAPs and macrophages act as a combinatorial source of WNT-activity by secreting distinct subunits of the C1 complement complex, promoting a fibrogenic phenotype in responsive cells, such as fibro-adipogenic progenitors. Pharmacologically interfering with the activity of C1r/s is inhibiting WNT-signaling in fibro-adipogenic progenitors and ameliorating the dystrophic phenotype. During acute physiological regeneration, the repertoire of FAPs and macrophages is different and less effective in producing complement C1.

## APPENDIX TABLES

**Appendix Table S1. List of human biopsies used for complement and WNT-signaling analysis.**

| ID in Figures | Muscle | Age (years) | Mutation on dystrophin gene | Performed analysis |
|---|---|---|---|---|
| CNTR 1 in Fig.1J, L | Biceps | 15 | not applicable | qPCR, Western Blot |
| CNTR 2 in Fig.1J, L | Biceps | 17 | not applicable | qPCR, Western Blot |
| CNTR 3 in Fig.1J, L | Biceps | 16 | not applicable | qPCR, Western Blot |
| CNTR 1 in Appendix Fig. S1B-C | Biceps | 40 | not applicable | Immunofluorescence |
| CNTR 2 in Appendix Fig. S1B-C | Biceps | 25 | not applicable | Immunofluorescence |
| CNTR in Fig. 1M and CNTR 3 in Appendix Fig. S1B-C | Biceps | unknown | not applicable | Immunofluorescence |
| CNTR 1 in Appendix Fig. S6G, H | Lumbar | 60 | not applicable | Immunofluorescence |
| CNTR 2 in Appendix Fig. S6G, H | Lumbar | 42 | not applicable | Immunofluorescence |
| CNTR 3 in Appendix Fig. S6G, H | Cervical | 68 | not applicable | Immunofluorescence |
| BMD in Fig. 1M | Biceps | 8 | DEL3-7 | Immunofluorescence |
| DMD 1 in Fig.1J, L | Biceps | 12 | DEL49-50 | qPCR, Western Blot |
| DMD 2 in Fig.1J, L | Biceps | 12 | 10219G>T | qPCR, Western Blot |
| DMD 3 in Fig.1J, L | Biceps | 11 | DUP44-47 | qPCR, Western Blot |
| DMD 4 in Fig.1J, L | Biceps | 13 | DUP22 | qPCR, Western Blot |
| DMD 1 in Appendix Fig. S1B-C | Quadriceps | 1 | DUP52 | Immunofluorescence |
| DMD 2 in Appendix Fig. S1B-C | Biceps | 9 | DUP1-9 | Immunofluorescence |
| DMD 3 in Appendix Fig. S1B-C | Biceps | 14 | DEL51 | Immunofluorescence |
| DMD 4 in Appendix Fig. S1B | Biceps | 9 | 8668G>A | Immunofluorescence |

Muscles, age, and dystrophin gene mutations of dystrophic patients and healthy controls (CNTR) analyzed for the expression of the subunits of the C1 complex and WNT-signaling targets are indicated (see **Figure 1J, L, M, Appendix Figure S1B, C** and **Appendix Figure S6G, H**). BMD, Beker muscular dystrophy.

**Appendix Table S2. Statistical analysis of C1qa, C1qb and C1qc expression in WT and MDX cells.**

| Analyzed comparison | P value (One-Way ANOVA test) | | | | | | | | |
|---|---|---|---|---|---|---|---|---|---|
| | WT | | | MDX | | | WT INJ | | |
| | C1qa | C1qb | C1qc | C1qa | C1qb | C1qc | C1qa | C1qb | C1qc |
| **MAC vs. MuSCs** | **<0.01** | **<0.01** | **<0.01** | **<0.01** | **<0.01** | **0.06** | **<0.01** | **0.02** | **0.03** |
| **MAC vs. FAPs** | **<0.01** | **<0.01** | **<0.01** | **<0.01** | **<0.01** | **0.05** | **<0.01** | **0.02** | **0.03** |
| **MAC vs. Lin$^{-ve}$ Sca1$^{-ve}$ VCAM$^{-ve}$** | **<0.01** | **<0.01** | **<0.01** | **<0.01** | **<0.01** | 0.65 | **<0.01** | **0.03** | 0.19 |
| **MAC vs. Lin$^{+ve}$ F4/80$^{-ve}$** | **<0.01** | **<0.01** | **<0.01** | **<0.01** | **<0.01** | **0.05** | **<0.01** | **0.02** | **0.03** |
| **MAC vs. Fibers** | **<0.01** | **<0.01** | **<0.01** | **<0.01** | **<0.01** | **0.02** | NA | NA | NA |
| MuSCs vs. FAPs | >0.99 | >0.99 | >0.99 | >0.99 | >0.99 | >0.99 | >0.99 | >0.99 | >0.99 |
| MuSCs vs. Lin$^{-ve}$ Sca1$^{-ve}$ VCAM$^{-ve}$ | >0.99 | >0.99 | >0.99 | 0.99 | >0.99 | 0.58 | 0.90 | >0.99 | 0.72 |
| MuSCs vs. Lin$^{+ve}$ F4/80$^{-ve}$ | >0.99 | >0.99 | >0.99 | >0.99 | >0.99 | >0.99 | 0.95 | >0.99 | >0.99 |
| MuSCs vs. Fibers | >0.99 | >0.99 | >0.99 | >0.99 | >0.99 | >0.99 | NA | NA | NA |
| FAPs vs. Lin$^{-ve}$ Sca1$^{-ve}$ VCAM$^{-ve}$ | >0.99 | >0.99 | >0.99 | >0.99 | >0.99 | 0.48 | 0.96 | >0.99 | 0.73 |
| FAPs vs. Lin$^{+ve}$ F4/80$^{-ve}$ | >0.99 | >0.99 | >0.99 | >0.99 | >0.99 | >0.99 | 0.99 | >0.99 | >0.99 |
| FAPs vs. Fibers | >0.99 | >0.99 | >0.99 | >0.99 | >0.99 | >0.99 | NA | NA | NA |
| Lin$^{-ve}$ Sca1$^{-ve}$ VCAM$^{-ve}$ vs. Lin$^{+ve}$ F4/80$^{-ve}$ | >0.99 | >0.99 | >0.99 | >0.99 | >0.99 | 0.50 | >0.99 | >0.99 | 0.79 |
| Lin$^{-ve}$ Sca1$^{-ve}$ VCAM$^{-ve}$ vs. Fibers | >0.99 | >0.99 | >0.99 | 0.99 | >0.99 | 0.34 | NA | NA | NA |
| Lin$^{+ve}$ F4/80$^{-ve}$ vs. Fibers | >0.99 | >0.99 | >0.99 | >0.99 | >0.99 | >0.99 | NA | NA | NA |

One-way ANOVA test performed on *WT* and *MDX* cells shown in **Figure 2A-C**. Tukey's multiple comparison test was used as a *post hoc test*. Statistically significant differences are indicated in bold.

**Appendix Table S3. Statistical analysis of C1r, C1s and E2F1 expression in WT and MDX cells.**

| Analyzed comparison | P value (One-Way ANOVA test) | | | | | | | | |
|---|---|---|---|---|---|---|---|---|---|
| | WT | | | MDX | | | WT INJ | | |
| | C1r | C1s | E2F1 | C1r | C1s | E2F1 | C1r | C1s | E2F1 |
| MAC vs. MuSCs | >0.99 | >0.99 | **0.02** | >0.99 | >0.99 | 0.35 | >0.99 | >0.99 | 0.19 |
| MAC vs. Lin$^{-ve}$ Sca1$^{-ve}$ VCAM$^{-ve}$ | 0.27 | **<0.01** | **0.03** | 0.87 | **<0.01** | 0.06 | 0.89 | **0.02** | 0.10 |
| MAC vs. Lin$^{+ve}$ F4/80$^{-ve}$ | >0.99 | >0.99 | **0.03** | >0.99 | >0.99 | 0.28 | >0.99 | >0.99 | 0.10 |
| MAC vs. Fibers | >0.99 | >0.99 | **0.01** | >0.99 | >0.99 | NA | NA | NA | **0.02** |
| MuSCs vs. Lin$^{-ve}$ Sca1$^{-ve}$ VCAM$^{-ve}$ | 0.32 | **<0.01** | >0.99 | 0.71 | **<0.01** | 0.74 | 0.93 | **0.03** | >0.99 |
| MuSCs vs. Lin$^{+ve}$ F4/80$^{-ve}$ | >0.99 | >0.99 | >0.99 | >0.99 | >0.99 | >0.99 | >0.99 | >0.99 | >0.99 |
| MuSCs vs. Fibers | 0.99 | >0.99 | >0.99 | >0.99 | >0.99 | NA | NA | NA | 0.79 |
| **FAPs vs. MAC** | **<0.01** | **<0.01** | >0.99 | **<0.01** | **<0.01** | >0.99 | **<0.01** | **<0.01** | 0.46 |
| **FAPs vs. MuSCs** | **<0.01** | **<0.01** | **0.02** | **<0.01** | **<0.01** | 0.27 | **<0.01** | **<0.01** | **<0.01** |
| **FAPs vs. Lin$^{-ve}$ Sca1$^{-ve}$ VCAM$^{-ve}$** | **<0.01** | 0.88 | **0.02** | **<0.01** | **<0.01** | **0.04** | **<0.01** | 0.84 | **<0.01** |
| **FAPs vs. Lin$^{+ve}$ F4/80$^{-ve}$** | **<0.01** | **<0.01** | **0.02** | **<0.01** | **<0.01** | 0.22 | **<0.01** | **<0.01** | **<0.01** |
| **FAPs vs. Fibers** | **<0.01** | **<0.01** | **<0.01** | **<0.01** | **<0.01** | NA | NA | NA | **<0.01** |
| Lin$^{-ve}$ Sca1$^{-ve}$ VCAM$^{-ve}$ vs. Lin$^{+ve}$ F4/80$^{-ve}$ | 0.27 | **<0.01** | >0.99 | 0.69 | **<0.01** | 0.82 | 0.85 | **0.02** | >0.99 |
| Lin$^{-ve}$ Sca1$^{-ve}$ VCAM$^{-ve}$ vs. Fibers | 0.18 | **<0.01** | >0.99 | 0.72 | **<0.01** | NA | NA | NA | 0.94 |
| Lin$^{+ve}$ F4/80$^{-ve}$ vs. Fibers | >0.99 | >0.99 | >0.99 | >0.99 | >0.99 | NA | NA | NA | 0.94 |

One-way ANOVA test performed on *WT* and *MDX* cells shown in **Figure 2D-E** and in **Appendix Figure S7A**. Tukey's multiple comparison test was used as a *post hoc test*. Statistically significant differences are indicated in bold.

**Appendix Table S4. List of primary antibodies used for FACS-isolation of murine and human cells.**

| Antibody | Company | Dilution |
|---|---|---|
| APC/Fire 750 anti-mouse CD45 | BioLegend (103154) | 1:100 |
| APC/Fire 750 anti-mouse CD31 | BioLegend (102434) | 1:100 |
| FITC anti-mouse F4/80 | BioLegend (123107) | 1:100 |
| Brillant Violet 421 anti-mouse Ly-6A/E (Sca1) | BioLegend (108127) | 1:100 |
| Biotin anti-mouse CD016 (VCAM) | BioLegend (105704) | 1:100 |
| APC anti-mouse CD45 | BioLegend (103112) | 1:100 |
| APC anti-mouse CD31 | BioLegend (102410) | 1:100 |
| Pacific Blue anti-mouse Ly-6A/E (Sca-1) | BioLegend (108120) | 1:100 |
| PE anti-mouse CD206 | Biotechne (FAB2535P) | 1:20 |
| APC anti-mouse F4/80 | BioLegend (123116) | 1:100 |
| FITC anti-mouse CD45 | BioLegend (103108) | 1:100 |
| Pacific Blue anti-human CD45 | BioLegend (304021) | 1:100 |
| Pacific Blue anti-human CD31 | BioLegend (303113) | 1:100 |
| PE anti-human CD34 | Miltenyi (130-081-002) | 1:30 |

**Appendix Table S5. Calculation of STO/RAW-264.7 conditioned media ratio used to treat murine cells.**

| MDX Hindlimb Muscles ~ 1 year-old | % of total cell number | |
|---|---|---|
| | **Macrophages** | **FAPs** |
| Biological Replicate 1 | 3.60 | 9.60 |
| Biological Replicate 2 | 3.30 | 13.70 |
| Biological Replicate 3 | 3.39 | 10.4 |
| Average | 3.43 | 11.2 |
| StDev | 0.15 | 2.17 |

FAPs/MAC = 11.2/3.43 = 3.27

Number of cells per area at confluence (inversed proportional to cellular size) = RAW-264.7/STO = 2.5

Used conditioned media ratio (vol/vol):

STO/RAW-254.7 = 3.27 x 2.5 = 8.18/1

STO/RAW-264.7 conditioned media ratio used to treat C2C12 cells (**Figure 3J-M**) was calculated considering the *in vivo* proportion of macrophages and FAPs in dystrophic mice and the number of STO and RAW-264.7 cells per area at confluence, when media was collected.

**Appendix Table S6. Primer list for SYBER Green RT-PCR.**

| Gene | Forward Primer 5'→3' | Reverse Primer 5'→3' | $T_{Ann}$ | Span an exon-exon junction? |
|---|---|---|---|---|
| mAxin2 | cagagggacaggaaccactc | tgccagtttctttggctctt | 60 °C | Yes |
| mC1qa | tctcagccattcggcagaac | tggttggtgaggaccttgtca | 60 °C | No |
| mC1qb | gggaatccactgctgtccggc | ctcagcctcaggggcttcctgt | 60 °C | Yes |
| mC1qc | agagccaggaatcccagccgtcc | gcatgccaggctcgccctt | 60 °C | No |
| mC1r | aaccatattacaagatgctgacca | ccttgggctgtgcaggta | 60 °C | Yes |
| mC1s | gaccagaggcaggagaggaggc | gctcagtgtcaccttcaggagc | 60 °C | Yes |
| mE2F1 | gaggctggatctggagactg | cccggagatttcacacctttc | 60 °C | Yes |
| mCol1a1 | tccggctcctgctcctctta | gtatgcagctgacttcagggatgt | 60 °C | Yes |
| mCol3a1 | gcccacagccttctacac | ccagggtcaccatttctc | 60 °C | Yes |
| mFibronectin1 | tgcctcgggaatggaaag | atggtaggtcttcccatcgtcata | 60 °C | Yes |
| mLGR5 | tcgccttccccaggtcccttc | gccgtggtccacaccccgat | 60 °C | Yes |
| mTGFβ2 | cgagcggagcgacgaggagt | tgggcgggatggcattttcgg | 60 °C | Yes |
| mHPRT | tcagaccgctttttgccgcga | atcgctaatcacgacgctgggac | 60 °C | No |
| h/mHPRT | aactggaaagaatgtcttgattgt | gaatttcaaatccaacaaagtctgg | 60 °C | Yes |
| mLRP6 | tcctcgagctctggcact | cctccccactcagtccaata | 60 °C | Yes |
| hC1qa | acaacaggaggcaggccc | acacagagcaccagccatc | 60 °C | Yes |
| hC1qb | agtaggctctcggctcctg | tcagacgcctcctgggaa | 60 °C | Yes |
| hC1qc | gggaagcagatctgaggacatc | cggagaaggaactgggca | 60 °C | Yes |
| hC1r | gctgagaacatgtctgtgaggtt | aggaggtacaagagccaccact | 60 °C | Yes |
| hC1s | agaggggttgcccagcat | ggctcagtgtcaccttcaga | 60 °C | Yes |
| mTNFα | aagttcccaaatggcctccc | tggtttgctacgacgtggg | 60 °C | Yes |
| mTGFβ1 | ccccactgatacgcctgagt | agccctgtattccgtctcctt | 60 °C | Yes |
| mCD206 | tataggtggagagctggcga | tccactgctcgtaatcagcc | 60 °C | Yes |
| mCD163 | tcctcaagaggagaggtcttg | ggaattttccgaggatttcagca | 60 °C | Yes |
| mIL-10 | ggcgctgtcatcgatttctc | atggccttgtagacaccttgg | 60 °C | Yes |

296 **Appendix Table S7. List of primary antibodies used for immunofluorescence.**

| Antigen | Specie | Company | Dilution |
|---|---|---|---|
| Axin2 | Rabbit | Abcam (32197) | 1:20 |
| TGFβ2 | Rabbit | Santa Cruz Biotechnology (sc-90) | 1:30 |
| C4 | Rat | Santa Cruz Biotechnology (16D2) | 1:50 |
| C1q | Rabbit | Abcam (182451) | 1:80 |
| C1q | Rat | Abcam (11861) | 1:50 |
| C1q | Mouse | Abcam (71940) | 1:100 |
| C1s | Rabbit | LS Bio (LS-C483829) | 1:100 |
| Laminin2α | Rat | Abcam (11576) | 1:1000 |
| GFP | Chicken | Aves Labs (1020) | 1: 250 |
| Collagen1 | Rabbit | Cederlane (CL50151AP) | 1:200 |
| Collagen 3 | Rabbit | Abcam (7778) | 1:200 |
| F4/80-Alexa Fluor 594 | Rat | BioLegend (123140) | 1:50 |
| β-catenin | Rabbit | Abcam (246504) | 1:100 |
| PDGFRα | Goat | Biotechne (AF1062) | 1:100 |
| HSP47 | Rabbit | Abclonal (A11698) | 1:50 |
