# Supplementary material for: Combinatorial activation of the WNT‐dependent fibrogenic program by distinct complement subunits in dystrophic muscle
Source: EMBO Mol Med. 2023 Nov 6;15(12):e17405. doi: 10.15252/emmm.202317405 (PMC10701616; doi:10.15252/emmm.202317405)
Supplement: Supplementary file 2 — Expanded View Figures PDF [file EMMM-15-e17405-s010.pdf]

Expanded View Figures

Figure EV1. Canonical WNT signaling is increased in dystrophic FAPs.

A, B Representative immunofluorescence (left) and quantification (right) of Axin2 (A) and TGFβ2 (B) signal intensities in the PDGFRα^{+ve} cells (i.e., FAPs) of the *gastrocnemius* of ~1-year-old *WT* and *MDX* stained with anti-Axin2 (yellow in A), anti-PDGFRα (green), anti-TGFβ2 (red in B), anti-laminin 2α (white) antibodies, and Hoechst (blue). High-magnification images in the left panels show representative PDGFRα^{+ve} cells. *N* (biological replicates) = 3 for all samples (except for *WT* TGFβ2 analysis, *N* = 2). Scale bar: 20 μm (low-magnification images), scale bar: 2 μm (high-magnification images).

C–H *Axin2* (C), *TGFβ2* (D), *LGR5* (E), *collagen 1a1* (F), *collagen 3a1* (G), and *fibronectin* (H) mRNA expression in FACS-isolated FAPs from ~3-months-old *WT* and *MDX* hindlimb muscles. *N* (biological replicates) = 3.

Data information: Data are presented as mean with interquartile range in (A and B) and as mean ± SEM in (C–H). In (A and B), dots represent single cells' measurements (131 for *WT* and 130 for *MDX* in Axin2 analysis, 126 for *WT* and 215 for *MDX* in TGFβ2 analysis). Statistical differences were calculated by unpaired two-tailed Mann–Whitney test in (A and B) and by unpaired two-tailed Student's *t*-test in (C–H). *P*-values are as indicated.
Source data are available online for this figure.

© 2023 The Authors

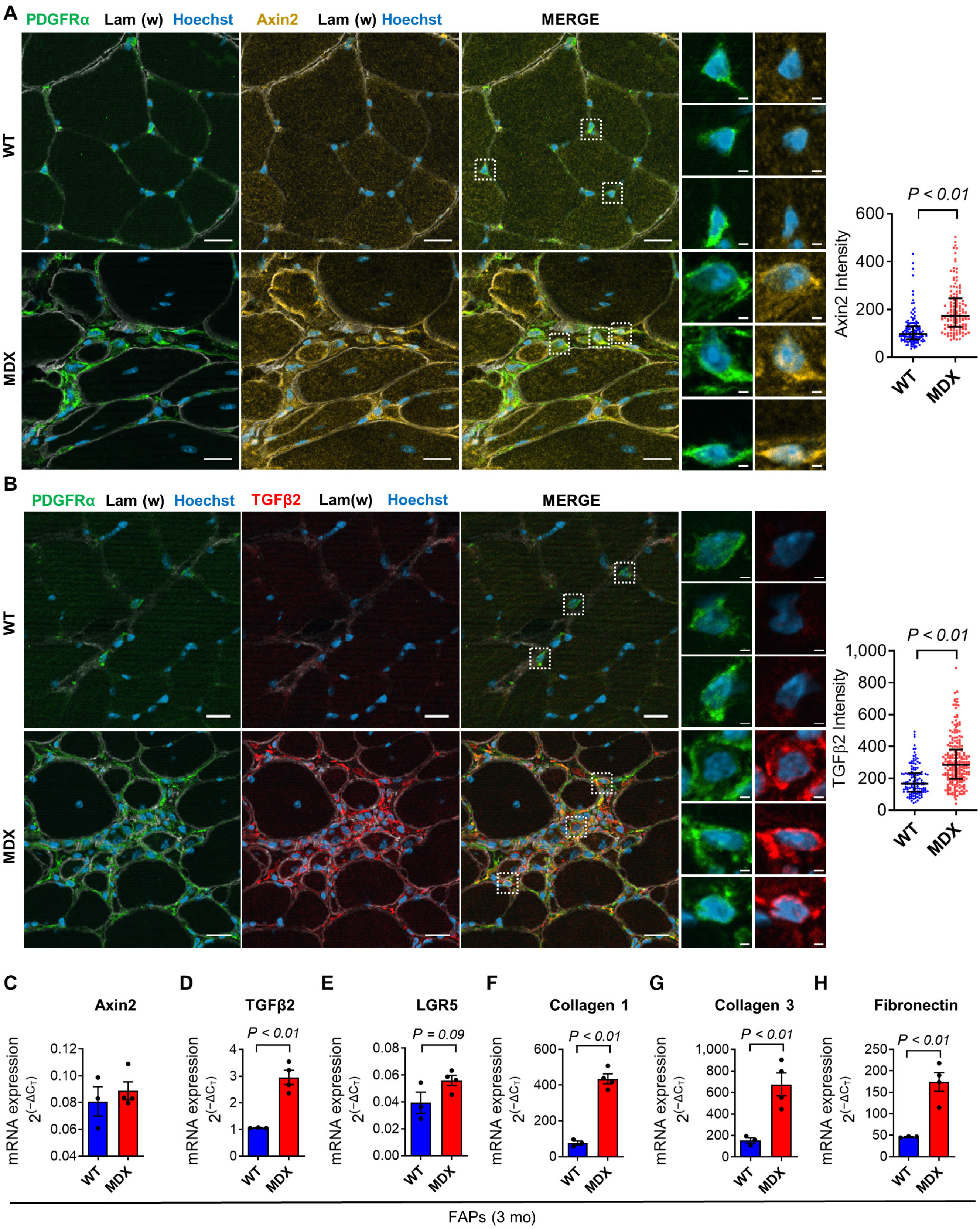

Figure EV1.

© 2023 The Authors

Figure EV2. C1 and C4 complement levels are increased in dystrophic muscles.

Representative immunofluorescence of ~1-year-old WT and MDX gastrocnemius stained with anti-C1q (red), anti-C4 (yellow) antibodies, and Hoechst (blue). High-magnification images represent different zoomed areas of the muscle. Note the expression of both C4 and C1q in the same muscle areas. Scale bar: 100 μm (low-magnification images), scale bar: 20 μm (high-magnification images).

Source data are available online for this figure.

© 2023 The Authors

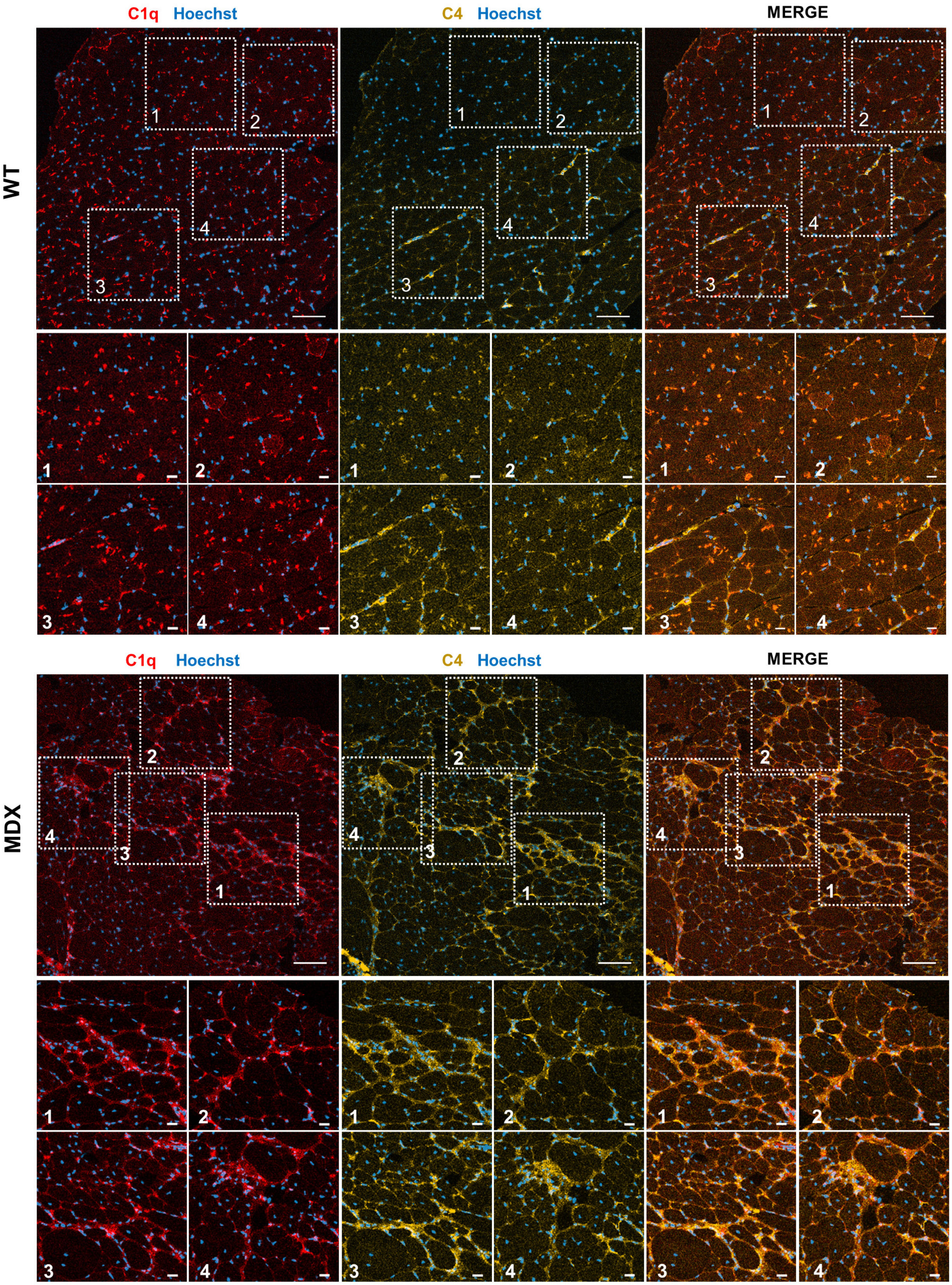

Figure EV2.

 © 2023 The Authors

Figure EV3. Complement expression in different subsets of macrophages in dystrophic muscles.

A Representative gating and sorting strategy used to FACS isolate different macrophage populations from *MDX* mice. Macrophages were gated as $CD45^{+ve}F4/80^{+ve}$ cells. Within the macrophage population, $CD206^{-ve}$, $CD206^{Low}$, and $CD206^{High}$ subpopulations were isolated.

B, C Number of $CD206^{-ve}$, $CD206^{Low}$, and $CD206^{High}$ macrophages from *MDX* mice FACS-isolated as in (A). Data are expressed as the percentage of $F4/80^{+ve}$ cells (B) and the number of cells per mg of tissue (C). *N* (biological samples) = 4.

D–H *CD206* (D), *CD163* (E), *C1qa* (F), *C1qb* (G), and *C1qc* (H) mRNA expression in $CD206^{-ve}$, $CD206^{Low}$, and $CD206^{High}$ macrophages from *MDX* mice FACS isolated as in (A). *N* (biological samples) = 4.

I, J *IL-10/TNFα* (I) and *IL-10/TGFβ1* (J) mRNA expression in $CD206^{-ve}$, $CD206^{Low}$, and $CD206^{High}$ macrophages from *MDX* mice FACS isolated as in (A). *N* (biological samples) = 4.

Data information: Data are presented as mean ± SEM. Statistical differences between two groups were calculated by unpaired two-tailed Student's *t*-test. *P*-values are as indicated.
Source data are available online for this figure.

© 2023 The Authors

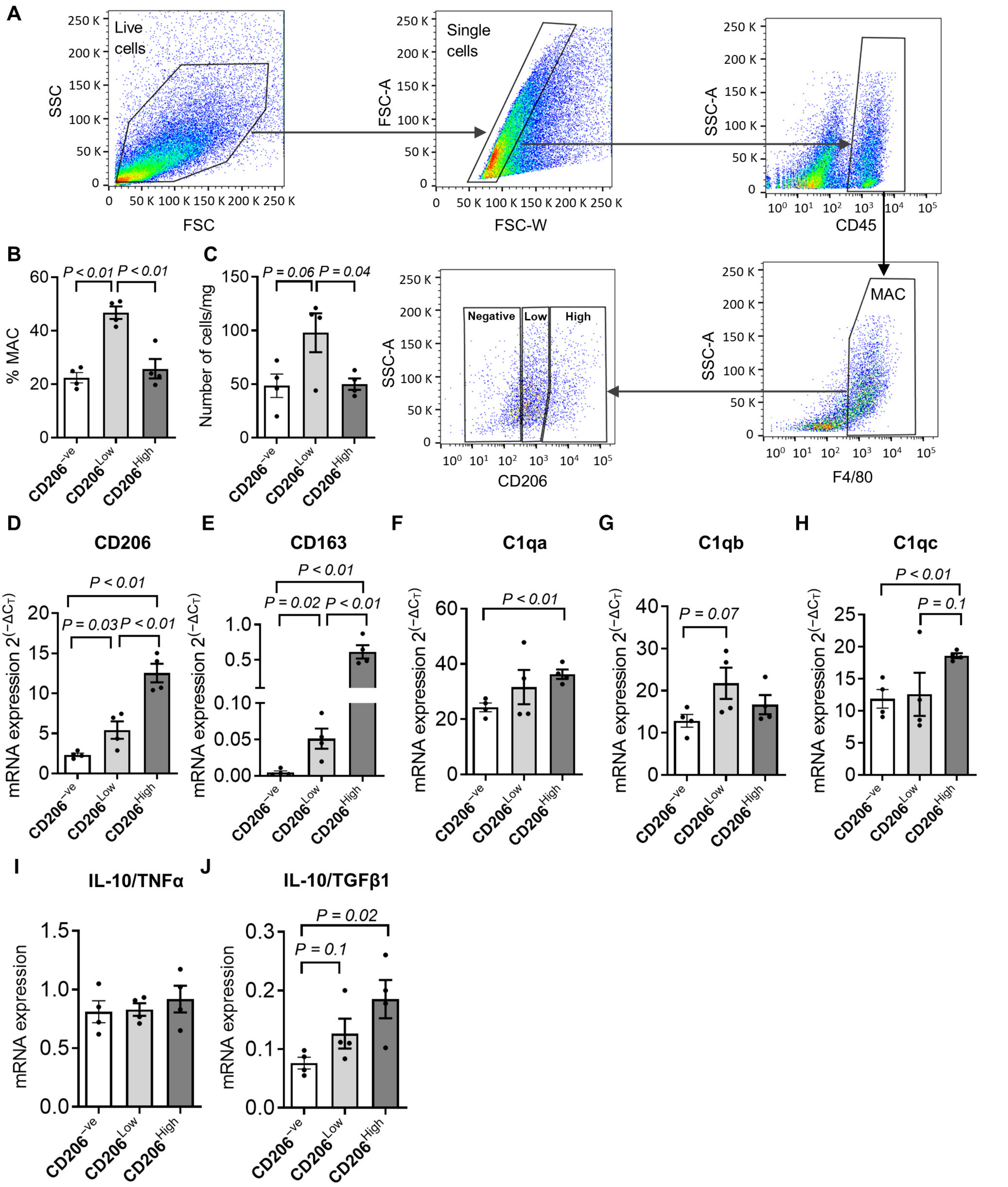

Figure EV3.

 © 2023 The Authors

Figure EV4. The fib-MDX skeletal muscle: macrophages/FAPs interplay.

A Representative immunofluorescence of *gastrocnemius* of ~1-year-old *WT* 4 days after cardiotoxin injury (*WT* INJ), *MDX*, and fib-*MDX* mice stained with anti-PDGFrα (green), anti-F4/80 (orange) antibodies, and Hoechst (blue). White dollar symbols indicate macrophages (i.e., F4/80^{+ve} cells), red asterisks indicate FAPs (i.e., PDGFRα^{+ve} cells), and dotted lines indicate examples of distance measured between macrophages and FAPs. Scale bar: 10 μm.

B Distance (μm) between macrophages (MAC) and FAPs measured in the *gastrocnemius* of ~1-year-old *WT* 4 days after cardiotoxin injury (*WT* INJ), *MDX*, and fib-*MDX* mice stained as in (A). *N* (biological samples) = 3.

C Example of a contact between a macrophage and a FAP in muscles represented in (A). Scale bar: 10 μm.

D Percentage of contacts (i.e., a distance of 3 μm or less) between macrophages and FAPs calculated in the same muscles as in (A). *N* (biological samples) = 3.

E, F *TNFα/TGFβ1* (E) and *TNFα/IL-10* (F) mRNA expression in F4/80^{+ve} macrophages FACS isolated from ~3-months-old *MDX*, ~1-year-old *MDX*, and ~1-year-old fib-*MDX* hindlimb muscles. *N* (biological replicates) = 4.

G, H *CD206* (G) and *CD163* (H) mRNA expression in macrophages FACS isolated as in (E and F). *N* (biological replicates) = 4.

I Representative immunofluorescence (left) and quantification (right) of the TGFβ2^{+ve} FAPs (i.e., PDGFRα^{+ve} and TGFβ2^{+ve} cells) expressed as percentage of the total number of FAPs (i.e., PDGFRα^{+ve} cells) in the *gastrocnemius* of ~1-year-old *MDX* and fib-*MDX* mice stained with anti-PDGFrα (green), anti-TGFB2 (yellow), anti-Laminin2α (gray) antibodies, and Hoechst (blue). *N* (biological samples) = 3. Scale bar: 20 μm (low-magnification images), scale bar: 5 μm (high-magnification images).

Data information: In (B) data are presented as median with interquartile range. Each dot on the graph represents a distance measurement (307 for *WT* INJ, 261 for *MDX*, and 303 for fib-*MDX*). Statistical differences were calculated by the Kruskal–Wallis test. Dunn's multiple–comparison test was used as a *post hoc* test. In (D–I), data are presented as mean ± SEM. In (I) each graph dot represents the percentage of TGFβ2^{+ve} cells calculated in 344 to 412 (*MDX*) and 315 to 617 (fib-*MDX*) FAPs randomly selected in different muscles' interstitial regions for each biological sample. Statistical differences between two groups were calculated in (E–I) by unpaired two-tailed Student's *t*-test and between three groups in (D) by one-way ANOVA test using Tukey's multiple-comparison test as a *post hoc* test. *P*-values are as indicated.
Source data are available online for this figure.

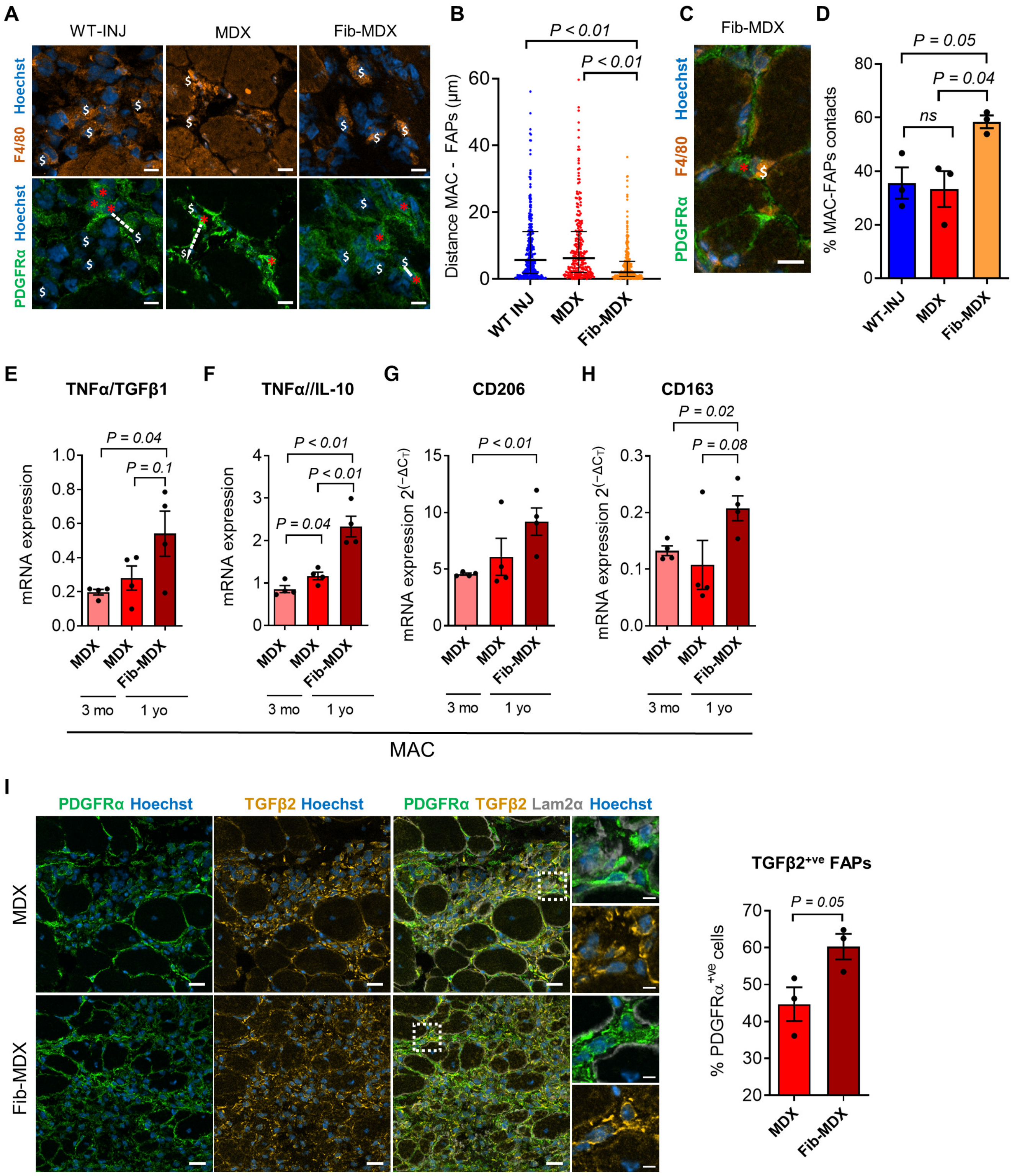

Figure EV4.

 © 2023 The Authors

Figure EV5. *In vivo* inhibition of C1r/s in *WT* muscles after acute injury: FAPs, macrophages, and tissue analysis.

A Scheme of the C1r/s inhibition experiment in *WT* muscles after acute cardiotoxin injury. Note that the length of the treatment with the C1r/s inhibitor corresponds to the time between the last needle injury and the moment of muscle dissection in the fib-*MDX* experiments (Fig 6A).

B–F *C1r* (B), *C1s* (C), *C1qa* (D), *C1qb* (E), and *C1qc* (F), mRNA expression in FACS-isolated FAPs (C1r and C1s), and macrophages (C1qa, C1qb, and C1qc) from fib-*MDX* and *WT* mice processed as in (A). *N* (biological samples) = 3. Fib-*MDX* samples are the same as used in the analysis shown in Fig 5E–I.

G–K *Axin2* (G), *LGR5* (H), *TGFβ2* (I), *collagen 1a1* (J), and *collagen 3a1* (K) mRNA expression in FACS-isolated FAPs from *WT* mice processed as in (A). *N* (biological samples) = 3.

L Representative immunofluorescence of *gastrocnemius* muscles from *WT* mice processed as in (A). Muscles were stained with anti-collagen 1 (green), anti-laminin 2α (red) antibodies, and Hoechst (blue). Scale bar: 50 μm.

M Fibrotic area quantification of the same muscles as in (L). *N* (biological samples) = 3.

N Cross-sectional area (CSA) quantification of the muscle fibers of the same muscles as in (L). *N* (biological samples) = 3.

O Collagen 1 pixel intensity quantification in the interstitial space between myofibers of the same muscles as in (L). *N* (biological samples) = 3.

Data information: Data are presented as mean ± SEM. In (M and O) each graph dot represents the average value of 62 to 342 (M) and 31 to 45 (O) measurements on different muscle regions for each biological sample. In (N) each graph dot represents the median value of 351 to 415 cross-sectional area measurements on different muscle regions for each biological sample. The statistical differences were calculated in (B–F) by unpaired two-tailed Student's *t*-test and in (G–K) and (M–O) by paired two-tailed Student's *t*-test. *P*-values are as indicated.
Source data are available online for this figure.

© 2023 The Authors

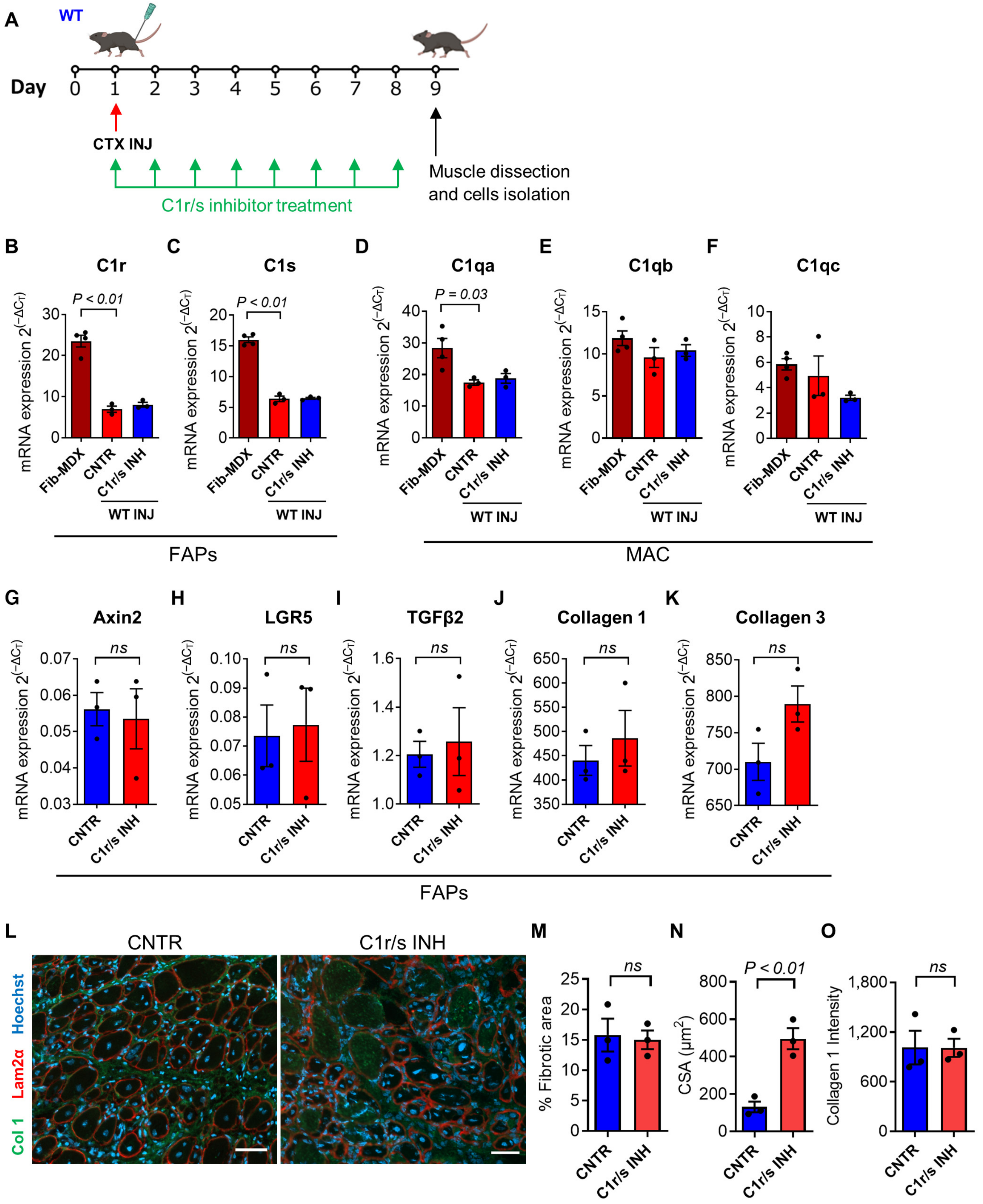

Figure EV5.

 © 2023 The Authors
