# Supplementary figures and images for: Combinatorial activation of the WNT‐dependent fibrogenic program by distinct complement subunits in dystrophic muscle
Source: EMBO Mol Med. 2023 Nov 6;15(12):e17405. doi: 10.15252/emmm.202317405 (PMC10701616; doi:10.15252/emmm.202317405)

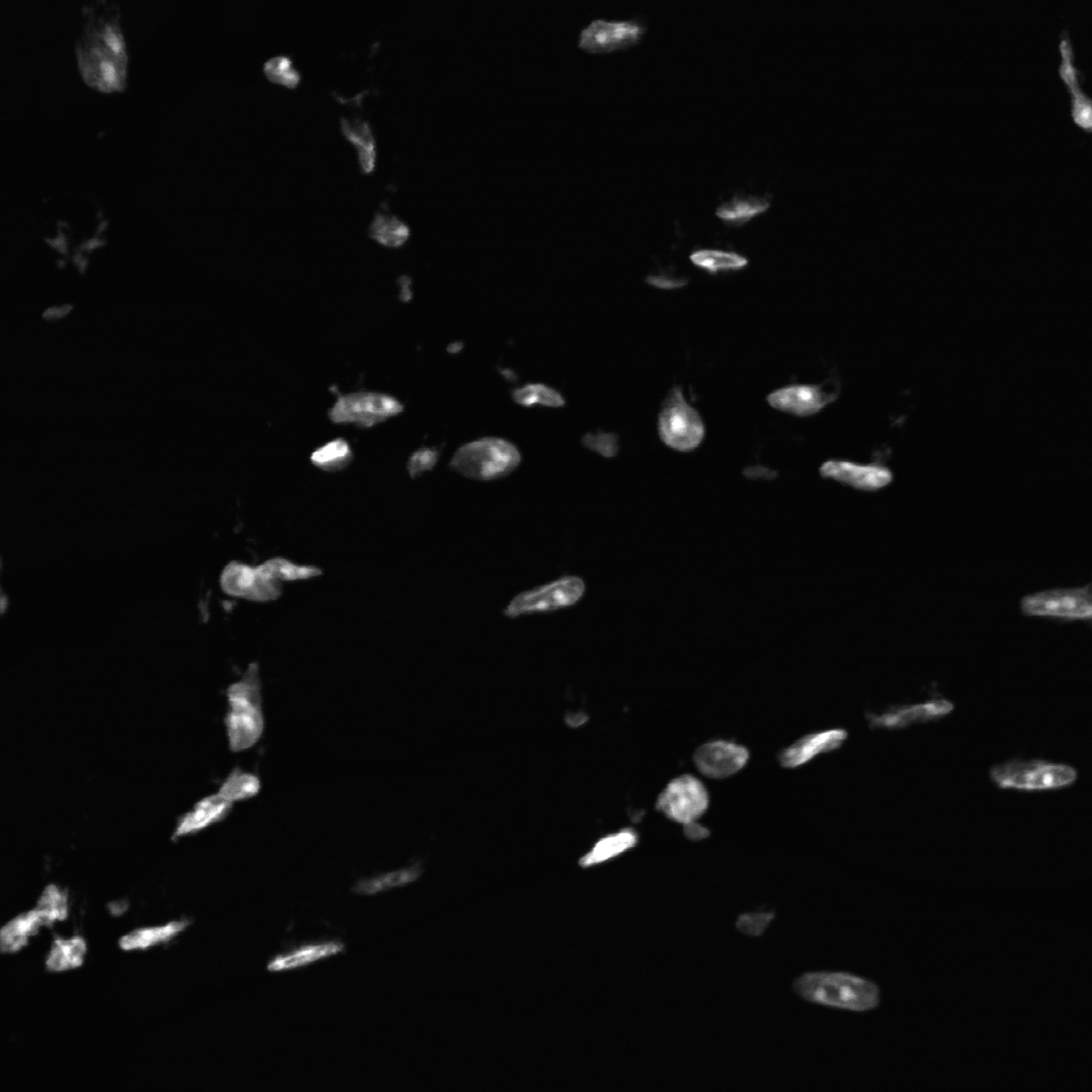

Supplement: Supplementary file 3 — Source Data for Expanded View [file EMMM-15-e17405-s003.zip › Figures_EV/Figure_EV_1/Figure_EV_1_A_B/Fig_EV_1_A/MDX/MDX_Axin2.tif]

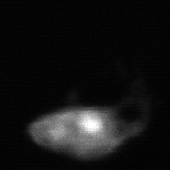

Supplement: Supplementary file 3 — Source Data for Expanded View [file EMMM-15-e17405-s003.zip › Figures_EV/Figure_EV_1/Figure_EV_1_A_B/Fig_EV_1_A/MDX/MDX_Axin2_cell1.tif]

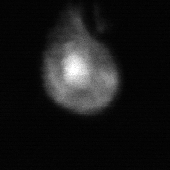

Supplement: Supplementary file 3 — Source Data for Expanded View [file EMMM-15-e17405-s003.zip › Figures_EV/Figure_EV_1/Figure_EV_1_A_B/Fig_EV_1_A/MDX/MDX_Axin2_cell2.tif]

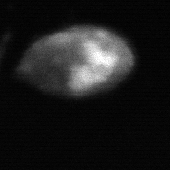

Supplement: Supplementary file 3 — Source Data for Expanded View [file EMMM-15-e17405-s003.zip › Figures_EV/Figure_EV_1/Figure_EV_1_A_B/Fig_EV_1_A/MDX/MDX_Axin2_cell3.tif]

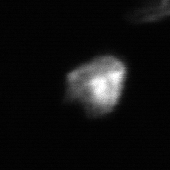

Supplement: Supplementary file 3 — Source Data for Expanded View [file EMMM-15-e17405-s003.zip › Figures_EV/Figure_EV_1/Figure_EV_1_A_B/Fig_EV_1_A/WT/WT_Axin2_cell1.tif]

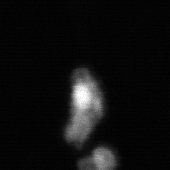

Supplement: Supplementary file 3 — Source Data for Expanded View [file EMMM-15-e17405-s003.zip › Figures_EV/Figure_EV_1/Figure_EV_1_A_B/Fig_EV_1_A/WT/WT_Axin2_cell2.tif]

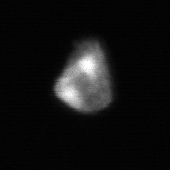

Supplement: Supplementary file 3 — Source Data for Expanded View [file EMMM-15-e17405-s003.zip › Figures_EV/Figure_EV_1/Figure_EV_1_A_B/Fig_EV_1_A/WT/WT_Axin2_cell3.tif]

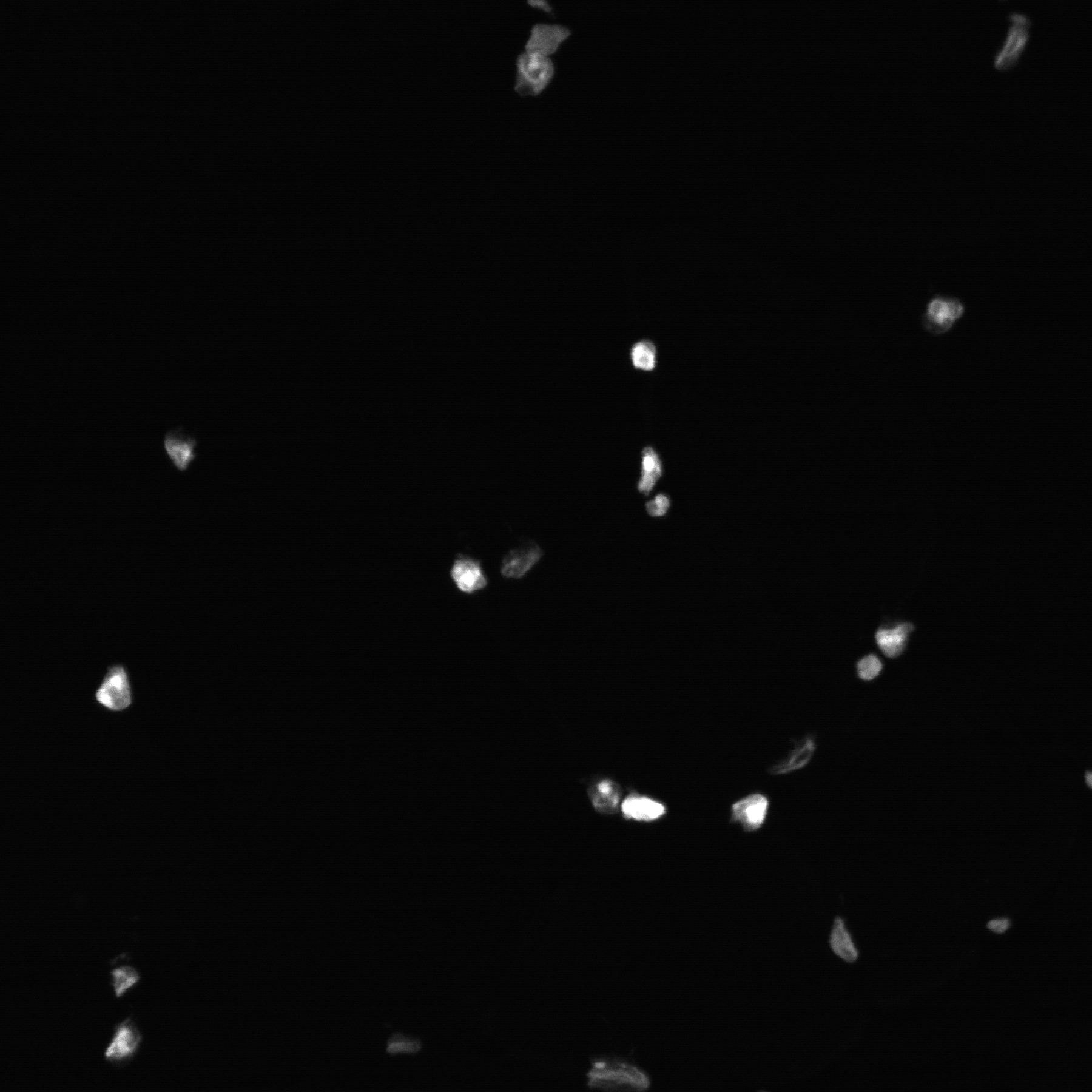

Supplement: Supplementary file 3 — Source Data for Expanded View [file EMMM-15-e17405-s003.zip › Figures_EV/Figure_EV_1/Figure_EV_1_A_B/Fig_EV_1_A/WT/WT_Axin2_roi.tif]

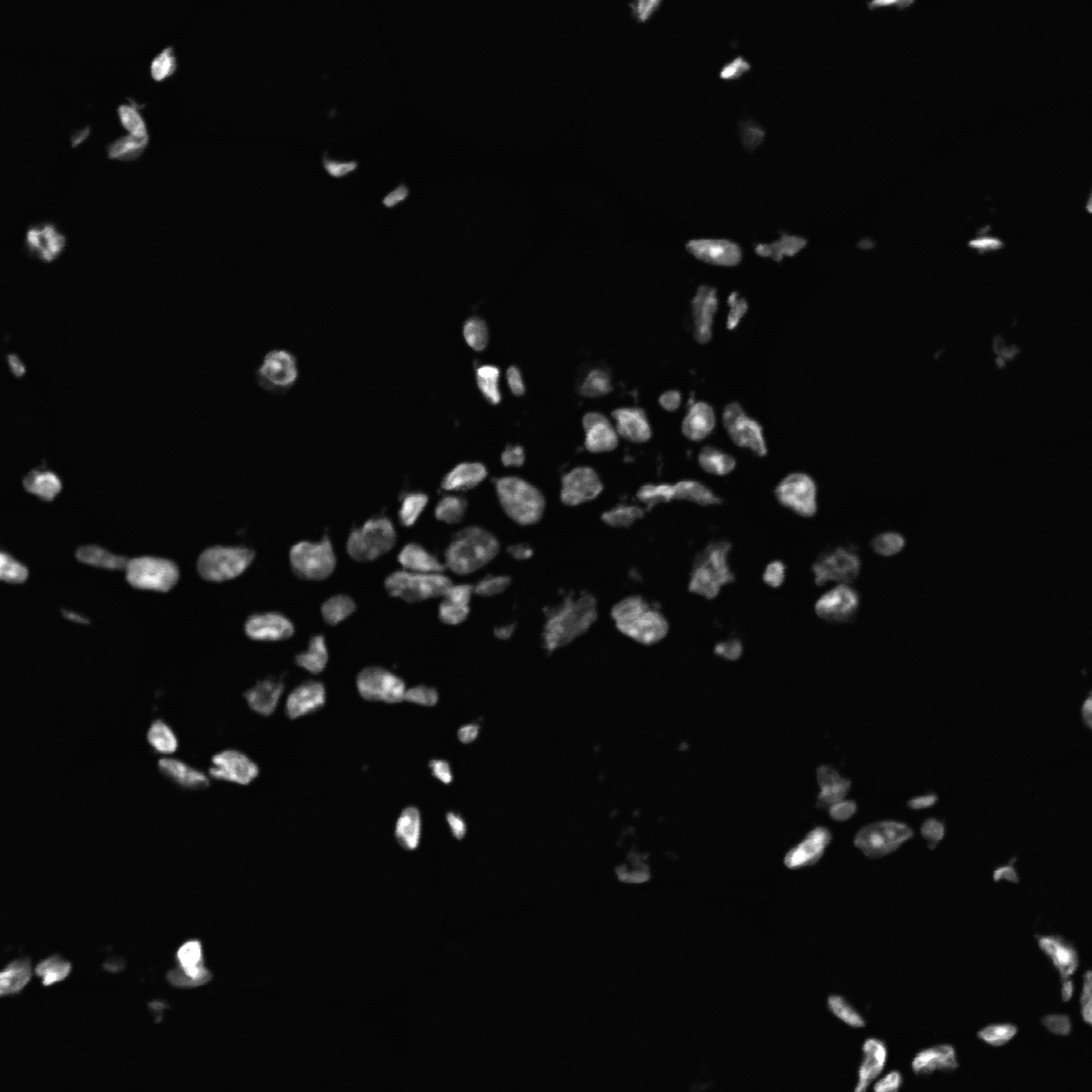

Supplement: Supplementary file 3 — Source Data for Expanded View [file EMMM-15-e17405-s003.zip › Figures_EV/Figure_EV_1/Figure_EV_1_A_B/Fig_EV_1_B/MDX/MDX_TGFB2.tif]

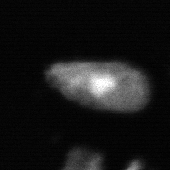

Supplement: Supplementary file 3 — Source Data for Expanded View [file EMMM-15-e17405-s003.zip › Figures_EV/Figure_EV_1/Figure_EV_1_A_B/Fig_EV_1_B/MDX/MDX_TGFB2_cell1.tif]

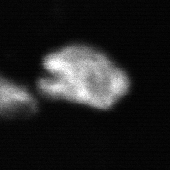

Supplement: Supplementary file 3 — Source Data for Expanded View [file EMMM-15-e17405-s003.zip › Figures_EV/Figure_EV_1/Figure_EV_1_A_B/Fig_EV_1_B/MDX/MDX_TGFB2_cell2.tif]

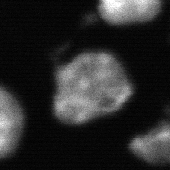

Supplement: Supplementary file 3 — Source Data for Expanded View [file EMMM-15-e17405-s003.zip › Figures_EV/Figure_EV_1/Figure_EV_1_A_B/Fig_EV_1_B/MDX/MDX_TGFB2_cell3.tif]

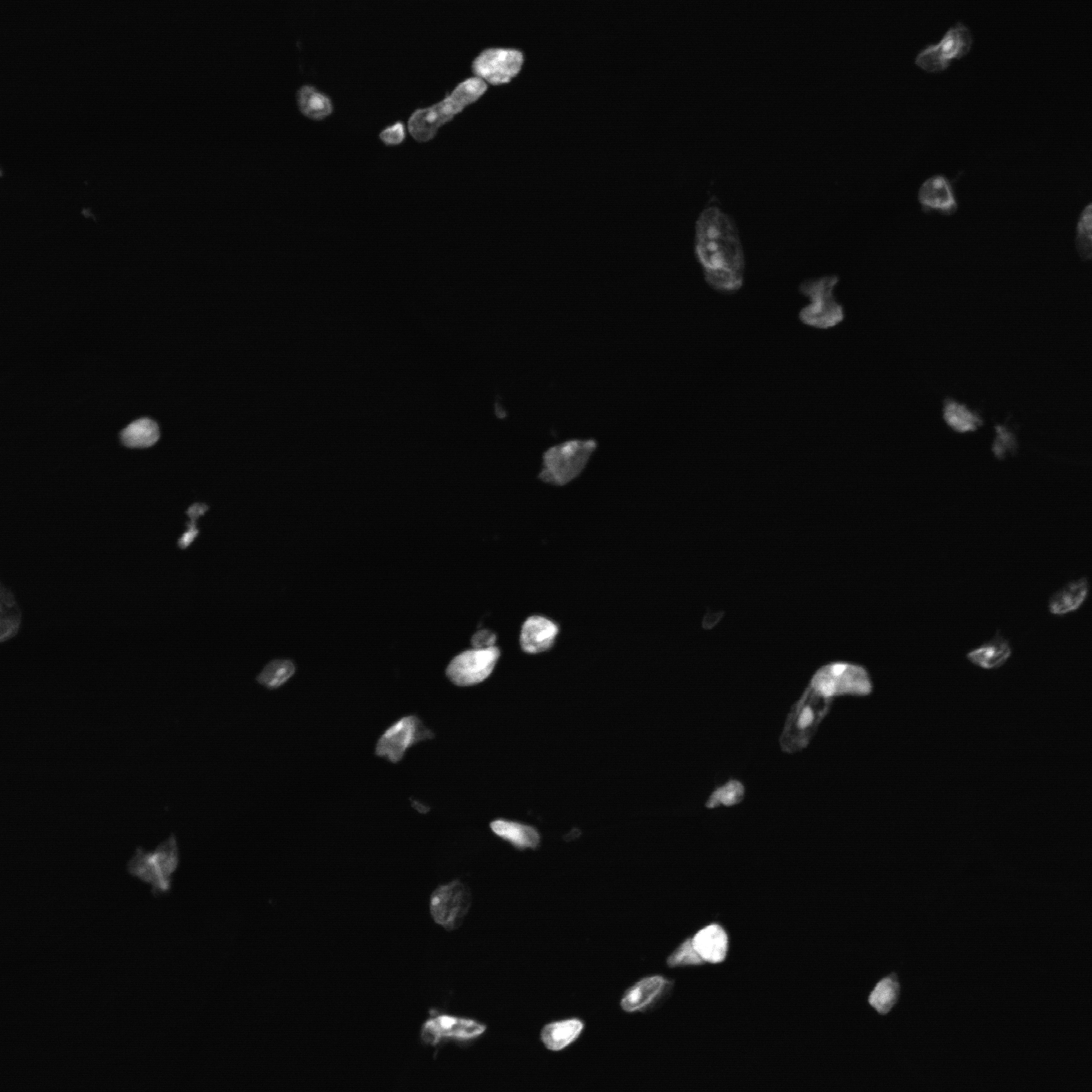

Supplement: Supplementary file 3 — Source Data for Expanded View [file EMMM-15-e17405-s003.zip › Figures_EV/Figure_EV_1/Figure_EV_1_A_B/Fig_EV_1_B/WT/WT_TGFB2.tif]

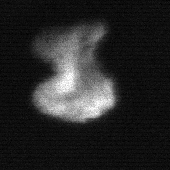

Supplement: Supplementary file 3 — Source Data for Expanded View [file EMMM-15-e17405-s003.zip › Figures_EV/Figure_EV_1/Figure_EV_1_A_B/Fig_EV_1_B/WT/WT_TGFB2_cell1.tif]

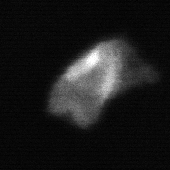

Supplement: Supplementary file 3 — Source Data for Expanded View [file EMMM-15-e17405-s003.zip › Figures_EV/Figure_EV_1/Figure_EV_1_A_B/Fig_EV_1_B/WT/WT_TGFB2_cell2.tif]

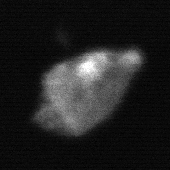

Supplement: Supplementary file 3 — Source Data for Expanded View [file EMMM-15-e17405-s003.zip › Figures_EV/Figure_EV_1/Figure_EV_1_A_B/Fig_EV_1_B/WT/WT_TGFB2_cell3.tif]

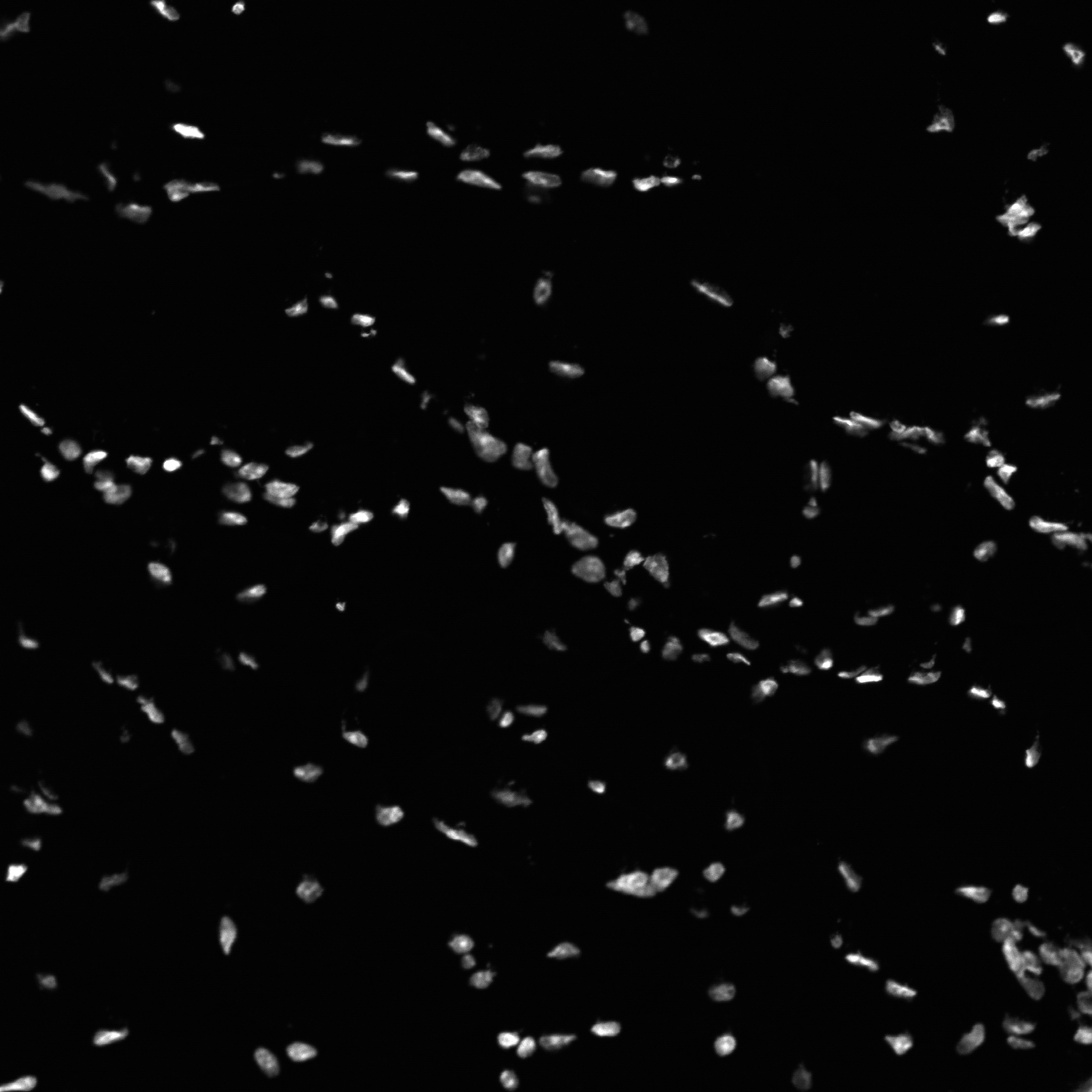

Supplement: Supplementary file 3 — Source Data for Expanded View [file EMMM-15-e17405-s003.zip › Figures_EV/Figure_EV_2/Figure_EV_2_A/MDX/MDX_high_magnification_1.tif]

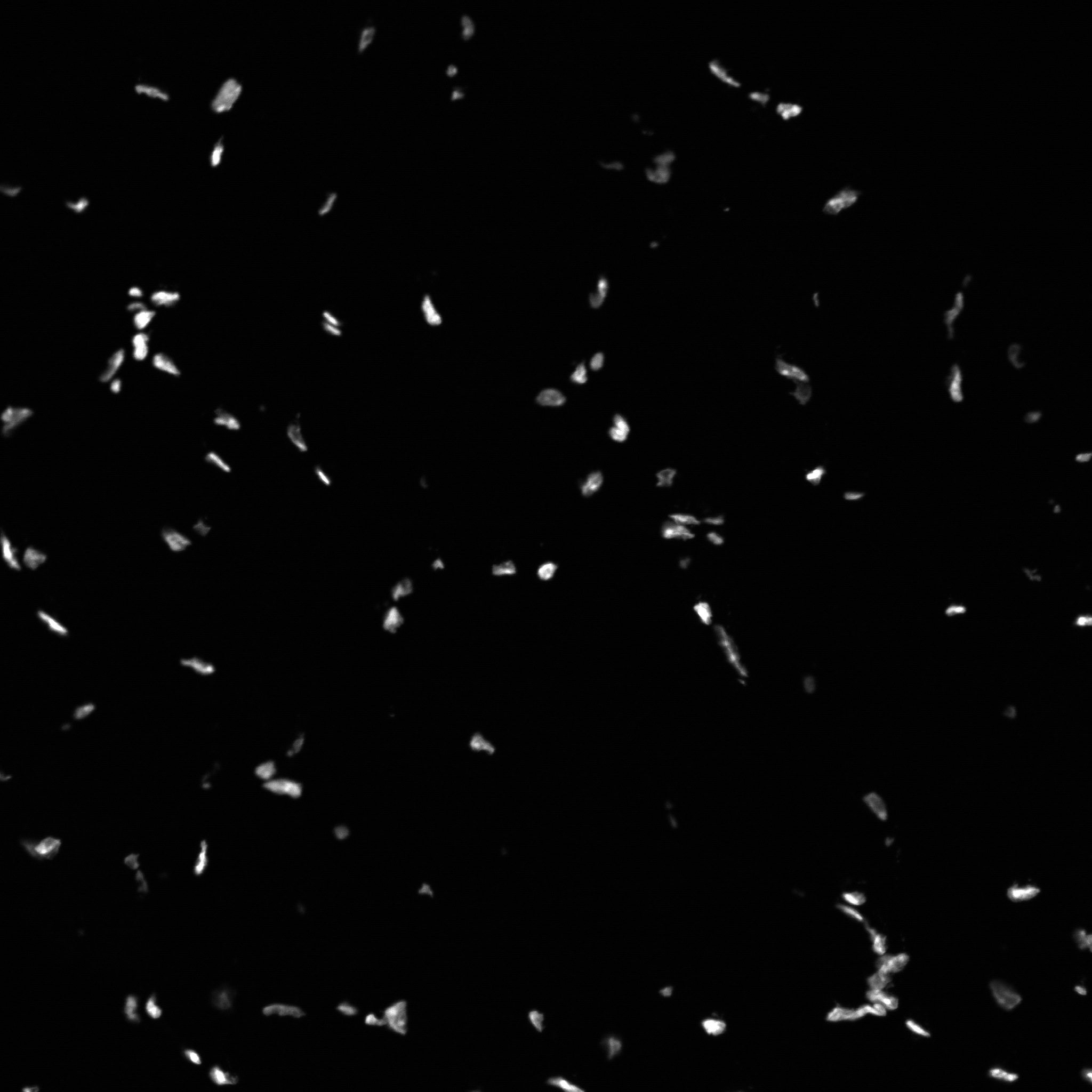

Supplement: Supplementary file 3 — Source Data for Expanded View [file EMMM-15-e17405-s003.zip › Figures_EV/Figure_EV_2/Figure_EV_2_A/MDX/MDX_high_magnification_2.tif]

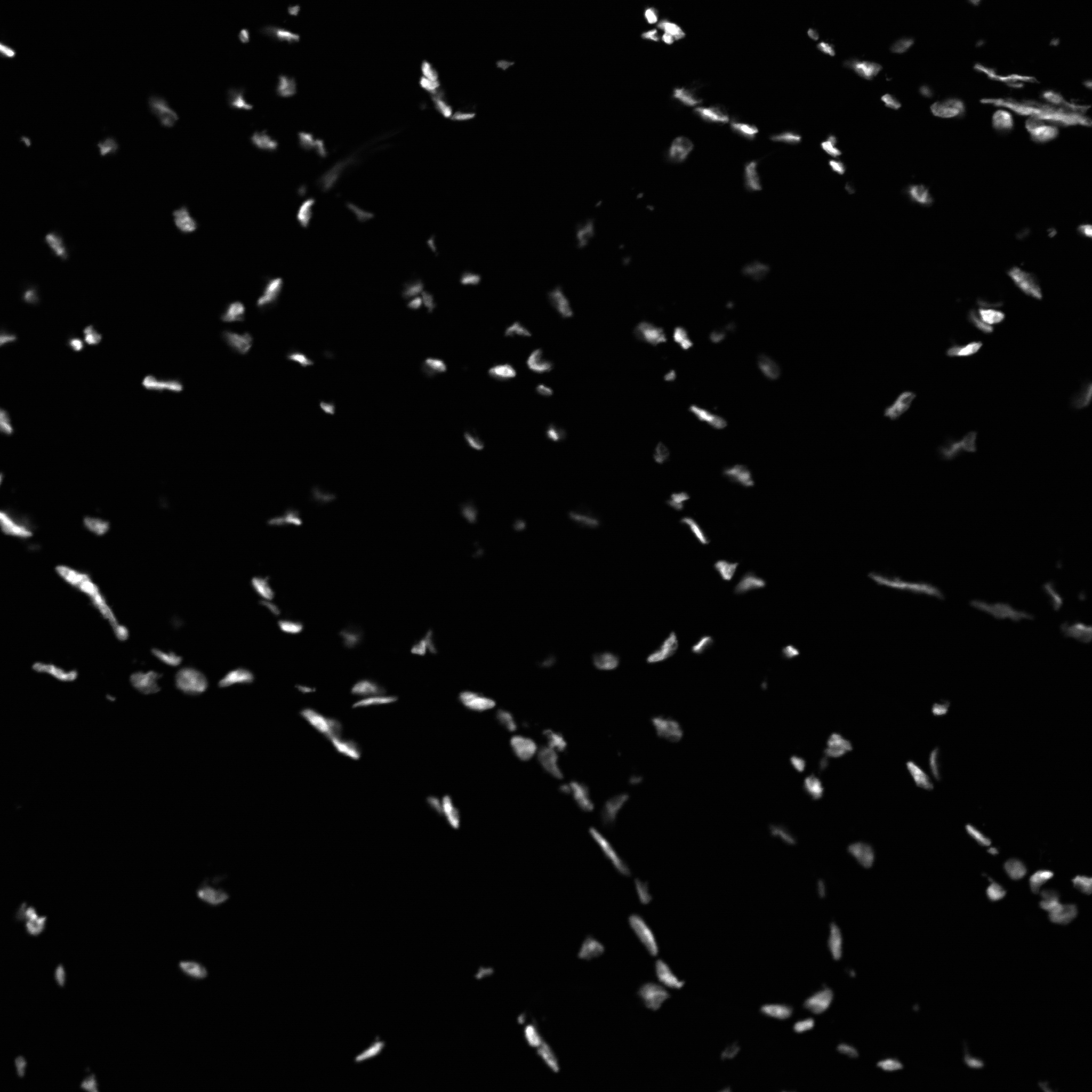

Supplement: Supplementary file 3 — Source Data for Expanded View [file EMMM-15-e17405-s003.zip › Figures_EV/Figure_EV_2/Figure_EV_2_A/MDX/MDX_high_magnification_3.tif]

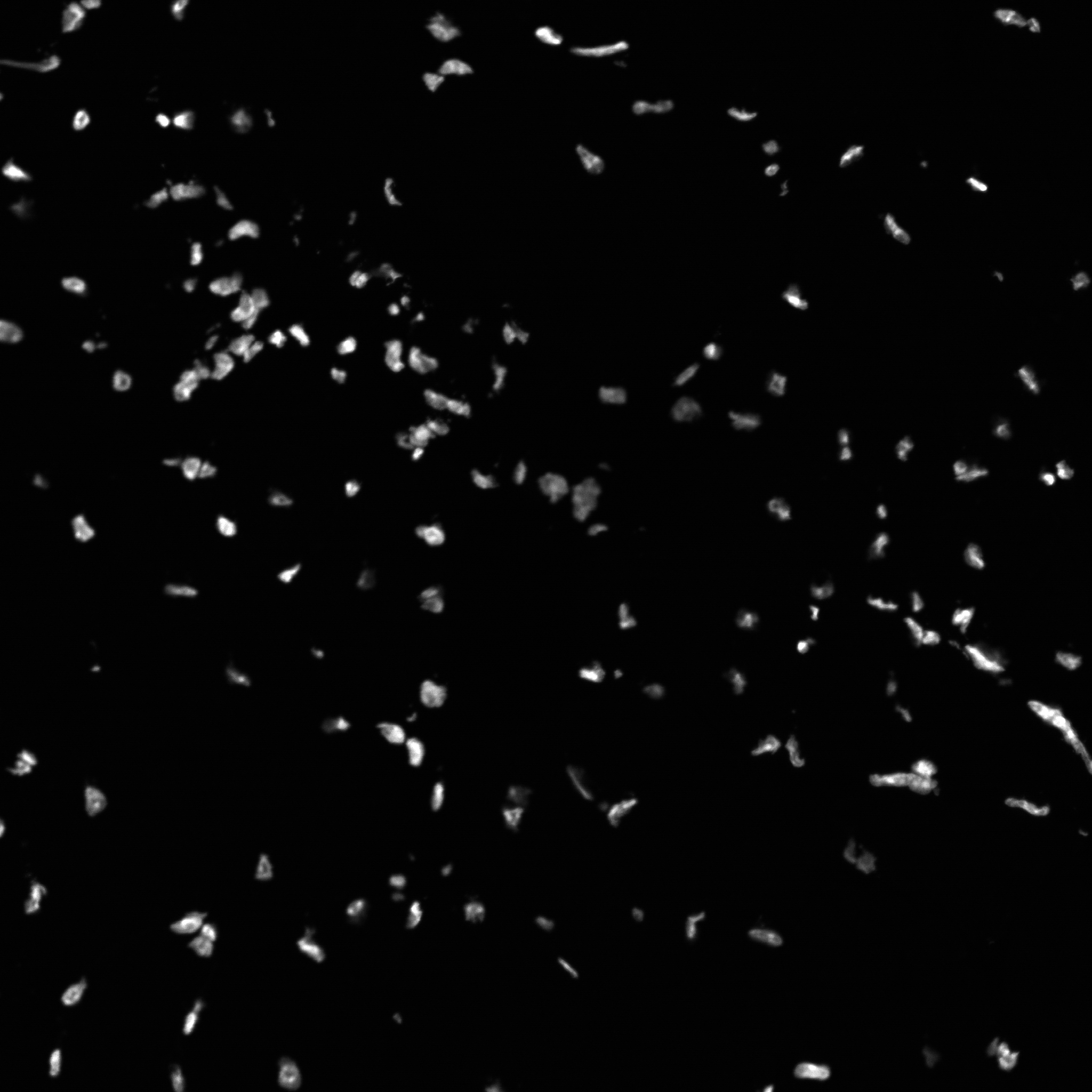

Supplement: Supplementary file 3 — Source Data for Expanded View [file EMMM-15-e17405-s003.zip › Figures_EV/Figure_EV_2/Figure_EV_2_A/MDX/MDX_high_magnification_4.tif]

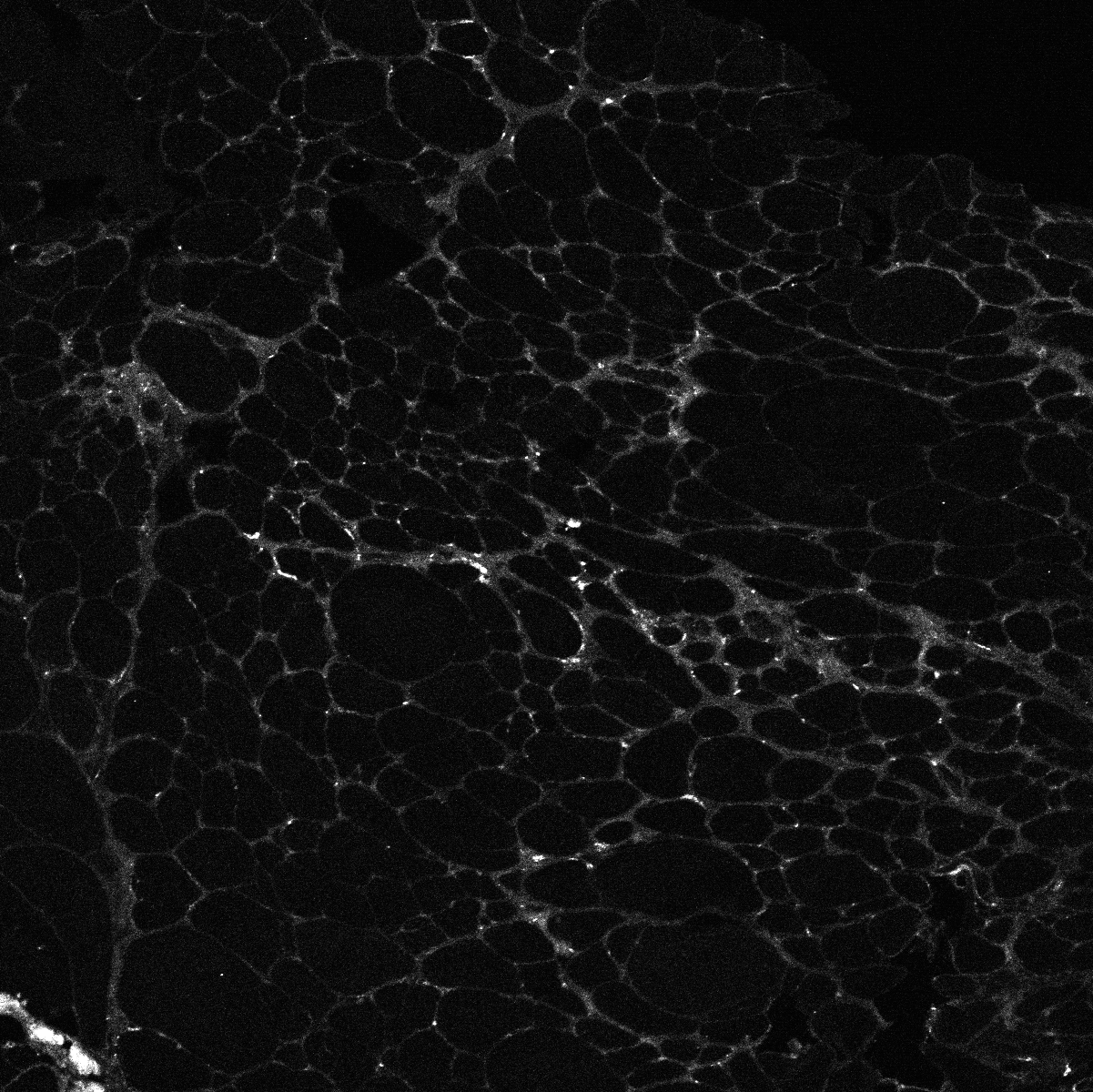

Supplement: Supplementary file 3 — Source Data for Expanded View [file EMMM-15-e17405-s003.zip › Figures_EV/Figure_EV_2/Figure_EV_2_A/MDX/MDX_low_magnification.tif]

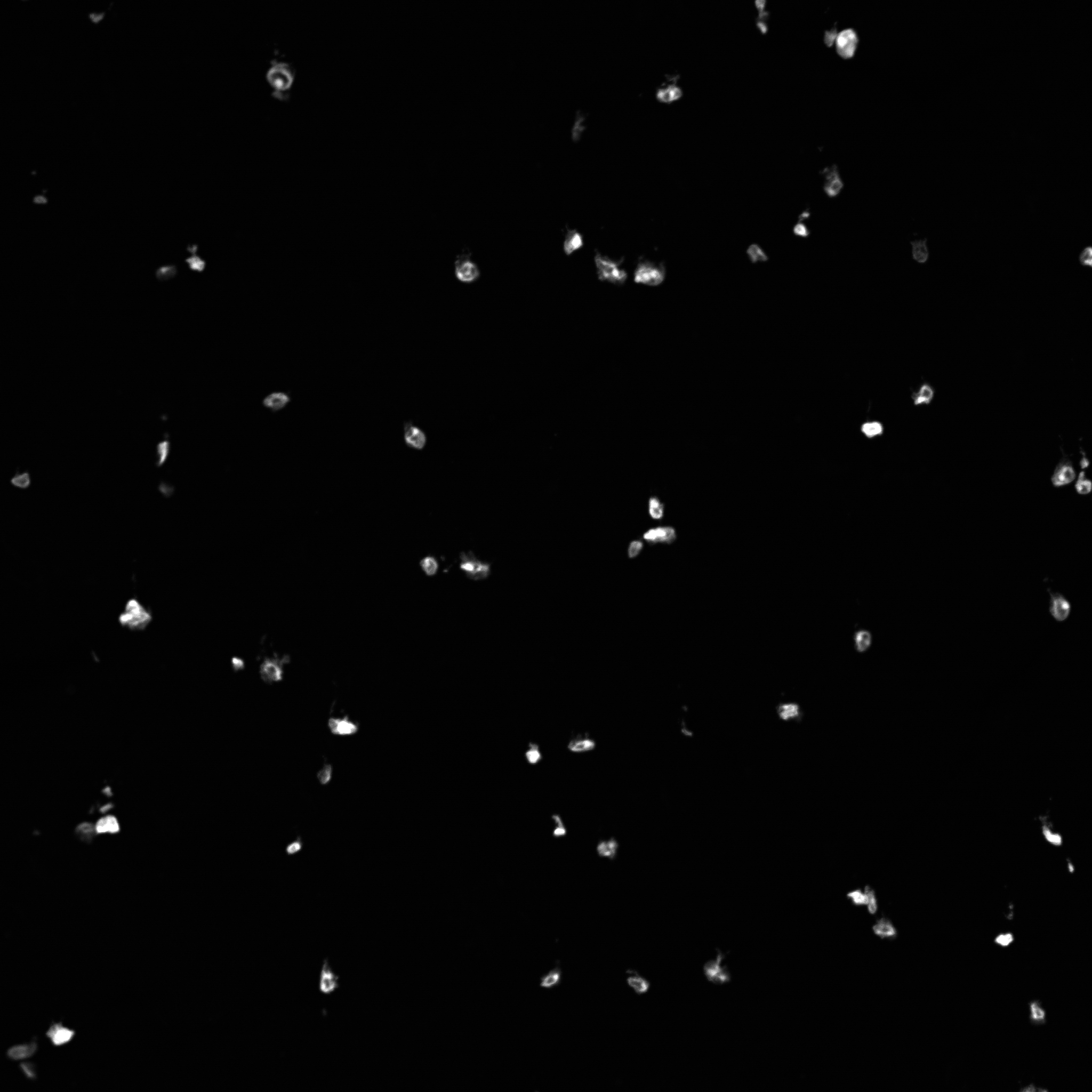

Supplement: Supplementary file 3 — Source Data for Expanded View [file EMMM-15-e17405-s003.zip › Figures_EV/Figure_EV_2/Figure_EV_2_A/WT/WT_high_magnification_1.tif]

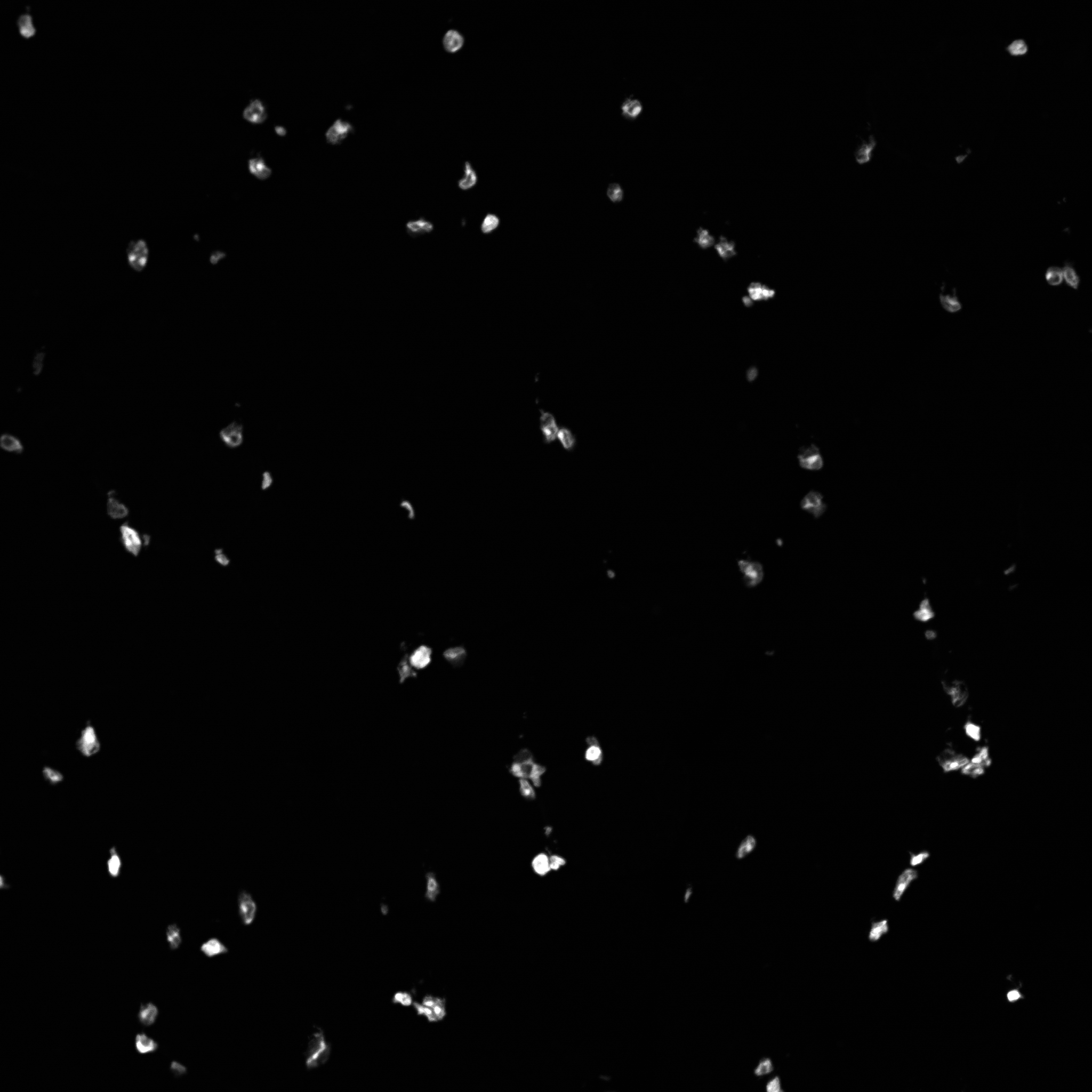

Supplement: Supplementary file 3 — Source Data for Expanded View [file EMMM-15-e17405-s003.zip › Figures_EV/Figure_EV_2/Figure_EV_2_A/WT/WT_high_magnification_2.tif]

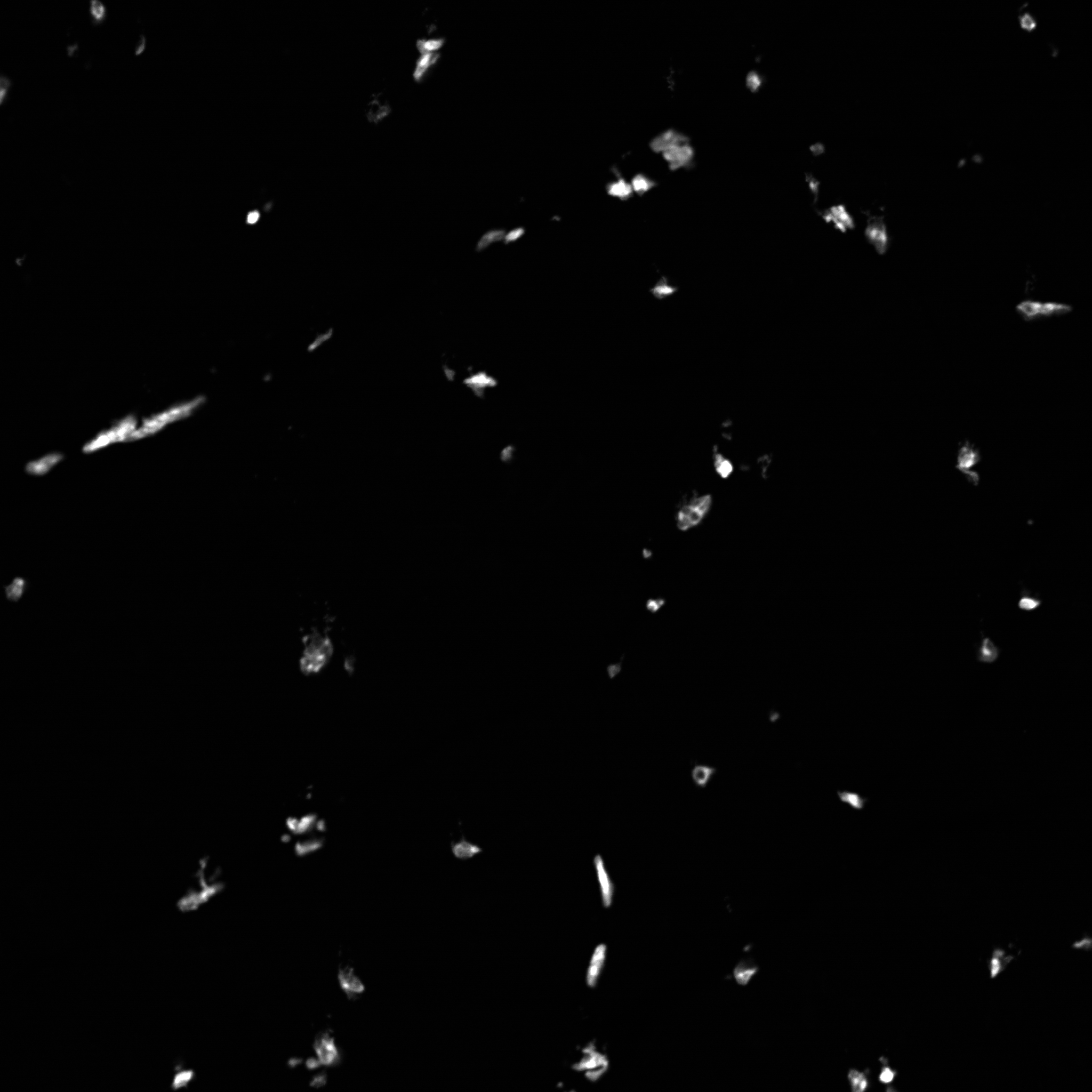

Supplement: Supplementary file 3 — Source Data for Expanded View [file EMMM-15-e17405-s003.zip › Figures_EV/Figure_EV_2/Figure_EV_2_A/WT/WT_high_magnification_3.tif]

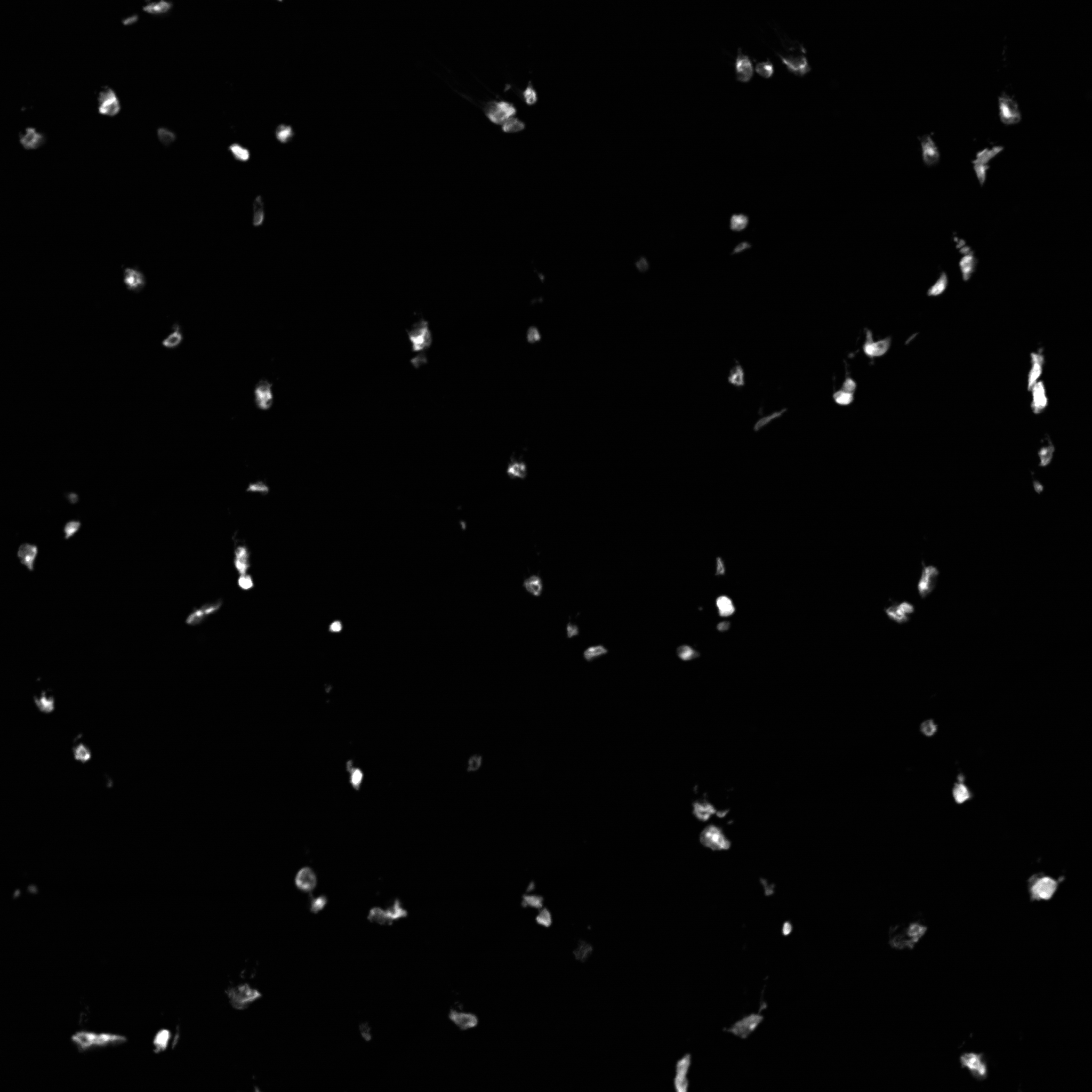

Supplement: Supplementary file 3 — Source Data for Expanded View [file EMMM-15-e17405-s003.zip › Figures_EV/Figure_EV_2/Figure_EV_2_A/WT/WT_high_magnification_4.tif]

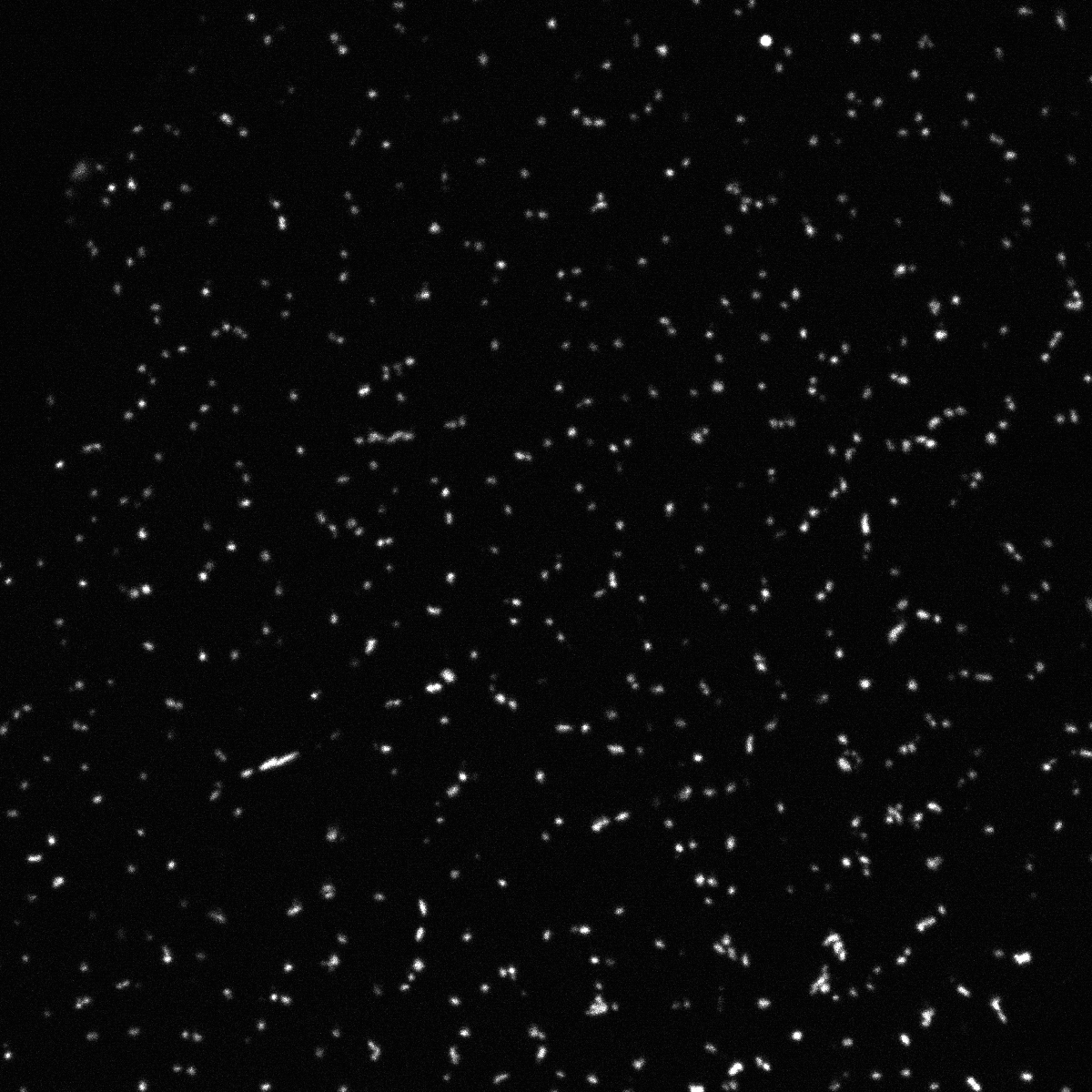

Supplement: Supplementary file 3 — Source Data for Expanded View [file EMMM-15-e17405-s003.zip › Figures_EV/Figure_EV_2/Figure_EV_2_A/WT/WT_low_magnification.tif]

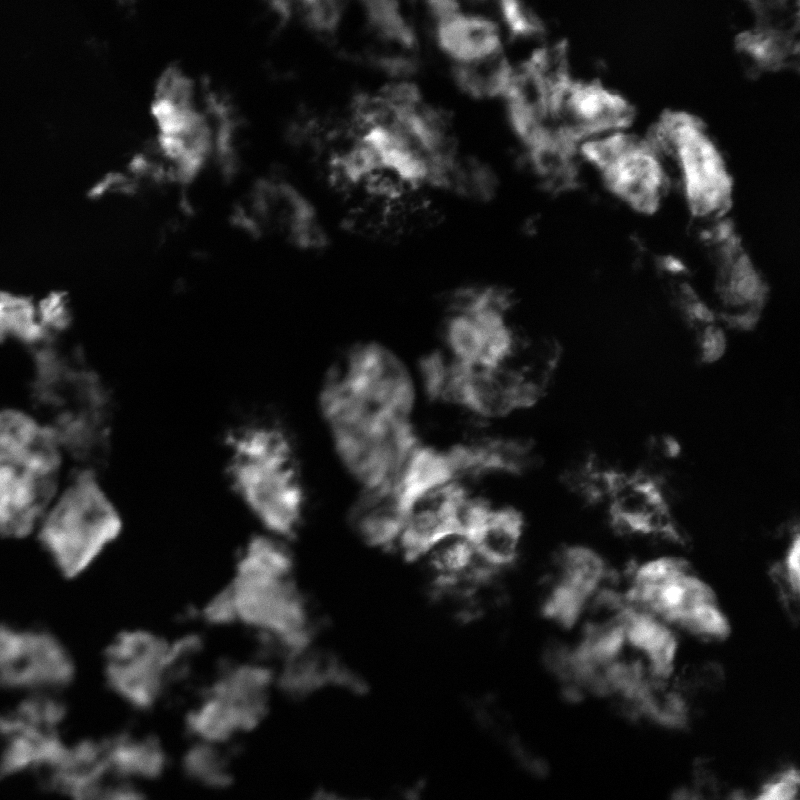

Supplement: Supplementary file 3 — Source Data for Expanded View [file EMMM-15-e17405-s003.zip › Figures_EV/Figure_EV_4/Figure_EV_4_A_B_C_D/Figure_EV_4_A/Fib-MDX/Fib_MDX.tif]

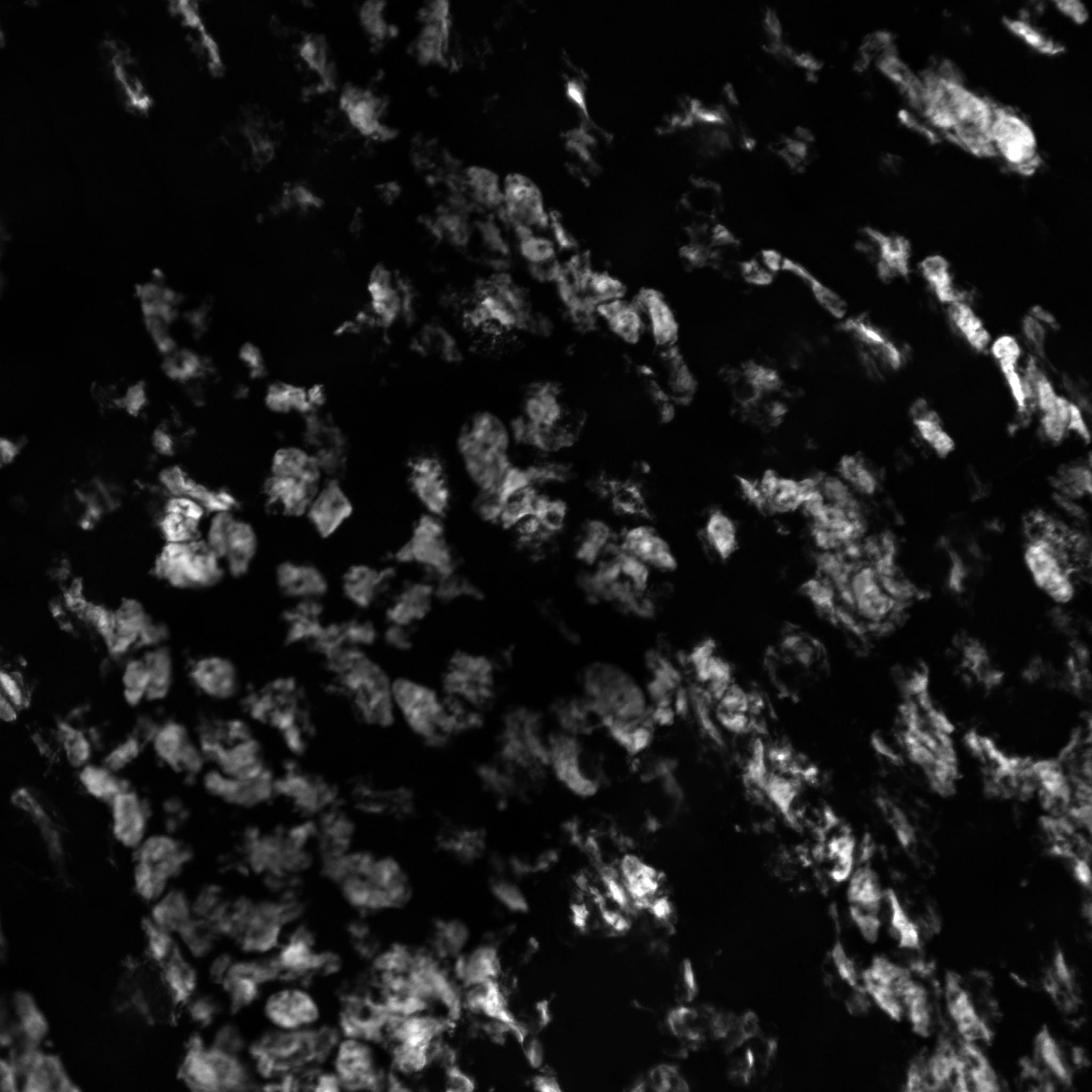

Supplement: Supplementary file 3 — Source Data for Expanded View [file EMMM-15-e17405-s003.zip › Figures_EV/Figure_EV_4/Figure_EV_4_A_B_C_D/Figure_EV_4_A/Fib-MDX/Fib_MDX_uncropped.tif]

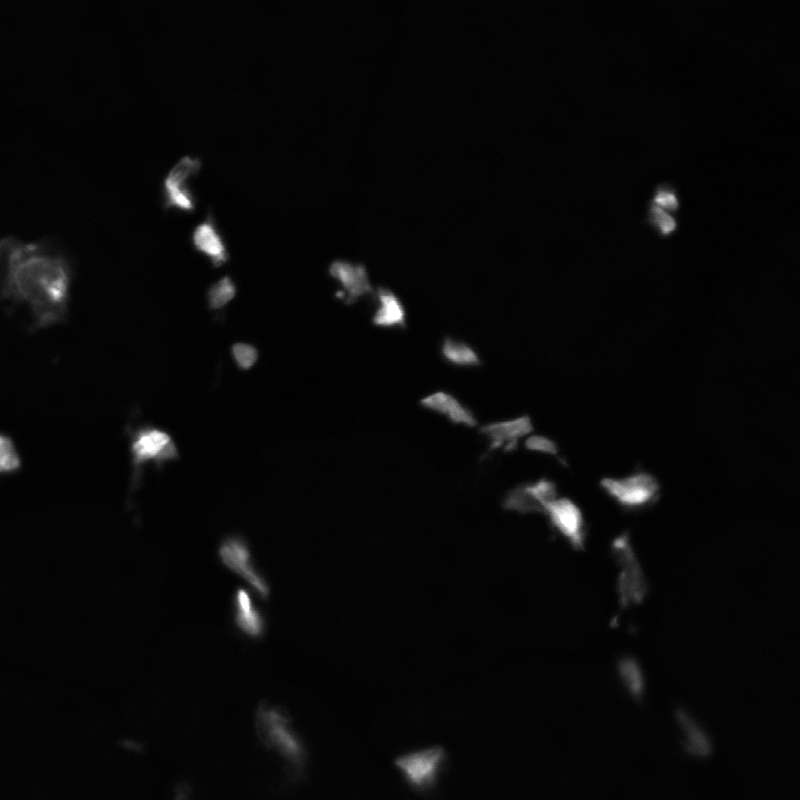

Supplement: Supplementary file 3 — Source Data for Expanded View [file EMMM-15-e17405-s003.zip › Figures_EV/Figure_EV_4/Figure_EV_4_A_B_C_D/Figure_EV_4_A/MDX/MDX.tif]

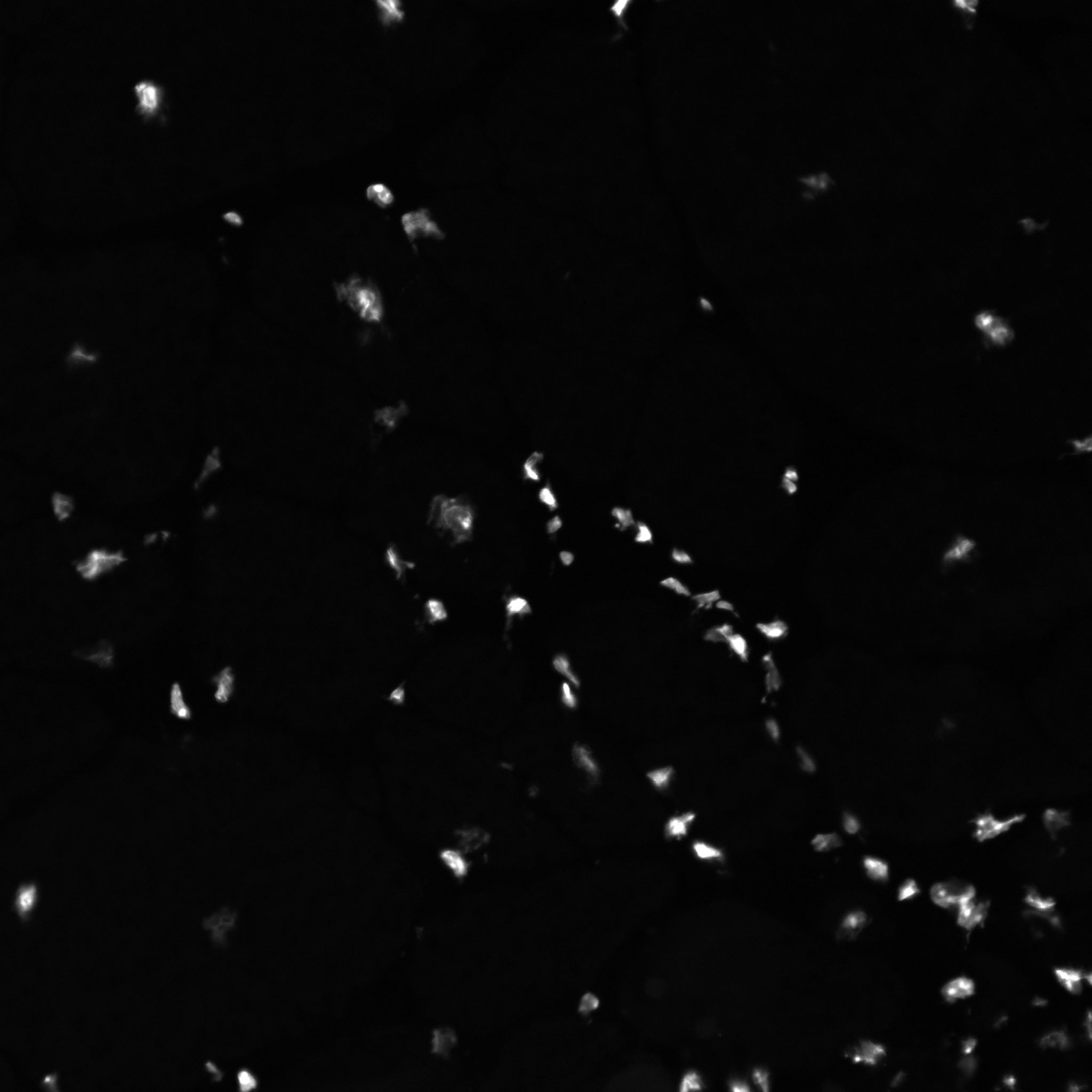

Supplement: Supplementary file 3 — Source Data for Expanded View [file EMMM-15-e17405-s003.zip › Figures_EV/Figure_EV_4/Figure_EV_4_A_B_C_D/Figure_EV_4_A/MDX/MDX_uncropped.tif]

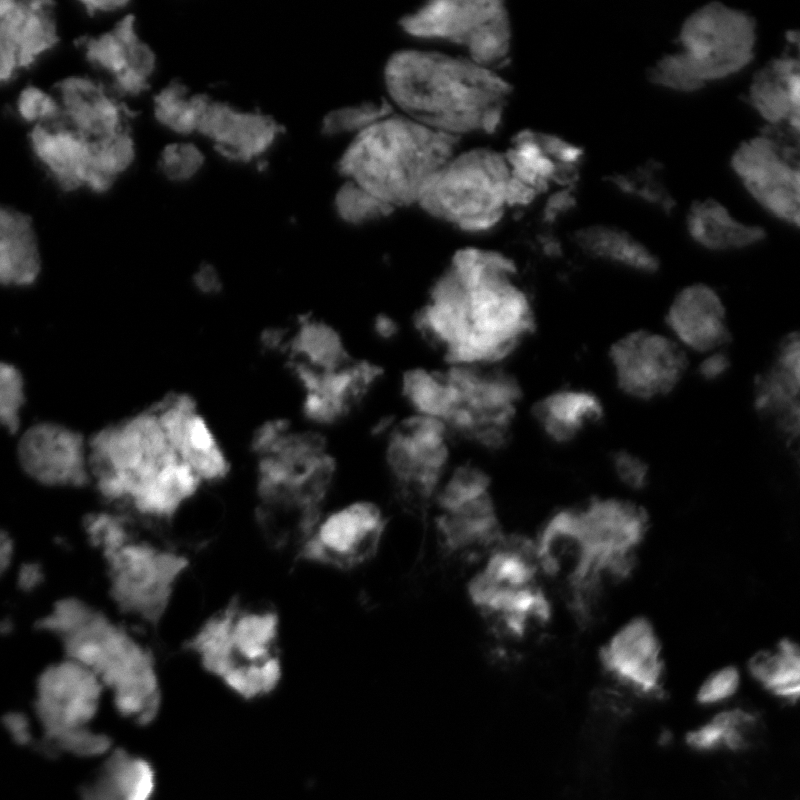

Supplement: Supplementary file 3 — Source Data for Expanded View [file EMMM-15-e17405-s003.zip › Figures_EV/Figure_EV_4/Figure_EV_4_A_B_C_D/Figure_EV_4_A/WT_INJ/WT_INJ.tif]

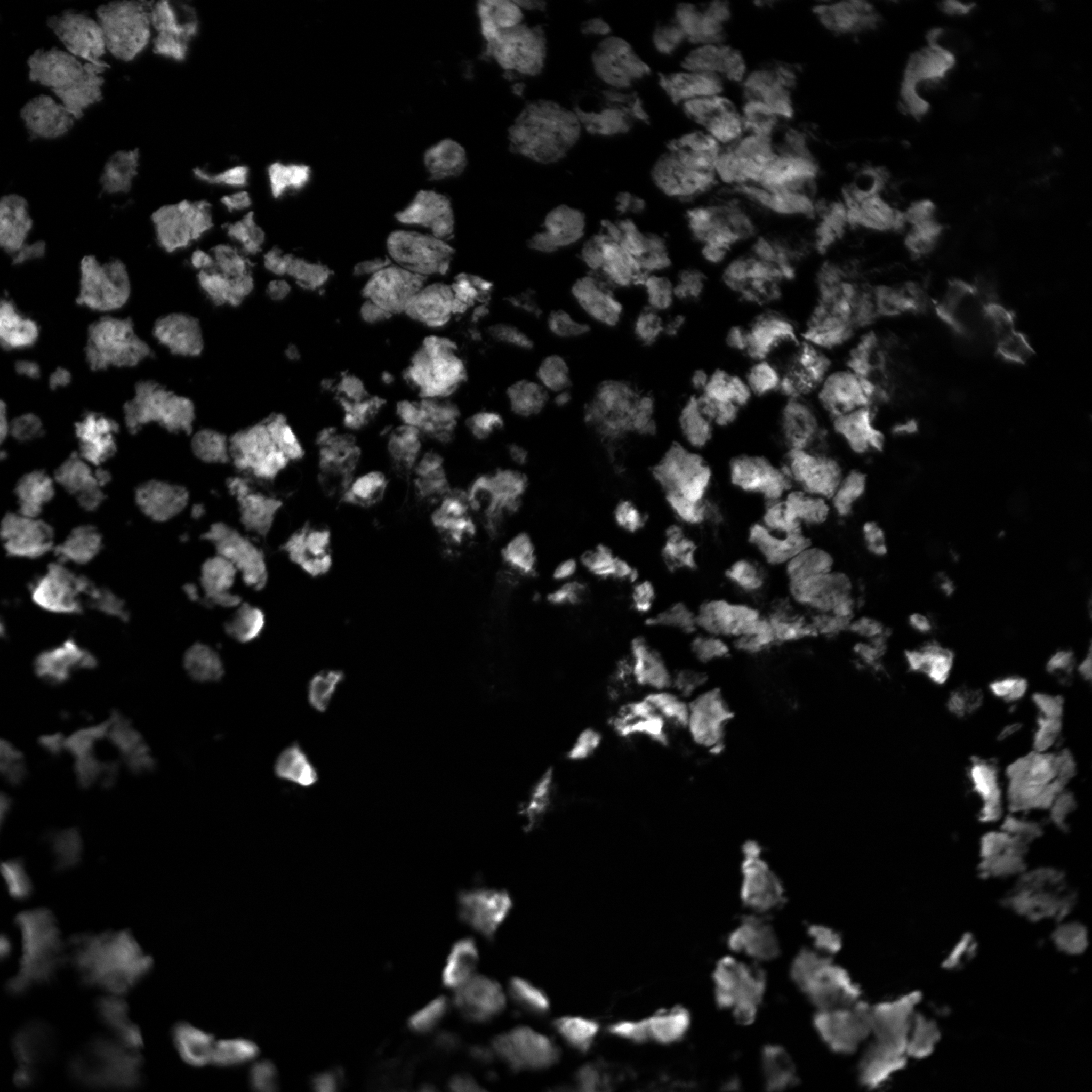

Supplement: Supplementary file 3 — Source Data for Expanded View [file EMMM-15-e17405-s003.zip › Figures_EV/Figure_EV_4/Figure_EV_4_A_B_C_D/Figure_EV_4_A/WT_INJ/WT_INJ_uncropped.tif]

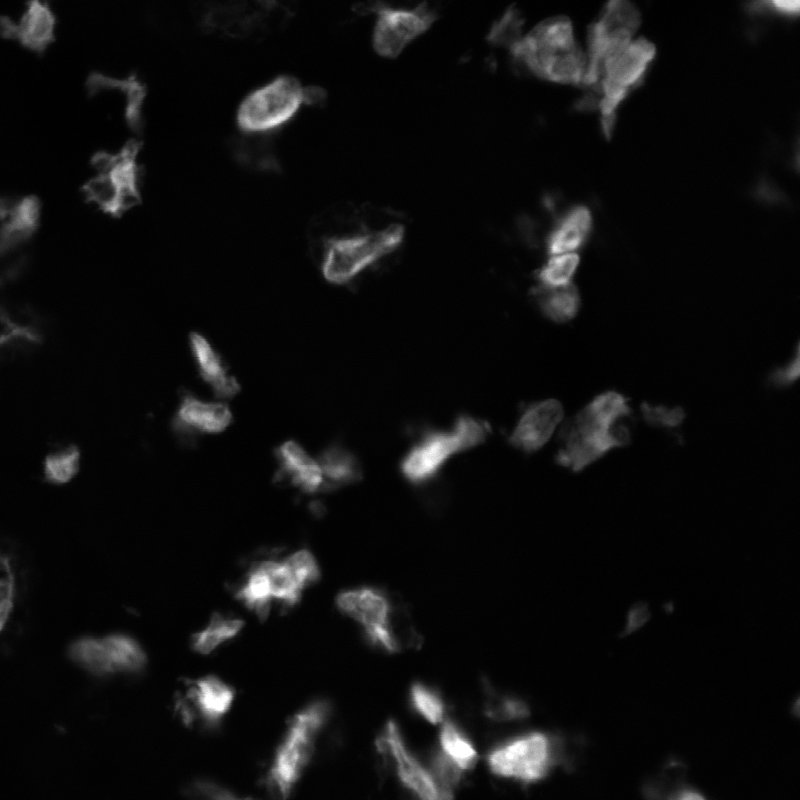

Supplement: Supplementary file 3 — Source Data for Expanded View [file EMMM-15-e17405-s003.zip › Figures_EV/Figure_EV_4/Figure_EV_4_A_B_C_D/Figure_EV_4_C/Fib_MDX.tif]

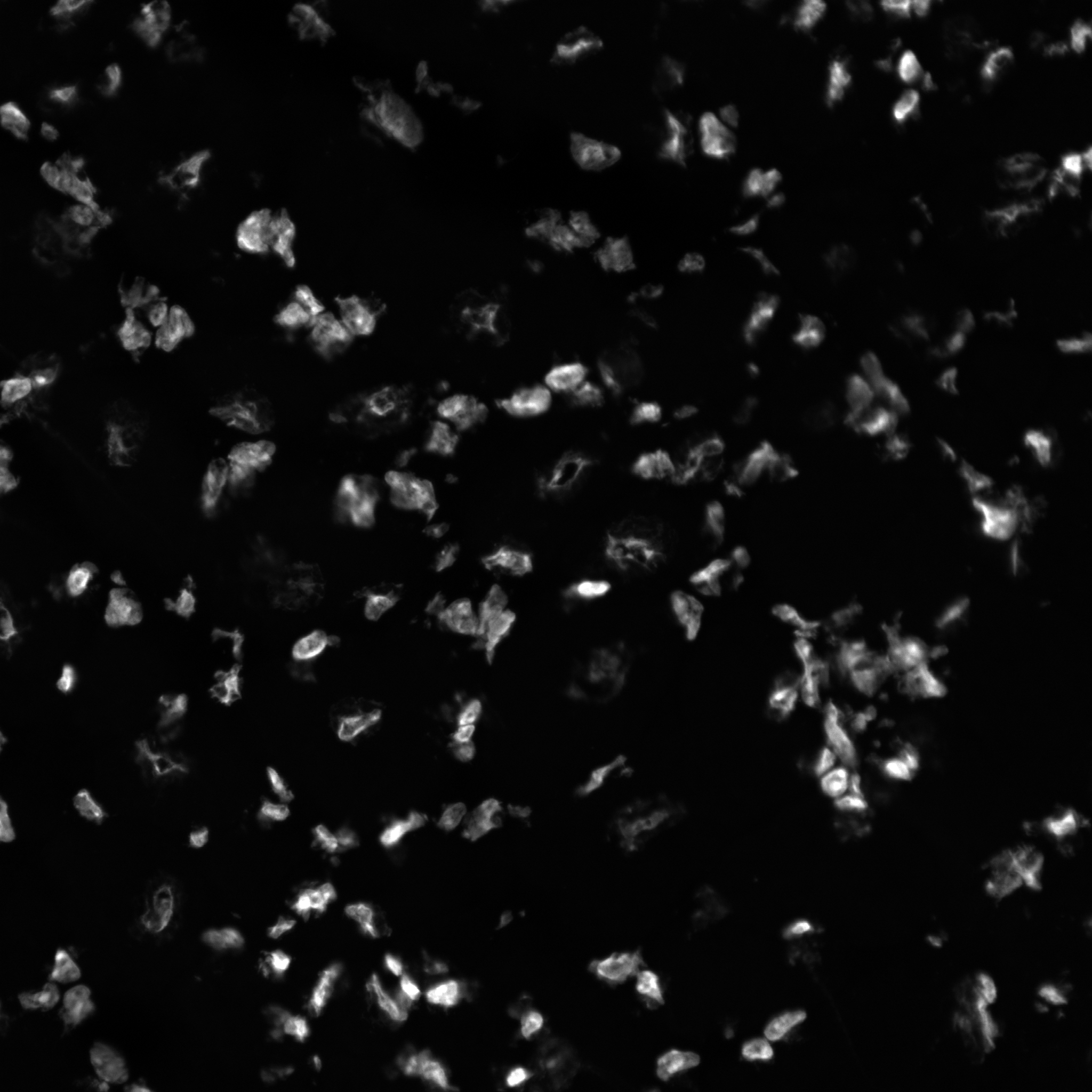

Supplement: Supplementary file 3 — Source Data for Expanded View [file EMMM-15-e17405-s003.zip › Figures_EV/Figure_EV_4/Figure_EV_4_A_B_C_D/Figure_EV_4_C/Fib_MDX_uncropped.tif]

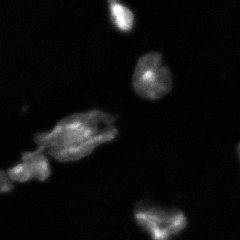

Supplement: Supplementary file 3 — Source Data for Expanded View [file EMMM-15-e17405-s003.zip › Figures_EV/Figure_EV_4/Figure_EV_4_I/Fib-MDX/Fib-MDX_high_magnification.tif]

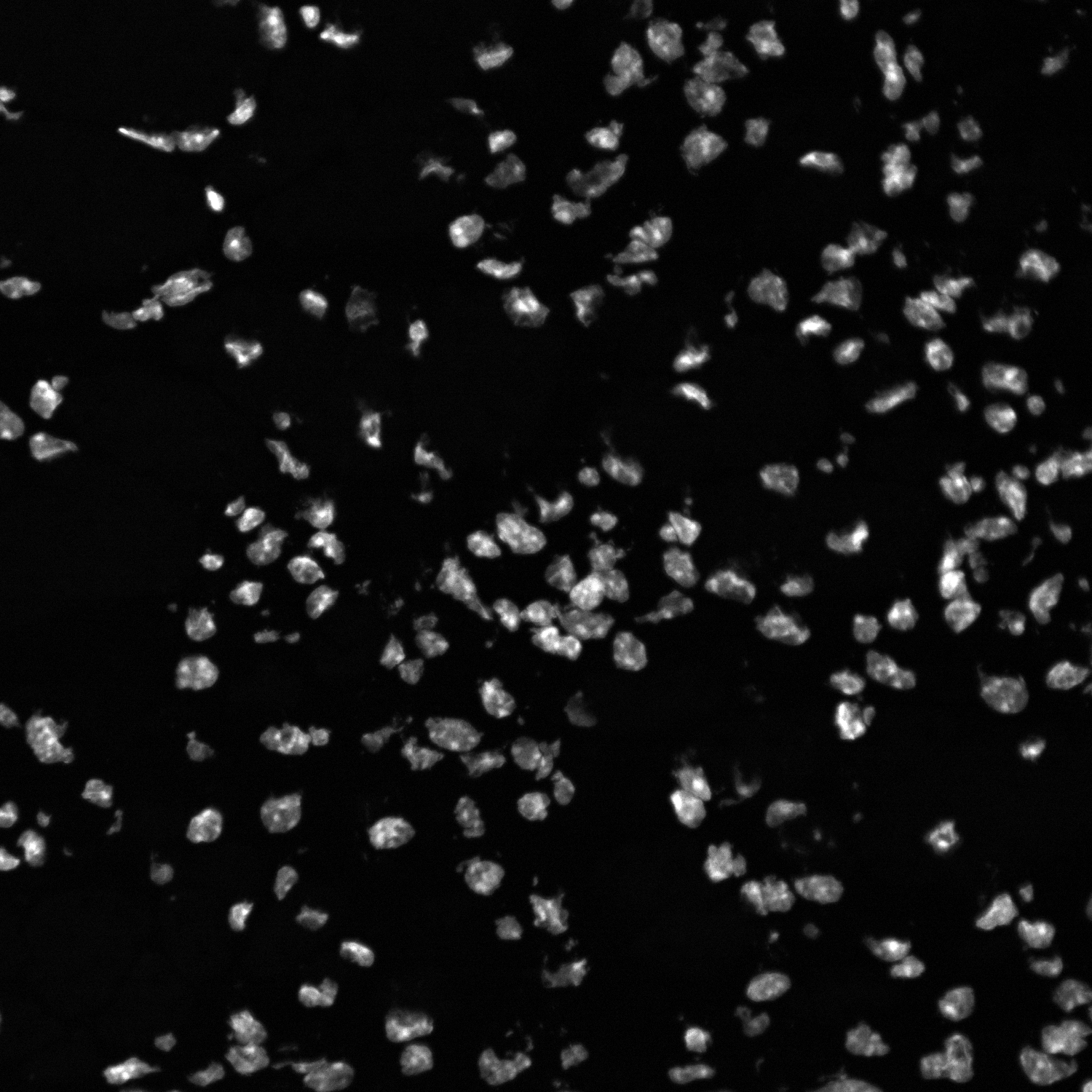

Supplement: Supplementary file 3 — Source Data for Expanded View [file EMMM-15-e17405-s003.zip › Figures_EV/Figure_EV_4/Figure_EV_4_I/Fib-MDX/Fib-MDX_low_magnification.tif]

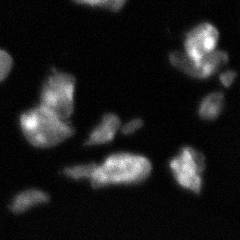

Supplement: Supplementary file 3 — Source Data for Expanded View [file EMMM-15-e17405-s003.zip › Figures_EV/Figure_EV_4/Figure_EV_4_I/MDX/MDX_high_magnification.tif]

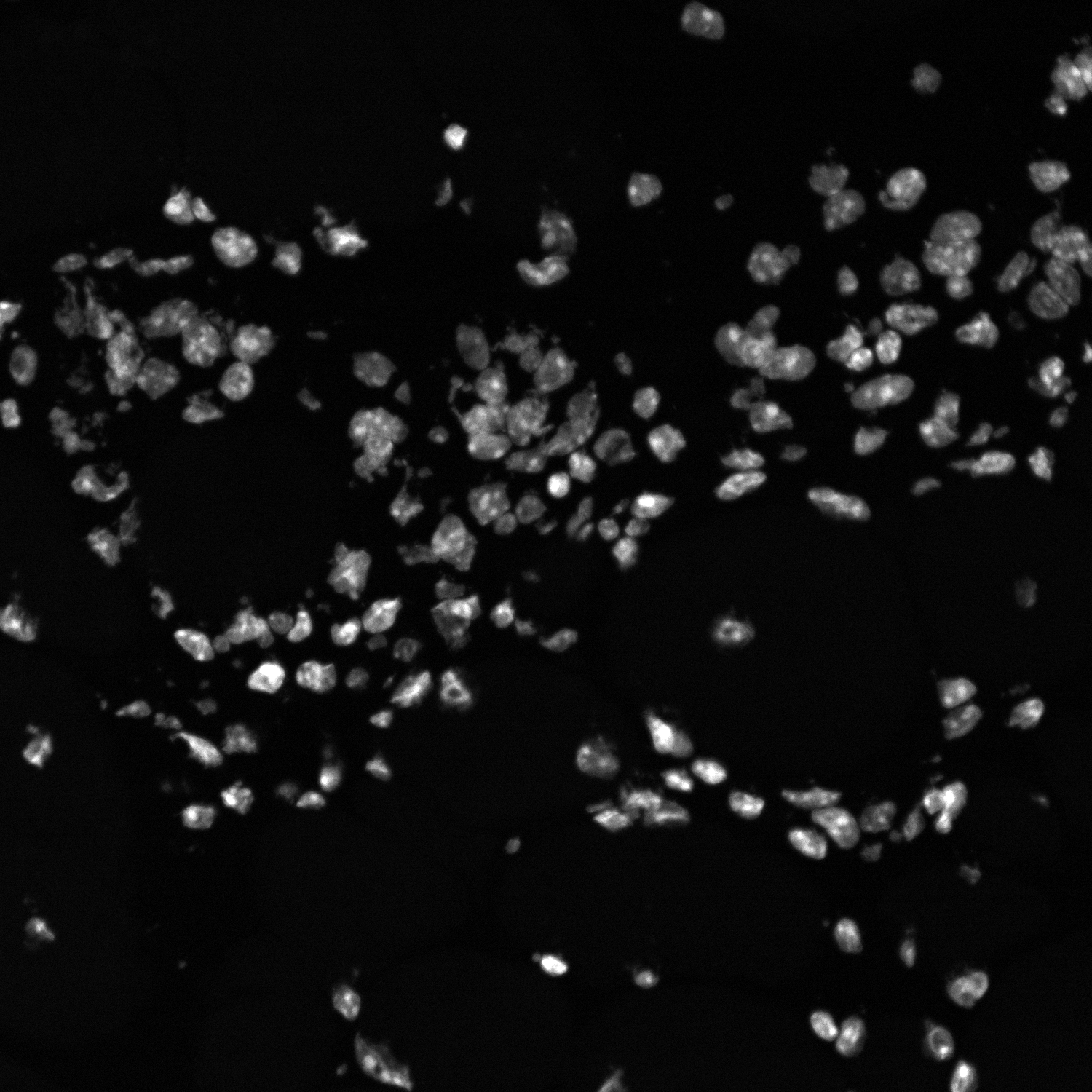

Supplement: Supplementary file 3 — Source Data for Expanded View [file EMMM-15-e17405-s003.zip › Figures_EV/Figure_EV_4/Figure_EV_4_I/MDX/MDX_low_magnification.tif]

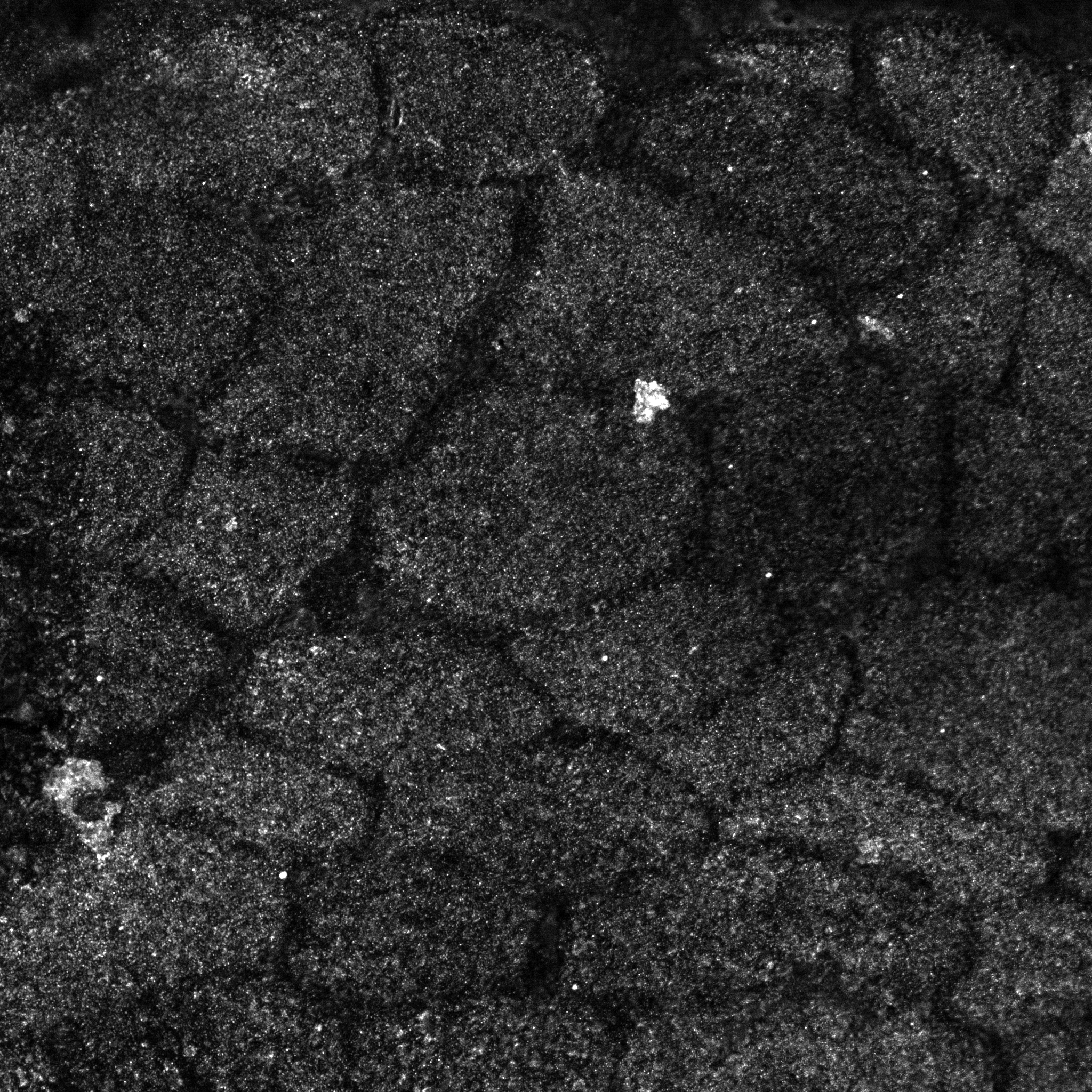

Supplement: Supplementary file 5 — Source Data for Figure 1 [file EMMM-15-e17405-s007.zip › Figure_1/Figure_1_M/CNTR/CNTR_AXIN2.tif]

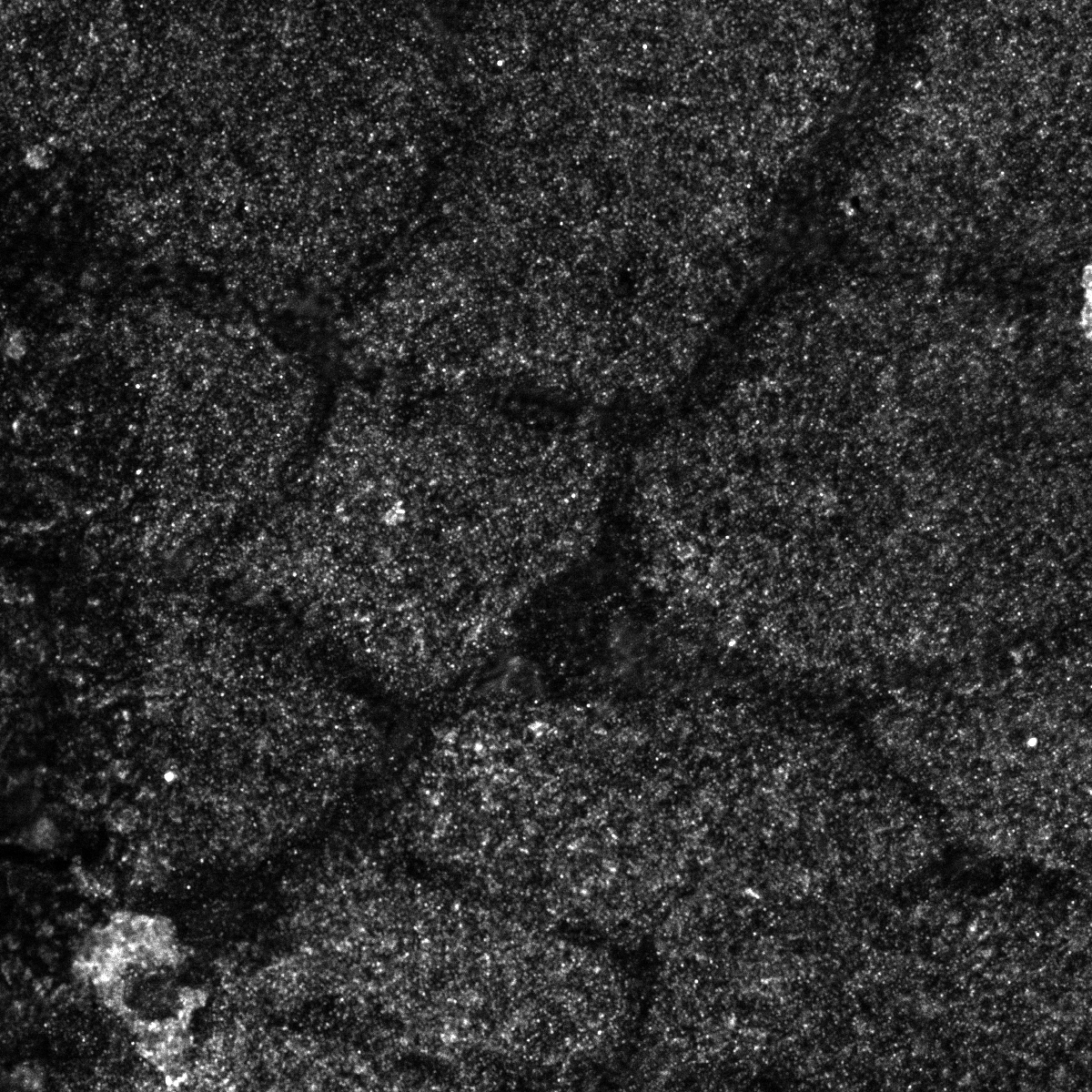

Supplement: Supplementary file 5 — Source Data for Figure 1 [file EMMM-15-e17405-s007.zip › Figure_1/Figure_1_M/CNTR/CNTR_AXIN2_uncropped.tif]

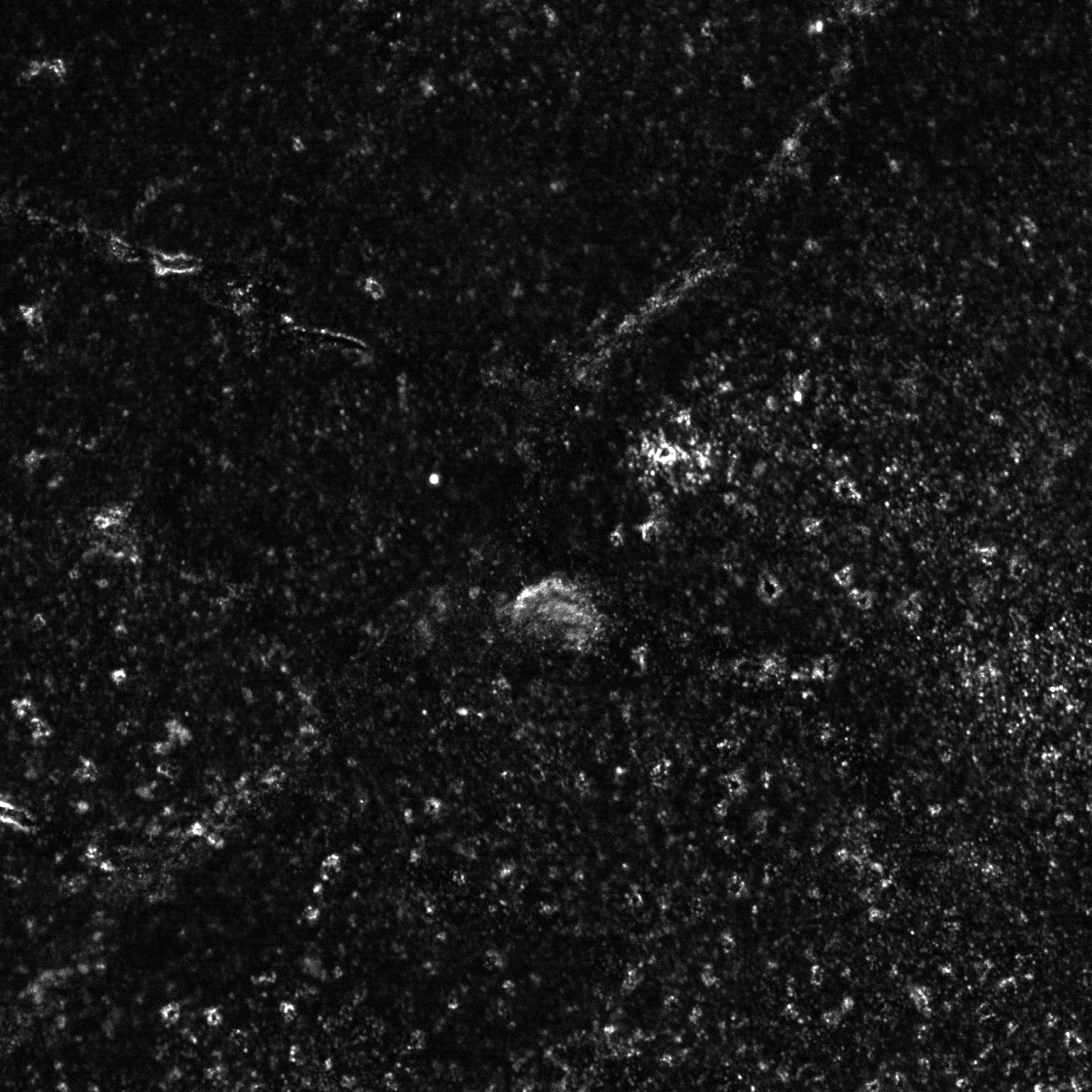

Supplement: Supplementary file 5 — Source Data for Figure 1 [file EMMM-15-e17405-s007.zip › Figure_1/Figure_1_M/CNTR/CNTR_C1s.tif]

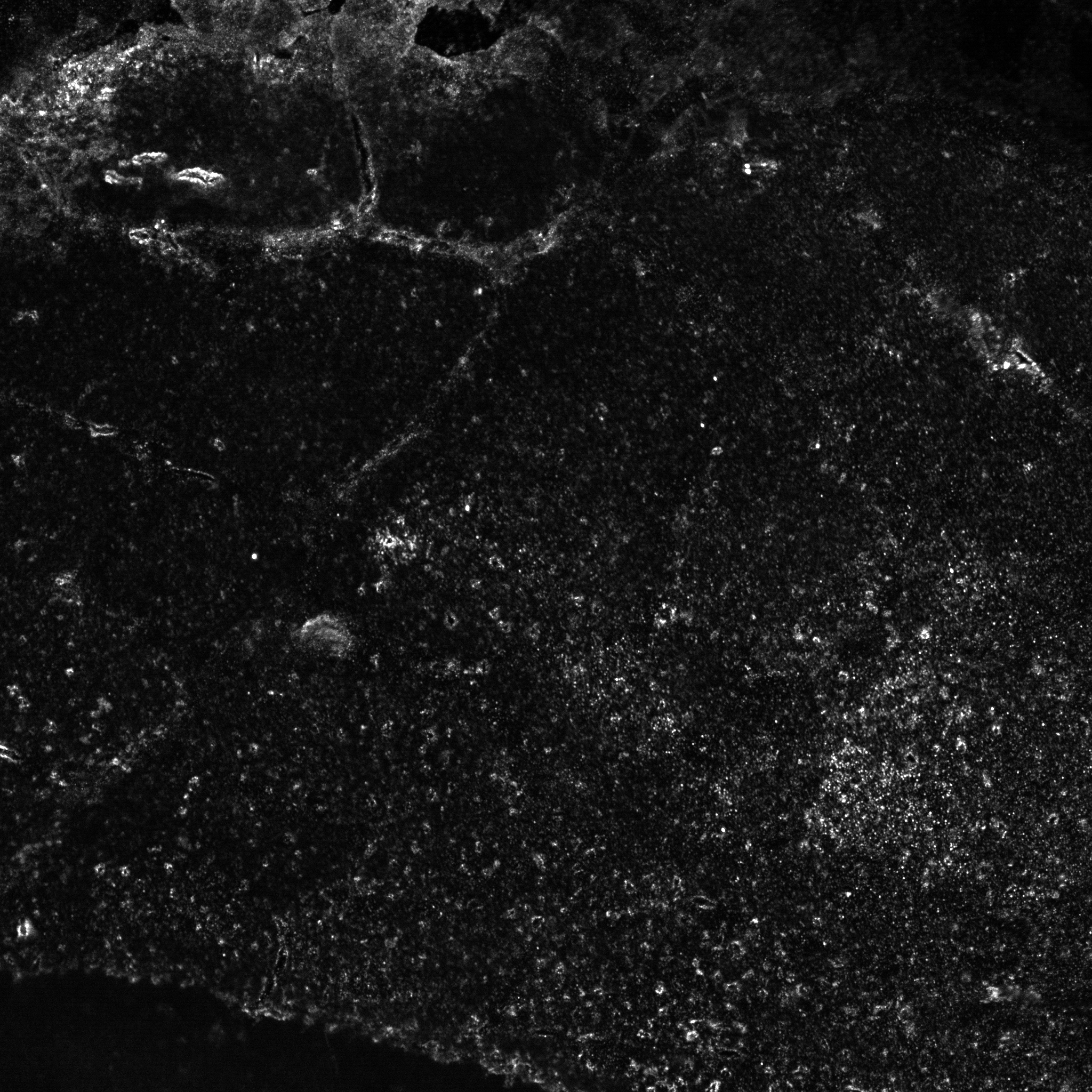

Supplement: Supplementary file 5 — Source Data for Figure 1 [file EMMM-15-e17405-s007.zip › Figure_1/Figure_1_M/CNTR/CNTR_C1S_uncropped.tif]

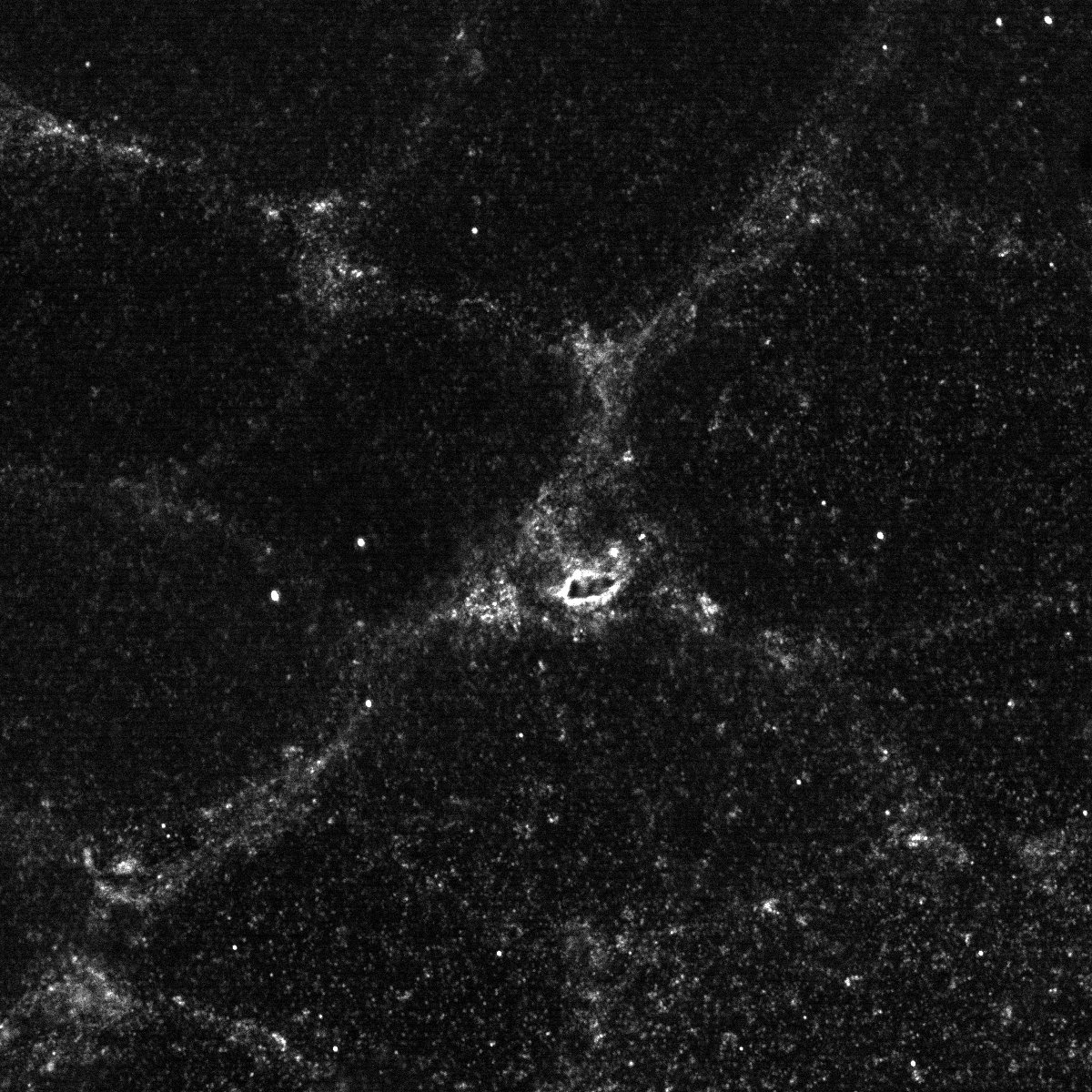

Supplement: Supplementary file 5 — Source Data for Figure 1 [file EMMM-15-e17405-s007.zip › Figure_1/Figure_1_M/CNTR/CNTR_TGFB2.tif]

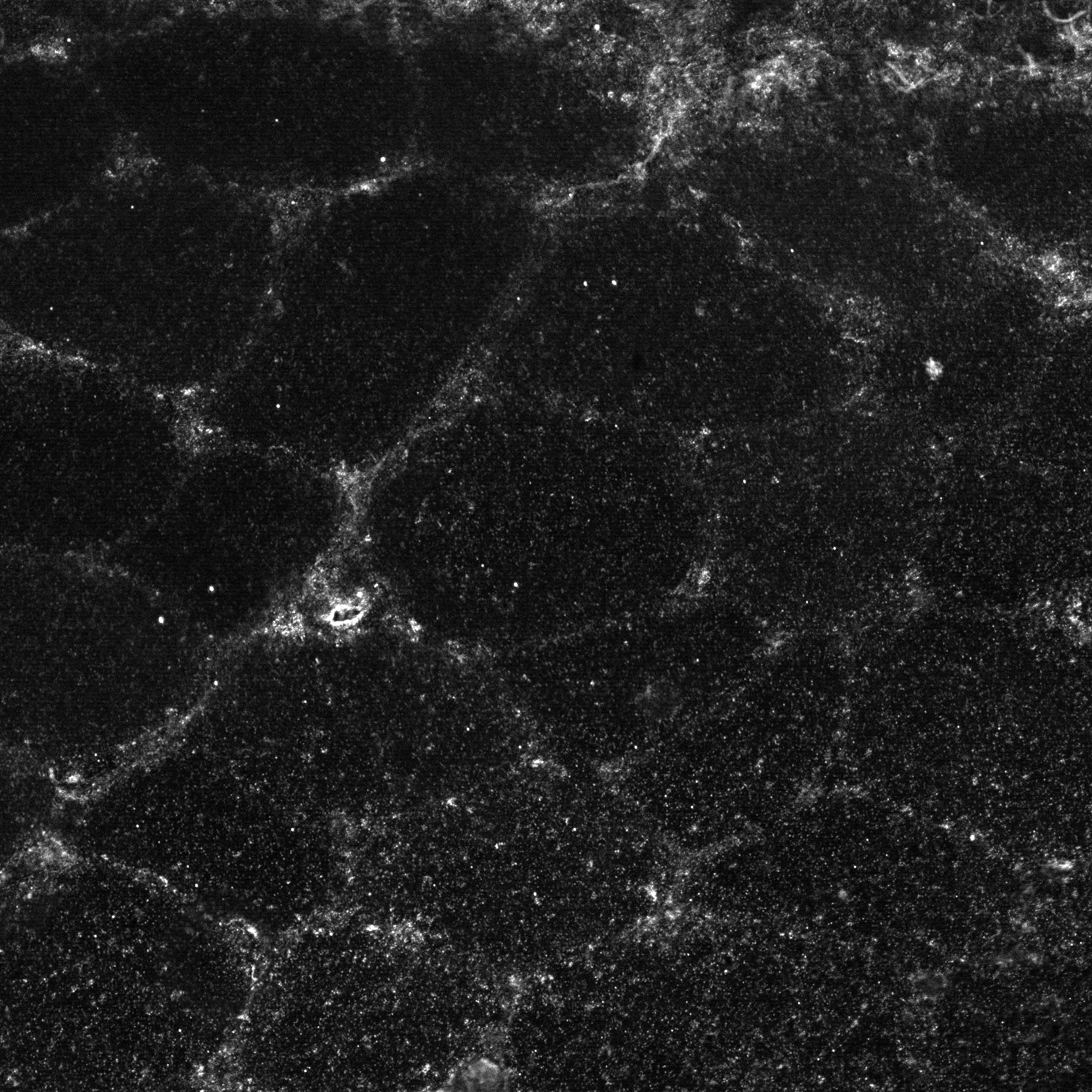

Supplement: Supplementary file 5 — Source Data for Figure 1 [file EMMM-15-e17405-s007.zip › Figure_1/Figure_1_M/CNTR/CNTR_TGFB2_uncropped.tif]

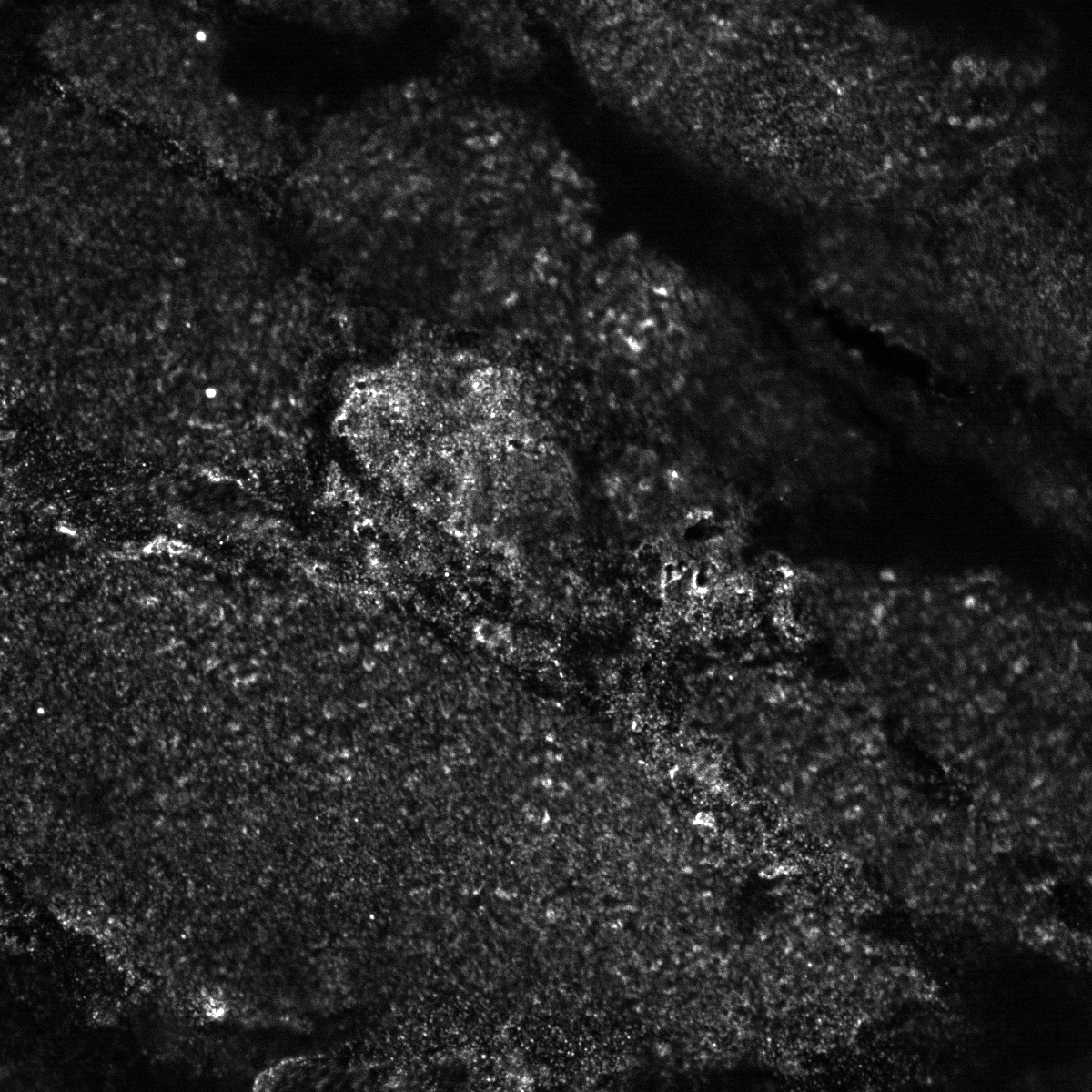

Supplement: Supplementary file 5 — Source Data for Figure 1 [file EMMM-15-e17405-s007.zip › Figure_1/Figure_1_M/DYSTROPHIC/DYSTROPHIC_AXIN2.tif]

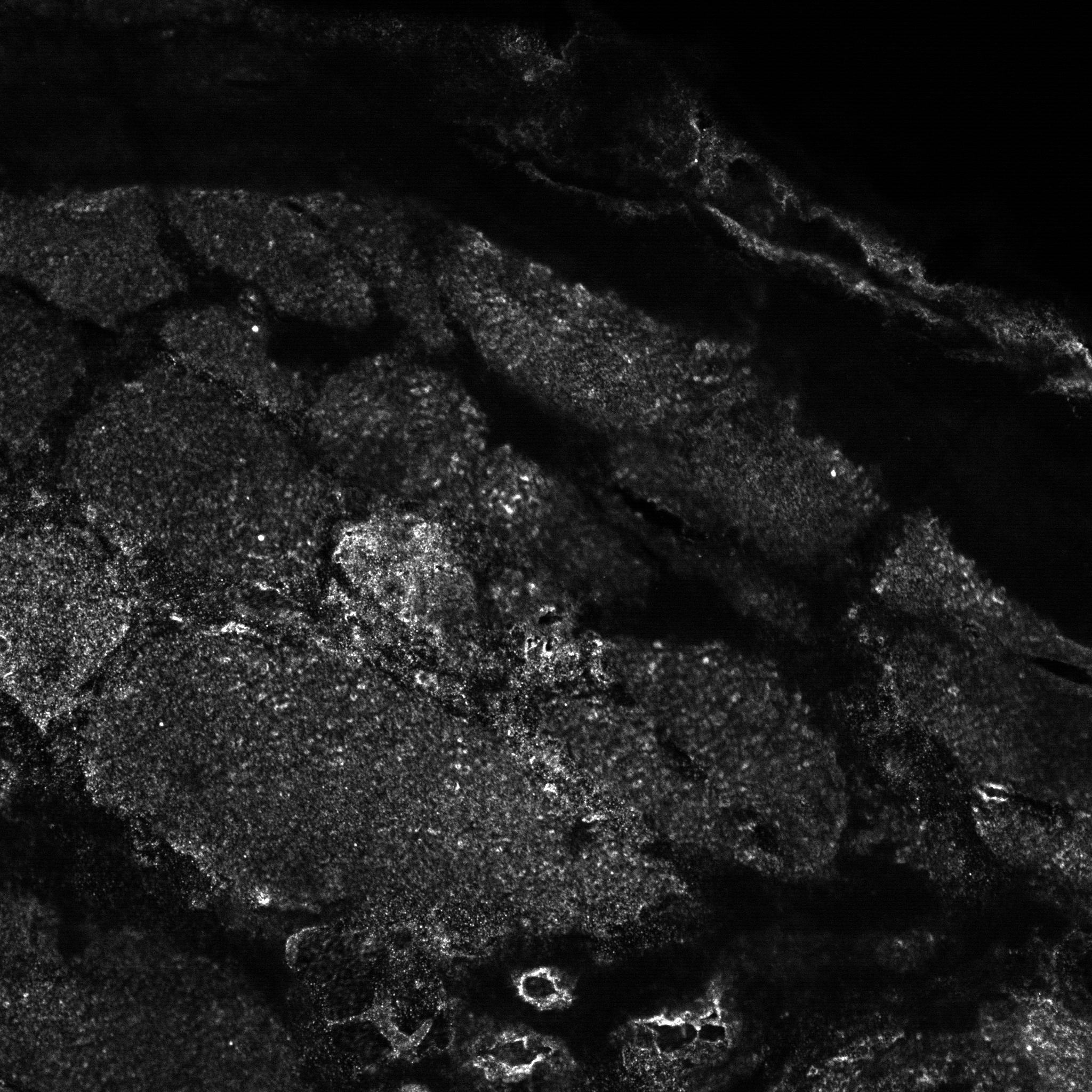

Supplement: Supplementary file 5 — Source Data for Figure 1 [file EMMM-15-e17405-s007.zip › Figure_1/Figure_1_M/DYSTROPHIC/DYSTROPHIC_AXIN2_uncropped.tif]

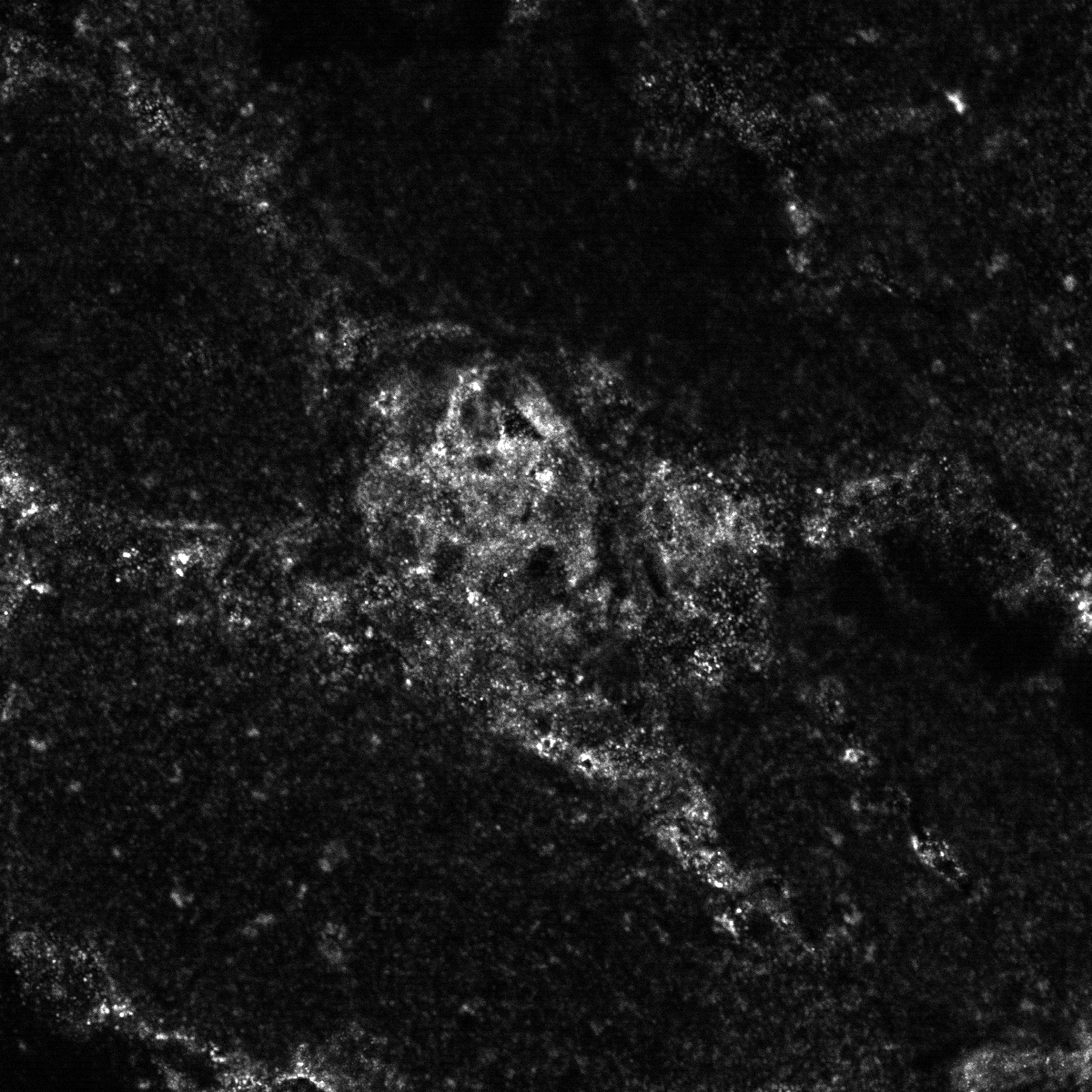

Supplement: Supplementary file 5 — Source Data for Figure 1 [file EMMM-15-e17405-s007.zip › Figure_1/Figure_1_M/DYSTROPHIC/DYSTROPHIC_C1s.tif]

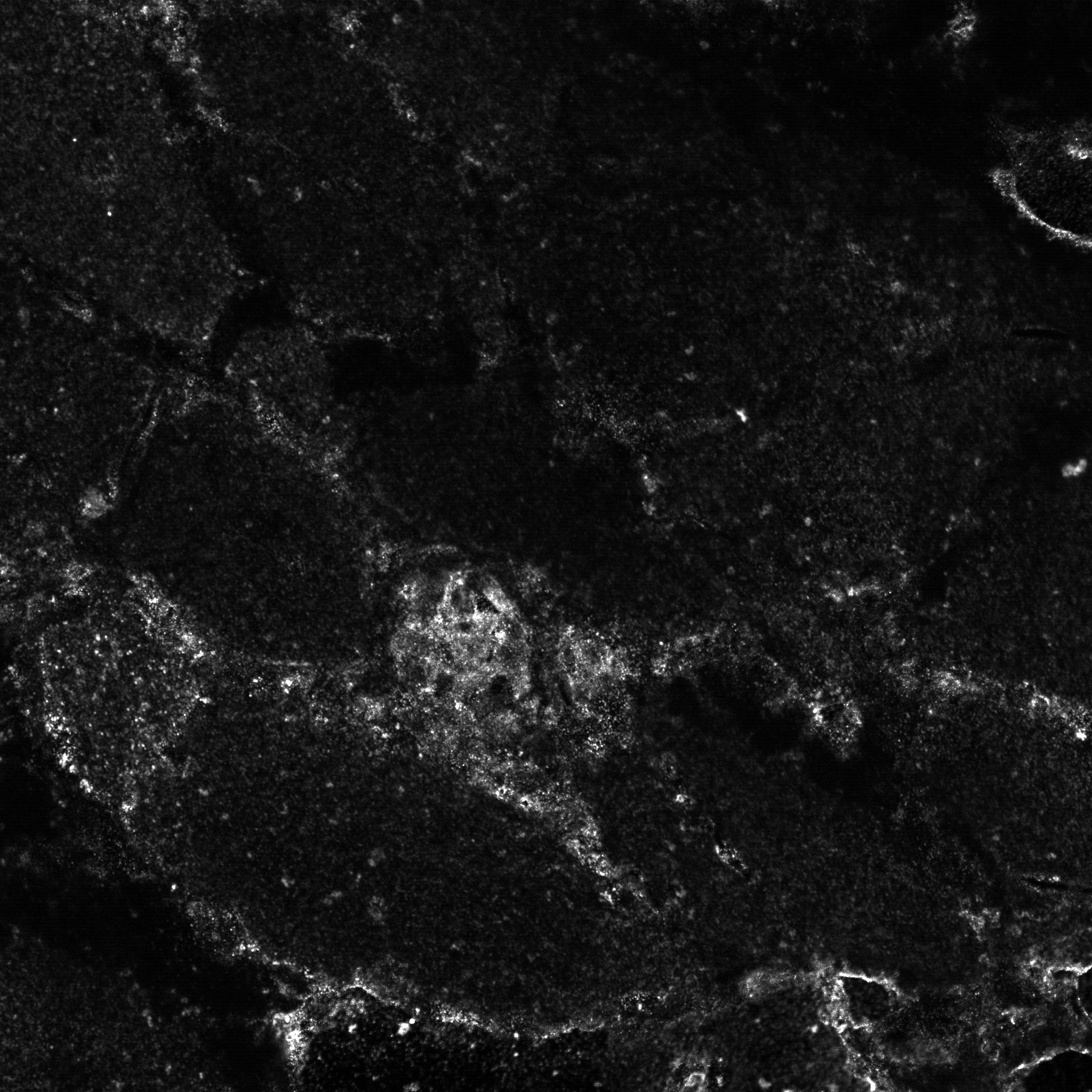

Supplement: Supplementary file 5 — Source Data for Figure 1 [file EMMM-15-e17405-s007.zip › Figure_1/Figure_1_M/DYSTROPHIC/DYSTROPHIC_C1s_uncropped.tif]

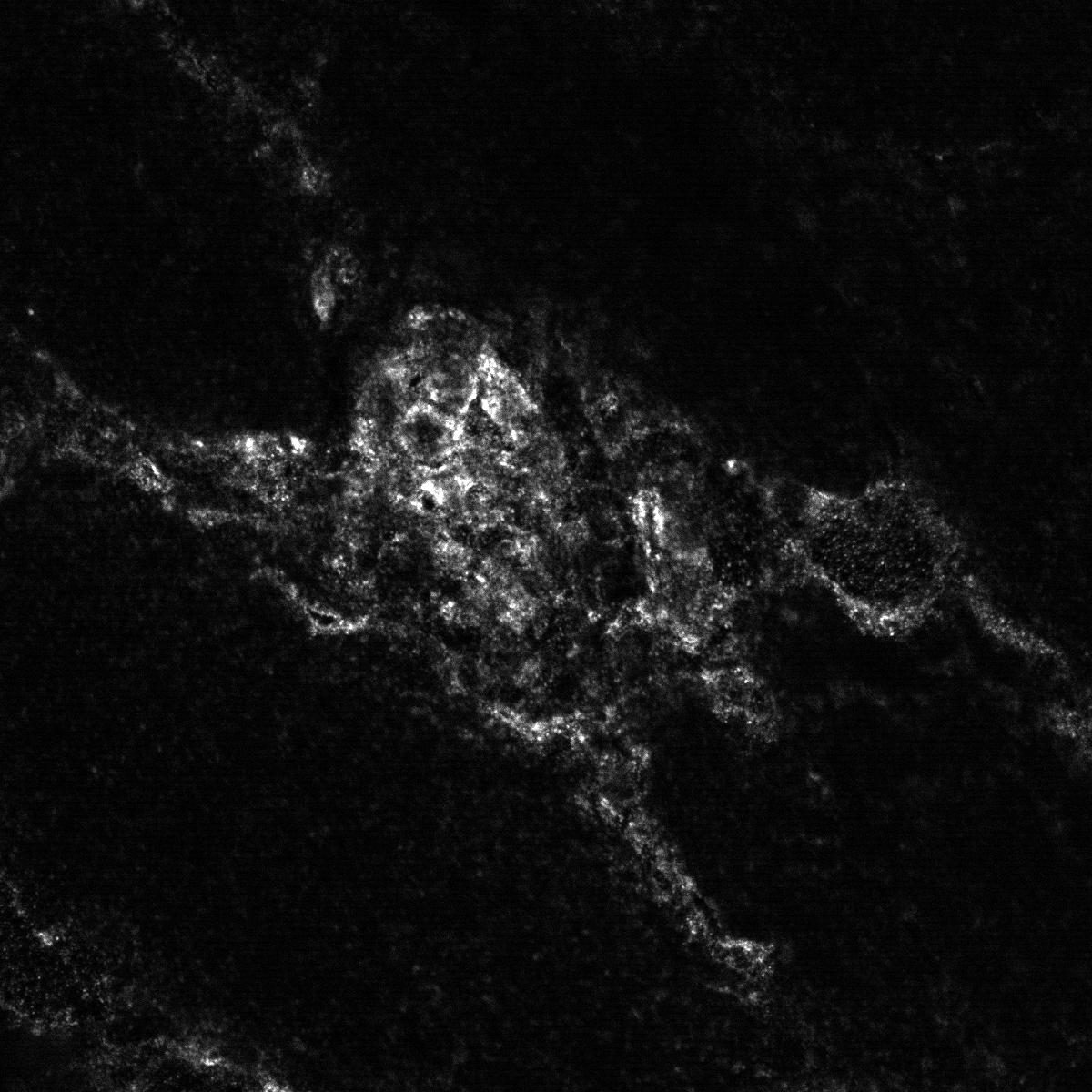

Supplement: Supplementary file 5 — Source Data for Figure 1 [file EMMM-15-e17405-s007.zip › Figure_1/Figure_1_M/DYSTROPHIC/DYSTROPHIC_TGFB2.tif]

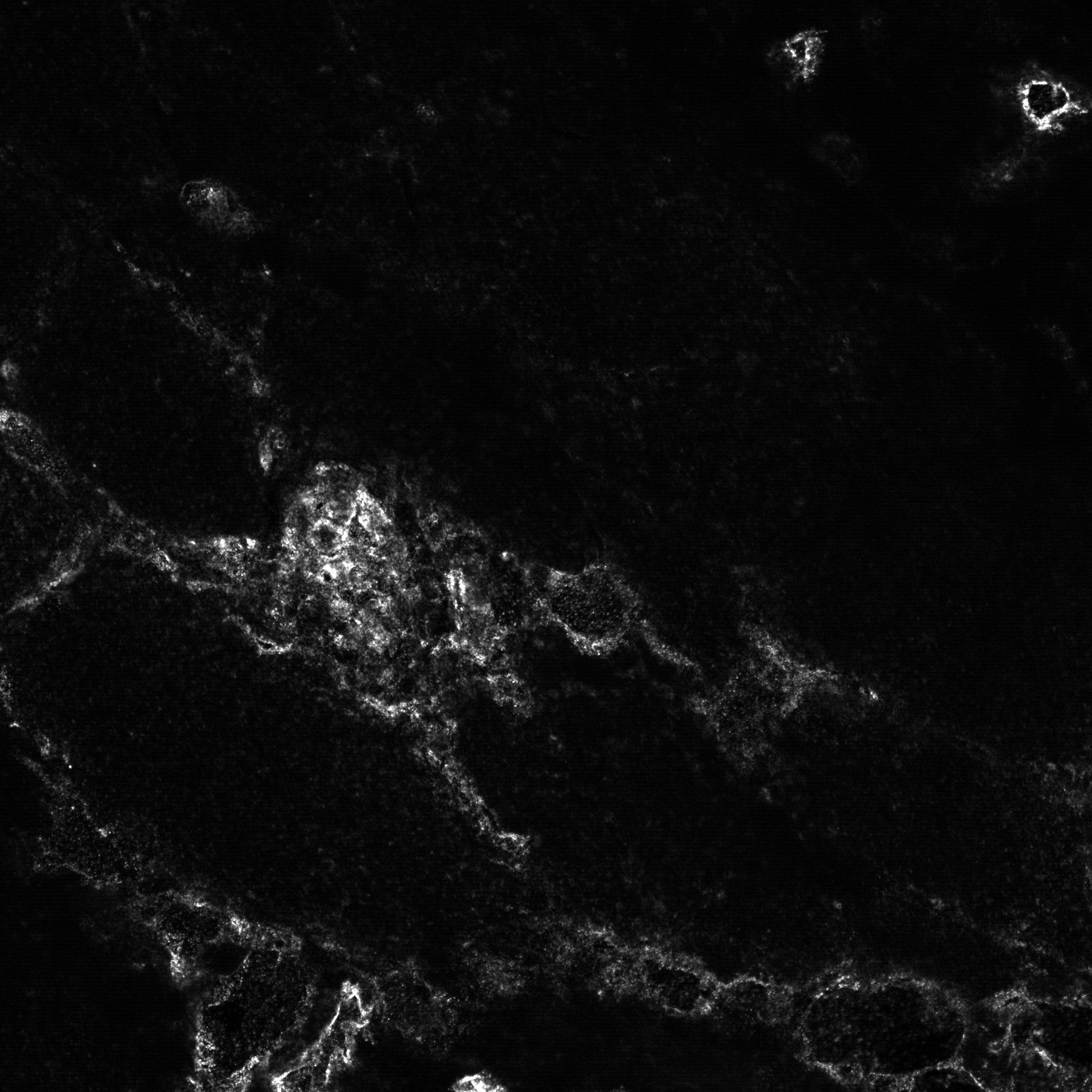

Supplement: Supplementary file 5 — Source Data for Figure 1 [file EMMM-15-e17405-s007.zip › Figure_1/Figure_1_M/DYSTROPHIC/DYSTROPHIC_TGFB2_uncropped.tif]

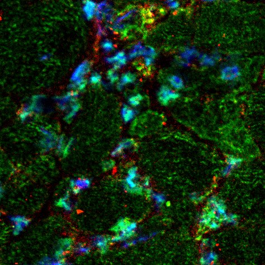

Supplement: Supplementary file 8 — Source Data for Figure 4 [file EMMM-15-e17405-s006.zip › Figure_4/Figure_4_D_E/Figure_4_D/C1q_Axin2_high_magnification.tif]

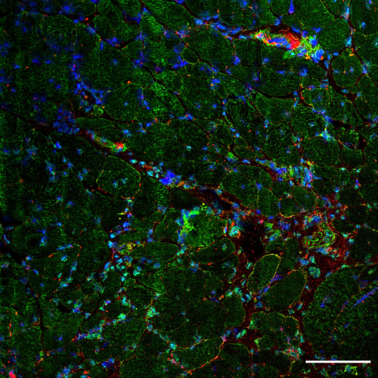

Supplement: Supplementary file 8 — Source Data for Figure 4 [file EMMM-15-e17405-s006.zip › Figure_4/Figure_4_D_E/Figure_4_D/C1q_Axin2_low_magnification.tif]

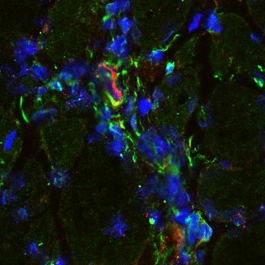

Supplement: Supplementary file 8 — Source Data for Figure 4 [file EMMM-15-e17405-s006.zip › Figure_4/Figure_4_D_E/Figure_4_E/C1q_TGFB2_high_magnification.tif]

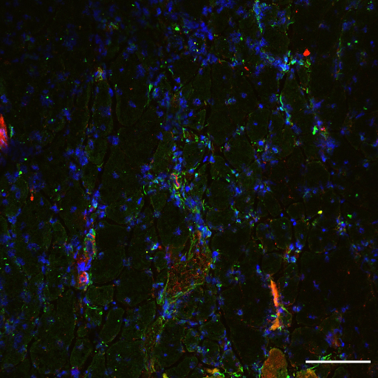

Supplement: Supplementary file 8 — Source Data for Figure 4 [file EMMM-15-e17405-s006.zip › Figure_4/Figure_4_D_E/Figure_4_E/C1q_TGFB2_low_magnification.tif]

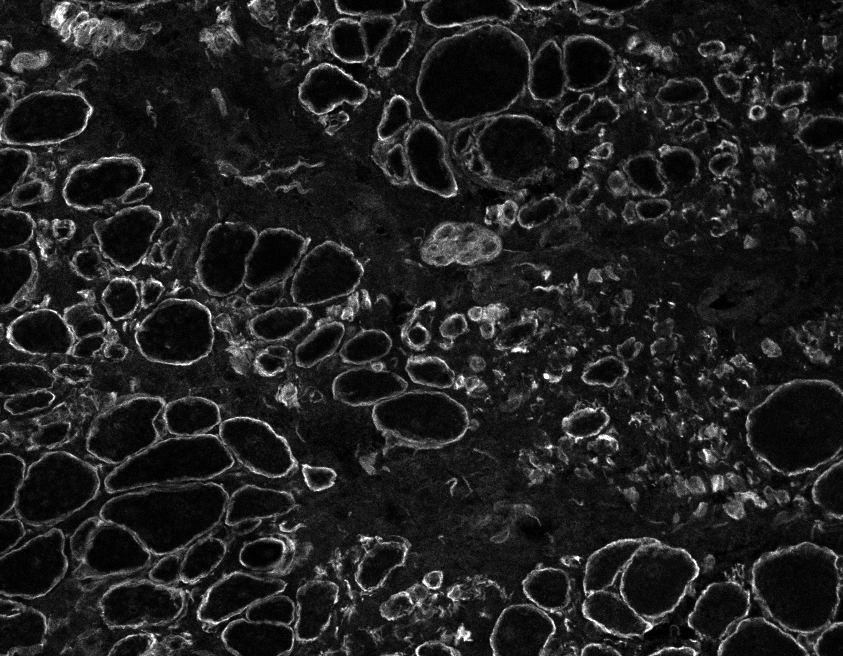

Supplement: Supplementary file 9 — Source Data for Figure 5 [file EMMM-15-e17405-s001.zip › Figure_5/Figure_5_B/Fib_MDX/Fib_MDX.tif]

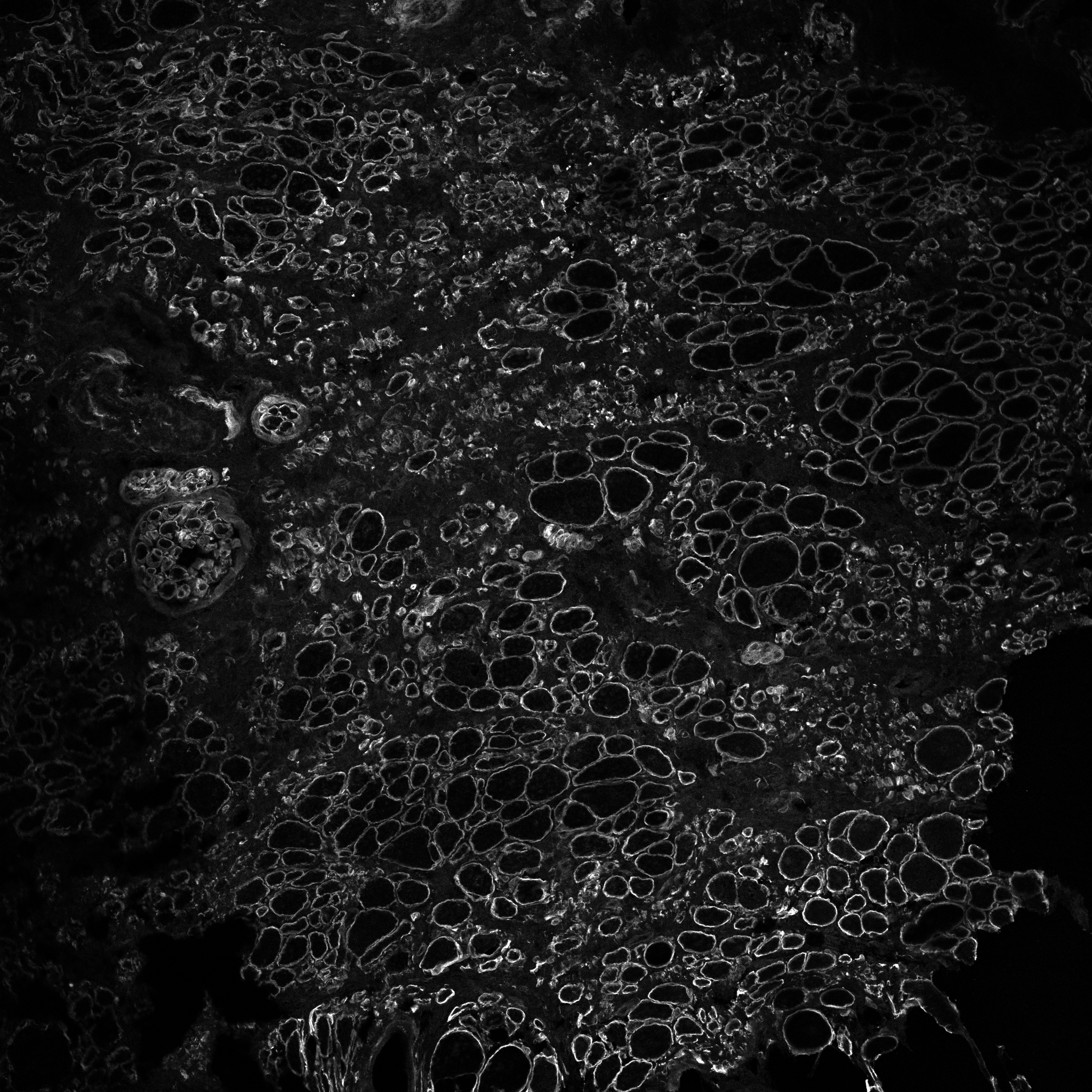

Supplement: Supplementary file 9 — Source Data for Figure 5 [file EMMM-15-e17405-s001.zip › Figure_5/Figure_5_B/Fib_MDX/Fib_MDX_uncropped.tif]

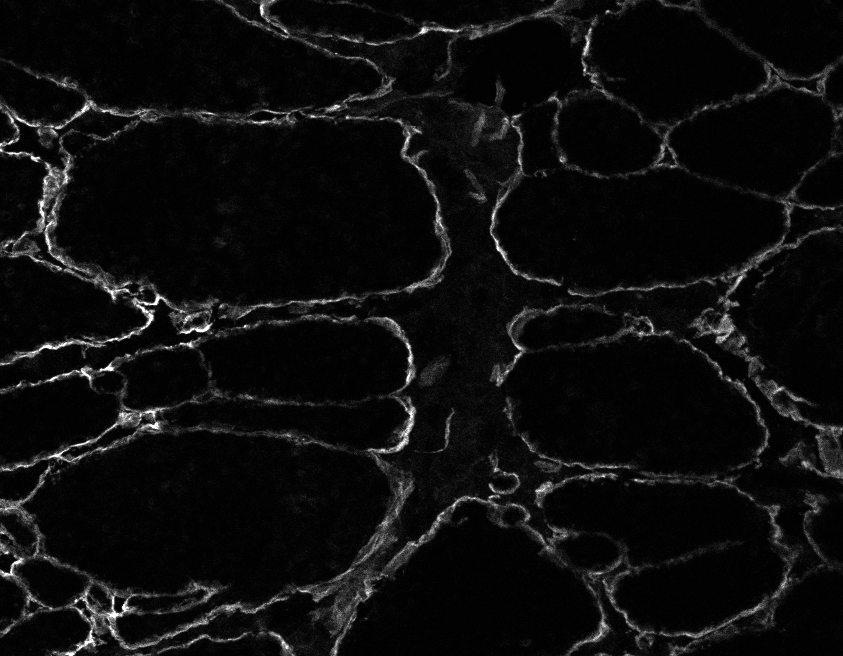

Supplement: Supplementary file 9 — Source Data for Figure 5 [file EMMM-15-e17405-s001.zip › Figure_5/Figure_5_B/MDX/MDX.tif]

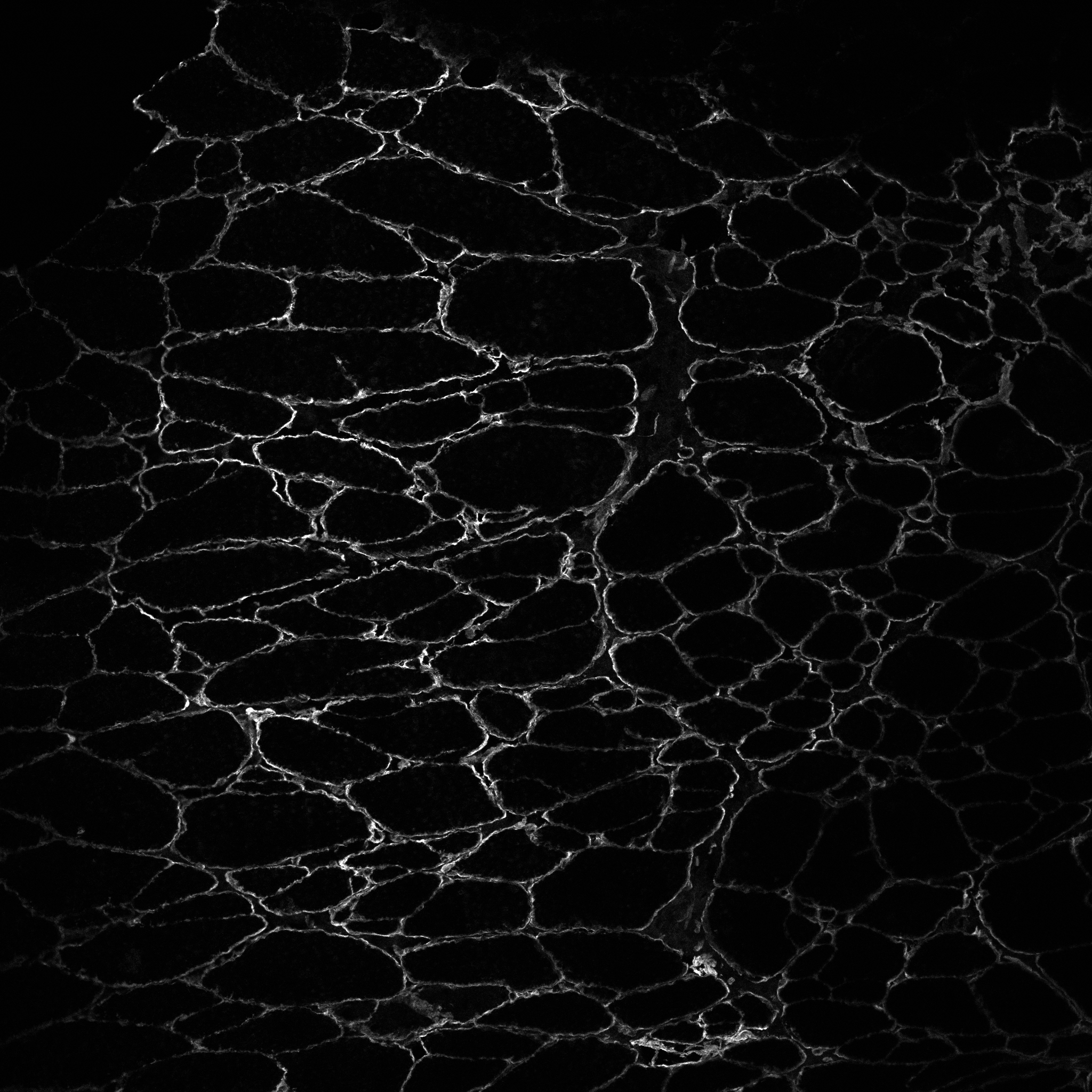

Supplement: Supplementary file 9 — Source Data for Figure 5 [file EMMM-15-e17405-s001.zip › Figure_5/Figure_5_B/MDX/MDX_uncropped.tif]
