# Supplementary material for: Combinatorial activation of the WNT‐dependent fibrogenic program by distinct complement subunits in dystrophic muscle
Source: EMBO Mol Med. 2023 Nov 6;15(12):e17405. doi: 10.15252/emmm.202317405 (PMC10701616; doi:10.15252/emmm.202317405)
Supplement: Supplementary file 11 [file EMMM-15-e17405-s008.pdf]

EMBO Molecular Medicine

Combinatorial activation of the WNT-dependent fibrogenic program by distinct complement subunits in dystrophic muscle

Stefano Biressi, Francesca Florio, Sara Vencato, Filomena Papa, Michela Libergoli, Eyemen Kheir, Imen Ghzaiel, Thomas Rando, and Yvan Torrente
DOI: 10.15252/emmm.202317405

Corresponding author: Stefano Biressi (stefano.biressi@unitn.it)

Review Timeline:

Submission Date:	10th Jan 23
Editorial Decision:	16th Feb 23
Revision Received:	16th Sep 23
Editorial Decision:	4th Oct 23
Revision Received:	10th Oct 23
Accepted:	11th Oct 23

Editor: Zeljko Durdevic

Transaction Report:

(Note: With the exception of the correction of typographical or spelling errors that could be a source of ambiguity, letters and reports are not edited. Depending on transfer agreements, referee reports obtained elsewhere may or may not be included in this compilation. Referee reports are anonymous unless the Referee chooses to sign their reports.)

16th Feb 2023

Dear Prof. Biressi,

Thank you for the submission of your manuscript to EMBO Molecular Medicine. Please accept my apologies for the unusual delay in getting back to you due to the longer time one referee required to complete evaluation of your manuscript.

We have now received feedback from the three reviewers who agreed to evaluate your manuscript. As you will see from the reports below, the referees acknowledge the interest of the study but also raise serious concerns that should be addressed in a major revision. Experiment suggested by the referee #2 regarding testing muscles at different time points is not required and should be addressed in writing. If you would like to discuss further the points raised by the referees, I am available to do so via email or video. Let me know if you are interested in this option.

Further consideration of a revision that addresses reviewers' concerns in full will entail a second round of review. EMBO Molecular Medicine encourages a single round of revision only and therefore, acceptance or rejection of the manuscript will depend on the completeness of your responses included in the next, final version of the manuscript. For this reason, and to save you from any frustrations in the end, I would strongly advise against returning an incomplete revision.

We would welcome the submission of a revised version within three months for further consideration. Please let us know if you require longer to complete the revision.

I look forward to receiving your revised manuscript.

Yours sincerely,

Zeljko Durdevic

Zeljko Durdevic
Editor
EMBO Molecular Medicine

*****

When submitting your revised manuscript, please carefully review the instructions that follow below. We perform an initial quality control of all revised manuscripts before re-review; failure to include requested items will delay the evaluation of your revision.

We require:

1) A .docx formatted version of the manuscript text (including legends for main figures, EV figures and tables). Please make sure that the changes are highlighted to be clearly visible.

2) Individual production quality figure files as .eps, .tif, .jpg (one file per figure). For guidance, download the 'Figure Guide PDF': (https://www.embopress.org/page/journal/17574684/authorguide#figureformat).

3) A .docx formatted letter INCLUDING the reviewers' reports and your detailed point-by-point responses to their comments. As part of the EMBO Press transparent editorial process, the point-by-point response is part of the Review Process File (RPF), which will be published alongside your paper.

4) A complete author checklist, which you can download from our author guidelines (https://www.embopress.org/page/journal/17574684/authorguide#submissionofrevisions). Please insert information in the checklist that is also reflected in the manuscript. The completed author checklist will also be part of the RPF.

5) Please note that all corresponding authors are required to supply an ORCID ID for their name upon submission of a revised

manuscript.

6) It is mandatory to include a 'Data Availability' section after the Materials and Methods. Before submitting your revision, primary datasets produced in this study need to be deposited in an appropriate public database, and the accession numbers and database listed under 'Data Availability'. Please remember to provide a reviewer password if the datasets are not yet public (see https://www.embopress.org/page/journal/17574684/authorguide#dataavailability).

In case you have no data that requires deposition in a public database, please state so in this section. Note that the Data Availability Section is restricted to new primary data that are part of this study.

7) For data quantification: please specify the name of the statistical test used to generate error bars and P values, the number (n) of independent experiments (specify technical or biological replicates) underlying each data point and the test used to calculate p-values in each figure legend. The figure legends should contain a basic description of n, P and the test applied. Graphs must include a description of the bars and the error bars (s.d., s.e.m.). See also 'Figure Legend' guidelines: https://www.embopress.org/page/journal/17574684/authorguide#figureformat

8) At EMBO Press we ask authors to provide source data for the main manuscript figures. Our source data coordinator will contact you to discuss which figure panels we would need source data for and will also provide you with helpful tips on how to upload and organize the files.

9) Our journal encourages inclusion of *data citations in the reference list* to directly cite datasets that were re-used and obtained from public databases. Data citations in the article text are distinct from normal bibliographical citations and should directly link to the database records from which the data can be accessed. In the main text, data citations are formatted as follows: "Data ref: Smith et al, 2001" or "Data ref: NCBI Sequence Read Archive PRJNA342805, 2017". In the Reference list, data citations must be labeled with "[DATASET]". A data reference must provide the database name, accession number/identifiers and a resolvable link to the landing page from which the data can be accessed at the end of the reference. Further instructions are available at .

10) We replaced Supplementary Information with Expanded View (EV) Figures and Tables that are collapsible/expandable online. A maximum of 5 EV Figures can be typeset. EV Figures should be cited as 'Figure EV1, Figure EV2" etc... in the text and their respective legends should be included in the main text after the legends of regular figures.

- For the figures that you do NOT wish to display as Expanded View figures, they should be bundled together with their legends in a single PDF file called *Appendix*, which should start with a short Table of Content. Appendix figures should be referred to in the main text as: "Appendix Figure S1, Appendix Figure S2" etc.

- Additional Tables/Datasets should be labeled and referred to as Table EV1, Dataset EV1, etc. Legends have to be provided in a separate tab in case of .xls files. Alternatively, the legend can be supplied as a separate text file (README) and zipped together with the Table/Dataset file.

See detailed instructions here:

.

11) The paper explained: EMBO Molecular Medicine articles are accompanied by a summary of the articles to emphasize the major findings in the paper and their medical implications for the non-specialist reader. Please provide a draft summary of your article highlighting

- the medical issue you are addressing,

- the results obtained and

- their clinical impact.

This may be edited to ensure that readers understand the significance and context of the research. Please refer to any of our published articles for an example.

12) For more information: There is space at the end of each article to list relevant web links for further consultation by our readers. Could you identify some relevant ones and provide such information as well? Some examples are patient associations, relevant databases, OMIM/proteins/genes links, author's websites, etc...

13) Author contributions: You will be asked to provide CRediT (Contributor Role Taxonomy) terms in the submission system. These replace a narrative author contribution section in the manuscript.

14) A Conflict of Interest statement should be provided in the main text.

15) Every published paper now includes a 'Synopsis' to further enhance discoverability. Synopses are displayed on the journal webpage and are freely accessible to all readers. They include a short stand first (maximum of 300 characters, including space) as well as 2-5 one-sentences bullet points that summarizes the paper. Please write the bullet points to summarize the key NEW findings. They should be designed to be complementary to the abstract - i.e. not repeat the same text. We encourage inclusion of key acronyms and quantitative information (maximum of 30 words / bullet point). Please use the passive voice. Please attach these in a separate file or send them by email, we will incorporate them accordingly.

Please also suggest a striking image or visual abstract to illustrate your article as a PNG file 550 px wide x 300-600 px high.

EMBO Molecular Medicine has a "scooping protection" policy, whereby similar findings that are published by others during review or revision are not a criterion for rejection. Should you decide to submit a revised version, I do ask that you get in touch after three months if you have not completed it, to update us on the status.

Please note: When submitting your revision you will be prompted to enter your funding and payment information. This will allow Wiley to send you a quote for the article processing charge (APC) in case of acceptance. This quote takes into account any reduction or fee waivers that you may be eligible for. Authors do not need to pay any fees before their manuscript is accepted and transferred to the publisher.

EMBO Press participates in many Publish and Read agreements that allow authors to publish Open Access with reduced/no publication charges. Check your eligibility: https://authorservices.wiley.com/author-resources/Journal-Authors/open-access/affiliation-policies-payments/index.html

***** Reviewer's comments *****

Referee #1 (Remarks for Author):

The work of F.Florio et al addresses the role of complement subunits in modulating fibrosis during skeletal muscle regeneration in a mdx mouse model deficient for the Dystrophin protein, responsible for Duchenne myopathy in humans. Since the mouse model of this disease does not recapitulate the exacerbated inflammatory aspects observed in humans, an mdx mouse model with daily needle micro-injuries extends the study. The authors try to understand the involvement of distinct complement subunits in the origin of the fibrosis observed in the pathological models studied. They show that C1 subunits are expressed in injured and mdx muscles mainly by macrophages (C1qa/qb/qc) and FAPS (C1r/s). Activation of the canonical WNT pathway leading to nuclear accumulation of beta catenin can occur by cleavage of LRP6 by C1 complement. It is known that activation of the canonical WNT pathway promises fibrosis of muscle tissue, and the authors attempted to relate fibrosis/WNT to complement activation. Overall, the results presented are robust and support a role for the complement, and the identification of the cellular origin of the complement subunits involved, in the observed fibrosis. My major point of criticism is the suspected requirement of the canonical Wnt pathway in this process, which is not as well demonstrated at the cellular FAPS level.

Major points
1- Figure S1B is a magnification of a region of the gastrocnemius muscle of 5-month-old mdx mice. It is important to show a larger scale image, a transversal section of the entire gastrocnemius muscle at multiple levels, and then zoom in to better realize the frequency of the reported observation. Sections of control muscle should also be shown to demonstrate the specific labeling in the mdx model.

2- Figure S6C is the only one that shows beta-catenin labeling, and the labeling shown shows canonical WNT pathway activation in macrophages. If the authors want to convince of the importance of the involvement of this pathway in FAPS activation, it is required to demonstrate AXIN2/Beta catenin axis in FAPS PDGFR-alpha positive cells. The link between the Wnt pathway activated in vivo in FAPS and fibrosis deserves more than an additional figure. Why doesn't Figure 1G show serial sections? This would be more informative than sections of human tissue that are not at the same level and do not allow visualization of whether the AXIN2+ cells are the ones expressing TGFbeta2.
3- In Figure 3, the culture of FAPS, rather than myoblasts, in the presence of C1complement proteins in the presence or absence of XAV-939, should allow the characterization of the robust activation of TGFbeta2 and other profibrotic genes (Collagens, Fibronectin) expression and directly demonstrate in FAPS the involvement of the WNT pathway in this process.

Referee #2 (Remarks for Author):

In this interesting work, the authors study the effect of C1 in fibrosis in Duchenne muscular dystrophy, using mouse models and samples from patients. The authors conclude that 1) there is enhanced local production of the C1 complex by macrophages and fibroadipogenic progenitors (FAPs) in dystrophic muscles; 2) this is linked with enhanced WNT-signaling and activation of a fibrogenic program in FAPs; and 3) C1 complement contributes to the etiology of fibrosis in the muscle affected by DMD. This is a study of special relevance to the neuromuscular field, and I commend the authors for the elegant design, clarity of writing, and the promising results, which have great potential for translation into the clinic. However, there are some issues that need to be resolved before publication:

1) The statistical analysis is one of the main issues I had with this work: It needs to be clearly stated in each piece of data within the figure legend. Key information is missing from most of the analyses performed: normality tests, post hoc tests after ANOVA, type of Student-t test (paired, unpaired, ratio...), etc. Some data was not correctly analyzed (i.e., Fig 2G Mann whiney test was erroneously used for more than 2 groups). Also, inconsistent analysis with the same type of data (Fig2, WT vs mdx MACs statistical analysis was not shown in some graphs).

2) Data visualization: For consistency, please, present data in a similar manner (Fig 2F vs 2G; Fig 3J vs 3L; Fig5B vs 5D; Fig5F-K vs Fig6B-F), and avoid using fold change over each control (fold over mean is acceptable) when this is not necessary (Fig 3L, Fig5D, Fig6B-F).

3) There are packages of experiments in which analyses were performed in a different way, hindering proper comparison and data interpretation (i.e., Fig. 3 J-M were performed at different times, and in different media). Please justify and include proper controls.

4) qPCR: DCT method is only suitable for similar primer efficiencies. Thus, this must be verified by using standard curves or all the primers involved in these analyses. Also, only one endogenous was used for all the samples, so they need unchanged endogenous levels among all the samples and different conditions must be verified. Alternatively, 2-3 additional endogenous genes may be used for normalization.

5) I do not fully agree with the following sentence in line 135: "This observation suggests that the events leading to increased WNT-activity in dystrophic muscles differ from those occurring in aging tissues and likely do not depend on the release of complement C1 or other WNT activators in the circulation". Please, state if there is any previous evidence of increased C1 or Wnt signaling in the mdx mouse at 7 months of age. Further experiments in muscles were performed in older animals (1-year-old), but serum was not evaluated for C1 complement levels at 1 year of age.

6) Further experiments or analysis should be performed:

a. Verify Axin2 upregulation (qPCR) in mdx mouse and DMD samples (Figures 1 c, d, Line 151).
b. Different mouse muscles should be tested (i.e., quad, diaphragm, heart...), and ideally at different time points (1-7 months, 1 year), to evaluate the effect of C1/WNT axis in the disease progression, as stated in the hypothesis (line 115). Also, it would be very interesting to find out how early in the disease is this event (C1/Wnt activation). Muscles showing early fibrosis should be used for this analysis (i.e. diaphragm)
c. Data from Fig 1 sections should be quantified, and statistical analysis performed. Western blots are preferred for protein quantification. How many patients were analyzed?
d. Include uninjured WT analysis in the analysis of the proliferation marker E2F1 (Fig2S D) and CD31 analysis in Fig 2F.
e. It would be interesting to evaluate the effect of C1 in macrophages, in the same way as performed for fibroblasts and myoblasts (Fig 3)

7) Some concepts should be further clarified:

- In the introduction expand on the following: fibrogenic features of dystrophic MuSC (lines 69 & 87), type of collagens in FAPs upon injury and in the mdx mouse (lines 73 & 83), and mechanisms facilitating TGFb activation of Wnt signaling pathway (line 93). Also, previous evidence of increased wnt signaling in Duchenne patients has already been reported (Liu et al., 2016, https://doi.org/10.3988/jcn.2016.12.3.351). Please, include this or other relevant references in line 82.
- In the Results section, clarify which cell types correspond to Lin-veSca1-veVCAM-ve cells, Lin+veF4/80-ve cells (line 161), and specify the muscles analyzed in mouse hindlimbs.
- Positive and negative controls need to be clearly stated in each figure. For instance, I understand that positive control in Fig3K is Wnt3A, but is it the same concentration as Wnt3A+XAV? What is the control solution in Fig3M?
- In line 183, it is not clear what is being compared, and the text does not correspond with the statistical analysis shown in the figures (Fig2A-C). The post hoc test used needs to be stated.
- Line 195, does not seem to refer to the appropriate figures.
- Please, better explain the model (Figure 6K) at the end of the results.
- TGFb: In view of these results, it would be interesting to further discuss the implication of TGFb in dystrophy, in relation to C1 levels and Wnt signaling pathway. Also, which might be the main TGFb-producing cell in the muscle.

Referee #3 (Remarks for Author):

The study by Florio et investigates the interplay between FAPs and macrophages in dystrophic muscle and the role of their interdependence to produce C1 complex that induces fibrogenesis by FAPs. The study is very novel and highly interesting, especially regarding the lack of current information concerning the interplay of these two cells types that are involved in the pathogenesis of chronic inflammation and fibrosis in dystrophic muscle. The study is carefully written and the data are convincing.
Nevertheless, the study would benefit from additional experiments to increase its impact.

The main concern is the importance and the specificity of the FAP/MAC interplay in fibrogenesis. It appears from data in Fig2 that the C1 elements are already expressed by the cells during normal regeneration and that their expression is increased in mdx. In their manuscript, the authors talked about "regenerating areas" in mdx. These areas should be better define, since later in the manuscript, there is a shift toward "fibrogenesis". It is suggested that the interplay between MACs and FAPs should be compared (not for all the experiments, but the essential ones) in mdx, fib/mdx and regenerating muscle. It will allow to define whether this pathway may be beneficial to a certain extent, or that , alternatively, its chronic, or increased, expression is detrimental for the muscle. For instance what happens if C1s/r inhibitors are given during regeneration of normal muscle? Is it beneficial or detrimental? This would show the specificity of the mechanism in fibrosis (versus ECM remodeling).

Figure 4 is not very informative. Indeed, it was previously shown by several publications that FAPs and macrophages are close to each other, and notably in the areas of high inflammation and fibrosis. In such areas, a very high cellularity is observed. In A-C panels, it would be interesting to compare with regenerating muscle, for instance at day 4 after CTX injury, and not resting muscle. In D,E panels, since a high cellularity is observed, the intensity is mathematically expected to raise, especially using epifluorescence acquisition (was a confocal microscope used?).

Figure 5 should show in the fib-mdx model the increase of the C1 components by MAC and FAPs. Indeed the increase in the number of FAPs and MAC has been already reported previously (Lemos Nat Med 2015, Juban Cell Rep 2018). Also the increase of expression of fibrogenic genes by FA¨s is expected in fib-mdx muscles.

Figure 5 : histology (eg HE staining) of several muscles of the hind limb should be presented as it is the first time that needle injury is performed on the entire limb (as supplemental data for instance).

Figure 3 : these results are interesting, but finally, they seem quite lost in the final big picture, since there is no evaluation of MuSCs in vivo in the following figures (at least Wnt signaling evaluation). How the authors put the MuSCs back in the FAP/MAC interplay on FAP fibrosis (Fig6K)?

How were the regenerating areas defined? In regenerating muscle, areas containing high density of macrophages are regenerating areas while in dystrophic muscle they can be both associated with regeneration and with fibrosis in mouse and human (Juban Cell Rep 2018).

Figure 5 and 6, notably Figure 6: outcome of fibrogenesis by FAPs should be evaluated at the protein level, and not through solely RTqPCR.

Macrophages have been shown to exhibit a mixed phenotype in fib-mdx (Lemos 2015). Are macrophages expressing C1 components of the pro-inflammatory or anti-inflamamtory phenotype? Since pro-inflammatory MACs are fibrotic in fib-mdx, this information may add insights on the interplay between the two cell types.

Minor:

Fig 1G, H, I not at the same magnification (3 magnifications)

Line 223 : Fig3e-G should be 3e-F

Line 224 : (Fig. 3A-F, H-M) . H-M does not refer to C1 expression

How was the distance between FAPs and MACs calculated? from edge to edge or from nucleus to nucleus?

In the methods, authors indicate that "the percentage of the interstitial fibrotic area was calculated by subtracting the total myofibers area from the total muscle area". This approach sounds not robust. Indeed, interstitial space is not only collagen and, depending on the stiffness of the muscle, be more or less altered by the cutting of the sections. Thus collagen are deposition is

preferred.

Dear Reviewers and Members of the Editorial board,

We thank the Reviewers for expressing interest in our work and giving suggestions to improve it. Based on the three Reviewer's constructive comments, we have made extensive revisions of the manuscript in terms of additional experiments, organization of the workflow and reworking of the discussion.

Specifically, by following the suggestions that we have received, we have significantly extended the characterization of WNT-signaling in FAPs, we introduced a quantification of complement proteins to confirm our previous qualitative histological analysis, we have performed additional experiments to get insight into the heterogeneity of macrophages in terms of C1q expression, we have expanded our initial observations suggesting differences between acute and chronic (i.e., dystrophy-associated) injury in terms of activation of the C1/WNT axis, we repeated histological quantifications by using confocal microscopy, we added requested additional controls to the experiments, and expanded the introduction and discussion to include all the aspects highlighted by the Reviewers. We have listed below a detailed point-by-point response to all the comments (in blue). We hope to have answered exhaustively to all the points raised by the Reviewers.

In our opinion, the changes we have introduced have largely improved the manuscript, and we hope you will agree and consider it suitable for publication.

POINT-BY-POINT RESPONSE TO THE COMMENTS

Referee #1 (Remarks for Author):

The work of F.Florio et al addresses the role of complement subunits in modulating fibrosis during skeletal muscle regeneration in a mdx mouse model deficient for the Dystrophin protein, responsible for Duchenne myopathy in humans. Since the mouse model of this disease does not recapitulate the exacerbated inflammatory aspects observed in humans, an mdx mouse model with daily needle micro-injuries extends the study. The authors try to understand the involvement of distinct complement subunits in the origin of the fibrosis observed in the pathological models studied. They show that C1 subunits are expressed in injured and mdx muscles mainly by macrophages (C1qa/qb/qc) and FAPS (C1r/s). Activation of the canonical WNT pathway leading to nuclear accumulation of beta catenin can occur by cleavage of LRP6 by C1 complement. It is known that activation of the canonical WNT pathway promises fibrosis of muscle tissue, and the authors attempted to relate fibrosis/WNT to complement activation. Overall, the results presented are robust and support a role for the complement, and the identification of the cellular origin of the complement subunits involved, in the observed fibrosis. My major point of criticism is the suspected requirement of the canonical Wnt pathway in this process, which is not as well demonstrated at the cellular FAPS level.

We thank the Reviewer for revising our paper and for the constructive suggestions.

Major points

1- Figure S1B is a magnification of a region of the gastrocnemius muscle of 5-month-old mdx mice. It is important to show a larger scale image, a transversal section of the entire gastrocnemius muscle at multiple levels, and then zoom in to better realize the frequency of the reported observation. Sections of control muscle should also be shown to demonstrate the specific labeling in the mdx model.

Following the Referee's suggestion, Figure S1B from the previous version of the paper has been substituted by **Figure EV2**, which shows both *WT* and *MDX* muscle sections. Multiple

high- and low-magnification images highlight the deposition of both C4 and C1q in the interstitial spaces of the *MDX* muscles.

2- Figure S6C is the only one that shows beta-catenin labeling, and the labeling shown shows canonical WNT pathway activation in macrophages. If the authors want to convince of the importance of the involvement of this pathway in FAPS activation, it is required to demonstrate AXIN2/Beta catenin axis in FAPS PDGFR-alpha positive cells. The link between the Wnt pathway activated in vivo in FAPS and fibrosis deserves more than an additional figure. Why doesn't Figure 1G show serial sections? This would be more informative than sections of human tissue that are not at the same level and do not allow visualization of whether the AXIN2+ cells are the ones expressing TGFbeta2.

We thank the Reviewer for having highlighted this aspect. We have now significantly expanded the study of the WNT-signaling pathway in dystrophic muscles, specifically in the FAPs, both at gene and protein levels. New data are enclosed in the following figures/panels:

Figure 1A-F and **Figure EV1C-H** show the increased expression of WNT (and collagen-related) genes in *MDX* FAPs compared to *WT* FAPs at two different ages of mice (i.e., 1 year-old in Figure 1A-F and 3 months-old in Figure EV1C-H).

Figure EV1A-B show the increased expression of WNT target proteins (i.e., Axin2 in A and TGFβ2 in B) in *MDX* PDGFRα^{+ve} FAPs compared to the *WT*.

Figure 1M shows serial sections of CNTR and dystrophic human muscles. The areas enriched in WNT-target proteins (i.e., Axin2 and TGFβ2) are also enriched in complement (i.e., C1s).

3- In Figure 3, the culture of FAPS, rather than myoblasts, in the presence of C1complement proteins in the presence or absence of XAV-939, should allow the characterization of the robust activation of TGFbeta2 and other profibrotic genes (Collagens, Fibronectin) expression and directly demonstrate in FAPS the involvement of the WNT pathway in this process.

We have expanded our study of the C1/WNT axis in muscles' FACS-isolated FAPs. In the experiment shown in **Figure 3O,** muscle-isolated FAPs (which produce C1r/s – see Figure 2D – E) were cultured in the presence of C1q and C1q+XAV-939 and the transcription of the WNT-target Axin2 was quantified. Axin2 expression was increased in the presence of C1q but was unchanged when C1q was added together with XAV-939. A similar result was also obtained when we analyzed the expression of the pro-fibrotic WNT-target TGFβ2 (**Additional Figure 1A**, attached to this document).

Referee #2 (Remarks for Author):

In this interesting work, the authors study the effect of C1 in fibrosis in Duchenne muscular dystrophy, using mouse models and samples from patients. The authors conclude that 1) there is enhanced local production of the C1 complex by macrophages and fibroadipogenic progenitors (FAPs) in dystrophic muscles; 2) this is linked with enhanced WNT-signaling and activation of a fibrogenic program in FAPs; and 3) C1 complement contributes to the etiology of fibrosis in the muscle affected by DMD. This is a study of special relevance to the neuromuscular field, and I commend the authors for the elegant design, clarity of writing, and the promising results, which have great potential for translation into the clinic. However, there are some issues that need to be resolved before publication:

We thank the Reviewer for revising our paper, for the positive feedback and for the constructive suggestions.

1) The statistical analysis is one of the main issues I had with this work: It needs to be clearly stated in each piece of data within the figure legend. Key information is missing from most of the analyses performed: normality tests, post hoc tests after ANOVA, type of Student-t test (paired, unpaired, ratio...), etc. Some data was not correctly analyzed (i.e., Fig 2G Mann whiney test was erroneously used for more than 2 groups). Also, inconsistent analysis with the same type of data (Fig2, WT vs mdx MACs statistical analysis was not shown in some graphs).

We apologize for not having exhaustively detailed the performed statistical analyses. The statistical approach used in this work is the same used in many other published studies in the same field (i.e., muscle biology, specifically studies on FAPs in mice: (Mueller et. al, 2017 in Nature, doi: 10.1038/nature20160, Biferali et. al, 2021 in Science Advances, doi: 10.1126/sciadv.abd9371, Madaro et. al, 2019 in Nature Cell Biology, doi: 10.1038/s41556-018-0151-ym, Heredia et. al, 2013 in Cell, doi: 10.1016/j.cell.2013.02.053). Now, the manuscript has been updated following EMBO Molecular Medicine guidelines. The revised version of the manuscript provides complete information on the statistical analysis performed for each piece of data. All the information is now enclosed in figure legends, Table 2 and Table 3 (in the "Appendix" file), and in the "Study design and statistical analysis" paragraph in the Material and Methods section.

We can add here additional details concerning the figures mentioned by the Referee. Specifically, the statistical differences were calculated in the updated version of Figure 2G (**Appendix Figure S6H** in the new version) by the Kruskal-Wallis test, Dunn's multiple comparison test was used as a *post hoc* test. With the analysis presented in **Figure 2A-E** and in **Appendix Figure S7A** (Figure S2D in the previously submitted version), we tried to address two different biological questions: (1) Which cell type in the muscle produces the highest level of complement subunits/E2F1? (2) Within the two different cell types that produce the highest levels of complement in the muscle (i.e., MACs for C1qa, C1qb and C1qc and FAPs for C1r and C1s), is there a differential expression in *WT* muscles vs *WT*

INJ, *WT* vs *MDX* and *MDX* vs *WT* INJ? In order to answer the first question, we have performed a one-way ANOVA test among different cell type groups in each different biological set of samples (i.e., *WT*, *WT* INJ, and *MDX*). Tukey's multiple comparison test was used as a *post hoc* test to compare every mean to every other mean. We have enclosed the complete results of this analysis and the corresponding p values in Table 2 and Table 3 in the "Appendix", and the manuscript refers to these tables in the text (lines 189, 191, 256, 257, 954). In order to answer the second question, the statistical analysis was performed between two groups at the time (i.e., MACs in *WT* vs MAC in *MDX* for C1qa, C1qb, C1qc; FAPs in *WT* vs FAPs in *MDX* for C1r, C1s, etc.) and statistical differences were calculated by unpaired two-tailed Student's test. The corresponding p values are reported in the figure, and the text of the legend has been updated. We apologize for the confusion that this figure might have generated while attempting to deliver a clear message of the results without showing graphs too full of potentially confounding elements (i.e., p value of all performed comparisons).

2) Data visualization: For consistency, please, present data in a similar manner (Fig 2F vs 2G; Fig 3J vs 3L; Fig5B vs 5D; Fig5F-K vs Fig6B-F), and avoid using fold change over each control (fold over mean is acceptable) when this is not necessary (Fig 3L, Fig5D, Fig6B-F).

Following the Referee's indications, we have updated the figures as follows:

Appendix Figure S6G vs 6H (Fig.2F vs. 2G in the previous version), **Figure 3K vs 3M** (Fig. 3J vs. 3L in the previous version), **Appendix Figure S13C vs. 13D** (Fig.5B vs. 5D in the previous version), **Fig.5J-N vs. Fig.6B-F** (Fig.5F-K vs. Fig.6B-F in the previous version) now show data in a similar manner and do not show data expressed as fold change over each control.

3*) There are packages of experiments in which analyses were performed in a different way, hindering proper comparison and data interpretation (i.e., Fig. 3 J-M were performed at different times, and in different media). Please justify and include proper controls.*

The initial experiment (reported in Fig. 3G and 3H in the current version) was performed at multiple time-points (6 and 24 hours) with the goal of identifying the optimal experimental conditions to disclose the activation of the WNT-signaling. Notably, similar results were obtained at the two time-points, suggesting that this type of assay could be performed without significant differences in the 6-24 hours time-window. For this reason, we decided to perform all the subsequent experiments at time-points between 6 and 24 hours, leaving the precise definition of the time-point to laboratory practicalities.

For each experiment, negative control ($CNTR^{-ve}$) consists of the cells' medium plus the solutions in which inhibitors/reagents were resuspended. If no inhibitors and reagents were used (i.e., Fig. 3K of the revised paper, which shows an experiment performed with

conditioned media), a CNTR^{-ve} consisting uniquely of the medium was added to the experiment. This is now clearly stated in the legends.

4) qPCR: DCT method is only suitable for similar primer efficiencies. Thus, this must be verified by using standard curves or all the primers involved in these analyses. Also, only one endogenous was used for all the samples, so they need unchanged endogenous levels among all the samples and different conditions must be verified. Alternatively, 2-3 additional endogenous genes may be used for normalization.

Both mHPRT and hHPRT primers used as reference genes in this study have been previously validated and used as normalizer genes in the muscle field in published papers (i.e., Biressi et. al, 2014; Florio et al. 2022). The efficiency of all the primers used in our analyses was validated. It is similar and ≥ 96%. This is now indicated in the Material and Methods section. Melt curves of all primers show a single prominent amplicon with melt peaks between 75 and 85°C.

As suggested, we have expanded our study using other normalizers in experiments with both human and mouse-derived samples (see examples in **Additional Figures 2-5** attached to this document). Our analysis shows that 1) When using a different normalizer gene (i.e., 36B4) the observed differences (and unchanged gene expression) are consistent with the ones obtained using HPRT and presented in the paper; 2) the expression of different endogenous genes (i.e., GAPDH in **Additional Figure 2**, Csnk2a2 in **Additional Figure 3**, ACTB in **Additional Figure 4-5**) does not change in CNTR vs. DMD (**Additional Figure 2**), *WT* vs. *WT*-INJ, *WT* vs. *MDX*, *MDX* vs. *WT*-INJ (**Additional Figure 3**), CNTR vs. C1r/s INH (**Additional Figures 4-5**) providing further reliability to our data.

5) I do not fully agree with the following sentence in line 135: "This observation suggests that the events leading to increased WNT-activity in dystrophic muscles differ from those occurring in aging tissues and likely do not depend on the release of complement C1 or other WNT activators in the circulation". Please, state if there is any previous evidence of increased C1 or Wnt signaling in the mdx mouse at 7 months of age.
Further experiments in muscles were performed in older animals (1-year-old), but serum was not evaluated for C1 complement levels at 1 year of age.

We thank the Reviewer for the suggestion. We have now evaluated the serum of 1 year-old mice for C1q and found no increase compared to the serum collected from age-matched *WT* mice. We have also evaluated C1s levels and found only a trend toward an increase in dystrophic serum compared to *WT*. These data are now enclosed in **Appendix Figure S2A, B** and discussed in the Discussion section. Being both C1q and C1s required for fruitful WNT-signaling activation (Naito et al. 2012), we believe that our data now support the statement in lines 155-158 of the revised version: “These data suggest that the events leading to increased WNT-activity in dystrophic muscles differ from those occurring in aging tissues and likely do not depend on the release of complement C1 in the circulation”.

6*) Further experiments or analysis should be performed:*

a. Verify Axin2 upregulation (qPCR) in mdx mouse and DMD samples (Figures 1 c, d, Line 151).

Following both Referee 1 and Referee 2 suggestions, we have expanded the study of the WNT-signaling pathway in dystrophic muscles, both at gene and protein levels. Axin2 qPCR on whole muscle extract is confounded by the expression of Axin2 in a subset of muscle fibers (Huraskin et al. Development 2016;143(17):3128-42; PMID: 27578179). Therefore, we have investigated Axin2 (and other markers of activated WNT-signaling) on purified cells or by performing quantitative histological analyses, which allow for the exclusion of the myofibers from the analysis. New data are enclosed in the following figures/panels:

Figure 1A-F and **Figure EV1C-H** show the increased expression of WNT and collagen-related genes in *MDX* FAPs compared to *WT* FAPs at two different ages (i.e., 1 year-old mice in Fig. 1A-F, and 3 months-old mice in Fig. EV1C-H).

Figure EV1A-B show the increased expression of WNT target proteins (i.e., Axin2 in A and TGFβ2 in B) in *MDX* PDGFRα^{+ve} FAPs compared to the *WT*.

Appendix Figure S1A (already present in the previously submitted version of the paper) shows that the WNT-target protein LGR5 expression is increased in *MDX* gastrocnemius and diaphragm compared to *WT*.

Appendix Figure S1B, C shows that the expression of the WNT target proteins Axin2 and TGFβ2 is increased in DMD muscles compared to healthy CNTR (human).

b. Different mouse muscles should be tested (i.e., quad, diaphragm, heart...), and ideally at different time points (1-7 months, 1 year), to evaluate the effect of C1/WNT axis in the disease progression, as stated in the hypothesis (line 115). Also, it would be very interesting to find out how early in the disease is this event (C1/Wnt activation). Muscles showing early fibrosis should be used for this analysis (i.e. diaphragm)

We have evaluated C1 complex components (i.e., C1q and C1s) protein levels in different muscles at different time points (i.e., ~1 month-old, ~3 months-old and ~1 year-old *WT* and *MDX* mice). Data are reported in **Additional Figure 6** (attached to this document).

c. Data from Fig 1 sections should be quantified, and statistical analysis performed. Western blots are preferred for protein quantification. How many patients were analyzed?

We have quantified data from Figure 1 (of the previously submitted version of the paper) sections, performed both western blot and immunofluorescence analysis according to the availability of human samples, and performed statistical analysis. For each analysis, at least 3 patients and 3 controls were used.

Data are enclosed in the following figures/panel:

Figure 1K of the revised paper (previously Figure 1E) and **Additional Figure 6A** attached to this document show C1q protein quantification in *WT* and *MDX* muscles.

Appendix Figure S3A (previously Figure 1F) and **Additional Figure 6B** attached to this document show C1s quantification in *WT* and *MDX* muscles.

Figure 1L shows C1s protein quantification (western blot) in CNTR and DMD human samples.

Appendix Figure S1B-C (previously Figure 1H-I) show Axin2 and TGFβ2 protein quantification in CNTR and DMD human samples.

d. Include uninjured WT analysis in the analysis of the proliferation marker E2F1 (Fig2S D) and CD31 analysis in Fig 2F.

Appendix Figure S7 (previously Supplementary Figure 2D) has been updated and now includes the analysis of the proliferation marker E2F1 in *WT* uninjured cells.

Unfortunately, due to the unavailability of fresh human biopsies (frozen biopsies are unsuitable for FACS-isolation of CD31 cells), we could not perform a detailed analysis of CD31 cells in terms of C1q protein expression. Due to the difficulties in obtaining fresh human samples, we decided to give precedence to the evaluation of C1s (see Appendix Fig. S6H), which is the target of the pharmacological intervention (i.e., C1r/s INH) described in this project. We are confident that the Reviewer will recognize that a detailed investigation of C1q expression in CD31^{+ve} (i.e., endothelial cells) is not the main focus of this manuscript, nor the central point of the experiments reported in Figure 2F of the previous version (now Appendix Figure S6G).

e. It would be interesting to evaluate the effect of C1 in macrophages, in the same way as performed for fibroblasts and myoblasts (Fig 3)

We have evaluated the effect of C1 in macrophages through similar experiments to the ones performed for fibroblasts and myoblasts. Our observation in both RAW-264.7 and muscle-isolated macrophages suggests that in our experimental setting, the canonical WNT signaling is not induced by complement in these cells (**Additional Figure 7** attached to this document). In agreement with the working model and the biological hypothesis of this work (**Figure 6K** and **Appendix Figure S17** of the revised paper), we propose macrophages as sources of complement components (i.e., C1q) but not as cells directly responsive to the WNT/C1 pathway. FAPs, on the other side, act both as a source of complement components (i.e., C1r/C1s) as well as target cells of the WNT/C1 axis.

7) Some concepts should be further clarified:

- In the introduction expand on the following: fibrogenic features of dystrophic MuSC (lines 69 & 87), type of collagens in FAPs upon injury and in the mdx mouse (lines 73 & 83), and mechanisms facilitating TGFb activation of Wnt signaling pathway (line 93). Also, previous evidence of increased wnt signaling in Duchenne patients has already been reported (Liu et al., 2016, https://doi.org/10.3988/jcn.2016.12.3.351). Please, include this or other relevant references in line 82.

We thank the Reviewer for the suggestions. We have now expanded the relevant points and added the suggested references. Please see lines 76, 79, 86, 87, 92-95, and 100-102 of the revised version.

- In the Results section, clarify which cell types correspond to Lin-veSca1-veVCAM-ve cells, Lin+veF4/80-ve cells (line 161), and specify the muscles analyzed in mouse hindlimbs.

We now report in the legend of **Appendix Figure S4** that analysis has been performed by dissecting muscles from the distal hindlimb of *WT* and *MDX* mice. We also clarified in the text (lines 181-183 of the revised version) that $Lin^{+ve}F4/80^{-ve}$ cells correspond to endothelial cells and cells of hematopoietic lineage with the exclusion of mature macrophages and that $Lin^{-ve}Sca1^{-ve}VCAM^{-ve}$ cells correspond to all the mononucleated cells non included in the other gates.

- Positive and negative controls need to be clearly stated in each figure. For instance, I understand that positive control in Fig3K is Wnt3A, but is it the same concentration as Wnt3A+XAV? What is the control solution in Fig3M?

Following EMBO Molecular Medicine guidelines on figure legends ("*Experimental details should, where possible, be given in the Materials and Methods section, and not repeated in the figure legends.*") we have preferentially added relevant details to the Materials and Methods sections. When reagents were used at the concentration indicated in Materials and Methods, this information was not repeated in the figure legends. However, when reagents were used at different concentrations (i.e., WNT3A), the exact concentration has been reported in the figure legend for each piece of data.

Within the same experiment (i.e., Fig. 3K, now Fig. 3L), WNT3A was always used at the same concentration (i.e., 50 ng/ml in both WNT3A and WNT3A+XAV conditions).

For each experiment, negative control ($CNTR^{-ve}$) consists of the cell growth medium plus the solutions in which inhibitors/reagents were resuspended. If no inhibitors/reagents were used in specific experiments (i.e., Fig. 3K of the revised paper, which shows an experiment performed with conditioned media), $CNTR^{-ve}$ consists of cells' medium. This is now clearly stated in the legends.

- In line 183, it is not clear what is being compared, and the text does not correspond with the statistical analysis shown in the figures (Fig2A-C). The post hoc test used needs to be stated.

Both figure legends and the main text have been updated following EMBO Molecular Medicine guidelines. The revised version of the manuscript provides complete information on the statistical analysis performed for each piece of data. All the information is now enclosed in figure legends, Table 2, Table 3 (in the "Appendix" file), and in the "Study design and statistical analysis" paragraph in the Material and Methods section.

- Line 195, does not seem to refer to the appropriate figures.

We have now run through the paper, checking that the references to the figures are located in the appropriate position.

- Please, better explain the model (Figure 6K) at the end of the results.

We have now described in great detail the model in **Appendix Figure S17** of the revised version.

- TGFb: In view of these results, it would be interesting to further discuss the implication of TGFb in dystrophy, in relation to C1 levels and Wnt signaling pathway. Also, which might be the main TGFb-producing cell in the muscle.

Following this interesting input from the Referee, we have expanded the Discussion section of the manuscript addressing this point (lines 459-468). In addition, we have evaluated TGFβ2 gene expression in different types of muscle cells isolated from *MDX* dystrophic samples (**Appendix Figure S14G**). Within the analyzed cells, we found that FAPs and MuSCs robustly express TGFβ2, with FAPs being the main source of TGFβ2 (compared to $CD45^{+ve}$ and $CD31^{+ve}$ cells).

Referee #3 (Remarks for Author):

The study by Florio et investigates the interplay between FAPs and macrophages in dystrophic muscle and the role of their interdependence to produce C1 complex that induces fibrogenesis by FAPs. The study is very novel and highly interesting, especially regarding the lack of current information concerning the interplay of these two cells types that are involved in the pathogenesis of chronic inflammation and fibrosis in dystrophic muscle. The study is carefully written and the data are convincing. Nevertheless, the study would benefit from additional experiments to increase its impact.

We thank the Reviewer for revising our paper and for the positive feedback.

The main concern is the importance and the specificity of the FAP/MAC interplay in fibrogenesis. It appears from data in Fig2 that the C1 elements are already expressed by the cells during normal regeneration and that their expression is increased in mdx. In their manuscript, the authors talked about "regenerating areas" in mdx. These areas should be better defined, since later in the manuscript, there is a shift toward "fibrogenesis". It is suggested that the interplay between MACs and FAPs should be compared (not for all the experiments, but the essential ones) in mdx, fib/mdx and regenerating muscle. It will allow to define whether this pathway may be beneficial to a certain extent, or that, alternatively, its chronic, or increased, expression is detrimental for the muscle. For instance what happens if C1s/r inhibitors are given during regeneration of normal muscle? Is it beneficial or detrimental? This would show the specificity of the mechanism in fibrosis (versus ECM remodeling).
Figure 4 is not very informative. Indeed, it was previously shown by several publications that FAPs and macrophages are close to each other, and notably in the areas of high inflammation and fibrosis. In such areas, a very high cellularity is observed. In A-C panels, it would be interesting to compare with regenerating muscle, for instance at day 4 after CTX injury, and not resting muscle.

We thank the Reviewer for underlining the intriguing possibility of complement playing a different role during acute homeostatic injury and chronic dystrophy-associated injury. Following Reviewer’s inputs and suggestions, we have performed a series of new experiments. Now, we present a revised version of the manuscript that expands our analysis to regenerating muscles (i.e., *WT* muscles after acute injury). As suggested, the FAPs/MAC interplay was further studied with respect to fib-*MDX* and injured muscles. Moreover, we conducted a study administering C1r/s inhibitor *in vivo* to *WT* injured mice (similar to the study on fib-*MDX* mice). New data covering these points are enclosed in the following figures/panels:

Figure EV4A-D show the **i**nterplay of MACs and FAPs in *MDX*, fib-*MDX,* and regenerating muscles (4 days after cardiotoxin injury).

Figure EV5 and **Appendix Figure S16** show the results of our *in vivo* administration of C1r/s inhibitor in *WT* muscles after cardiotoxin injury. The experimental conditions were set with the aim of being similar to the C1r/s inhibition in fib-*MDX* mice (i.e., the length of the treatment with the C1r/s inhibitor corresponds to the time between the last needle injury and the moment of muscle dissection in the fib-*MDX* experiments). We have analyzed FAPs and MAC in these muscles with respect to complement subunits expression, WNT-signaling and

fibrogenesis (FAPs), inflammation, and macrophages heterogeneity (MAC). *WT* muscle sections after acute injury were also analyzed for collagen deposition, fibrotic area, and muscle fiber cross-sectional area.

Altogether, our data suggest that a different repertoire of macrophages is infiltrating dystrophic and acutely injured muscles with respect to C1q expression. C1r/s inhibition in acutely injured muscle does not appear to alter ECM production, suggesting that C1 is not exerting a pro-fibrotic role in that context. Intriguingly, both in acute and chronic injury, the administration of C1r/s inhibitors promotes an increase in fiber size. The exact mechanistic basis of this response remains to be explored.

Following the indication of the Reviewer, we have also rephrased the sentence where we define the "regenerative foci" (lines 301-302 of the current version).

In D,E panels, since a high cellularity is observed, the intensity is mathematically expected to raise, especially using epifluorescence acquisition (was a confocal microscope used?).

Figure 4D-E (of the revised paper) shows the same experiments as Figure 4D-E (of the previous version of the paper) repeated using a confocal microscope for image acquisition.

The results obtained with the two microscopes are largely overlapping.

Figure 5 should show in the fib-mdx model the increase of the C1 components by MAC and FAPs. Indeed the increase in the number of FAPs and MAC has been already reported previously (Lemos Nat Med 2015, Juban Cell Rep 2018). Also the increase of expression of fibrogenic genes by FAPs is expected in fib-mdx muscles.

As suggested, the expression of C1qa/b/c and C1r/s was evaluated in the MAC and FAPs of the fib-*MDX* mouse model compared to the *MDX*. Data are enclosed in **Figure 5E-I** and suggest an increased expression of C1 subunits by FAPs and MAC in the fib-*MDX* model.

Figure 5: histology (eg HE staining) of several muscles of the hind limb should be presented as it is the first time that needle injury is performed on the entire limb (as supplemental data for instance).

We have now added the H&E staining in the revised paper (**Appendix Figure S13A**). Gastrocnemius muscles from the fib-*MDX* mice appear to be profoundly affected, presenting large portions of degenerating tissue and inflammatory infiltration. An efficient degeneration has been previously demonstrated for another hindlimb muscle, the tibialis anterior (Desguerre *et al*, 2012).

Figure 3: these results are interesting, but finally, they seem quite lost in the final big picture, since there is no evaluation of MuSCs in vivo in the following figures (at least Wnt signaling evaluation). How the authors put the MuSCs back in the FAP/MAC interplay on FAP fibrosis (Fig6K)?

We have now evaluated both WNT-signaling and fibrosis-related gene expression in MuSCs after *in vivo* complement inhibition in the fib-*MDX,* and these data are enclosed in **Appendix Figure S15C-G**. We did not observe a shift in the fibrogenic program nor in the WNT-signaling activation in these cells following our complement inhibition in our experimental setting. While being both WNT and fibrosis-related genes overexpressed in fib-*MDX* FAPs compared to *MDX* (see Fig. 5J-N), WNT and fibrogenesis appear to be only partially activated in fib-*MDX* MuSCs compared to the *MDX* (see Appendix Figure S14A-E). Moreover, WNT activation by complement is mediated by the WNT coreceptor LRP5/6 (Naito et. al, 2012). We report here that in dystrophic muscles, LRP6 is mainly expressed by the FAPs compared to all other cell types (including MuSCs, which do not express high levels of LRP6 - see Appendix Figure S14F). We may speculate the possibility that MuSCs are less responsive to the C1/WNT axis in the fib-*MDX* mouse model and that different experimental settings and/or models might be useful to address their role in this pathway. We have further discussed these experiments in the Discussion section of this revised version of the manuscript.

How were the regenerating areas defined? In regenerating muscle, areas containing high density of macrophages are regenerating areas while in dystrophic muscle they can be both associated with regeneration and with fibrosis in mouse and human (Juban Cell Rep 2018).

We apologize for the confusion and thank the Reviewer for highlighting this point. We conducted our analyses in areas with high cellularization, and we have now clearly stated this in the manuscript (lines 301-302).

Figure 5 and 6, notably Figure 6: outcome of fibrogenesis by FAPs should be evaluated at the protein level, and not through solely RTqPCR.

We have added the analysis of FAPs at the protein level. Data are presented in the following figures/panels:

Figure 5B shows the dramatic alteration of muscle histology and deposition of interstitial collagen in the fib-*MDX* muscles compared to the *MDX,* and **Appendix Figure S13B** shows the accumulation of FAPs in the collagen-rich interstitium of these muscles.

Figure EV4I shows that the percentage of TGFβ2^{+ve} FAPs (i.e., PDGFRα^{+ve} cells) is increased in fib-*MDX* muscles compared to *MDX*.

With respect to the *in vivo* inhibition of complement, the intensity of the staining for TGFβ2 and the intracellular fibrotic marker HSP47 was reduced in FAPs in the muscles of the fib-*MDX* mice treated with the complement inhibitor (**Appendix Figure S15A, B**).

Macrophages have been shown to exhibit a mixed phenotype in fib-mdx (Lemos 2015). Are macrophages expressing C1 components of the pro-inflammatory or anti-inflamamtory phenotype? Since pro-inflammatory MACs are fibrotic in fib-mdx, this information may add insights on the interplay between the two cell types.

We than the Reviewer for raising this interesting observation. Following Reviewer's input, we have analyzed macrophages in *WT*, injured, dystrophic, and fib-*MDX* muscles with respect to complement expression and pro-inflammatory/anti-inflammatory phenotype. Data are enclosed in the following figures/panels:

Figure EV4E-H show TNFα/TGFβ1, TNFα/IL-10, CD206, and CD163 expression in *MDX* and fib-*MDX* macrophages.

Appendix Figure S16A-D show IL-10/TNFα, IL-10/TGFβ1, CD206, and CD163 expression in fib-*MDX* and *WT* injured (with and without C1r/s inhibition) macrophages.

Appendix Figure S9 shows the analysis of complement, CD163, CD206, and IL-10 expression in different subsets of macrophages in resting murine and human muscles (see also Appendix Figure S6 A-D).

Figure EV3 and **Appendix Figure S10** show both complement and inflammation/fibrotic markers expression in different subsets of macrophages in dystrophic and *WT* injured muscles.

Overall, our data indicate a preferential expression of C1q in a specific macrophage subpopulation dominating the dystrophic muscle and characterized by expression of CD206 and CD163 but low levels of the anti-inflammatory marker IL-10 (see Figure EV3D-H).

Minor:

Fig 1G, H, I not at the same magnification (3 magnifications)

Figure 1G, H, and I (of the previous version of the paper) has been replaced with **Figure 1M**, which shows serial sections of CNTR and dystrophic human muscles. Now, the images are all at the same magnification.

Line 223: Fig3e-G should be 3e-F
Line 224: (Fig. 3A-F, H-M). H-M does not refer to C1 expression.

We apologize for the confusion. We have now corrected it as suggested by the Reviewer.

How was the distance between FAPs and MACs calculated? from edge to edge or from nucleus to nucleus?

The distance between FAPs and MACs was calculated from nucleus-to-nucleus. This is now clearly stated in the text (Materials and Methods and Appendix Supplementary Methods

sections). To clarify this aspect, examples of the distance analysis are presented **Appendix Figure S11E-F** of the revised manuscript.

In the methods, authors indicate that "the percentage of the interstitial fibrotic area was calculated by subtracting the total myofibers area from the total muscle area". This approach sounds not robust. Indeed, interstitial space is not only collagen and, depending on the stiffness of the muscle, be more or less altered by the cutting of the sections. Thus collagen are deposition is preferred.

Following this suggestion, we have recalculated the percentage of the interstitial fibrotic area as the percentage of area with collagen 1 deposition over the total muscle area, and the analysis of new experiments presented in this revised version of our manuscript was performed using this approach. Data are shown in **Figure 6H** and **Figure EV5M** and are in line with the quantification of the interstitial space that we reported in the previous version.

Additional figures for reviewers have been removed.

4th Oct 2023

Dear Prof. Biressi,

Thank you for the submission of your revised manuscript to EMBO Molecular Medicine. I am pleased to inform you that we will be able to accept your manuscript pending the following final amendments:

1) In the main manuscript file, please do the following:
- Please address all comments suggested by our data editors listed below:
o Please indicate the statistical test used for data analysis in the legends of figures 4d-e.
o Please note that information related to n is missing in the legend of figures 2f-g; 4a, c.
o Although 'n' is provided, please describe the nature of entity for 'n' in the legends of figures 3a-c, e-f.
o Please note that the data citation does not refer to deposited experimental data, but refers to a journal article.
o Please note that the data callouts in the text for the data citation does not include "Data ref:" as a prefix.
- Data availability: This paragraph should contain information only about deposited data produced in this study. From what I could gather it seems that you have not performed any high-throughput experiments within the study but analyzed published datasets. Please clarify this and in case you only reanalyzed published datasets - remove these information from data availability paragraph, clearly indicate it in the text, and refer to the original manuscript and add data citation at an appropriate place in M&M section. Also, in that case please replace the current text with following sentence: This study includes no data deposited in external repositories.

Please check "Author Guidelines" for more information.
https://www.embopress.org/page/journal/17574684/authorguide#availabilityofpublishedmaterial
2) Appendix: Please add page numbers in ToC and move all supplementary methods to M&M section in the main manuscript file.
3) Tables: Please move all tables to "Appendix", label them Appendix Table S1 etc., and update their callouts in the main manuscript text.
4) Source data: Thank you for providing all requested source data. Please convert all .nd2 image files to .tiff. Zipp all source data files for EV figures and submit it as one file.
5) Synopsis:
- Synopsis image: Please label all the objects and different colors in the provided image. Synopsis image should be scientifically meaningful. Alternatively, you can use a striking image from one of the figures to illustrate your article. Please upload the image as a high-resolution jpeg file 550 px-wide x (250-400)-px high.
- Please check your synopsis text and image before submission with your revised manuscript. Please be aware that in the proof stage minor corrections only are allowed (e.g., typos).
6) For more information: This space should be used to list relevant web links for further consultation by our readers. Could you identify some relevant ones and provide such information as well? Some examples are patient associations, relevant databases, OMIM/proteins/genes links, author's websites, etc...
7) As part of the EMBO Publications transparent editorial process initiative (see our Editorial at http://embomolmed.embopress.org/content/2/9/329), EMBO Molecular Medicine will publish online a Review Process File (RPF) to accompany accepted manuscripts. This file will be published in conjunction with your paper and will include the anonymous referee reports, your point-by-point response and all pertinent correspondence relating to the manuscript. Let us know whether you agree with the publication of the RPF and as here, if you want to remove or not any figures from it prior to publication. Please note that the Authors checklist will be published at the end of the RPF.
8) Please provide a point-by-point letter INCLUDING my comments as well as the reviewer's reports and your detailed responses (as Word file).

I look forward to seeing a revised form of your manuscript as soon as possible.

Yours sincerely,

Zeljko Durdevic

Zeljko Durdevic
Editor
EMBO Molecular Medicine

*** Instructions to submit your revised manuscript ***

*** PLEASE NOTE *** As part of the EMBO Publications transparent editorial process initiative (see our Editorial at https://www.embopress.org/doi/pdf/10.1002/emmm.201000094), EMBO Molecular Medicine will publish online a Review Process File to accompany accepted manuscripts.

In the event of acceptance, this file will be published in conjunction with your paper and will include the anonymous referee reports, your point-by-point response and all pertinent correspondence relating to the manuscript. If you do NOT want this file to be published, please inform the editorial office at contact@embomolmed.org.

When submitting your revised manuscript, please include:

1) a .docx formatted version of the manuscript text (including Figure legends and tables)

2) Separate figure files*

3) supplemental information as Expanded View and/or Appendix. Please carefully check the authors guidelines for formatting Expanded view and Appendix figures and tables at https://www.embopress.org/page/journal/17574684/authorguide#expandedview

4) a letter INCLUDING the reviewer's reports and your detailed responses to their comments (as Word file).

5) The paper explained: EMBO Molecular Medicine articles are accompanied by a summary of the articles to emphasize the major findings in the paper and their medical implications for the non-specialist reader. Please provide a draft summary of your article highlighting
- the medical issue you are addressing,
- the results obtained and
- their clinical impact.
This may be edited to ensure that readers understand the significance and context of the research.
Please refer to any of our published articles for an example.

6) For more information: There is space at the end of each article to list relevant web links for further consultation by our readers. Could you identify some relevant ones and provide such information as well? Some examples are patient associations, relevant databases, OMIM/proteins/genes links, author's websites, etc...

7) Author contributions: the contribution of every author must be detailed in a separate section.

8) EMBO Molecular Medicine now requires a complete author checklist (https://www.embopress.org/page/journal/17574684/authorguide) to be submitted with all revised manuscripts. Please use the checklist as guideline for the sort of information we need WITHIN the manuscript. The checklist should only be filled with page numbers were the information can be found. This is particularly important for animal reporting, antibody dilutions (missing) and exact values and n that should be indicted instead of a range.

9) Every published paper now includes a 'Synopsis' to further enhance discoverability. Synopses are displayed on the journal webpage and are freely accessible to all readers. They include a short stand first (maximum of 300 characters, including space) as well as 2-5 one sentence bullet points that summarise the paper. Please write the bullet points to summarise the key NEW findings. They should be designed to be complementary to the abstract - i.e. not repeat the same text. We encourage inclusion of key acronyms and quantitative information (maximum of 30 words / bullet point). Please use the passive voice. Please attach these in a separate file or send them by email, we will incorporate them accordingly.

You are also welcome to suggest a striking image or visual abstract to illustrate your article. If you do please provide a jpeg file 550 px-wide x 400-px high.

10) A Conflict of Interest statement should be provided in the main text

11) Please note that we now mandate that all corresponding authors list an ORCID digital identifier. This takes <90 seconds to complete. We encourage all authors to supply an ORCID identifier, which will be linked to their name for unambiguous name

identification.

Currently, our records indicate that the ORCID for your account is 0000-0001-8631-3419.

Please click the link below to modify this ORCID:
Link Not Available

12) The system will prompt you to fill in your funding and payment information. This will allow Wiley to send you a quote for the article processing charge (APC) in case of acceptance. This quote takes into account any reduction or fee waivers that you may be eligible for. Authors do not need to pay any fees before their manuscript is accepted and transferred to our publisher.

*Additional important information regarding Figures

Each figure should be given in a separate file and should have the following resolution:
Graphs 800-1,200 DPI
Photos 400-800 DPI
Colour (only CMYK) 300-400 DPI"

Figures are not edited by the production team. All lettering should be the same size and style; figure panels should be indicated by capital letters (A, B, C etc). Gridlines are not allowed except for log plots. Figures should be numbered in the order of their appearance in the text with Arabic numerals. Each Figure must have a separate legend and a caption is needed for each panel.

*Additional important information regarding figures and illustrations can be found at https://bit.ly/EMBOPressFigurePreparationGuideline. See also figure legend preparation guidelines: https://www.embopress.org/page/journal/17574684/authorguide#figureformat

The system will prompt you to fill in your funding and payment information. This will allow Wiley to send you a quote for the article processing charge (APC) in case of acceptance. This quote takes into account any reduction or fee waivers that you may be eligible for. Authors do not need to pay any fees before their manuscript is accepted and transferred to our publisher.

***** Reviewer's comments *****

Referee #1 (Remarks for Author):

The manuscript by Florio et al has been greatly improved, and the authors have responded to most of the criticisms raised.

Referee #3 (Remarks for Author):

I thank the authors for their huge effort in providing so many new experiments and for clarifying all the issues I (and the other reviewers) raised.

The authors addressed the remaining editorial issues.

11th Oct 2023

Dear Prof. Biressi,

We are pleased to inform you that your manuscript is accepted for publication and is now being sent to our publisher to be included in the next available issue of EMBO Molecular Medicine.

Please read below for additional IMPORTANT information regarding your article, its publication and the production process.

Congratulations on your interesting work,

Zeljko Durdevic

Zeljko Durdevic
Editor
EMBO Molecular Medicine

Follow us on Twitter @EmboMolMed
Sign up for eTOCs at embopress.org/alertsfeeds

*** *** *** IMPORTANT INFORMATION *** *** ***

SPEED OF PUBLICATION
The journal aims for rapid publication of papers, using using the advance online publication "Early View" to expedite the process: A properly copy-edited and formatted version will be published as "Early View" after the proofs have been corrected. Please help the Editors and publisher avoid delays by providing e-mail address(es), telephone and fax numbers at which author(s) can be contacted.

Should you be planning a Press Release on your article, please get in contact with embomolmed@wiley.com as early as possible, in order to coordinate publication and release dates.

LICENSE AND PAYMENT:

All articles published in EMBO Molecular Medicine are fully open access: immediately and freely available to read, download and share.

EMBO Molecular Medicine charges an article processing charge (APC) to cover the publication costs. You, as the corresponding author for this manuscript, should have already received a quote with the article processing fee separately. Please let us know in case this quote has not been received.

Once your article is at Wiley for editorial production you will receive an email from Wiley's Author Services system, which will ask you to log in and will present you with the publication license form for completion. Within the same system the publication fee can be paid by credit card, an invoice, pro forma invoice or purchase order can be requested.

Payment of the publication charge and the signed Open Access Agreement form must be received before the article can be published online.

PROOFS

You will receive the proofs by e-mail approximately 2 weeks after all relevant files have been sent o our Production Office. Please return them within 48 hours and if there should be any problems, please contact the production office at embopressproduction@wiley.com.

Please inform us if there is likely to be any difficulty in reaching you at the above address at that time. Failure to meet our

deadlines may result in a delay of publication.

All further communications concerning your paper proofs should quote reference number EMM-2023-17405-V3 and be directed to the production office at embopressproduction@wiley.com.

Thank you,

Zeljko Durdevic
Editor
EMBO Molecular Medicine

EMBO Press Author Checklist

Corresponding Author Name: Stefano Biressi	
Journal Submitted to: EMBO Molecular Medicine	
Manuscript Number: EMM-2023-17405	

USEFUL LINKS FOR COMPLETING THIS FORM
The EMBO Journal - Author Guidelines
EMBO Reports - Author Guidelines
Molecular Systems Biology - Author Guidelines
EMBO Molecular Medicine - Author Guidelines

Reporting Checklist for Life Science Articles (updated January
This checklist is adapted from Materials Design Analysis Reporting (MDAR) Checklist for Authors. MDAR establishes a minimum set of requirements in transparent reporting in the life sciences (see Statement of Task: 10.31222/osf.io/9sm4x). Please follow the journal's guidelines in preparing your
Please note that a copy of this checklist will be published alongside your article.

Abridged guidelines for figures
1. Data
The data shown in figures should satisfy the following conditions:
- the data were obtained and processed according to the field's best practice and are presented to reflect the results of the experiments in an accurate and unbiased manner.
- ideally, figure panels should include only measurements that are directly comparable to each other and obtained with the same assay.
- plots include clearly labeled error bars for independent experiments and sample sizes. Unless justified, error bars should not be shown for technical
- if n<5, the individual data points from each experiment should be plotted. Any statistical test employed should be justified.
- Source Data should be included to report the data underlying figures according to the guidelines set out in the authorship guidelines on Data

2. Captions
Each figure caption should contain the following information, for each panel where they are relevant:
- a specification of the experimental system investigated (eg cell line, species name).
- the assay(s) and method(s) used to carry out the reported observations and measurements.
- an explicit mention of the biological and chemical entity(ies) that are being measured.
- an explicit mention of the biological and chemical entity(ies) that are altered/varied/perturbed in a controlled manner.
- the exact sample size (n) for each experimental group/condition, given as a number, not a range;
- a description of the sample collection allowing the reader to understand whether the samples represent technical or biological replicates (including how many animals, litters, cultures, etc.).
- a statement of how many times the experiment shown was independently replicated in the laboratory.
- definitions of statistical methods and measures:
  - common tests, such as t-test (please specify whether paired vs. unpaired), simple χ2 tests, Wilcoxon and Mann-Whitney tests, can be unambiguously identified by name only, but more complex techniques should be described in the methods section;
  - are tests one-sided or two-sided?
  - are there adjustments for multiple comparisons?
  - exact statistical test results, e.g., P values = x but not P values < x;
  - definition of 'center values' as median or average;
  - definition of error bars as s.d. or s.e.m.

Please complete ALL of the questions below.
Select "Not Applicable" only when the requested information is not relevant for your study.

Materials

Newly Created Materials	Information included in the manuscript?	In which section is the information available? (Reagents and Tools Table, Materials and Methods, Figures, Data Availability Section)
New materials and reagents need to be available; do any restrictions apply?	Not Applicable	

Antibodies	Information included in the manuscript?	In which section is the information available? (Reagents and Tools Table, Materials and Methods, Figures, Data Availability Section)
For **antibodies** provide the following information:<br>- Commercial antibodies: RRID (if possible) or supplier name, catalogue number and or/clone number<br>- Non-commercial: RRID or citation	Yes	Materials and Methods, Tables

DNA and RNA sequences	Information included in the manuscript?	In which section is the information available? (Reagents and Tools Table, Materials and Methods, Figures, Data Availability Section)
**Short novel DNA or RNA including primers, probes**: provide the sequences.	Yes	Materials and Methods, Tables

Cell materials	Information included in the manuscript?	In which section is the information available? (Reagents and Tools Table, Materials and Methods, Figures, Data Availability Section)
**Cell lines:** Provide species information, strain. Provide accession number in repository **OR** supplier name, catalog number, clone number, and/**OR** RRID.	Yes	Materials and Methods
**Primary cultures:** Provide species, strain, sex of origin, genetic modification status.	Yes	Materials and Methods
Report if the cell lines were recently **authenticated** (e.g., by STR profiling) and tested for mycoplasma contamination.	Yes	Materials and Methods

Experimental animals	Information included in the manuscript?	In which section is the information available? (Reagents and Tools Table, Materials and Methods, Figures, Data Availability Section)
**Laboratory animals or Model organisms:** Provide species, strain, sex, age, genetic modification status. Provide accession number in repository **OR** supplier name, catalog number, clone number, **OR** RRID.	Yes	Materials and Methods
**Animal observed in or captured from the field:** Provide species, sex, and age where possible.	Not Applicable	
Please detail **housing and husbandry conditions**.	Yes	Materials and Methods

Plants and microbes	Information included in the manuscript?	In which section is the information available? (Reagents and Tools Table, Materials and Methods, Figures, Data Availability Section)
**Plants:** provide species and strain, ecotype and cultivar where relevant, unique accession number if available, and source (including location for collected wild specimens).	Not Applicable	
**Microbes:** provide species and strain, unique accession number if available, and source.	Not Applicable	

Human research participants	Information included in the manuscript?	In which section is the information available? (Reagents and Tools Table, Materials and Methods, Figures, Data Availability Section)
If collected and within the bounds of privacy constraints report on age, sex and gender or ethnicity for all study participants.	Yes	Materials and Methods, Tables

Core facilities	Information included in the manuscript?	In which section is the information available? (Reagents and Tools Table, Materials and Methods, Figures, Data Availability Section)
If your work benefited from core facilities, was their service mentioned in the acknowledgments section?	Yes	Acknowledgments

Design

Study protocol	Information included in the manuscript?	In which section is the information available? (Reagents and Tools Table, Materials and Methods, Figures, Data Availability Section)
If study protocol has been **pre-registered, provide DOI in the manuscript**. For clinical trials, provide the trial registration number **OR** cite DOI.	Not Applicable	
Report the **clinical trial registration number** (at ClinicalTrials.gov or equivalent), where applicable.	Not Applicable	

Laboratory protocol	Information included in the manuscript?	In which section is the information available? (Reagents and Tools Table, Materials and Methods, Figures, Data Availability Section)
Provide DOI OR other citation details if **external detailed step-by-step protocols** are available.	Not Applicable	

Experimental study design and statistics	Information included in the manuscript?	In which section is the information available? (Reagents and Tools Table, Materials and Methods, Figures, Data Availability Section)
Include a statement about **sample size** estimate even if no statistical methods were used.	Yes	Materials and Methods
Were any steps taken to minimize the effects of subjective bias when allocating animals/samples to treatment (e.g. **randomization procedure**)? If yes, have they been described?	Yes	Materials and Methods
Include a statement about **blinding** even if no blinding was done.	Yes	Materials and Methods
Describe **inclusion/exclusion criteria** if samples or animals were excluded from the analysis. Were the criteria pre-established?<br><br>If sample or data points were omitted from analysis, report if this was due to attrition or intentional exclusion and provide justification.	Not Applicable	
For every figure, are **statistical tests** justified as appropriate? Do the data meet the assumptions of the tests (e.g., normal distribution)? Describe any methods used to assess it. Is there an estimate of variation within each group of data? Is the variance similar between the groups that are being statistically compared?	Yes	Materials and Methods, Figures Legends

Sample definition and in-laboratory replication	Information included in the manuscript?	In which section is the information available? (Reagents and Tools Table, Materials and Methods, Figures, Data Availability Section)
In the figure legends: state number of times the experiment was **replicated** in laboratory.	Yes	Figures Legends
In the figure legends: define whether data describe **technical or biological replicates**.	Yes	Figures Legends

Ethics

Ethics	Information included in the manuscript?	In which section is the information available? (Reagents and Tools Table, Materials and Methods, Figures, Data Availability Section)
Studies involving **human participants**: State details of **authority granting ethics approval** (IRB or equivalent committee(s), provide reference number for approval.	Yes	Materials and Methods
Studies involving **human participants**: Include a statement confirming that **informed consent** was obtained from all subjects and that the experiments conformed to the principles set out in the WMA Declaration of Helsinki and the Department of Health and Human Services Belmont Report.	Yes	Materials and Methods
Studies involving **human participants:** For publication of **patient photos**, include a statement confirming that consent to publish was obtained.	Not Applicable	
Studies involving experimental **animals**: State details of **authority granting ethics approval** (IRB or equivalent committee(s), provide reference number for approval. Include a statement of compliance with ethical regulations.	Yes	Materials and Methods
Studies involving **specimen and field samples:** State if relevant **permits** obtained, provide details of authority approving study; if none were required, explain why.	Not Applicable	

Dual Use Research of Concern (DURC)	Information included in the manuscript?	In which section is the information available? (Reagents and Tools Table, Materials and Methods, Figures, Data Availability Section)
Could your study fall under dual use research restrictions? Please check biosecurity documents and list of **select agents and toxins** (CDC): https://www.selectagents.gov/sat/list.htm	Not Applicable	
If you used a select agent, is the security level of the lab appropriate and reported in the manuscript?	Not Applicable	
If a study is subject to dual use research of concern regulations, is the name of the **authority granting approval and reference number** for the regulatory approval provided in the manuscript?	Not Applicable	

Reporting

The MDAR framework recommends adoption of discipline-specific guidelines, established and endorsed through community initiatives. Journals have their own policy about requiring specific guidelines and recommendations to complement MDAR.

Adherence to community standards	Information included in the manuscript?	In which section is the information available? (Reagents and Tools Table, Materials and Methods, Figures, Data Availability Section)
State if relevant guidelines or checklists (e.g., **ICMJE, MIBBI, ARRIVE, PRISMA**) have been followed or provided.	Not Applicable	
For **tumor marker prognostic studies,** we recommend that you follow the **REMARK** reporting guidelines (see link list at top right). See author guidelines, under 'Reporting Guidelines'. Please confirm you have followed these guidelines.	Not Applicable	
For **phase II and III randomized controlled trials**, please refer to the **CONSORT** flow diagram (see link list at top right) and submit the CONSORT checklist (see link list at top right) with your submission. See author guidelines, under 'Reporting Guidelines'. Please confirm you have submitted this list.	Not Applicable	

Data Availability

Data availability	Information included in the manuscript?	In which section is the information available? (Reagents and Tools Table, Materials and Methods, Figures, Data Availability Section)
Have **primary datasets** been deposited according to the journal's guidelines (see 'Data Deposition' section) and the respective accession numbers provided in the Data Availability Section?	Yes	Data Availability
Were **human clinical and genomic datasets** deposited in a public access-controlled repository in accordance to ethical obligations to the patients and to the applicable consent agreement?	Not Applicable	
Are **computational models** that are central and integral to a study available without restrictions in a machine-readable form? Were the relevant accession numbers or links provided?	Not Applicable	
If publicly available data were reused, provide the respective **data citations in the reference list**.	Yes	Main Text, References, Data Availability Section
